# Electrochemical Enantioselective Ruthenium(II)-Catalyzed C–H Activations to Atropostable Indoles and Chiral Spiropyrazolones

**DOI:** 10.1021/jacs.6c01678

**Published:** 2026-05-06

**Authors:** Siyu Liu, Jiawei Xu, Parveen Rawal, João C. A. Oliveira, Lutz Ackermann

**Affiliations:** Wöhler Research Institute for Sustainable Chemistry, 9375 Georg-August-Universität Göttingen, Göttingen 37077, Germany

## Abstract

Ruthenium­(II) catalysis is characterized by a remarkable diversity in C–H activation; however, compared to other transition metals, enantioselective C–H activations continue to be underdeveloped. Meanwhile, organic electrosynthesis has attracted considerable attention in recent years as a sustainable alternative to traditional redox approaches. Herein, we disclose an unprecedented electrochemistry-enabled ruthenium­(II)-catalyzed enantioselective C–H activation strategy to provide versatile access to atroposelective indoles as well as chiral spiroazoles. This approach combines the redox flexibility of ruthenium with the sustainable nature of molecular electrosynthesis, delivering the desired products in high chemical yields with excellent enantioselectivities, thereby paving the way for sustainable asymmetric syntheses.

## Graphical abstract

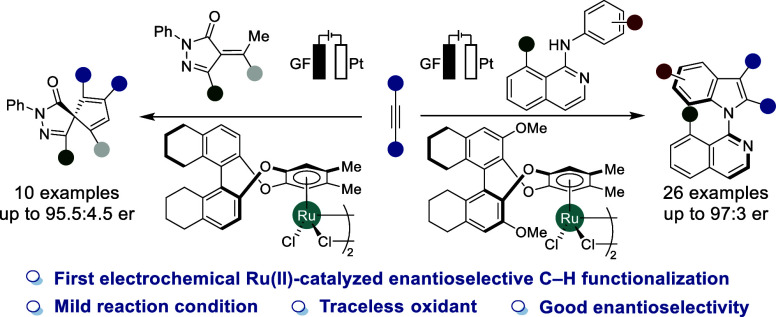

## Introduction

The development of transition metal-catalyzed asymmetric C–H activation represents a major advancement with major implications for modern molecular syntheses.^1^ This strategy enables the direct functionalization of inert C–H bonds in an enantioselective manner, providing a highly efficient and atom-economical approach to the construction of chiral molecules. In recent years, a series of asymmetric C–H activation reactions catalyzed by transition metals such as palladium,^2a,2b^ rhodium,^3^ and iridium^4^ have been successively reported, significantly expanding the synthetic toolbox for asymmetric catalysis. Inspired by early studies on stereoselective C–H activations by Murai, Sokolov,^2a,2b^ Yu, Colobert, Wencel–Delord, You, Shi, and others^2^ disclosed atroposelective palladium­(II)-catalyzed C–H activation. In this context, both You and Waldmann reported on rhodium­(III)-catalyzed C–H activation for the synthesis of atropisomers.^3b,3d^ Despite these advances, the development of ruthenium­(II)-catalyzed enantioselective C–H activation continues to be underdeveloped.^5^ Recent breakthroughs in this field were achieved through three main strategies: (1) exploiting the use of a chiral directing group by Cui,^6^ Wang,^7^ Sahoo,^8^ and Shi;^9^ (2) the merger of a chiral carboxylic acid (CCA) with an achiral ruthenium­(II) species by our group,^10^ Matsunaga,^11^ and Shi;^12^ and (3) a ruthenium­(II) catalyst bearing a chiral η^6^-arene ligand by Wang^13^ and Perekalin.^14^

Electrosynthesis has emerged as a powerful, sustainable approach to organic synthesis, offering a greener alternative to traditional redox chemistry with the aid of protons and electrons as traceless redox agents.^15^ In recent years, the merger of electrosynthesis with transition metal catalysis opened innovative avenues for the development of highly selective and atom-economical C–H functionalization reactions.^16^ Among several kinds of transition metals, ruthenium­(II) compounds hold unique advantages due to their relatively low costs, versatile redox properties, and proven capability in mediating diverse C–H activation, in particular annulation reactions under thermal conditions.^17^ Therefore, electrochemical ruthenium­(II)-catalyzed C–H annulation reactions attracted considerable attention, being widely developed by Xu, Zhong, and Ackermann, among others (Scheme 1a),^18^ especially for the synthesis of various heterocyclic structures.^19^ However, to date, enantioselective ruthena-electrocatalyzed C–H activations have proven elusive.

**Scheme 1:**
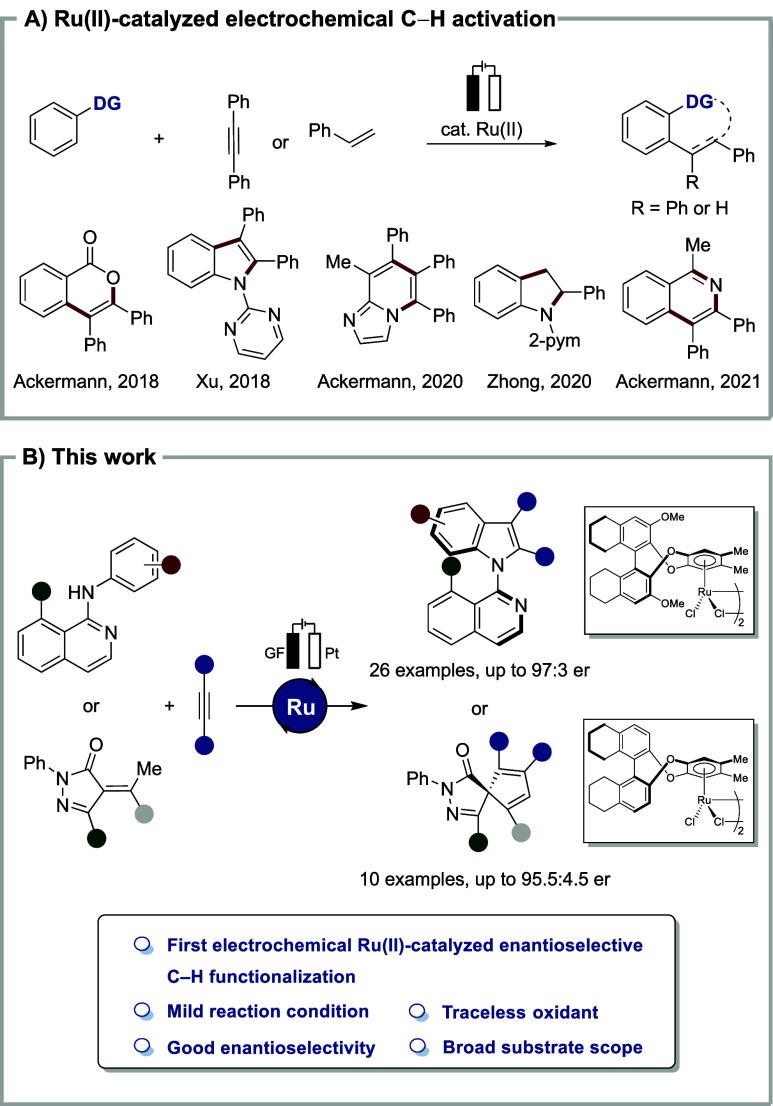
(A) Ruthena­(II)-Electrocatalyzed C–H Activation; (B) Enantioselective Ruthena­(II)-Catalyzed Electrochemical Assembly of Indoles and Spiropyrazolones (This Work)

Within our continued program on ruthenium­(II)-catalyzed C–H activation and electrocatalysis, we herein report on the unprecedented electrochemically driven chiral ruthenium­(II)-catalyzed asymmetric C–H activation. Our strategy thus enabled the enantioselective construction of C–N axial and central chirality, thereby allowing the efficient assembly of atropostable indoles and spiropyrazolones with high chemical and optical yields (Scheme 1b).

## Results and Discussion

We initiated our studies by optimizing the reaction conditions for the envisioned ruthenium­(II)-catalyzed electrochemical synthesis of atroposelective indoles (Table 1). Isoquinolin-1-amine **1a** and alkyne **2a** were selected as the model substrates to evaluate the catalytic efficiency of the chiral BenRu^II^ catalysts developed by Wang.^13b^ The reactions were conducted under the most user-friendly constant current electrolysis (CCE at 2.0 mA) in a mixed solvent system of *tert*-amyl alcohol and water (^*t*^AmOH/H_2_O = 3:1) at 60 °C for 10 h under a nitrogen atmosphere, with KPF_6_ as the electrolyte and NaOPiv as the additive. Orienting experimentation revealed that among the four assessed chiral BenRu^II^ catalysts, the methoxy-substituted catalyst **Ru3** exhibited the highest catalytic activity. The latter delivered the desired product **3** in 45% isolated yield with an enantiomeric ratio (er) of 92:8 (Table 1, entry 3). Motivated by this finding, we turned our attention to the exploration of several reaction mixtures. A systematic evaluation thus revealed that HFIP/H_2_O significantly improved the reaction outcome, affording the desired product **3** in 70% yield with an enhanced enantioselectivity of 93:7 er (Table 1, entries 5–7). Subsequently, we explored the impact of additives, serving as the supporting electrolyte. Compared to NaOPiv, both NaOAc and KOAc presented higher efficacy, with NaOAc significantly improving the enantioselectivity, affording product **3** with an er of 95.5:4.5 (Table 1, entries 8 and 9). A decrease of the reaction temperature to 40 or 50 °C led to significantly reduced chemical yields (Table 1, entries 10 and 11), although the enantioselectivity could be slightly improved at 40 °C. In addition, we tested a reduced applied current. Lowering the current to 1.0 mA did not affect the reaction efficacy, resulting, however, in a slight decrease in the enantioselectivity to 94:6 er. Control experiments mirrored the essential role of both the chiral ruthenium catalyst and electrolysis. In the absence of either **Ru3** or electric current, no or only trace formation of product **3** was detected (entries 12 and 13), which confirmed that the reaction proceeds through a catalyst- and electricity-dependent process.

**Table 1:**
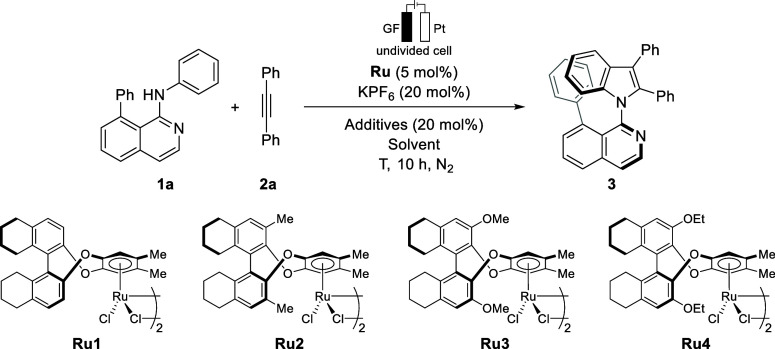
Optimization of the Reaction Conditions^a^

entry	catalyst	additive	solvent	*T*	yield^b^	er
1	**Ru1**	NaOPiv	^*t*^AmOH/H_2_O = 3:1	60 °C	74%	55:45
2	**Ru2**	NaOPiv	^*t*^AmOH/H_2_O = 3:1	60 °C	67%	69:31
3	**Ru3**	NaOPiv	^*t*^AmOH/H_2_O = 3:1	60 °C	45%	92:8
4	**Ru4**	NaOPiv	^*t*^AmOH/H_2_O = 3:1	60 °C	63%	88:12
5	**Ru3**	NaOPiv	MeOH/H_2_O = 3:1	60 °C	89%	88:12
6	**Ru3**	NaOPiv	TFE/H_2_O = 3:1	60 °C	39%	82:18
7	**Ru3**	NaOPiv	HFIP/H_2_O = 3:1	60 °C	70%	93:7
8	**Ru3**	KOAc	HFIP/H_2_O = 3:1	60 °C	98%	93:7
9	**Ru3**	NaOAc	HFIP/H_2_O = 3:1	60 °C	99%	95.5:4.5
10	**Ru3**	NaOAc	HFIP/H_2_O = 3:1	40 °C^c^	17%	97:3
11	**Ru3**	NaOAc	HFIP/H_2_O = 3:1	50 °C^c^	21%	95:5
11^d^	**Ru3**	NaOAc	HFIP/H_2_O = 3:1	60 °C	99%	94:6
12^e^	**Ru3**	NaOAc	HFIP/H_2_O = 3:1	60 °C	trace	-
13	-	NaOAc	HFIP/H_2_O = 3:1	60 °C	N.D	-

a Reaction conditions: undivided cell, **1a** (0.30 mmol), **2a** (0.10 mmol), Ru (5 mol %), KPF_6_ (20 mol %), additive (20 mol %), solvent (4.0 mL), constant current at 2.0 mA, 60 °C, 10 h, graphite felt (GF) anode (10 mm × 15 mm × 6 mm), Pt-plate cathode (10 mm × 15 mm × 0.25 mm), N_2_.

b Isolated yield.

c 36 h.

d Constant current at 1.0 mA.

e Without current. ^*t*^AmOH = *tert*-amyl alcohol, TFE = 2,2,2-trifluoroethanol, and HFIP = 1,1,1,3,3,3-hexafluor-2-propanol.

With the optimal reaction conditions in hand, we explored suitable anilines **1**, along with alkyne **2a**, for the developed ruthenium­(II)-catalyzed C–H activation to access atropostable indoles (Scheme 2). Thus, anilines bearing mono- or disubstituted functional groups with different electronic effects at various positions provided the desired indoles with good-to-excellent chemical yields (up to 93% yield) and excellent levels of enantioselectivity (up to 97:3 er) (**4**–**12**). Electron-donating substituents, such as methyl (**4** and **12**) and methoxy (**9**) moieties, required a slightly higher reaction temperature (70 °C). Halogen and electron-withdrawing substituents were well-tolerated by the electrocatalysis. Additionally, naphthylamine was revealed to be a suitable substrate, delivering the product **13** in 68% yield and a 95.5:4.5 er. Next, we assessed the effects of substituents at the 8-position of the isoquinoline.

**Scheme 2:**
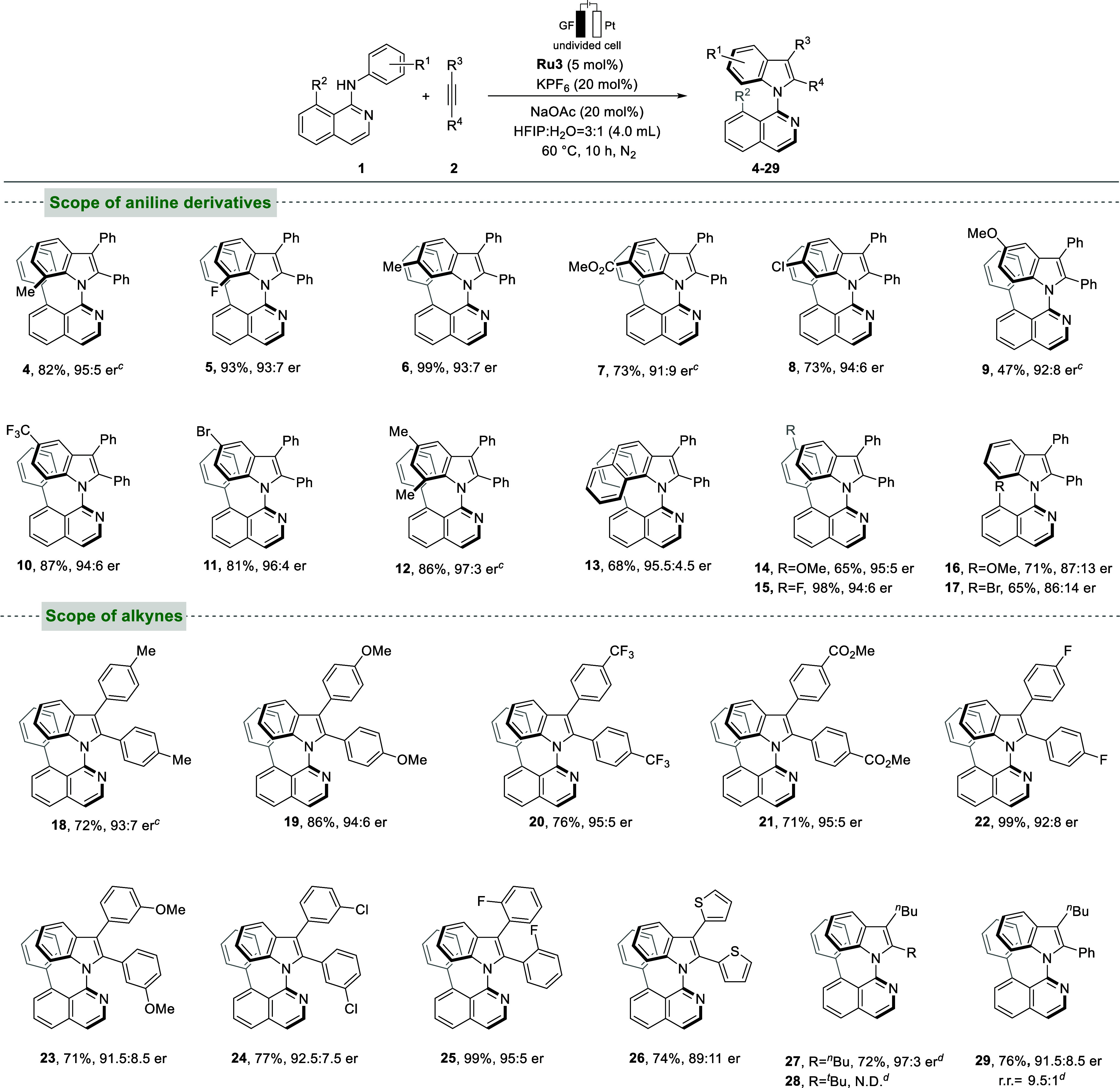
Scope of Atroposelective Indole Synthesis^a^ ^,^ ^b^

a Reaction conditions: undivided cell, **1** (0.30 mmol), **2** (0.10 mmol), **Ru3** (5 mol %), KPF_6_ (20 mol %), NaOAc (20 mol %), HFIP/H_2_O = 3:1 (4.0 mL), constant current at 2.0 mA, 60 °C, 10 h, graphite felt (GF) anode (10 mm × 15 mm × 6 mm), Pt-plate cathode (10 mm × 15 mm × 0.25 mm), N_2_.
b Isolated yield.
c Reaction at 70 °C.
d Reaction with 10 mol % **Ru3**.

Pleasingly, *para*-methoxy- or fluoro-decorated arenes displayed comparable reactivity and enantioselectivity (**14** and **15**). However, the introduction of MeO or Br substituents at the 8-position of the isoquinoline, when compared to substituted phenyls, led to a significant decrease in enantioselectivity (**16** and **17**). This highlights the crucial role of this phenyl substituent in restricting the axial rotation and therefore stabilizing the axial chirality. Thereafter, we directed our attention to robustness with respect to substituted alkynes. Here, a wide range of alkynes **2** bearing substituents at the *para*-, *meta*-, and *ortho*-positions of the arene reacted successfully with **1a** to yield the desired product with excellent enantiomer selectivity in good to high yields (**18**–**25**). Tolane with electron-withdrawing groups, such as trifluoromethyl, ester, or fluoro (**20**–**22**), exhibited better reactivity to give an excellent yield (up to 99%) and enantioselectivity (up to 95:5 er). Extension of the alkyne scope to heteroarene groups, such as thiophene, was also successful, affording product **26** in 74% yield with 89:11 er, without compromising the high chemoselectivity. 5-Decyne proved likewise compatible with our enantioselective electrocatalysis to deliver the product **27** with an outstanding er value of 97:3. The use of an unsymmetrical dialkyl-substituted alkyne, 2,2-dimethyl-3-octyne, under otherwise identical reaction conditions did not furnish the desired product **28**. However, the alkyl-aryl alkyne delivered the axially chiral indole **29** with good yield, high enantioselectivity, and notable regioselectivity. It is worth noting that when we tried to introduce these alkyl-substituted alkynes into our system, they showed much lower activity compared to diphenylacetylene; thus, for products **27**–**29**, higher catalyst loading (10 mol %) was used.

Recently, Mei and Ackermann successfully developed electrochemical C–H [3 + 2] spiroannulations for the synthesis of chiral spiro-pyrazolones employing chiral Cp^*x*^Rh­(III) catalysts.^20^ In view of the much lower cost of ruthenium compared with rhodium, applying ruthenium­(II) catalysts to this reaction constitutes a highly appealing and yet unexplored avenue of research. Next, we extended our electrochemical ruthenium-catalyzed asymmetric C–H activation strategy to enantioselective spiroannulations. The reaction conditions were optimized based on the system described above for atroposelective indole synthesis (see Scheme 3a and Supporting Information). During the initial experiments, **Ru1** exhibited the highest efficiency. Best results were obtained with AgSbF_6_ and NaOAc as additives and ^*n*^Bu_4_NOAc as the supporting electrolyte in a 3:1 mixture of TFE/MeOH at 40 °C for 18 h. Notably, incorporation of chiral carboxylic acid (CCA) **L1** as an external ligand enabled the formation of model product **31** with a 71% isolated yield and 96:4 er, highlighting its essential role in achieving high enantioselectivity and stereochemical control. The chiral match or mismatch effect was also assessed in the presence of acid **L2**, which presents the opposite configuration of **L1**, delivering the expected product in a 59% isolated yield and 94:6 er (Scheme 3a, entry 2). Other CCAs, such as **L3** and **L4**, or a different solvent system composed of TEA and H_2_O, all led to a lower yield and er (Scheme 3a, entries 3–5). When ^*n*^Bu_4_NPF_6_ was used in lieu of ^*n*^Bu_4_NOAc as the supporting electrolyte, the chemical yield dropped by 10% (Scheme 3a, entry 6). Further investigation found that the absence of **L1** would decrease the chemical and optical yield. However, without the AgSbF_6_ and **Ru1**, it would lead to failure of the reaction.

**Scheme 3:**
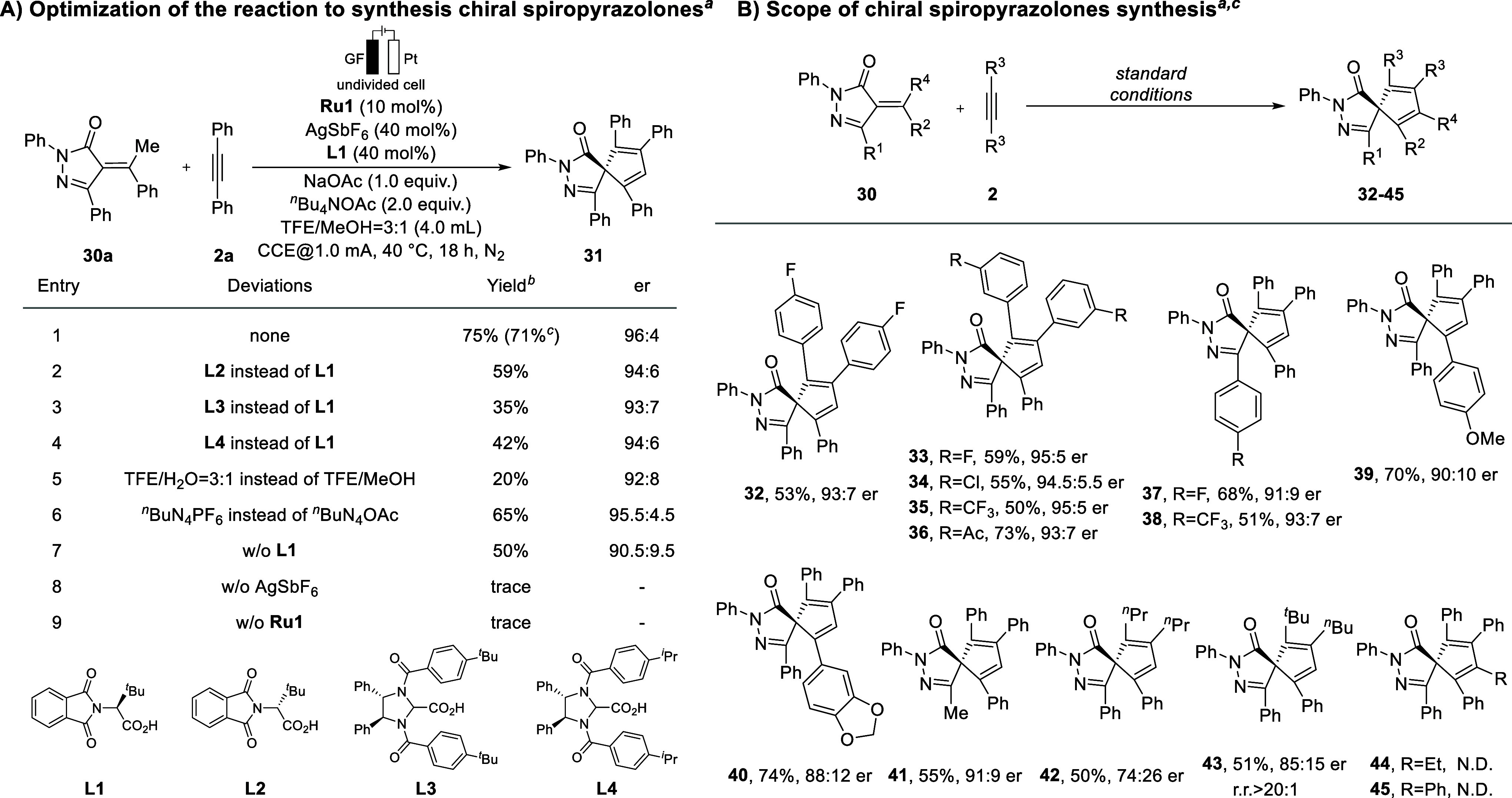
Optimization and Scope of Chiral Spiropyrazolones Synthesis

a Reaction conditions: undivided cell, 30a (0.30 mmol), **2a** (0.10 mmol), **Ru1** (10 mol %), AgSbF_6_ (40 mol %), **L1** (40 mol %), NaOAc (0.10 mmol), ^*n*^Bu_4_NOAc (0.20 mmol), TFE/MeOH = 3:1 (4.0 mL), constant current at 1.0 mA, 40 °C, 18 h, graphite felt (GF) anode (10 mm × 15 mm × 6 mm), Pt-plate cathode (10 mm × 15 mm × 0.25 mm), N_2_.
b Yield was monitored by ^1^H NMR with 1,3,5,-trimethoxybenzene as an internal standard.
c Isolated yield.

With the optimal reaction conditions being established, we then examined the general applicability of the ruthenium­(II)-catalyzed electrochemical synthesis of chiral spiropyrazolones. Symmetrical diarylacetylenes with *meta*- and *para*-substitution afforded the desired products in good to excellent yields (50–73%) and 93:7–95:5 er (**32**–**36**). Substrates with a *para*-fluorophenyl or *para*-trifluoromethylphenyl substituent at the *R* ^1^ position were well-tolerated, furnishing the desired products **37** and **38** in good yield and enantioselectivity. Introduction of a *para*-methoxy substituent on the *R* ^2^ aryl group of *α*-arylidenepyrazolones was well-tolerated in the system and afforded the desired product **39** with 70% yield and 90:10 er. Acetal-protected product **40** was formed in 74% yield with 88:12 er. Replacing the phenyl moiety at the *R* ^1^ position with a methyl group delivered product **41** in 55% yield with a 91:9 er. Furthermore, both symmetric and unsymmetric aliphatic alkynes were investigated. The reaction with oct-4-yne delivered the envisioned product **42** in 50% yield and 74:26 er, whereas 2,2-dimethyl-3-octyne gave product **43** in 51% yield, 85:15 er, and excellent regioselectivity (over 20:1). However, substituting the methyl group with an *n*-propyl or a benzyl group was not well-tolerated.

Given the high enantioselectivity of ruthena-electrocatalysis, we next turned our attention to unraveling its modus operandi. We first did the preliminary isotope labeling experiments to gain some mechanistic insights. When a mixture of CD_3_OD/D_2_O was used as the medium under otherwise identical reaction conditions, 20% deuteration was observed in the *ortho*-position (Scheme 4a). This finding was indicative of fast and facile C–H cleavage. A kinetic isotope effect (KIE) study revealed a minor value of 2.3 by parallel reactions at low conversion (Scheme 4b). The C–H activation intermediate **46** was characterized by high-resolution mass spectrometry (HRMS) when the aniline substrate **1a** was reacted with an equal stoichiometric amount of the ruthenium­(II) catalyst (Scheme 4c). Furthermore, cyclic voltammetry (CV) was performed to investigate the redox properties of different substrates and catalysts under electrochemical conditions for atroposelective indole synthesis. The substrate aniline **1a** exhibited oxidation peaks at Ep/2 = 1.55 V and 2.05 V *vs.* SCE. However, when **1a** was thoroughly mixed with the ruthenium­(II) precatalyst, the fading of the oxidation peaks was supportive of a cyclometalation event (Scheme 4d). Headspace gas chromatography was used to probe the formation of molecular hydrogen (Supporting Information, Figure S4).

**Scheme 4:**
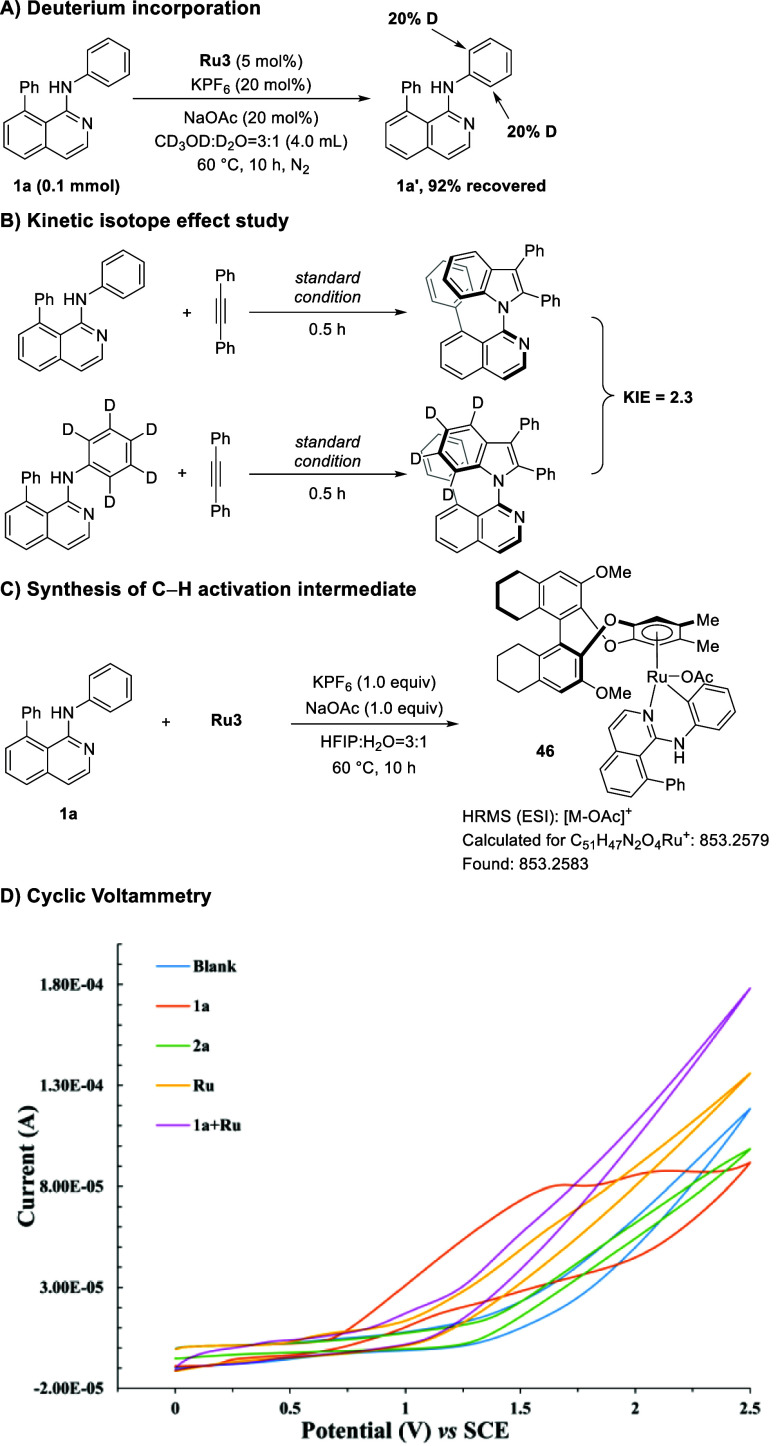
Mechanism Studies

### DFT-Based Mechanistic Studies

Intrigued by the catalyst mode of action, the origin of the enantioselectivity was explored by means of DFT calculations at the PBE0-D3­(BJ)/def2-TZVP,SDD­(Ru)+SMD­(HFIP)//PBE0-D3­(BJ)/def2-SVP,def2-TZVP­(Ru),SDD­(Ru) level of theory (Figure 1) (for more details, please see Supporting Information). Migratory insertion of an alkyne was shown to be the enantio-determining step (Figure 1). The latter, for the experimentally observed major enantiomer, proceeds via **^ent1^TS­(1–2)** with an energy of 9.3 kcal mol^–1^. For enantiomer 2, **^ent2^Int-2** was shown to be more exergonic than **^ent1^Int-1** by 3.1 kcal mol^–1^, undergoing migratory insertion through **^ent2^TS­(1–2)** with an energy barrier of 14.0 kcal mol^–1^. Although noncovalent interaction (NCI) analysis indicates the presence of attractive π–π interactions in both transition states, these are more pronounced in **^e^ ^nt1^TS­(1–2)** than in ^**ent2**^ **TS­(1**–**2)** (Figure 2). Additionally, the latter was shown to be destabilized by steric hindrance (Figure 2). Furthermore, the influence of the phenyl substituent at the 8-position of the isoquinolines on the axial rotation was further explored. Here, the rotational barrier of enantiomer 1 was calculated in the presence of phenyl, isopropyl substituents, and hydrogen. In the case of phenyl, the rotational barrier was shown to be 30.8 kcal mol^–1^ (*t*_1/2_ at 60 °C = 92.8 days), whereas for hydrogen, it was calculated to be 28.1 kcal mol^–1^ (*t*_1/2_ at 60 °C = 1.8 days). For isopropyl, the rotational barrier was shown to be higher than that for the phenyl substituent, with a rotation barrier of 33.1 kcal mol^–1^ (*t*_1/2_ at 60 °C = 8.2 years). This indicates that both phenyl and isopropyl significantly restrict the axial rotation. Additionally, the phenyl substituent provides stability through π–π interactions.

**Figure 1:**
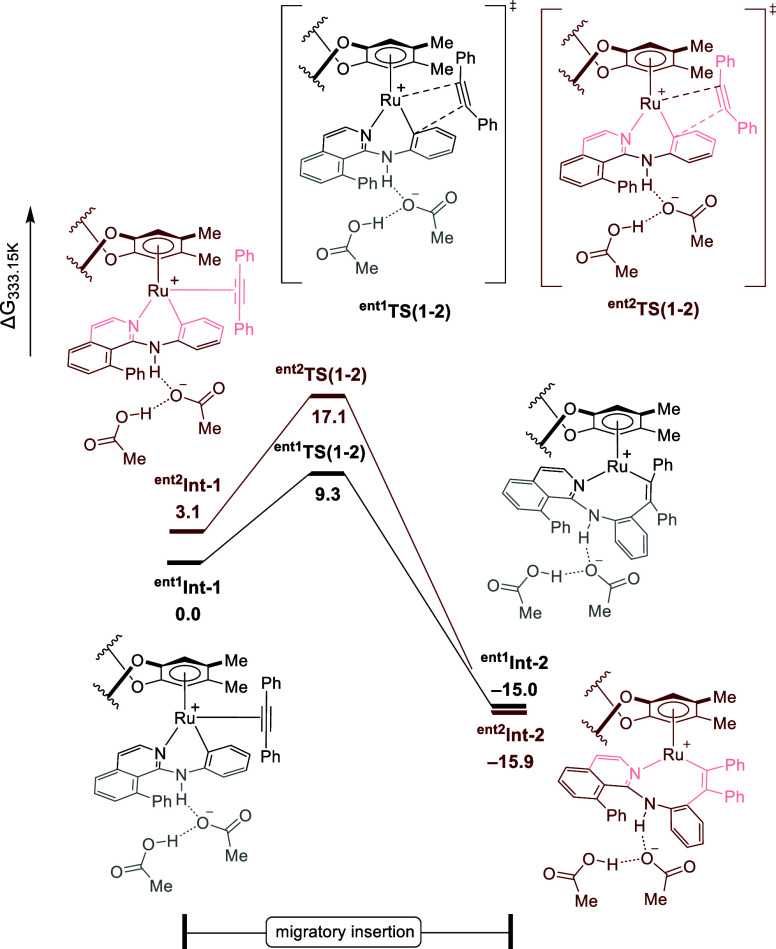
Computed relative Gibbs free energies (Δ*G*_333.15_) in kcal mol^–1^ for the enantio-determining migratory insertion elementary step for both enantiomers pathways at the PBE0-D3­(BJ)/def2-TZVP,SDD­(Ru)+SMD­(HFIP)//PBE0-D3­(BJ)/def2-SVP,def2-TZVP­(Ru),SDD­(Ru) level of theory.

**Figure 2:**
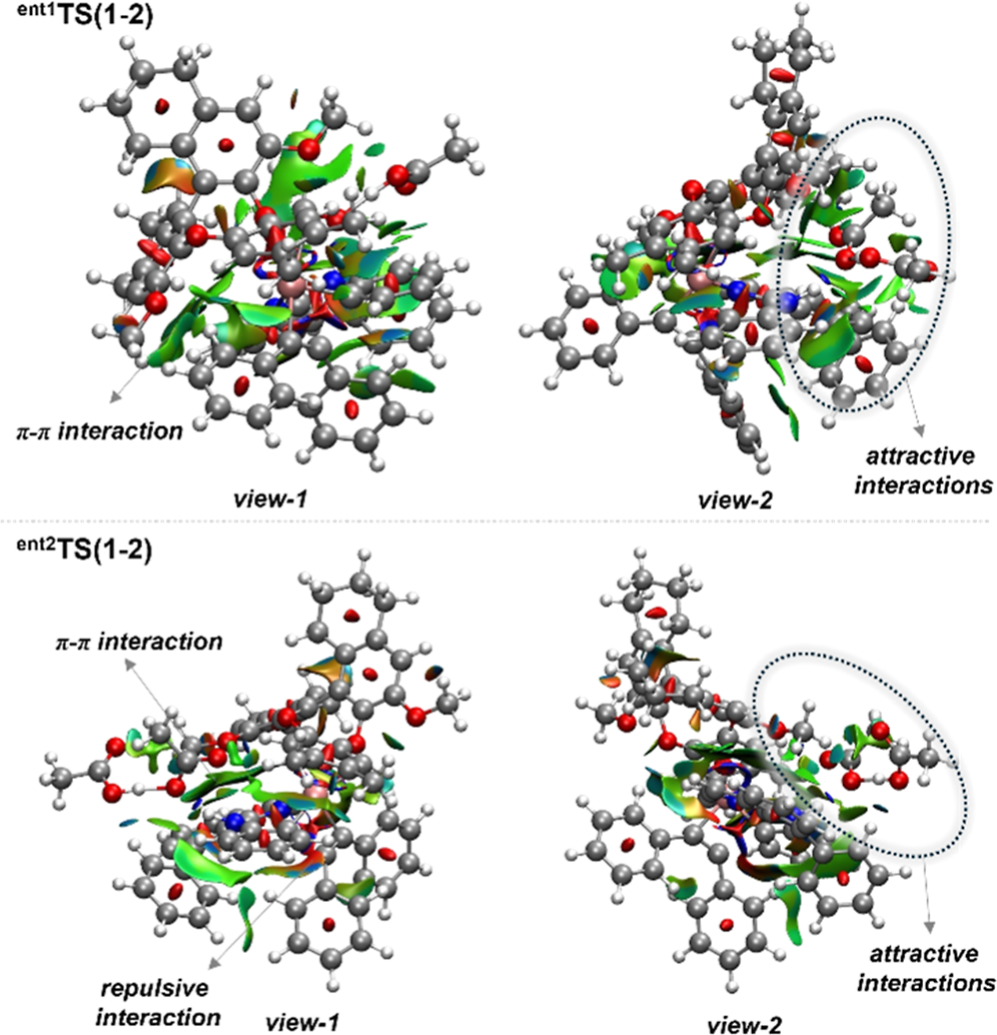
Noncovalent interaction (NCI) analysis of **^ent1^TS­(1–2)** and **^ent2^TS­(1–2)**.

Based on our experiment- and DFT-based mechanistic studies and literature precedents,^21–23^ a plausible reaction mechanism of ruthenium­(II)-catalyzed electrolytic synthesis of atroposelective indole is proposed in Scheme 5. First, the active BenRu^II^ catalyst, which is generated by a ligand exchange of the precatalyst with the additive NaOAc, coordinates with aniline **1a** and then activates the C–H bond to give a six-membered ruthenacycle **A**. Second, the acetate ligand is subsequently displaced by alkyne substrate **2a** via π-coordination, affording intermediate **B**. Coordination of alkyne **2a**, hence, promotes alkyne migratory insertion into the Ru–C bond, inducing dissociation of the metal center from the nitrogen and subsequent coordination to the amino group of the aniline moiety, thereby forming a six-membered ruthenacycle in intermediate **C**. In addition, in agreement with previous studies by the Wang group,^8^ the π–π interaction between the isoquinoline directing rings of the substrate and the chiral ligand might also help to constrain the rotation of the substrate, resulting in a preferred orientation. Finally, reductive elimination from intermediate **C** delivers indole product **3** along with a ruthenium(0) species, which is subsequently reoxidized at the anode to regenerate the catalytically active ruthenium­(II) complex. A plausible reaction mechanism for the synthesis of spiropyrazolones based on literature reports and previous mechanistic studies is proposed in the Supporting Information (page S58).^13,20a,24^

**Scheme 5:**
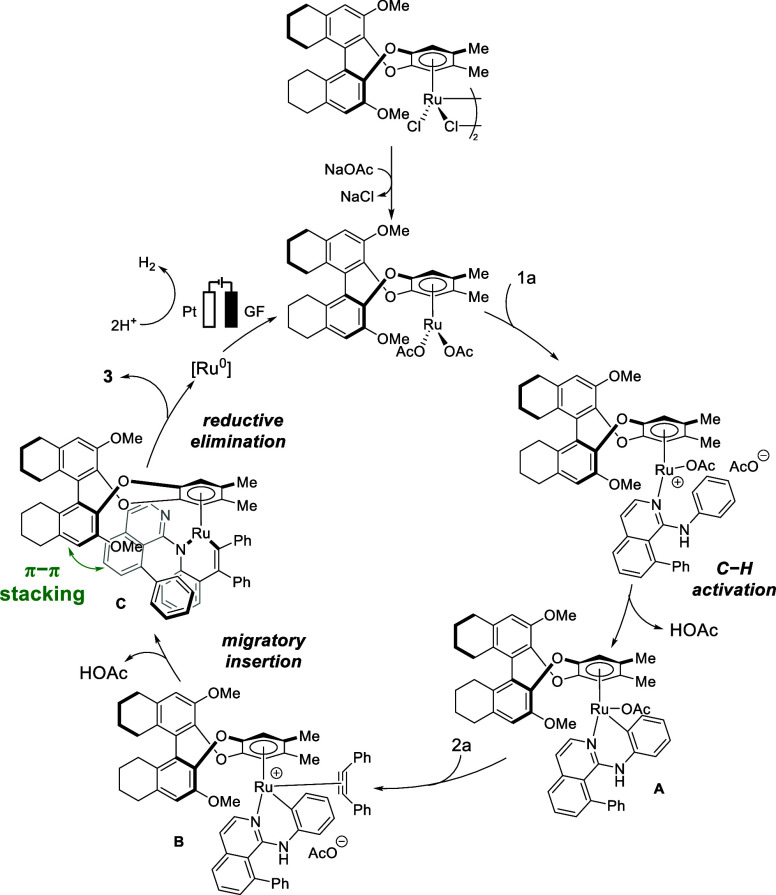
Proposed Mechanism for the Synthesis of Atroposelective Indole 3

## Conclusions

We have demonstrated that electrochemical ruthenium­(II) catalysis can enable enantioselective C–H activations. With electricity-electrons and protons-as a clean oxidant, this strategy efficiently converts readily available anilines and *α*-arylidenepyrazolone derivatives into biorelevant atropostable indoles and chiral spiropyrazolones with high levels of chemical yields and enantioselectivities, with molecular hydrogen as the sole stoichiometric byproduct through cathodic proton reduction. This finding mirrors the outstanding potential of merging sustainable ruthenium catalysis with electrochemical syntheses with major prospects for applied areas.

## Supplementary Material



## Notes

Experimental procedures, optimization studies, mechanistic experiments, characterization of the new compounds, and NMR spectra (PDF)
