# Supplementary material for: Electrochemical Enantioselective Ruthenium(II)-Catalyzed C–H Activations to Atropostable Indoles and Chiral Spiropyrazolones
Source: J Am Chem Soc. 2026 May 6;148(19):19773–80. doi: 10.1021/jacs.6c01678 (PMC13195655; doi:10.1021/jacs.6c01678)
Supplement: Supplementary file 1 [file ja6c01678_si_001.pdf]

Supporting Information

Electrochemical Enantioselective Ruthenium(II)-Catalyzed C–H Activations to Atropostable Indoles and Chiral Spiropyrazolones

Siyu Liu[‡], Jiawei Xu[‡], Parveen Rawal, João C. A. Oliveira, Lutz Ackermann[*]

Wöhler Research Institute for Sustainable Chemistry (WISCh), Georg-August-Universität, Tammannstraße 2, 37077 Göttingen, Germany

[*]Corresponding author: lutz.ackermann@chemie.uni-goettingen.de

[‡]These authors contributed equally: Siyu Liu, Jiawei Xu

Table of Contents

1. General Information 3

2. Synthesis and Characterization of Substrates 4

Synthesis of *N*-isoquinolylanilines 1 4

Synthesis of α-Arylidene Pyrazolones 30 9

3. Optimization of the Reaction Conditions for Chiral Spiropyrazolones Synthesis 11

4. General Procedure and Products Characterization 12

General Procedure A for the synthesis of atroposelective indole products 3-29 12

General Procedure B for the synthesis of chiral spiropyrazolones 31-45 12

Reaction Setup 13

Characterization data of the Products 14

5.Mechanistic studies 53

Preliminary isotope labelling experiments 53

Kinetic isotope effect (KIE) experiments by parallel reactions 54

Capture of the C−H activation intermediate 54

Cyclic Voltammetry studies 55

Cathodic Process Study 56

Proposed mechanism for the synthesis of chiral spiropyrazolones 57

6. Computational studies 58

Effect of isoquinoline's substituents at the 8th position on the rotational barrier 59

7. Cartesian coordinates of the optimized structure 61

8. References 97

9. NMR Spectra 100

1. General Information

If not otherwise marked, the reagents were obtained from commercial sources including Sigma-Aldrich, Alfa Aesar, and TCI Deutschland and used directly without further purification. Heating source: all the reactions that require heating were carried out in an oil bath or an aluminum heating block. TLC: Macherey-Nagel, TLC plates Alugram®Sil G/UV254. Detection under UV light at 254 nm. Chromatography: Separations were carried out on Merck Silica 60 (0.040–0.063 mm, 70–230 mesh ASTM). High resolution mass spectrometry (HRMS): APEX IV 7T FTICR, Bruker Daltonic. HPLC chromatograms were recorded on an Agilent 1290 Infinity using CHIRALPAK® IA-3, IB-3, IC-3, ID-3, IE-3, IF-3 and AD-3 columns (3.0 µm particle size; Ø: 4.6 mm and 250 mm length). Optical rotations were measured with Perkin Elmer 343 polarimeter at the stated temperature under a Na/Hg lamp, λ = 589 nm (c in g/100 mL), and reported as follows: $[\alpha]^{D}T$ = (c in g/100 mL, in $CDCl_3$). ^{1}H, ^{13}C, and ^{19}F-NMR spectra were recorded at 300 (^{1}H), 400 (^{1}H), 500 (^{1}H) , 600 (^{1}H), 75 [^{13}C, APT (Attached Proton Test)], 101 [^{13}C, APT (Attached Proton Test)], 126 [^{13}C, APT (Attached Proton Test)], 151 [^{13}C, APT (Attached Proton Test)] and 282 (^{19}F), 377 (^{19}F), 471 (^{19}F), 565 (^{19}F) MHz respectively, on Varian Bruker Avance III 400, Bruker Avance III HD 600. If not otherwise specified, chemical shifts (δ) are given in ppm. All chiral catalysts used in this work are synthesized according to Wang's work[1].

2. Synthesis and Characterization of Substrates

Synthesis of *N*-isoquinolylanilines 1[2]

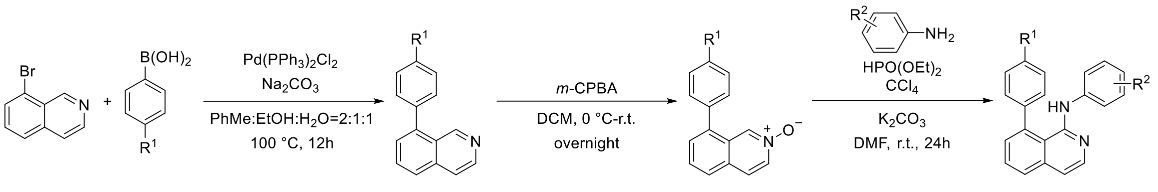

1a-1o

Step 1: To a flask charged 8-bromoisoquinoline (4.0 mmol, 1.0 equiv.), corresponding phenylboronic acid derivatives (4.8 mmol, 1.2 equiv.), $Pd(PPh_3)Cl_2$ (0.2 mmol, 5.0 %mol) and sodium carbonate (8.0 mmol, 2.0 equiv.), added 8mL toluene, 4 mL EtOH and 4 mL H_2O. Then the reaction mixture was stirred at 100 °C under N_2 for 12 h. After reaction completion, the reaction mixture was passed through a short pad of celite, and the filtrate was diluted with H_2O. The separated aqueous layer was extracted with EtOAc. The combined organic layers were washed with brine, dried over $MgSO_4$ and concentrated in vacuo. The residue as yellow oil was used for the next step without further purification.

Step 2: To a solution of the corresponding isoquinoline products (3.5 mmol, 1.0 equiv.) in CH_2Cl_2 (20 ml), added *m*-chloroperbenzoic acid (7.0 mmol, 2.0 equiv.) in small potion at 0 °C. The reaction mixture was then allowed to warm to room temperature and stirred overnight. After concentration, the residue was purified by flash chromatography on silica gel (EtOAc:MeOH = 10:1) to give the isoquinoline *N*-oxides.

Step 3: To a stirred mixture of isoquinoline *N*-oxides (2.0 mmol, 1.0 equiv.), K_2CO_3 (5.0 mmol, 2.5 equiv.) in DMF (10 mL) added diethyl phosphonate (5.0 mmol, 2.5 equiv.), CCl_4 (5.0 mL) and amines (3.0 mmol, 1.5 equiv.). The reaction mixture was allowed to stir at room temperature for 24 h. The reaction was quenched with water and extracted with EtOAc. The separated aqueous layer was extracted with EtOAc. The combined organic layers were washed with brine, dried over $MgSO_4$ and concentrated in vacuo. The residue was subjected to flash chromatography on silica gel (*n*-Hexane:EtOAc = 30:1) to give the *N*-isoquinolylanilines **1a-1m**.

Note: The *N*-isoquinolylanilines substrates **1n** and **1o** were synthesized according to procedures above with 8-methoxyisoquinoline and 8-bromoisoquinoline starting from Step 2. The characteristic data for **1a**, **1m** and **1n** are consistent with previous literature[3].

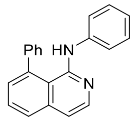

1a

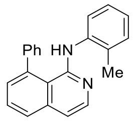

1b

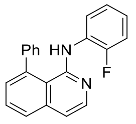

1c

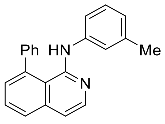

1d

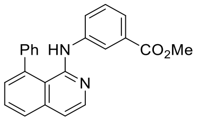

1e

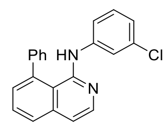

1f

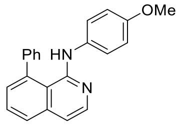

1g

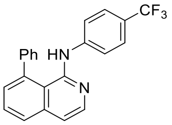

1h

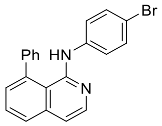

1i

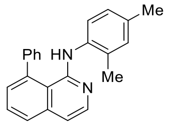

1j

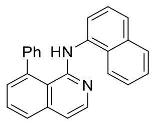

1k

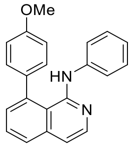

1l

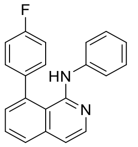

1m

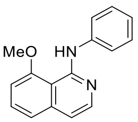

1n

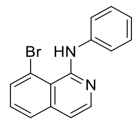

1o

Characterization data for *N*-isoquinolylanilines not described in the literature:

8-phenyl-*N*-(*o*-tolyl)isoquinolin-1-amine (1b). Prepared according to general procedure for *N*-isoquinolylaniline synthesis, isolation by column chromatography (*n*-hexane/EtOAc) yielded **1b** as a yellow solid. **^{1}H NMR** (400 MHz, $CDCl_3$) δ 8.05 (d, *J* = 5.6 Hz, 1H), 7.79 (dd, *J* = 8.1, 1.3 Hz, 1H), 7.74 (dd, *J* = 8.2, 1.4 Hz, 1H), 7.59 (dd, *J* = 8.1, 7.1 Hz, 1H), 7.55 – 7.40 (m, 6H), 7.29 (dd, *J* = 7.1, 1.4 Hz, 1H), 7.17 – 7.11 (m, 2H), 7.03 (dd, *J* = 7.1, 1.4 Hz, 1H), 6.92 (td, *J* = 7.4, 1.3 Hz, 1H), 6.36 (s, 1H), 1.63 (s, 3H). **^{13}C NMR** (101 MHz, $CDCl_3$) δ 153.1 (C_q), 142.0 (C_q), 141.3 (CH), 139.2 (C_q), 138.6 (C_q), 138.4 (C_q), 130.4 (CH), 130.2 (CH), 129.7 (C_q), 129.6 (CH), 129.0 (CH), 128.7 (CH), 128.5 (CH), 127.4 (CH), 126.3 (CH), 123.5 (CH), 122.6 (CH), 116.5 (C_q), 113.2 (CH), 17.7 (CH_3). **HRMS** (ESI): m/z $[M+Na]^+$ calculated for $C_{22}H_{18}N_2Na^+$ 333.1362, found 333.1359.

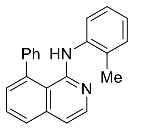

***N*-(2-fluorophenyl)-8-phenylisoquinolin-1-amine (1c).** Prepared according to general procedure for *N*-isoquinolylaniline synthesis, isolation by column chromatography (*n*-hexane/EtOAc) yielded **1c** as a yellow solid. **^{1}H NMR** (300 MHz, $CDCl_3$) δ 8.62 – 8.53 (m, 1H), 8.14 (d, *J* = 5.6 Hz, 1H), 7.75 (dd, *J* = 8.1, 1.4 Hz, 1H), 7.61 (dd, *J* = 8.1, 7.1 Hz, 1H), 7.55 – 7.47 (m, 5H), 7.33 (dd, *J* = 7.1, 1.4 Hz, 1H), 7.21 (d, *J* = 5.6 Hz, 1H), 7.11 – 6.99 (m, 2H), 6.93 – 6.80 (m, 2H). **^{13}C NMR** (75 MHz, $CDCl_3$) δ 152.7 (d, *J* = 243.8 Hz, C_q), 152.0 (C_q), 141.3 (C_q), 140.7 (CH), 139.0 (C_q), 138.6 (C_q), 130.3 (CH), 129.4 (CH), 129.1 (CH), 128.8 (CH),

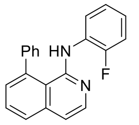

128.8 (d, J = 9.7 Hz, C_q), 128.6 (CH), 127.4 (CH), 124.0 (d, J = 3.6 Hz, CH), 121.8 (d, J = 7.4 Hz, CH), 121.1 (d, J = 1.5 Hz CH), 116.9 (C_q), 114.5 (d, J = 19.3 Hz, CH), 114.0 (CH). **^{19}F NMR** (377 MHz, $CDCl_3$) δ -131.28. **HRMS** (ESI): m/z $[M+Na]^+$ calculated for $C_{21}H_{15}FN_2Na^+$ 337.1111, found 337.1109.

8-phenyl-*N*-(*m*-tolyl)isoquinolin-1-amine (1d). Prepared according to general procedure for *N*-isoquinolylaniline

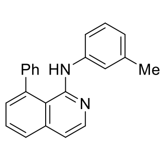

synthesis, isolation by column chromatography (*n*-hexane/EtOAc) yielded **1d** as a yellow oil. **^{1}H NMR** (300 MHz, $CDCl_3$) δ 8.12 (d, J = 5.6 Hz, 1H), 7.73 (dd, J = 8.2, 1.4 Hz, 1H), 7.63 – 7.49 (m, 6H), 7.31 (dd, J = 7.1, 1.4 Hz, 1H), 7.14 (d, J = 5.7 Hz, 1H), 7.10 – 7.02 (m, 2H), 6.91 (dt, J = 7.2, 1.5 Hz, 1H), 6.81 (s, 1H), 6.77 – 6.70 (m, 1H), 2.28 (s, 3H). **^{13}C NMR** (75 MHz, $CDCl_3$) δ 152.4 (C_q), 141.9 (C_q), 141.0 (CH), 140.1 (C_q), 139.0 (C_q), 138.5 (C_q), 138.3 (C_q), 129.6 (CH), 129.1 (CH), 128.6 (CH), 128.6 (CH), 127.5 (CH), 122.9 (CH), 119.8 (CH), 116.7 (C_q), 116.3 (CH), 113.1 (CH), 21.8 (CH_3). **HRMS** (ESI): m/z $[M+Na]^+$ calculated for $C_{22}H_{18}N_2Na^+$ 333.1362, found 333.1359.

Methyl 3-((8-phenylisoquinolin-1-yl)amino)benzoate (1e). Prepared according to general procedure for *N*-

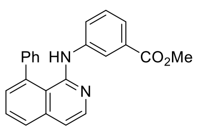

isoquinolylaniline synthesis, isolation by column chromatography (*n*-hexane/EtOAc) yielded **1e** as a yellow solid. **^{1}H NMR** (300 MHz, $CDCl_3$) δ 8.14 (d, J = 5.6 Hz, 1H), 7.76 (dd, J = 8.2, 1.4 Hz, 1H), 7.69 – 7.66 (m, 1H), 7.65 – 7.49 (m, 8H), 7.35 (dd, J = 7.1, 1.4 Hz, 1H), 7.28 – 7.22 (m, 1H), 7.19 (d, J = 5.7 Hz, 1H), 6.91 (s, 1H), 3.90 (s, 3H). **^{13}C NMR** (75 MHz, $CDCl_3$) δ 167.3 (C_q), 152.1 (C_q), 141.8 (C_q), 140.9 (CH), 140.5 (C_q), 139.0 (C_q), 138.2 (C_q), 130.6 (C_q), 129.7 (CH), 129.6 (CH), 129.2 (CH), 128.8 (CH), 127.6 (CH), 123.3 (CH), 122.9 (CH), 120.1 (CH), 116.7 (C_q), 113.8 (CH), 52.2 (CH_3). **HRMS** (ESI): m/z $[M+Na]^+$ calculated for $C_{23}H_{18}N_2NaO_2^+$ 377.1260, found 377.1256.

***N*-(3-chlorophenyl)-8-phenylisoquinolin-1-amine (1f).** Prepared according to general procedure for *N*-

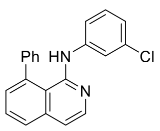

isoquinolylaniline synthesis, isolation by column chromatography (*n*-hexane/EtOAc) yielded **1f** as a red oil. **^{1}H NMR** (300 MHz, $CDCl_3$) δ 8.14 (d, J = 5.6 Hz, 1H), 7.76 (dd, J = 8.2, 1.4 Hz, 1H), 7.64 – 7.48 (m, 7H), 7.34 (dd, J = 7.1, 1.4 Hz, 1H), 7.19 (d, J = 5.6 Hz, 1H), 7.09 – 7.01 (m, 1H), 6.90 – 6.81 (m, 2H), 6.75 – 6.70 (m, 1H). **^{13}C NMR** (75 MHz, $CDCl_3$) δ 151.9 (C_q), 141.8 (C_q), 141.4 (C_q), 140.8 (CH), 139.0 (C_q),

138.1 (C_q), 134.4 (C_q), 129.8 (CH), 129.6 (CH), 129.6 (CH), 129.2 (CH), 128.8 (CH), 128.8 (CH), 127.6 (CH), 121.6 (CH), 118.7 (CH), 116.9 (CH), 116.7 (C_q), 114.0 (CH). **HRMS** (ESI): m/z $[M+Na]^+$ calculated for $C_{21}H_{15}ClN_2Na^+$ 353.0816, found 353.0815.

***N*-(4-methoxyphenyl)-8-phenylisoquinolin-1-amine (1g).** Prepared according to general procedure for *N*-isoquinolylaniline synthesis, isolation by column chromatography (*n*-hexane/EtOAc) yielded **1g** as a yellow solid. **^{1}H NMR** (300 MHz, $CDCl_3$) δ 8.06 (d, J = 5.6 Hz, 1H), 7.72 (dd, J = 8.2, 1.4 Hz, 1H), 7.61 – 7.47 (m, 6H), 7.30 (dd, J = 7.1, 1.4 Hz, 1H), 7.15 – 7.05 (m, 3H), 6.80 – 6.71 (m, 2H), 6.67 (s, 1H), 3.75 (s, 3H). **^{13}C NMR** (75 MHz, $CDCl_3$) δ 155.0 (C_q), 152.7 (C_q), 141.9 (C_q), 141.1 (CH), 139.0 (C_q), 138.3 (C_q), 133.6 (C_q), 129.6 (CH), 129.5 (CH), 129.0 (CH), 128.6 (CH), 127.4 (CH), 121.3 (CH), 116.4 (C_q), 114.1 (CH), 112.6 (CH), 55.6 (CH_3). **HRMS** (ESI): m/z $[M+Na]^+$ calculated for $C_{22}H_{18}N_2NaO^+$ 349.1311, found 349.1310.

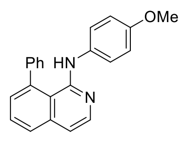

8-phenyl-*N*-(4-(trifluoromethyl)phenyl)isoquinolin-1-amine (1h). Prepared according to general procedure for *N*-isoquinolylaniline synthesis, isolation by column chromatography (*n*-hexane/EtOAc) yielded **1h** as a yellow solid. **^{1}H NMR** (500 MHz, $CDCl_3$) δ 8.16 (d, J = 5.6 Hz, 1H), 7.77 (dd, J = 8.2, 1.4 Hz, 1H), 7.63 (dd, J = 8.1, 7.1 Hz, 1H), 7.61 – 7.56 (m, 3H), 7.54 – 7.50 (m, 2H), 7.41 (d, J = 8.1 Hz, 2H), 7.36 (dd, J = 7.1, 1.4 Hz, 1H), 7.25 (s, 1H), 7.24 – 7.22 (m, 2H), 7.03 (s, 1H). **^{13}C NMR** (126 MHz, $CDCl_3$) δ 151.7 (C_q), 143.2 (C_q), 141.7 (C_q), 140.7 (CH), 139.0 (C_q), 138.1 (C_q), 130.0 (CH), 129.7 (CH), 129.2 (CH), 128.9 (CH), 128.8 (CH), 127.7 (CH), 126.0 (q, $J_{C\text{-}F}$ = 3.8 Hz, CH), 124.6 (q, $J_{C\text{-}F}$ = 271.1 Hz, C_q), 123.1 (q, $J_{C\text{-}F}$ = 32.5 Hz, C_q), 118.1 (CH), 116.8 (C_q), 114.5 (CH). **^{19}F NMR** (471 MHz, $CDCl_3$) δ -61.67. **HRMS** (ESI): m/z $[M+Na]^+$ calculated for $C_{22}H_{15}F_3N_2Na^+$ 387.1080, found 387.1078.

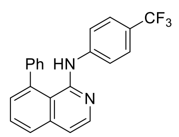

***N*-(4-bromophenyl)-8-phenylisoquinolin-1-amine (1i).** Prepared according to general procedure for *N*-isoquinolylaniline synthesis, isolation by column chromatography (*n*-hexane/EtOAc) yielded **1i** as a yellow solid. **^{1}H NMR** (300 MHz, $CDCl_3$) δ 8.11 (d, J = 5.7 Hz, 1H), 7.75 (dd, J = 8.2, 1.4 Hz, 1H), 7.64 – 7.48 (m, 6H), 7.33 (dd, J = 7.1, 1.4 Hz, 1H), 7.29 – 7.23 (m, 2H), 7.17 (d, J = 5.6 Hz, 1H), 7.10 – 7.03 (m, 2H), 6.83 (s, 1H). **^{13}C NMR** (75 MHz, $CDCl_3$) δ 152.0 (C_q), 141.8 (C_q), 140.8 (CH), 139.3 (C_q), 139.0 (C_q),

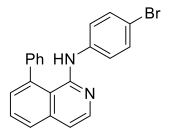

138.1 (C_q), 131.6 (CH), 129.8 (CH), 129.6 (CH), 129.2 (CH), 128.8 (CH), 128.7 (CH), 127.6 (CH), 120.5 (CH), 116.7 (C_q), 113.9 (C_q), 113.7 (CH). **HRMS** (ESI): m/z $[M+Na]^+$ calculated for $C_{21}H_{15}BrN_2Na^+$ 397.0311, found 397.0309.

***N*-(2,4-dimethylphenyl)-8-phenylisoquinolin-1-amine (1j).** Prepared according to general procedure for *N*-isoquinolylaniline synthesis, isolation by column chromatography (*n*-hexane/EtOAc) yielded **1j** as a pink solid.

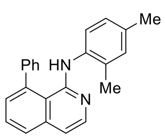

^{1}H NMR (300 MHz, $CDCl_3$) δ 8.03 (d, *J* = 5.6 Hz, 1H), 7.73 (dd, *J* = 8.2, 1.4 Hz, 1H), 7.58 (dd, *J* = 8.2, 7.2 Hz, 1H), 7.56 – 7.39 (m, 6H), 7.28 (dd, *J* = 7.1, 1.4 Hz, 1H), 7.09 (d, *J* = 5.6 Hz, 1H), 6.96 (dd, *J* = 8.1, 2.1 Hz, 1H), 6.88 (d, *J* = 2.2 Hz, 1H), 6.27 (s, 1H), 2.25 (s, 3H), 1.68 (s, 3H). **^{13}C NMR** (75 MHz, $CDCl_3$) δ 153.5 (C_q), 142.1 (C_q), 141.5 (CH), 139.2 (C_q), 138.6 (C_q), 135.7 (C_q), 133.4 (C_q), 131.1 (CH), 130.5 (C_q), 129.9 (CH), 129.6 (CH), 128.9 (CH), 128.6 (CH), 128.4 (CH), 127.3 (CH), 126.9 (CH), 123.6 (CH), 116.3 (C_q), 112.7 (CH), 21.0 (CH_3), 17.8 (CH_3). **HRMS** (ESI): m/z $[M+Na]^+$ calculated for $C_{23}H_{20}N_2Na^+$ 347.1519, found 347.1516.

***N*-(naphthalen-1-yl)-8-phenylisoquinolin-1-amine (1q).** Prepared according to general procedure for *N*-isoquinolylaniline synthesis, isolation by column chromatography (*n*-hexane/EtOAc) yielded **1q** as a yellow solid.

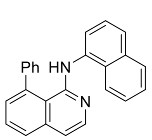

^{1}H NMR (300 MHz, $CDCl_3$) δ 8.13 (d, *J* = 7.5 Hz, 1H), 8.08 (d, *J* = 5.6 Hz, 1H), 7.82 – 7.73 (m, 2H), 7.68 – 7.58 (m, 3H), 7.57 – 7.32 (m, 7H), 7.25 – 7.19 (m, 1H), 7.18 (d, *J* = 5.8 Hz, 1H), 7.11 (s, 1H), 6.89 (d, *J* = 8.5 Hz, 1H). **^{13}C NMR** (75 MHz, $CDCl_3$) δ 153.5 (C_q), 141.9 (C_q), 141.2 (CH), 139.2 (C_q), 138.5 (C_q), 135.6 (C_q), 134.3 (C_q), 130.2 (CH), 129.7 (CH), 129.2 (CH), 128.8 (CH), 128.7 (CH), 128.5 (CH), 127.5 (CH), 126.0 (CH), 125.6 (CH), 125.2 (CH), 123.7 (CH), 121.3 (CH), 118.4 (CH), 116.8 (C_q), 113.5 (CH).**HRMS** (ESI): m/z $[M+Na]^+$ calculated for $C_{25}H_{18}N_2Na^+$ 369.1362, found 369.1360.

8-(4-methoxyphenyl)-*N*-phenylisoquinolin-1-amine (1l). Prepared according to general procedure for *N*-isoquinolylaniline synthesis, isolation by column chromatography (*n*-hexane/EtOAc) yielded **1l** as a yellow solid.

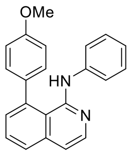

^{1}H NMR (300 MHz, $CDCl_3$) δ 8.14 (d, *J* = 5.6 Hz, 1H), 7.75 (dd, *J* = 8.2, 1.4 Hz, 1H), 7.61 (dd, *J* = 8.2, 7.1 Hz, 1H), 7.51 – 7.45 (m, 2H), 7.33 (dd, *J* = 7.1, 1.4 Hz, 1H), 7.30 – 7.21 (m, 4H), 7.17 (d, *J* = 5.7 Hz, 1H), 7.14 – 7.09 (m, 2H), 7.08 (s, 1H), 6.99 – 6.92 (m, 1H), 3.96 (s, 3H). **^{13}C NMR** (75 MHz, $CDCl_3$) δ

160.0 (C_q), 152.5 (C_q), 140.9 (CH), 140.3 (C_q), 139.0 (C_q), 138.0 (C_q), 133.8 (C_q), 130.8 (CH), 130.1 (CH), 128.8 (CH), 128.7 (CH), 127.3 (CH), 122.0 (CH), 119.3 (CH), 116.9 (C_q), 114.5 (CH), 113.2 (CH), 55.7 (CH_3). **HRMS** (ESI): m/z $[M+Na]^+$ calculated for C22H18N2NaO+ 349.1311, found 349.1310.

8-(4-fluorophenyl)-*N*-phenylisoquinolin-1-amine (1m). Prepared according to general procedure for *N*-isoquinolylaniline synthesis, isolation by column chromatography (*n*-hexane/EtOAc) yielded **1m** as a yellow solid.

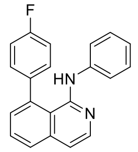

^{1}H NMR (300 MHz, $CDCl_3$) δ 8.10 (d, J = 5.6 Hz, 1H), 7.72 (dd, J = 8.2, 1.4 Hz, 1H), 7.57 (dd, J = 8.2, 7.1 Hz, 1H), 7.52 – 7.45 (m, 2H), 7.29 – 7.24 (m, 2H), 7.23 – 7.16 (m, 5H), 7.13 (d, J = 5.6 Hz, 1H), 6.95 – 6.88 (m, 1H), 6.73 (s, 1H). **^{13}C NMR** (75 MHz, $CDCl_3$) δ 162.9 (d, J_{C-F} = 249.3 Hz, C_q), 152.2 (C_q), 141.1 (CH), 140.1 (C_q), 139.1 (C_q), 137.8 (d, J_{C-F} = 3.6 Hz, C_q), 137.1 (C_q), 131.4 (d, J_{C-F} = 8.0 Hz, CH), 130.0 (CH), 128.9 (CH), 128.7 (CH), 127.7 (CH), 122.2 (CH), 119.2 (CH), 116.6 (C_q), 116.1 (d, J_{C-F} = 21.5 Hz, CH), 113.4 (CH). **^{19}F NMR** (282 MHz, $CDCl_3$) δ -112.7. **HRMS** (ESI): m/z $[M+Na]^+$ calculated for $C_{21}H_{15}FN_2Na^+$ 337.1111, found 337.1110.

Synthesis of α-Arylidene Pyrazolones 30

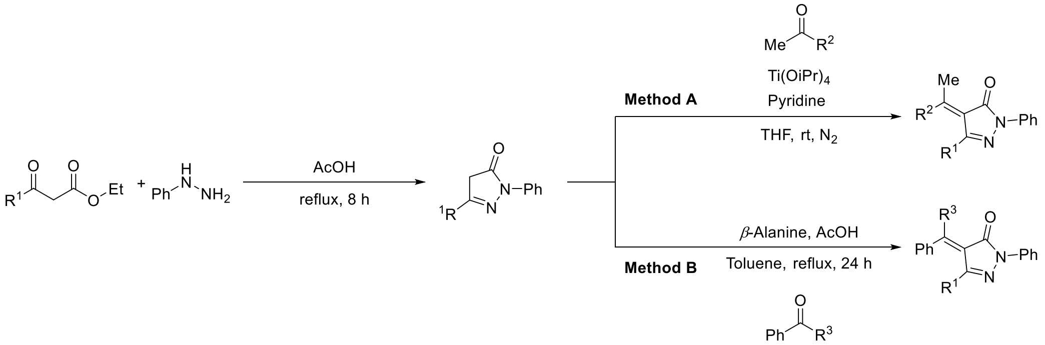

Step 1: The solution of phenylhydrazine (1.0 equiv.), ethyl benzoylacetate (1.2 equiv.) in acetic acid (2 M) was refluxed for 8 h and left to cool. The pure pyrazolone product was obtained as a yellow solid by recrystallization using acetic acid, which could be used directly in next step without further purification.

Step 2: Method A[4]: To a solution of the product from step 1 (1.0 equiv.) in dry THF (0.3 M) under argon atmosphere was added the corresponding ketones (1.2 equiv.) and pyridine (3.0 equiv.). The mixture was stirred at room temperature for 10 min followed by addition of titanium isopropoxide (2.5 equiv.). The solution was stirred at room

temperature for 48 h, then diluted with EtOAc and washed with 1M aqueous HCl, Na2CO3, and brine. The organic layer was dried over Na2SO4, removed under reduced pressure, and purified by column chromatography (n-Hexane/ Ethyl acetate = 40:1 to 20:1) to provide the desired product. **Method B[5]:** The mixture of the product from step 1 (1.0 equiv.), acetophenone derivatives (1.2 equiv.), *β*-Alanine (20 mol%) and AcOH (1.2 mL) in toluene (0.125 M) was refluxed for 24 h. The solvent of resulting reaction was evaporated. The residue was dissolved in EtOAc (40 mL) and washed with water and brine. The organic layer was dried over Na_2SO_4 and concentrated. The residue was subjected to column and then washed with *n*-hexane to provide desired product.

Note: The α-arylidene pyrazolone substrates **30a-f** was synthesized according to **Method A** from step 2 and **30g-h** was synthesized according to **Method B** from step 2. All characteristic data are consistent with previous literature[4,6].

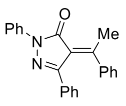

30a

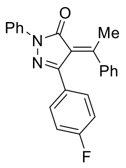

30b

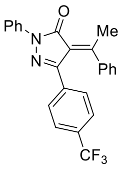

30c

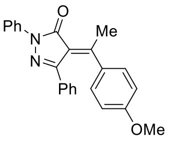

30d

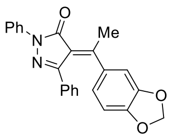

30e

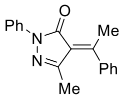

30f

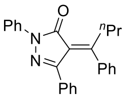

30g

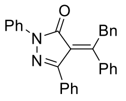

30h

3. Optimization of the Reaction Conditions for Chiral Spiropyrazolones Synthesis[a]

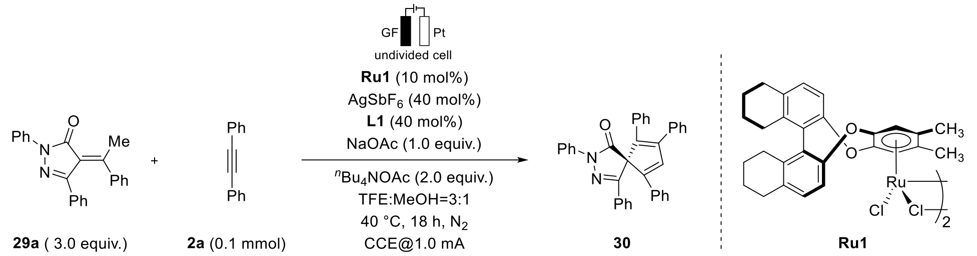

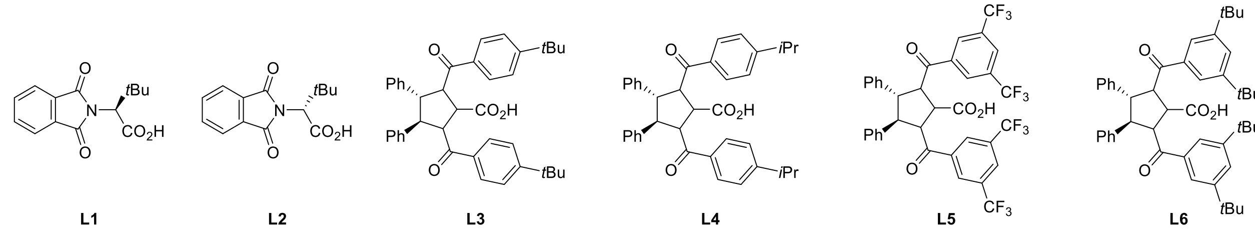

Entry	Deviations	Yield[b]	er[d]
1	none	75%(71%[c])	96:4
2	**L2** instead of **L1**	59%	94:6
3	**L3** instead of **L1**	35%	93:7
4	**L4** instead of **L1**	42%	94:6
5	**L5** instead of **L1**	50%	88.5:11.5
6	**L6** instead of **L1**	43%	91.5:8.5
7	**Ru1** (5 mol%)	27%	95.5:4.5
8	TFE:H_2O=3:1	20%	92:8
9	MeCN:MeOH=3:1	39%	84:16
10	MeCN:TFE=3:1	62%	85:15
11	MeCN	60%	88:12
12	^{n}BuN$_4$PF$_6$ instead of ^{n}BuN$_4$OAc	65%	95.5:4.5
13	**29a** (2.0 equiv.)	44%	95.5:4.5
14	DBU instead of NaOAc	54%	90.5:9.5
15	Et_3N instead of NaOAc	67%	88.5:11.5
16	Na_2CO_3 instead of NaOAc	70%	90.5:9.5
17	w/o electricity	30%	92.5:7.5
18	w/o **L1**	50%	90.5:9.5
19	w/o $AgSbF_6$	trace	-
20	w/o **Ru1**	trace	-

[a]Reaction conditions: **29a** (0.30 mmol, 3.0 equiv.), **2a** (0.10 mmol, 1.0 equiv.), **Ru1** (0.02 mmol, 10 mol%), $AgSbF_6$ (0.04 mmol, 40 mol%), **L1** (0.04 mmol, 40 mol%), NaOAc (0.10 mmol, 0.1 equiv.), ^{n}BuN$_4$OAc(0.2 mmol, 2.0 equiv.), TFE:MeOH=3:1 (4.0 mL), 40 °C, 18 h, CCE@1.0 mA, N_2. [b]Yields were determined by ^{1}H NMR using 1,3,5-trimethoxybenzene as the internal standard. [c]Isolation yields. [d]The er value was determined by HPLC analysis.

4. General Procedure and Products Characterization

General Procedure A for the synthesis of atroposelective indole products 3-29

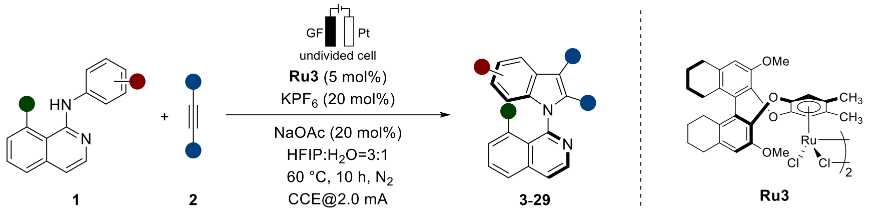

The electrolysis was carried out in an undivided cell setup. A GF anode (20 mm × 10 mm × 4 mm) and a platinum plate cathode (10 mm × 15 mm × 0.25 mm) with electrode holders made of stainless steel were used. A 10 mL Schlenk tube for electrolysis was charged with **1** (0.3 mmol, 3.0 equiv.), **2** (0.1 mmol, 1.0 equiv.), **Ru3** (6.3 mg, 0.005 mmol, 5 mol%), KPF_6 (3.7 mg, 0.02 mmol, 20 mol%), NaOAc (1.6 mg, 0.02 mmol, 20 mol%). Then a mixture of HFIP (3.0 mL) and H_2O (1.0 mL) were added. After sealing, the tube was charged with N_2. The resulting reaction mixture was then stirred at 60℃ with a constant current of 2.0 mA for 10 h. After completion of the reaction, the solvent was then removed under vacuum, and the residue was purified by column chromatography on silica gel to give corresponding product **3-28**. All products are characterized by NMR, HR-MS, optical rotations and HPLC chromatograms. Absolute configuration was determined by comparing the optical rotations of known products **3**, **16** and **17** with previous literature[2].

General Procedure B for the synthesis of chiral spiropyrazolones 31-45

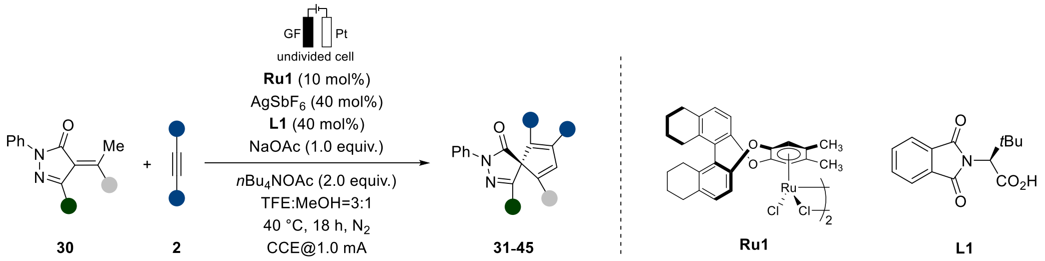

The electrolysis was carried out in an undivided cell setup. A GF anode (20 mm × 10 mm × 4 mm) and a platinum plate cathode (10 mm × 15 mm × 0.25 mm) with electrode holders made of stainless steel were used. A 10 mL Schlenk tube for electrolysis was charged **Ru1** (11.4 mg, 0.010 mmol, 10 mol%), $AgSbF_6$ (14 mg, 0.04 mmol, 40 mol%), **L1** (10.4 mg, 0.04 mmol, 40 mol%), NaOAc (8.2 mg, 0.1 mmol, 1.0 equiv.). Then a mixture of TFE (3.0 mL) and MeOH (1.0 mL) were added. The resulting mixture was stirred at 40℃ under air for 30 min. After that, **30** (0.3 mmol, 3.0 equiv.) and **2** (0.1 mmol, 1.0 equiv.) as well as $^{n}BuN_4OAc$ (60.3 mg, 0.2 mmol, 2.0 equiv.) were added, then the tube was

sealed and charged with N_2. The resulting reaction mixture was stirred at 40°C with a constant current of 1.0 mA for another 18h. After completion of the reaction, the solvent was then removed under vacuum, and the residue was purified by column chromatography on silica gel to give corresponding product **31-45**. All products are characterized by NMR, HR-MS, optical rotations and HPLC chromatograms. Absolute configuration was determined by comparing the optical rotations of known products with previous literature[4].

Reaction Setup

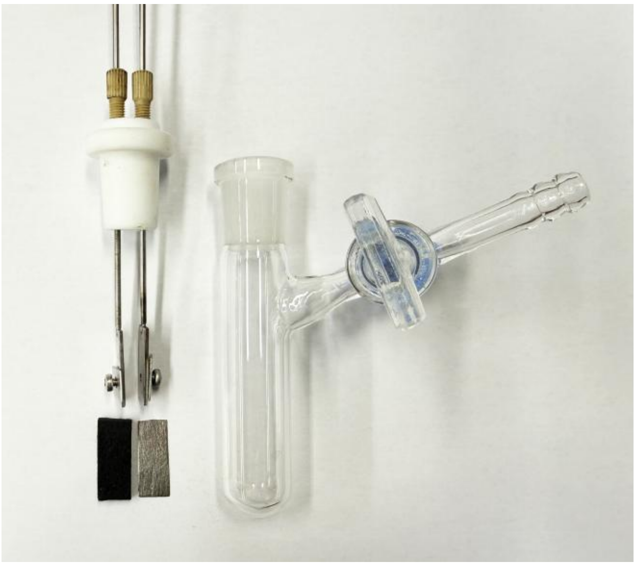

Figure S1. Reaction Setup

Characterization data of the Products

(*S*)-1-(2,3-diphenyl-1*H*-indol-1-yl)-8-phenylisoquinoline (3)

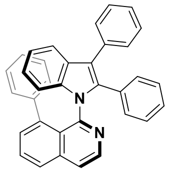

Prepared according to general procedure A at 0.1 mmol scale, column chromatography (*n*-hexane /EtOAc = 20:1) afforded the compound **3** as a yellow foam (47.0mg, 99%), with an enantiomeric ratio of 95.5:4.5.

^{1}H-NMR (500 MHz, $CDCl_3$) δ 8.66 (d, J = 5.5 Hz, 1H), 7.79 (d, J = 5.6 Hz, 1H), 7.77 (dd, J = 8.3, 1.2 Hz, 1H), 7.51 (dd, J = 8.2, 7.1 Hz, 1H), 7.46 – 7.42 (m, 1H), 7.35 – 7.32 (m, 1H), 7.28 – 7.24 (m, 3H), 7.23 – 7.12 (m, 4H), 7.11 – 7.07 (m, 1H), 7.02 – 6.98 (m, 3H), 6.92 – 6.87 (m, 1H), 6.86 – 6.82 (m, 2H), 6.68 – 6.65 (m, 2H), 6.56 (d, J = 7.6 Hz, 1H), 6.36 – 6.27 (m, 1H).

^{13}C-NMR (126 MHz, $CDCl_3$) δ 149.7 (C_q), 141.4 (CH), 140.0 (C_q), 139.9 (C_q), 138.8 (C_q), 138.1 (C_q), 135.2 (C_q), 135.0 (C_q), 132.0 (CH), 130.9 (C_q), 130.4 (CH), 129.9 (CH), 129.0 (CH), 128.3 (C_q), 128.2 (CH), 127.3 (CH), 127.1 (CH), 126.9 (CH), 126.8 (CH), 126.6 (CH), 126.3 (CH), 125.9 (CH), 125.8 (CH), 124.6 (C_q), 123.0 (CH), 121.5 (CH), 120.8 (CH), 119.1 (CH), 117.1 (C_q), 111.3 (CH).

HRMS (ESI): m/z $[M+H]^+$ calculated for $C_{35}H_{25}N_2^+$ 473.2012, found 473.2014.

$[\alpha]^D_{20}$ = -124.5 (c = 0.2, DCM).

HPLC Separation (Chiralpak® IA-3, *n*-hexane/*i*-PrOH 70/30, 1.0 mL/min, 250 nm): *t*r(minor) = 6.6 min, *t*r(major) = 8.6 min, 95.5:4.5 er.

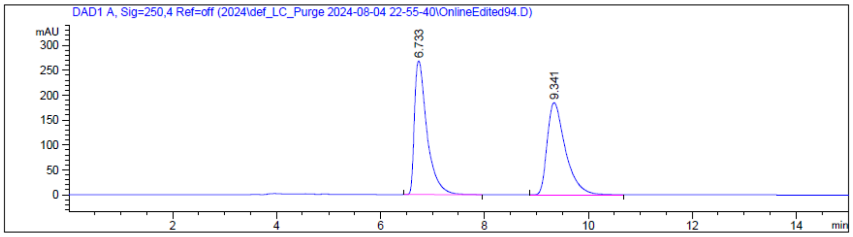

Peak #	RetTime [min]	Type	Width [min]	Area [mAU*s]	Height [mAU]	Area %
1	6.733	BB	0.2526	4491.04395	267.89532	49.9903
2	9.341	BB	0.3669	4492.79053	184.62335	50.0097

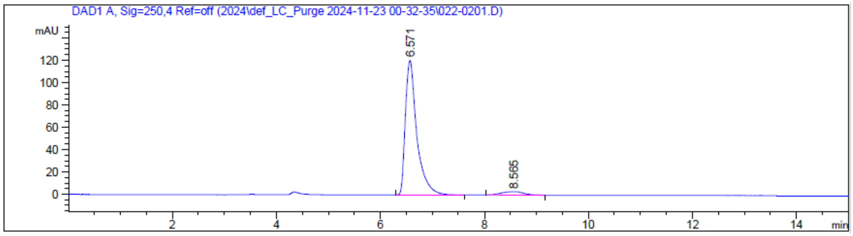

Peak #	RetTime [min]	Type	Width [min]	Area [mAU*s]	Height [mAU]	Area %
1	6.571	BB	0.2305	1848.41284	120.79942	95.4857
2	8.565	BB	0.3404	87.38823	3.03961	4.5143

(*S*)-1-(7-methyl-2,3-diphenyl-1*H*-indol-1-yl)-8-phenylisoquinoline (4)

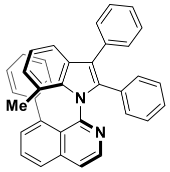

Prepared according to general procedure A at 0.1 mmol scale, column chromatography (*n*-hexane /EtOAc = 15:1) afforded the compound **4** as a yellow foam (39.7mg, 82%), with an enantiomeric ratio of 95:5.

^{1}H-NMR (500 MHz, $CDCl_3$) δ 8.62 (d, *J* = 5.5 Hz, 1H), 7.85 – 7.78 (m, 2H), 7.55 (dd, *J* = 8.1, 7.2 Hz, 1H), 7.25 – 7.12 (m, 6H), 7.08 (d, *J* = 7.6 Hz, 1H), 7.04 – 7.01 (m, 2H), 6.99 – 6.93 (m, 3H), 6.92 – 6.88 (m, 1H), 6.85 (t, *J* = 7.4 Hz, 2H), 6.79 – 6.75 (m, 2H), 6.57 (d, *J* = 7.6 Hz, 1H), 6.38 (t, *J* = 7.6 Hz, 1H), 1.98 (s, 3H).

^{13}C-NMR (126 MHz, $CDCl_3$) δ 151.1 (C_q), 140.5 (CH), 140.4 (C_q), 139.1 (C_q), 136.7 (C_q), 135.5 (C_q), 135.5 (C_q), 132.6 (CH), 131.3 (C_q), 130.9 (CH), 130.5 (CH), 129.8 (CH), 128.5 (C_q), 128.2 (CH), 128.1 (CH), 127.7 (CH), 127.2 (CH), 126.9 (C_q), 126.9 (CH), 126.7 (CH), 126.6 (CH), 126.2 (CH), 125.8 (CH), 125.6 (CH), 125.5 (CH), 122.7 (CH), 121.4 (C_q), 120.4 (CH), 117.3 (CH), 117.1 (C_q), 20.5 (CH_3).

HRMS (ESI): m/z $[M+H]^+$ calculated for $C_{36}H_{27}N_2^+$ 487.2169, found 487.2168.

$[\alpha]^D_{20}$ = -64.0 (c = 0.2, DCM).

HPLC Separation (Chiralpak® ID-3, *n*-hexane/*i*-PrOH 90/10, 1.0 mL/min, 250 nm): *t*r(minor) = 5.8 min, *t*r(major) = 9.5 min, 95:5 er.

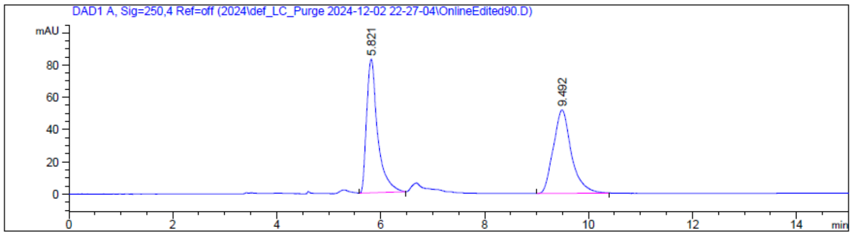

Peak #	RetTime [min]	Type	Width [min]	Area [mAU*s]	Height [mAU]	Area %
1	5.821	MM R	0.2464	1225.36438	82.87500	49.4270
2	9.492	MM R	0.4031	1253.77759	51.83540	50.5730

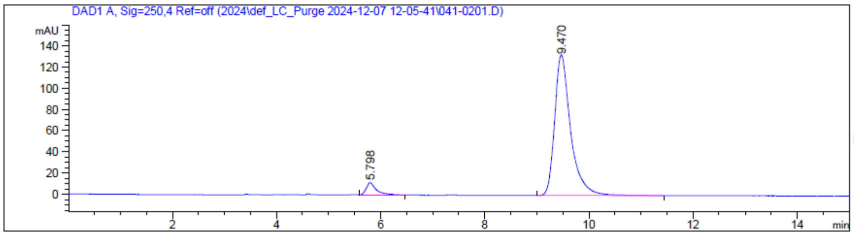

Peak #	RetTime [min]	Type	Width [min]	Area [mAU*s]	Height [mAU]	Area %
1	5.798	BB	0.1928	155.43394	11.49082	5.2056
2	9.470	MM R	0.3554	2830.48071	132.72864	94.7944

(*R*)-1-(7-fluoro-2,3-diphenyl-1*H*-indol-1-yl)-8-phenylisoquinoline (5)

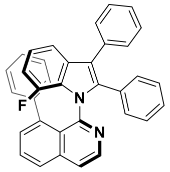

Prepared according to general procedure A at 0.1 mmol scale, column chromatography (*n*-hexane /EtOAc = 15:1) afforded the compound **6** as a yellow foam (45.9mg, 93%), with an enantiomeric ratio of 93:7.

^{1}H NMR (500 MHz, $CDCl_3$) δ 8.60 (d, *J* = 5.6 Hz, 1H), 7.82 – 7.79 (m, 2H), 7.54 (dd, *J* = 8.2, 7.1 Hz, 1H), 7.31 – 7.24 (m, 2H), 7.24 – 7.20 (m, 2H), 7.18 – 7.12 (m, 3H), 7.07 – 7.03 (m, 2H), 6.99 – 6.89 (m, 4H), 6.89 – 6.85 (m, 2H), 6.83 (d, *J* = 7.7 Hz, 1H), 6.76 – 6.72 (m, 2H), 6.48 (t, *J* = 7.4 Hz, 1H).

^{13}C NMR (126 MHz, $CDCl_3$) δ 150.5 (d, J_{C-F} = 244.5 Hz, C_q), 150.1 (C_q), 140.9 (CH), 140.0 (C_q), 139.4 (C_q), 139.4 (C_q), 136.5 (C_q), 134.9 (C_q), 132.0 (CH), 131.5 (d, J_{C-F} = 4.6 Hz, C_q), 130.6 (CH), 130.6 (C_q), 130.3 (CH), 129.7 (CH), 128.7 (CH), 128.3 (CH), 127.3 (CH), 127.3 (CH), 127.2 (CH), 126.7 (CH), 126.6 (CH), 126.3 (CH), 126.2 (CH), 126.0 (d, J_{C-F} = 8.8 Hz, C_q), 125.8 (CH), 125.5 (d, J_{C-F} = 1.2 Hz, C_q), 122.3 (CH), 120.4 (d, J_{C-F} = 6.5 Hz, CH), 117.4 (d, J_{C-F} = 2.2 Hz, C_q), 115.0 (d, J_{C-F} = 3.3 Hz, CH), 108.4 (d, J_{C-F} = 17.8 Hz, CH).

^{19}F NMR (471 MHz, $CDCl_3$) δ -130.45 (dd, J_{F-H} = 12.0, 4.5 Hz).

HRMS (ESI): m/z $[M+H]^+$ calculated for $C_{35}H_{24}FN_2^+$ 491.1918, found 491.1916.

$[\alpha]^D_{20}$ = -48.0 (c = 0.2, DCM).

HPLC Separation (Chiralpak® IE-3, *n*-hexane/*i*-PrOH 90/10, 1.0 mL/min, 230 nm): *t*r(major) = 9.1 min, *t*r(minor) = 10.5 min, 93:7 er.

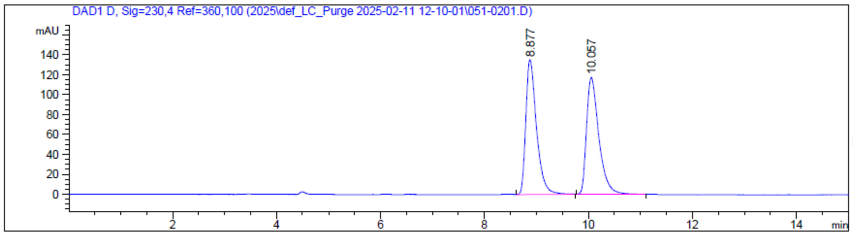

Peak #	RetTime [min]	Type	Width [min]	Area [mAU*s]	Height [mAU]	Area %
1	8.877	BV R	0.2132	1922.06897	134.89468	50.0522
2	10.057	MM R	0.2733	1918.06287	116.96372	49.9478

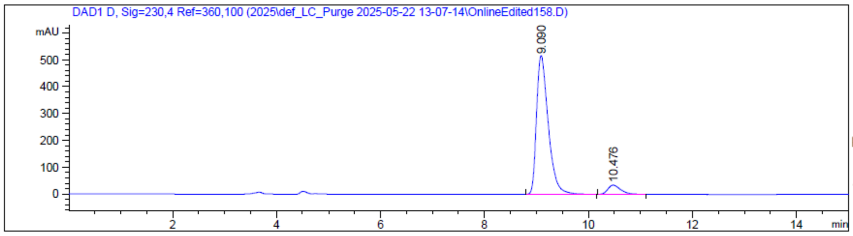

Peak #	RetTime [min]	Type	Width [min]	Area [mAU*s]	Height [mAU]	Area %
1	9.090	MM R	0.2600	8071.81152	517.34540	93.2822
2	10.476	MM R	0.2855	581.30151	33.93341	6.7178

(*S*)-1-(6-methyl-2,3-diphenyl-1*H*-indol-1-yl)-8-phenylisoquinoline (6)

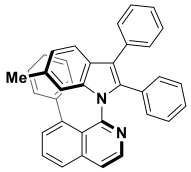

Prepared according to general procedure A at 0.1 mmol scale, column chromatography (*n*-hexane /EtOAc = 15:1) afforded the compound **6** as a yellow foam (48.0 mg, 99%), with an enantiomeric ratio of 93:7.

^{1}H-NMR (600 MHz, $CDCl_3$) δ 8.67 (d, *J* = 5.5 Hz, 1H), 7.78 (d, *J* = 5.5 Hz, 1H), 7.76 (dd, *J* = 8.3, 1.2 Hz, 1H), 7.50 (dd, *J* = 8.2, 7.1 Hz, 1H), 7.26 – 7.23 (m, 3H), 7.22 – 7.20 (m, 1H), 7.20 – 7.11 (m, 4H), 7.02 – 6.98 (m, 3H), 6.94 – 6.91 (m, 1H), 6.89 – 6.85 (m, 1H), 6.84 – 6.80 (m, 2H), 6.64 – 6.61 (m, 2H), 6.57 (d, *J* = 7.4 Hz, 1H), 6.33 (s, 1H), 2.48 (s, 3H).

^{13}C-NMR (151 MHz, $CDCl_3$) δ 149.9 (C_q), 141.4 (CH), 140.0 (C_q), 140.0 (C_q), 138.9 (C_q), 138.4 (C_q), 135.4 (C_q), 134.3 (C_q), 132.8 (C_q), 131.9 (CH), 131.1 (C_q), 130.4 (CH), 130.3 (CH), 129.8 (CH), 129.1 (CH), 128.1 (CH), 127.2 (CH), 127.1 (CH), 126.9 (CH), 126.6 (CH), 126.5 (CH), 126.3 (C_q), 126.3 (CH), 125.8 (CH), 124.6 (C_q), 122.5 (CH), 121.4 (CH), 118.8 (CH), 117.0 (C_q), 111.1 (CH), 22.1 (CH_3).

HRMS (ESI): m/z $[M+H]^+$ calculated for C36H27N2+ 487.2169, found 487.2169.

$[\alpha]^D{}_{20}$ = -81.5 (c = 0.2, DCM).

HPLC Separation (Chiralpak® IE-3, *n*-hexane/*i*-PrOH 90/10, 1.0 mL/min, 250 nm): *t*r(minor) = 9.0 min, *t*r(major) = 25.1 min, 93:7 er.

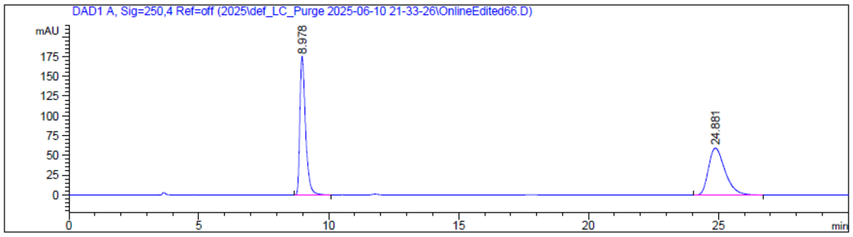

Peak #	RetTime [min]	Type	Width [min]	Area [mAU*s]	Height [mAU]	Area %
1	8.978	MM R	0.2525	2656.42993	175.36816	50.0344
2	24.881	MM R	0.7429	2652.77539	59.51276	49.9656

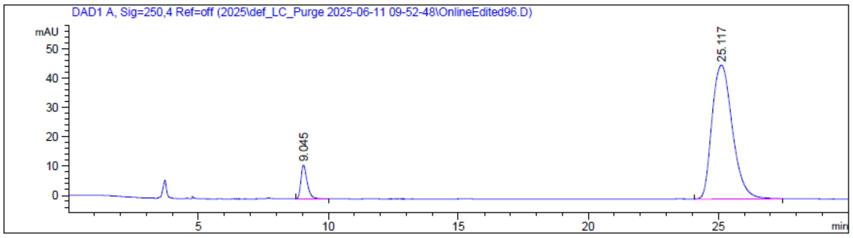

Peak #	RetTime [min]	Type	Width [min]	Area [mAU*s]	Height [mAU]	Area %
1	9.045	BB	0.2493	191.64893	11.50828	7.1645
2	25.117	MM R	0.9081	2483.34155	45.57590	92.8355

Methyl (*S*)-2,3-diphenyl-1-(8-phenylisoquinolin-1-yl)-1*H*-indole-6-carboxylate (7)

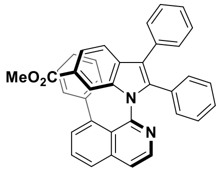

Prepared according to general procedure A at 0.1 mmol scale, column chromatography (*n*-hexane /EtOAc = 6:1) afforded the compound **7** as a yellow foam (38.6 mg, 73%), with an enantiomeric ratio of 91:9.

^{1}H-NMR (500 MHz, $CDCl_3$) δ 8.69 (d, *J*=5.5, 1H), 8.17 (d, *J*=0.9, 1H), 7.84 (d, *J*=5.5, 1H), 7.80 (dd, *J*=8.4, 1.4, 2H), 7.52 (dd, *J*=8.2, 7.1, 1H), 7.37 (dd, *J*=8.4, 0.7, 1H), 7.30 – 7.26 (m, 2H), 7.24 – 7.20 (m, 1H), 7.17 (dd, *J*=7.1, 1.2, 2H), 7.11 (d, *J*=7.7, 1H), 7.01 – 6.98 (m, 3H), 6.95 – 6.91 (m, 1H), 6.85 (t, *J*=7.5, 2H), 6.69 – 6.65 (m, 2H), 6.54 (d, *J*=7.6, 1H), 6.30 (t, *J*=7.5, 1H), 3.93 (s, 3H).

^{13}C-NMR (126 MHz, $CDCl_3$) δ 168.3 (C_q), 149.0 (C_q), 141.5 (CH), 140.0 (C_q), 139.6 (C_q), 138.8 (C_q), 138.4 (C_q), 137.5 (C_q), 134.5 (C_q), 132.2 (CH), 131.7 (C_q), 130.4 (CH), 130.3 (CH), 130.2 (C_q), 130.0 (CH), 129.1 (CH), 128.4 (CH), 127.4 (CH), 127.3 (CH), 127.0 (CH), 126.7 (CH), 126.5 (CH), 126.3 (CH), 125.9 (CH), 124.6 (C_q), 124.4 (C_q), 122.1 (CH), 121.9 (CH), 118.8 (CH), 117.2 (C_q), 113.8 (CH), 52.0 (CH_3).

HRMS (ESI): m/z $[M+H]^+$ calculated for $C_{37}H_{27}N_2O_2^+$ 531.2067, found 531.2065.

$[\alpha]^D_{20}$ = -48.0 (c = 0.1, DCM).

HPLC Separation (Chiralpak® IA-3, *n*-hexane/*i*-PrOH 80/20, 1.0 mL/min, 250 nm): *t*r(minor) = 5.2 min, *t*r(major) = 9.6 min, 91:9 er.

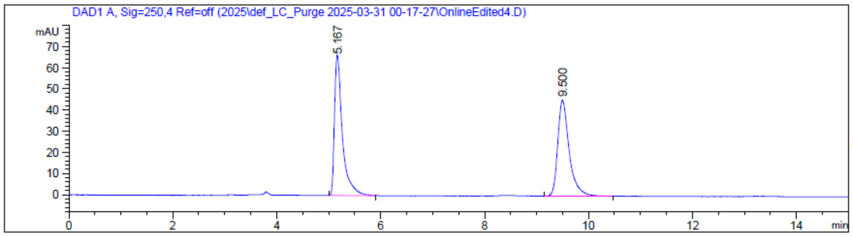

Peak #	RetTime [min]	Type	Width [min]	Area [mAU*s]	Height [mAU]	Area %
1	5.167	MM R	0.1755	697.94653	66.26739	50.0495
2	9.500	MM R	0.2558	696.56604	45.37939	49.9505

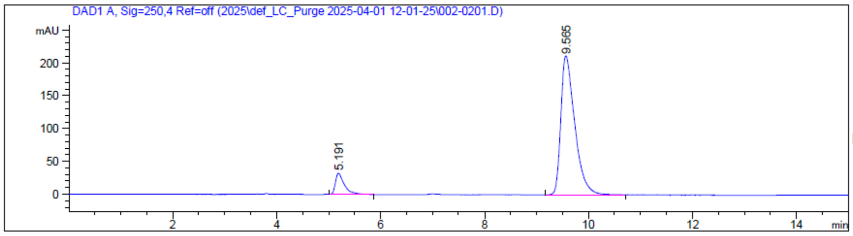

Peak #	RetTime [min]	Type	Width [min]	Area [mAU*s]	Height [mAU]	Area %
1	5.191	FM R	0.2010	388.59311	32.22226	8.9876
2	9.565	MM R	0.3096	3935.05347	211.82576	91.0124

(*S*)-1-(6-chloro-2,3-diphenyl-1*H*-indol-1-yl)-8-phenylisoquinoline (8)

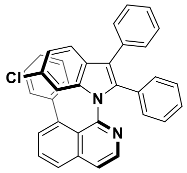

Prepared according to general procedure A at 0.1 mmol scale, column chromatography (*n*-hexane /EtOAc = 15:1) afforded the compound **8** as a yellow foam (36.8mg, 73%), with an enantiomeric ratio of 94:6.

^{1}H NMR (600 MHz, $CDCl_3$) δ 8.69 (d, *J* = 5.5 Hz, 1H), 7.84 (d, *J* = 5.5 Hz, 1H), 7.81 (dd, *J* = 8.2, 1.2 Hz, 1H), 7.55 (dd, *J* = 8.2, 7.1 Hz, 1H), 7.46 (d, *J* = 1.8 Hz, 1H), 7.32 – 7.27 (m, 3H), 7.26 – 7.19 (m, 3H), 7.16 (d, *J* = 7.6 Hz, 1H), 7.09 (dd, *J* = 8.5, 1.8 Hz, 1H), 7.05 (tt, *J* = 7.4, 1.3 Hz, 1H), 7.03 – 7.00 (m, 2H), 6.96 – 6.92 (m, 1H), 6.89 – 6.85 (m, 2H), 6.70 – 6.67 (m, 2H), 6.63 (d, *J* = 7.7 Hz, 1H), 6.47 (t, *J* = 7.5 Hz, 1H).

^{13}C NMR (151 MHz, $CDCl_3$) δ 149.1 (C_q), 141.4 (CH), 140.0 (C_q), 139.7 (C_q), 138.9 (C_q), 138.3 (C_q), 135.8 (C_q), 134.7 (C_q), 132.2 (CH), 130.5 (C_q), 130.3 (CH), 130.3 (CH), 130.0 (CH), 129.1 (CH), 128.7 (C_q), 128.3 (CH), 127.3 (CH), 127.2 (CH), 127.1 (CH), 127.0 (CH), 126.9 (C_q), 126.7 (CH), 126.5 (CH), 126.2 (CH), 125.9 (CH), 124.5 (C_q), 122.0 (CH), 121.4 (CH), 120.1 (CH), 117.0 (C_q), 111.3 (CH).

HRMS (ESI): m/z $[M+H]^+$ calculated for $C_{35}H_{24}ClN_2^+$ 507.1623, found 507.1623.

$[\alpha]^D_{20}$ = -75.0 (c = 0.1, DCM).

HPLC Separation (Chiralpak® ID-3, *n*-hexane/*i*-PrOH 90/10, 1.0 mL/min, 250 nm): *t*r(minor) = 6.1 min, *t*r(major) = 15.0 min, 94:6 er.

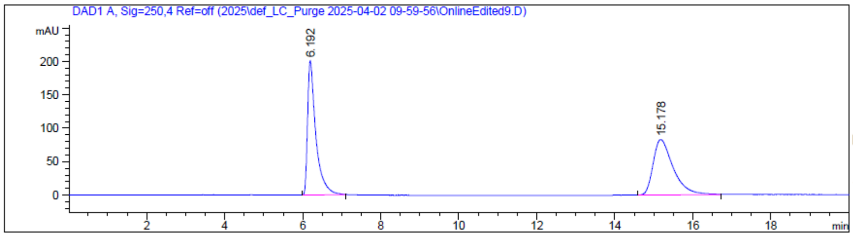

Peak #	RetTime [min]	Type	Width [min]	Area [mAU*s]	Height [mAU]	Area %
1	6.192	MM R	0.2461	2973.61230	201.39709	49.9357
2	15.178	MM R	0.5975	2981.27148	83.15916	50.0643

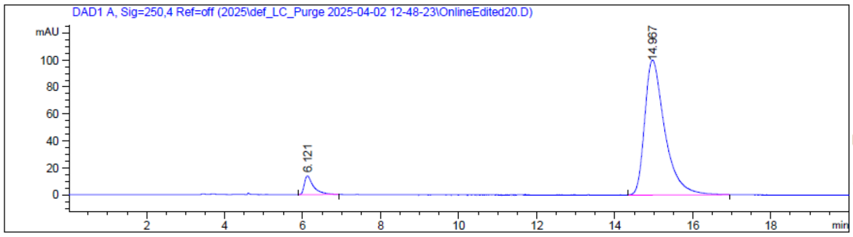

Peak #	RetTime [min]	Type	Width [min]	Area [mAU*s]	Height [mAU]	Area %
1	6.121	BB	0.2329	230.50618	13.94245	6.0450
2	14.967	MM R	0.5950	3582.64600	100.36021	93.9550

(*S*)-1-(5-methoxy-2,3-diphenyl-1H-indol-1-yl)-8-phenylisoquinoline (9)

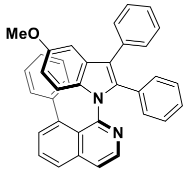

Prepared according to general procedure A at 0.1 mmol scale, column chromatography (*n*-hexane /EtOAc = 15:1) afforded the compound **9** as a yellow foam (23.6mg, 47%), with an enantiomeric ratio of 92:8.

^{1}H NMR (400 MHz, $CDCl_3$) δ 8.64 (d, *J* = 5.5 Hz, 1H), 7.79 – 7.74 (m, 2H), 7.49 (dd, *J* = 8.2, 7.1 Hz, 1H), 7.35 (dd, *J* = 8.8, 0.5 Hz, 1H), 7.29 – 7.24 (m, 2H), 7.22 – 7.10 (m, 4H), 7.06 – 7.01 (m, 1H), 7.00 – 6.97 (m, 2H), 6.93 – 6.86 (m, 2H), 6.85 – 6.79 (m, 3H), 6.65 – 6.60 (m, 2H), 6.57 (d, *J* = 6.6 Hz, 1H), 6.46 – 6.36 (m, 1H), 3.81 (s, 3H).

^{13}C NMR (101 MHz, $CDCl_3$) δ 155.2 (C_q), 149.8 (C_q), 141.4 (CH), 140.0 (C_q), 139.9 (C_q), 138.8 (C_q), 135.6 (C_q), 135.4 (C_q), 133.5 (C_q), 131.9 (CH), 130.9 (C_q), 130.4 (CH), 129.9 (CH), 128.9 (CH), 128.7 (C_q), 128.3 (CH), 127.3 (CH), 127.1 (CH), 126.9 (CH), 126.7 (CH), 126.6 (CH), 126.3 (CH), 125.9 (CH), 125.9 (CH), 124.5 (C_q), 121.4 (CH), 116.9 (C_q), 113.0 (CH), 112.1 (CH), 101.0 (CH), 56.2 (CH_3).

HRMS (ESI): m/z $[M+H]^+$ calculated for $C_{36}H_{27}N_2O^+$ 503.2118, found 503.2121.

$[\alpha]^D_{20}$ = -76.0 (c = 0.1, DCM).

HPLC Separation (Chiralpak® IA-3, *n*-hexane/*i*-PrOH 60/40, 1.0 mL/min, 250 nm): *t*r(minor) = 17.5 min, *t*r(major) = 25.7 min, 92:8 er.

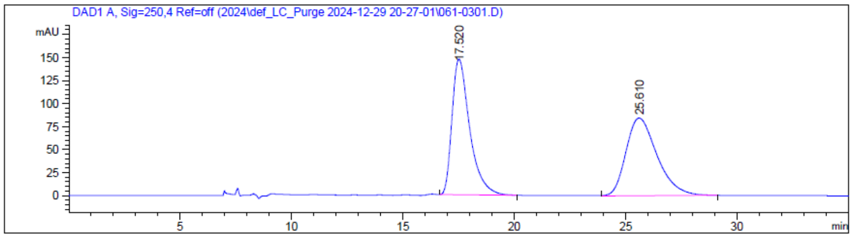

Peak #	RetTime [min]	Type	Width [min]	Area [mAU*s]	Height [mAU]	Area %
1	17.520	BB	0.7772	8143.79199	147.49397	49.9231
2	25.610	MM R	1.6104	8168.88330	84.35283	50.0769

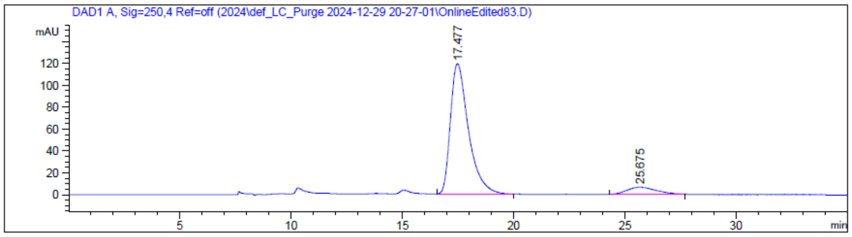

Peak #	RetTime [min]	Type	Width [min]	Area [mAU*s]	Height [mAU]	Area %
1	17.477	BB	0.7519	6720.28418	119.16615	91.9753
2	25.675	MM R	1.5111	586.33331	6.46691	8.0247

(*S*)-1-(2,3-diphenyl-5-(trifluoromethyl)-1*H*-indol-1-yl)-8-phenylisoquinoline (10)

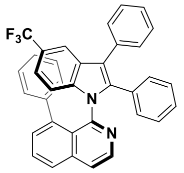

Prepared according to general procedure A at 0.1 mmol scale, column chromatography (*n*-hexane /EtOAc = 15:1) afforded the compound **10** as a yellow foam (47.1mg, 87%), with an enantiomeric ratio of 94:6.

^{1}H NMR (500 MHz, $CDCl_3$) δ 8.66 (d, J = 5.5 Hz, 1H), 7.81 (d, J = 5.5 Hz, 1H), 7.78 (dd, J = 8.3, 1.2 Hz, 1H), 7.52 (dd, J = 8.2, 7.1 Hz, 1H), 7.47 (dd, J = 1.7, 0.7 Hz, 1H), 7.35 – 7.30 (m, 2H), 7.30 – 7.26 (m, 2H), 7.24 – 7.16 (m, 3H), 7.12 (d, J = 7.5 Hz, 1H), 7.08 – 7.05 (m, 1H), 6.99 – 6.96 (m, 2H), 6.93 – 6.90 (m, 1H), 6.87 – 6.83 (m, 2H), 6.67 – 6.63 (m, 2H), 6.55 (d, J = 7.7 Hz, 1H), 6.44 (d, J = 7.6 Hz, 1H).

^{13}C NMR (126 MHz, $CDCl_3$) δ 149.0 (C_q), 141.4 (CH), 140.0 (C_q), 139.6 (C_q), 139.2 (C_q), 138.8 (C_q), 137.0 (C_q), 134.2 (Cq), 132.3 (CH), 130.4 (CH), 130.3 (CH), 130.2 (C_q), 130.1 (CH), 128.9 (CH), 128.5 (CH), 127.6 (C_q), 127.3 (CH), 127.0 (CH), 126.8 (CH), 126.6 (CH), 126.5 (CH), 125.8 (CH), 125.5 (q, $J_{C\text{-}F}$ = 271.4 Hz, C_q), 124.5 (C_q), 123.2 (q, $J_{C\text{-}F}$ = 31.6 Hz, C_q), 122.2 (CH), 119.6 (q, $J_{C\text{-}F}$ = 3.6 Hz, CH), 117.6 (C_q), 116.9 (q, $J_{C\text{-}F}$ = 4.3 Hz, CH), 111.8 (CH).

^{19}F NMR (471 MHz, $CDCl_3$) δ -60.2.

HRMS (ESI): m/z $[M+H]^+$ calculated for $C_{36}H_{24}F_3N_2^+$ 541.1886, found 541.1887.

$[\alpha]^{D}_{20}$ = -107.0 (c = 0.1, DCM).

HPLC Separation (Chiralpak® IE-3, *n*-hexane/*i*-PrOH 90/10, 1.0 mL/min, 250 nm): *t*r(minor) = 7.0 min, *t*r(major) = 7.9 min, 94:6 er.

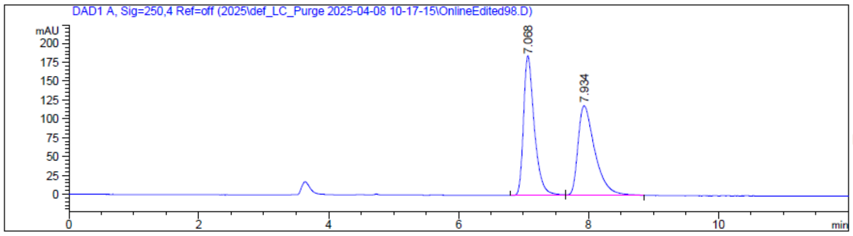

Peak #	RetTime [min]	Type	Width [min]	Area [mAU*s]	Height [mAU]	Area %
1	7.068	BV	0.1738	2141.14771	185.24583	50.0082
2	7.934	VV R	0.2661	2140.44849	118.86292	49.9918

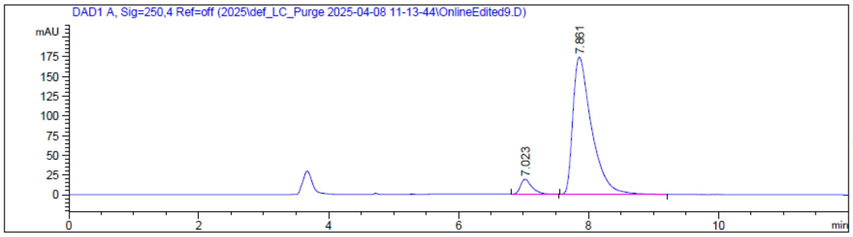

Peak #	RetTime [min]	Type	Width [min]	Area [mAU*s]	Height [mAU]	Area %
1	7.023	MM R	0.2016	230.46094	19.04968	6.2261
2	7.861	MM R	0.3325	3471.09399	173.96481	93.7739

(*S*)-1-(5-bromo-2,3-diphenyl-1H-indol-1-yl)-8-phenylisoquinoline (11)

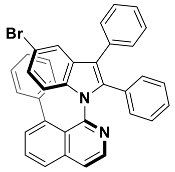

Prepared according to general procedure A at 0.1 mmol scale, column chromatography (*n*-hexane /EtOAc = 15:1) afforded the compound **11** as a yellow solid (44.9mg, 81%), with an enantiomeric ratio of 96:4.

^{1}H NMR (500 MHz, $CDCl_3$) δ 8.7 (d, J = 5.5 Hz, 1H), 7.8 (d, J = 5.5 Hz, 1H), 7.8 (dd, J = 8.3, 1.2 Hz, 1H), 7.5 (dd, J = 8.2, 7.1 Hz, 1H), 7.5 (dd, J = 1.7, 0.7 Hz, 1H), 7.4 – 7.3 (m, 2H), 7.3 – 7.3 (m, 2H), 7.2 – 7.2 (m, 3H), 7.1 (d, J = 7.5 Hz, 1H), 7.1 – 7.0 (m, 1H), 7.0 – 7.0 (m, 2H), 6.9 – 6.9 (m, 1H), 6.9 – 6.8 (m, 2H), 6.7 – 6.6 (m, 2H), 6.5 (d, J = 7.7 Hz, 1H), 6.5 – 6.4 (m, 1H).

^{13}C NMR (126 MHz, $CDCl_3$) δ 149.2 (C_q), 141.4 (CH), 139.9 (C_q), 139.7 (C_q), 138.8 (C_q), 136.7 (C_q), 136.2 (C_q), 134.4 (C_q), 132.2 (CH), 130.4 (CH), 130.3 (C_q), 130.3 (CH), 130.0 (CH), 129.9 (C_q), 128.9 (CH), 128.4 (CH), 127.3 (CH), 127.2 (CH), 127.2 (CH), 127.0 (CH), 126.9 (CH), 126.4 (CH), 126.3 (CH), 126.0 (CH), 125.7 (CH), 124.4 (C_q), 121.9 (CH), 121.6 (CH), 116.5 (C_q), 114.0 (C_q), 112.9 (CH).

HRMS (ESI): m/z $[M+H]^+$ calculated for $C_{35}H_{24}BrN_2^+$ 551.1117, found 551.1119.

$[\alpha]^{D}_{20}$ = -66.0 (c = 0.1, DCM).

HPLC Separation (Chiralpak® IE-3, *n*-hexane/*i*-PrOH 70/30, 1.0 mL/min, 250 nm): *t*r(minor) = 6.7 min, *t*r(major) = 9.3 min, 96:4 er.

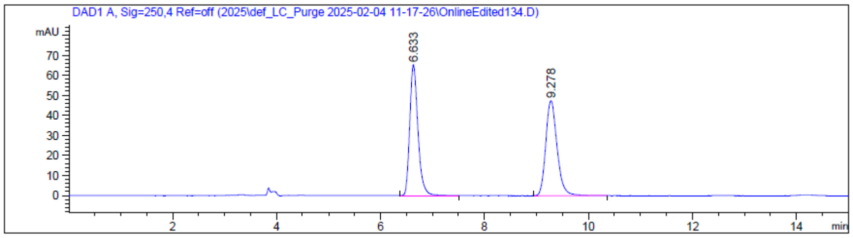

Peak #	RetTime [min]	Type	Width [min]	Area [mAU*s]	Height [mAU]	Area %
1	6.633	MM R	0.1827	716.71106	65.39278	50.0023
2	9.278	MM R	0.2517	716.64563	47.46132	49.9977

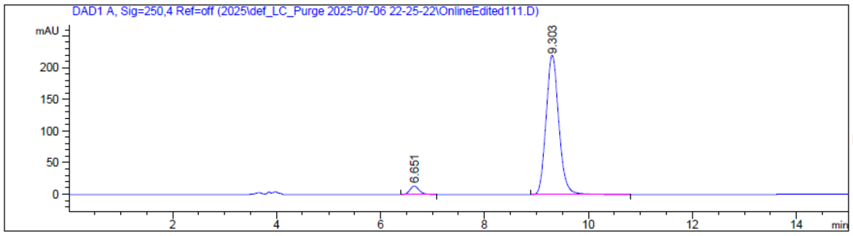

Peak #	RetTime [min]	Type	Width [min]	Area [mAU*s]	Height [mAU]	Area %
1	6.651	MM R	0.2044	166.25087	13.55671	4.2325
2	9.303	BB	0.2621	3761.70776	220.37248	95.7675

(*S*)-1-(5,7-dimethyl-2,3-diphenyl-1*H*-indol-1-yl)-8-phenylisoquinoline (12)

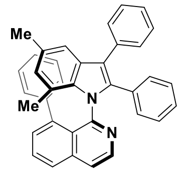

Prepared according to general procedure A at 0.1 mmol scale at 70°C, column chromatography (DCM /*n*-hexane = 2:1) afforded the compound **12** as a yellow foam (42.0 mg, 86%), with an enantiomeric ratio of 97:3.

^{1}H NMR (500 MHz, $CDCl_3$) δ 8.61 (dd, *J* = 5.6, 2.2 Hz, 1H), 7.83 – 7.77 (m, 2H), 7.57 – 7.51 (m, 1H), 7.26 – 7.23 (m, 2H), 7.21 – 7.13 (m, 3H), 7.07 (d, *J* = 6.9 Hz, 1H), 7.03 – 6.96 (m, 4H), 6.91 – 6.82 (m, 3H), 6.80 – 6.72 (m, 3H), 6.57 (d, *J* = 7.5 Hz, 1H), 6.43 – 6.34 (m, 1H), 2.36 (s, 3H), 1.94 (s, 3H).

^{13}C NMR (126 MHz, $CDCl_3$) δ 151.2 (C_q), 140.5 (CH), 140.4 (C_q), 139.1 (C_q), 139.1 (C_q), 135.7 (C_q), 135.5 (C_q), 135.2 (C_q), 132.5 (CH), 131.4 (C_q), 130.9 (CH), 130.6 (CH), 129.7 (CH), 129.5 (C_q), 128.7 (C_q), 128.2 (CH), 128.1 (CH), 127.7 (CH), 127.3 (CH), 127.1 (CH), 126.9 (CH), 126.6 (CH), 126.2 (CH), 125.7 (CH), 125.6 (CH), 122.5 (CH), 120.9 (C_q), 116.8 (CH), 116.7 (C_q), 21.4 (CH_3), 20.3 (CH_3).

HRMS (ESI): m/z $[M+H]^+$ calculated for $C_{37}H_{29}N_2^+$ 501.2325, found 501.2326.

$[\alpha]^D_{20}$ = -43.0 (c = 0.1, DCM).

HPLC Separation (Chiralpak® ID-3, *n*-hexane/*i*-PrOH 95/5, 1.0 mL/min, 250 nm): *t*r(minor) = 7.1 min, *t*r(major) = 9.0 min, 97:3 er.

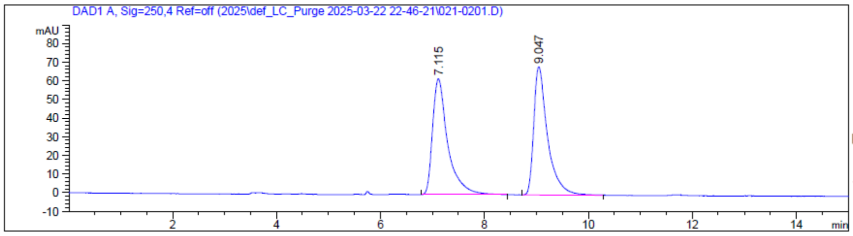

Peak #	RetTime [min]	Type	Width [min]	Area [mAU*s]	Height [mAU]	Area %
1	7.115	MM R	0.3383	1262.29248	62.19682	49.9884
2	9.047	MM R	0.3061	1262.88062	68.76819	50.0116

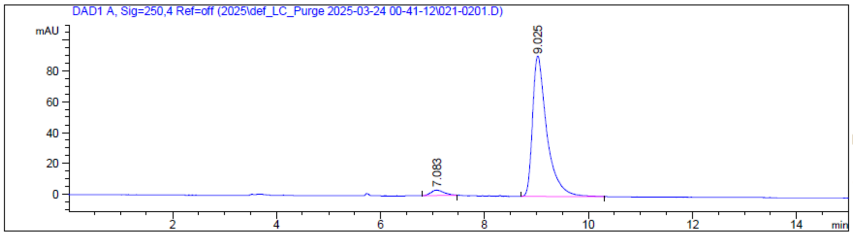

Peak #	RetTime [min]	Type	Width [min]	Area [mAU*s]	Height [mAU]	Area %
1	7.083	MM R	0.2946	61.67361	3.48855	3.4233
2	9.025	MM R	0.3179	1739.91772	91.21192	96.5767

(*S*)-2,3-diphenyl-1-(8-phenylisoquinolin-1-yl)-1*H*-benzo[*g*]indole (13)

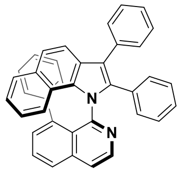

Prepared according to general procedure A at 0.1 mmol scale, column chromatography (*n*-hexane /EA = 15:1) afforded the compound **13** as yellow foam (35.8 mg, 68%), with an enantiomeric ratio of 95.5:4.5.

^{1}H NMR (500 MHz, $CDCl_3$) δ 8.72 (d, *J* = 5.5 Hz, 1H), 7.94 (d, *J* = 5.5 Hz, 1H), 7.92 – 7.90 (m, 1H), 7.87 (dd, *J* = 8.3, 1.3 Hz, 1H), 7.57 (dd, *J* = 8.2, 7.1 Hz, 1H), 7.49 (d, *J* = 8.7 Hz, 1H), 7.44 (d, *J* = 8.7 Hz, 1H), 7.35 – 7.31 (m, 1H), 7.31 – 7.27 (m, 2H), 7.24 – 7.20 (m, 1H), 7.17 (dd, *J* = 7.1, 1.3 Hz, 1H), 7.14 – 7.06 (m, 6H), 6.93 – 6.85 (m, 4H), 6.79 – 6.74 (m, 2H), 6.28 (d, *J* = 7.9 Hz, 1H), 6.01 – 5.94 (m, 1H).

^{13}C NMR (126 MHz, $CDCl_3$) δ 151.6 (C_q), 141.3 (CH), 140.2 (C_q), 139.8 (C_q), 139.0 (C_q), 135.2 (C_q), 135.0 (C_q), 132.7 (CH), 132.1 (C_q), 131.5 (C_q), 131.3 (C_q), 130.9 (CH), 130.6 (CH), 130.1 (CH), 129.4 (CH), 129.2 (CH), 128.2 (CH), 127.3 (CH), 127.2 (CH), 126.9 (CH), 126.7 (CH), 126.6 (CH), 126.2 (CH), 126.0 (C_q), 126.0 (CH), 125.4 (CH), 125.0 (CH), 124.8 (C_q), 123.3 (CH), 123.2 (CH), 123.2 (C_q), 121.9 (CH), 121.4 (CH), 119.1 (CH), 118.4 (C_q).

HRMS (ESI): m/z [M+H]+ calculated for $C_{39}H_{27}N_2^+$ 523.2169, found 523.2167.

$[\alpha]^D_{20}$ = -64.0 (c = 0.1, DCM).

HPLC Separation (Chiralpak® ID-3, *n*-hexane/*i*-PrOH 95/5, 1.0 mL/min, 250 nm): *t*r(minor) = 7.1 min, *t*r(major) = 9.0 min, 95.5:4.5 er.

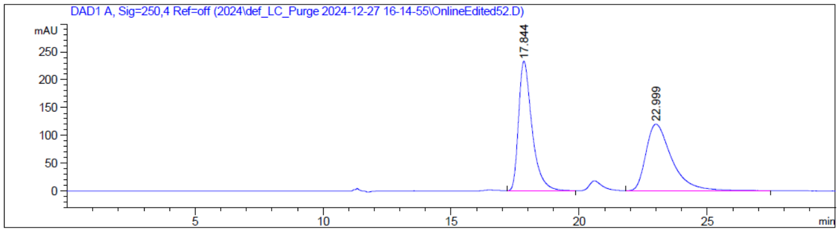

Peak #	RetTime [min]	Type	Width [min]	Area [mAU*s]	Height [mAU]	Area %
1	17.844	BB	0.5395	8472.51465	232.84856	50.2532
2	22.999	MM R	1.1677	8387.14648	119.70579	49.7468

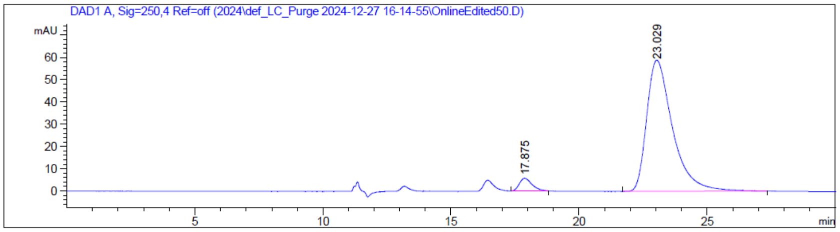

Peak #	RetTime [min]	Type	Width [min]	Area [mAU*s]	Height [mAU]	Area %
1	17.875	MM R	0.5675	195.96324	5.75501	4.5059
2	23.029	MM R	1.1737	4153.08594	58.97318	95.4941

(*S*)-1-(2,3-diphenyl-1*H*-indol-1-yl)-8-(4-fluorophenyl)isoquinoline (14)

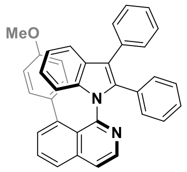

Prepared according to general procedure A at 0.1 mmol scale, column chromatography (n-hexane /EtOAc = 15:1) afforded the compound **14** as yellow foam (32.8mg, 65%), with an enantiomeric ratio of 95:5.

^{1}H NMR (400 MHz, $CDCl_3$) δ 8.66 (d, *J* = 5.5 Hz, 1H), 7.78 (d, *J* = 5.6 Hz, 1H), 7.73 (dd, *J* = 8.2, 1.2 Hz, 1H), 7.56 – 7.52 (m, 1H), 7.47 (dd, *J* = 8.2, 7.1 Hz, 1H), 7.41 – 7.36 (m, 1H), 7.30 – 7.25 (m, 3H), 7.22 – 7.18 (m, 1H), 7.15 – 7.10 (m, 2H), 7.06 (d, *J* = 6.9 Hz, 1H), 7.03 – 7.00 (m, 2H), 6.90 – 6.85 (m, 1H), 6.84 – 6.79 (m, 2H), 6.73 (d, *J* = 5.1 Hz, 1H), 6.63 – 6.58 (m, 2H), 6.43 (d, *J* = 8.1 Hz, 1H), 5.78 (d, *J* = 8.3 Hz, 1H), 3.69 (s, 3H).

^{13}C NMR (101 MHz, $CDCl_3$) δ 158.5 (C_q), 149.7 (C_q), 141.2 (CH), 140.0 (C_q), 139.6 (C_q), 138.2 (C_q), 135.3 (C_q), 135.0 (C_q), 131.6 (CH), 131.3 (C_q), 130.8 (C_q), 130.4 (CH), 130.3 (CH), 130.2 (CH), 129.8 (CH), 128.5 (C_q), 128.3 (CH), 127.1 (CH), 126.7 (CH), 125.9 (CH), 125.9 (CH), 124.6 (C_q), 123.0 (CH), 121.5 (CH), 120.8 (CH), 119.0 (CH), 117.0 (C_q), 113.0 (CH), 111.4 (CH), 110.5 (CH), 55.2 (CH_3).

HRMS (ESI): m/z $[M+H]^+$ calculated for $C_{36}H_{27}N_2O^+$ 503.2118, found 503.2121.

$[\alpha]^{D}_{20}$ = -92.0 (c = 0.1, DCM).

HPLC Separation (Chiralpak® IE-3, *n*-hexane/*i*-PrOH 80/20, 1.0 mL/min, 250 nm): *t*r(minor) = 8.0 min, *t*r(major) = 11.3 min, 95:5 er.

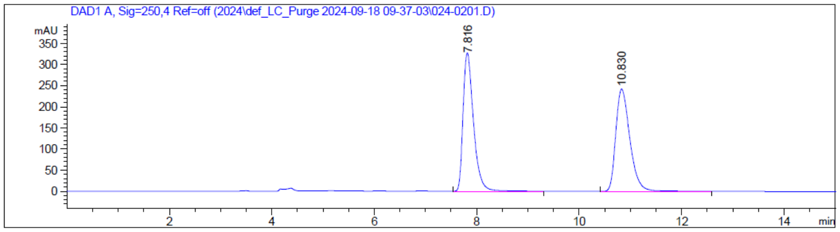

Peak #	RetTime [min]	Type	Width [min]	Area [mAU*s]	Height [mAU]	Area %
1	7.816	MM R	0.2357	4627.83447	327.30551	50.0168
2	10.830	MM R	0.3184	4624.71924	242.06720	49.9832

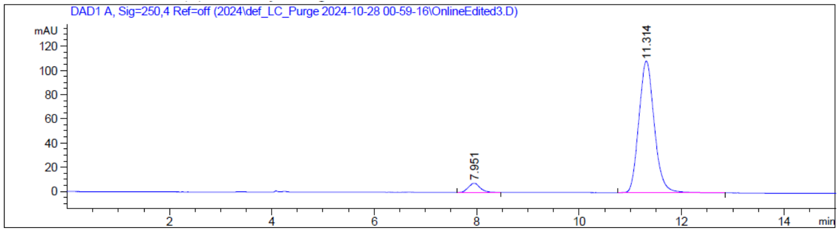

Peak #	RetTime [min]	Type	Width [min]	Area [mAU*s]	Height [mAU]	Area %
1	7.951	BB	0.2356	120.72453	7.62913	5.0206
2	11.314	MM R	0.3496	2283.86060	108.86620	94.9794

(*S*)-1-(2,3-diphenyl-1*H*-indol-1-yl)-8-(4-fluorophenyl)isoquinoline (15)

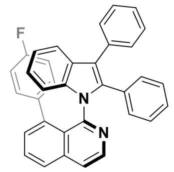

Prepared according to general procedure A at 0.1 mmol scale, column chromatography (n-hexane /EtOAc = 15:1) afforded the compound **15** as yellow foam (48.3mg, 98%), with an enantiomeric ratio of 94:6.

^{1}H-NMR (500 MHz, $CDCl_3$) δ 8.68 (d, J = 5.5 Hz, 1H), 7.80 (d, J = 5.5 Hz, 1H), 7.78 (dd, J = 8.2, 1.2 Hz, 1H), 7.50 (dd, J = 8.2, 7.1 Hz, 1H), 7.44 – 7.40 (m, 2H), 7.29 (t, J = 7.5 Hz, 2H), 7.27 – 7.23 (m, 1H), 7.23 – 7.19 (m, 1H), 7.15 – 7.11 (m, 2H), 7.10 – 7.02 (m, 3H), 6.93 – 6.86 (m, 2H), 6.86 – 6.82 (m, 2H), 6.68 – 6.64 (m, 2H), 6.54 – 6.44 (m, 1H), 6.05 – 5.93 (m, 1H).

^{13}C-NMR (126 MHz, $CDCl_3$) δ = 161.9 (d, $J_{C\text{-}F}$ =245.3, C_q), 149.5 (C_q), 141.5 (CH), 140.0 (C_q), 138.7 (C_q), 138.1 (C_q), 135.1 (C_q), 134.9 (C_q), 134.8 (d, $J_{C\text{-}F}$ =3.2, C_q), 132.0 (CH), 130.8 (C_q), 130.4 (CH), 130.3 (CH), 129.9 (CH), 128.9 (d, $J_{C\text{-}F}$ =8.2, CH), 128.4 (CH), 128.3 (C_q), 127.2 (CH), 126.9 (CH), 126.6 (CH), 126.0 (CH), 124.6 (C_q), 123.2 (CH), 121.7 (CH), 121.1 (CH), 119.2 (CH), 117.2 (C_q), 113.7 (d, $J_{C\text{-}F}$=21.8, CH), 112.5 (d, $J_{C\text{-}F}$ =21.5, CH), 111.2 (CH).

^{19}F NMR (377 MHz, $CDCl_3$) δ = -115.94.

HRMS (ESI): m/z [M+H]+ calculated for C35H24FN2+ 491.1918, found 491.1919.

$[\alpha]^{D}_{20}$ = -128.0 (c = 0.1, DCM).

HPLC Separation (Chiralpak® IA-3, *n*-hexane/*i*-PrOH 90/10, 1.0 mL/min, 250 nm): *t*r(minor) = 8.9 min, *t*r(major) = 11.6 min, 94:6 er.

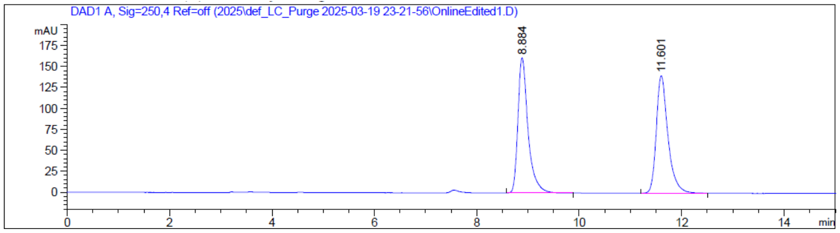

Peak #	RetTime [min]	Type	Width [min]	Area [mAU*s]	Height [mAU]	Area %
1	8.884	MM R	0.2292	2218.00049	161.26111	49.9985
2	11.601	MM R	0.2640	2218.13452	140.05663	50.0015

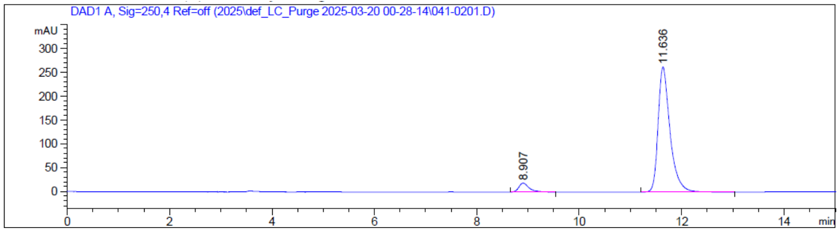

Peak #	RetTime [min]	Type	Width [min]	Area [mAU*s]	Height [mAU]	Area %
1	8.907	BB	0.2067	258.09314	18.62469	5.7375
2	11.636	MM R	0.2698	4240.25488	261.97583	94.2625

(*S*)-1-(2,3-diphenyl-1*H*-indol-1-yl)-8-methoxyisoquinoline (16)

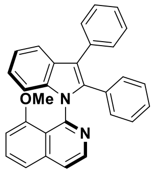

Prepared according to general procedure A at 0.1 mmol scale, column chromatography (*n*-hexane /Acetone = 15:1) afforded the compound **16** as white foam (30.3mg, 71%), with an enantiomeric ratio of 87:13.

^{1}H-NMR (500 MHz, $CDCl_3$) δ 8.52 (d, *J*=5.6, 1H), 7.84 – 7.80 (m, 1H), 7.64 (d, *J*=5.6, 1H), 7.49 – 7.43 (m, 3H), 7.37 – 7.32 (m, 3H), 7.26 – 7.22 (m, 1H), 7.20 – 7.13 (m, 2H), 7.07 – 7.01 (m, 3H), 6.99 – 6.92 (m, 3H), 6.63 (d, *J*=7.4, 1H), 3.42 (s, 3H).

^{13}C-NMR (126 MHz, $CDCl_3$) δ 156.0 (C_q), 148.7 (C_q), 142.0 (CH), 140.4 (C_q), 138.7 (C_q), 138.1 (C_q), 135.7 (C_q), 132.2 (C_q), 131.3 (CH), 130.6 (CH), 130.3 (CH), 128.4 (CH), 127.9 (C_q), 127.7 (CH), 127.0 (CH), 125.8 (CH), 122.6 (CH), 121.4 (CH), 120.6 (CH), 119.4 (CH), 118.8 (CH), 118.7 (C_q), 116.1 (C_q), 110.7 (CH), 106.9 (CH), 55.9 (CH_3).

HRMS (ESI): m/z $[M+H]^+$ calculated for $C_{30}H_{23}N_2O^+$ 427.1805, found 427.1805.

$[\alpha]^D_{20}$ = -60.0 (c = 0.1, DCM).

HPLC Separation (Chiralpak® IA-3, *n*-hexane/*i*-PrOH 70/30, 1.0 mL/min, 250 nm): *t*r(minor) = 6.2 min, *t*r(major) = 17.9 min, 87:13 er.

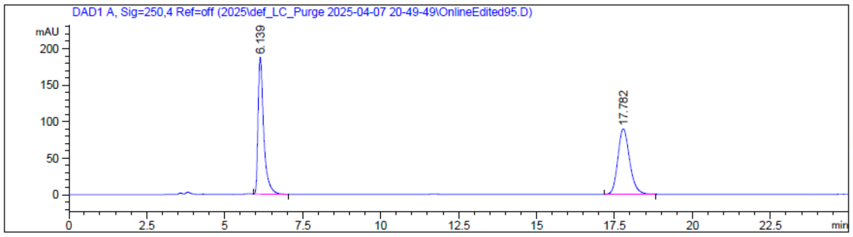

Peak #	RetTime [min]	Type	Width [min]	Area [mAU*s]	Height [mAU]	Area %
1	6.139	BB	0.1899	2354.90698	187.01756	50.0046
2	17.782	BB	0.3930	2354.47070	89.76939	49.9954

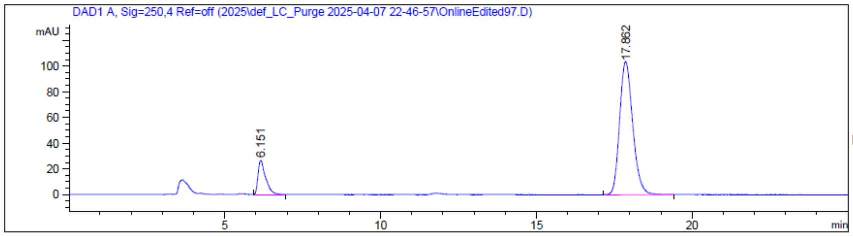

Peak #	RetTime [min]	Type	Width [min]	Area [mAU*s]	Height [mAU]	Area %
1	6.151	BB	0.2362	448.22491	26.65088	12.8450
2	17.862	MM R	0.4889	3041.26831	103.68578	87.1550

(*S*)-8-bromo-1-(2,3-diphenyl-1*H*-indol-1-yl)isoquinoline (17)

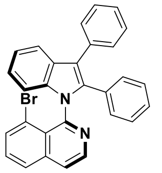

Prepared according to general procedure A at 0.1 mmol scale, column chromatography (*n*-hexane /EtOAc = 20:1) afforded the compound **17** as yellow foam (31.1mg, 65%), with an enantiomeric ratio of 86:14.

^{1}H-NMR (500 MHz, $CDCl_3$) δ = 8.50 (d, *J*=5.5, 1H), 7.85 – 7.79 (m, 3H), 7.72 (d, *J*=5.6, 1H), 7.46 – 7.42 (m, 3H), 7.35 – 7.31 (m, 2H), 7.26 – 7.16 (m, 3H), 7.13 – 7.09 (m, 2H), 7.02 – 6.95 (m, 3H), 6.92 – 6.87 (m, 1H).

^{13}C-NMR (126 MHz, $CDCl_3$) δ = 149.0 (C_q), 142.1 (CH), 140.5 (C_q), 139.1 (C_q), 137.6 (C_q), 135.4 (CH), 135.1 (C_q), 131.8 (C_q), 130.8 (CH), 130.4 (CH), 128.4 (CH), 128.3 (C_q), 127.8 (CH), 127.3 (CH), 127.2 (CH), 126.1 (CH), 125.2 (C_q), 123.1 (CH), 122.1 (CH), 121.1 (CH), 119.8 (CH), 118.9 (C_q), 118.1 (C_q), 110.7 (CH).

HRMS (ESI): m/z $[M+H]^+$ calculated for $C_{29}H_{20}BrN_2^+$ 475.0804, found 475.0804.

$[\alpha]^{D}_{20}$ = -47.0 (c = 0.1, DCM).

HPLC Separation (Chiralpak® AD-3, *n*-hexane/*i*-PrOH 70/30, 1.0 mL/min, 250 nm): *t*r(minor) = 14.0 min, *t*r(major) = 16.1 min, 86:14 er.

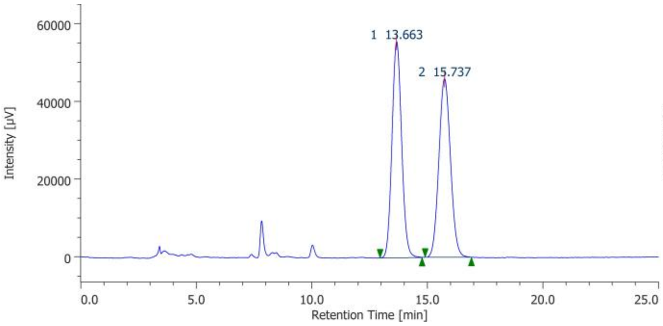

#	Peak Name	CH	tR [min]	Area [µV·sec]	Height [µV]	Area%	Height%	Quantity	NTP	Resolution	Symmetry Factor	Warning
1	Unknown	9	13.663	1609418	55640	50.080	54.773	N/A	5181	2.473	1.092	
2	Unknown	9	15.737	1604290	45942	49.920	45.227	N/A	4659	N/A	1.069	

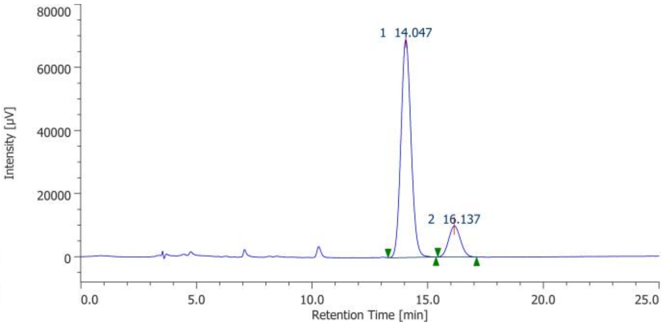

#	Peak Name	CH	tR [min]	Area [µV·sec]	Height [µV]	Area%	Height%	Quantity	NTP	Resolution	Symmetry Factor	Warning
1	Unknown	9	14.047	2094210	69020	85.601	87.518	N/A	4982	2.398	1.099	
2	Unknown	9	16.137	352261	9844	14.399	12.482	N/A	4597	N/A	1.088	

(*S*)-1-(2,3-di-*p*-tolyl-1*H*-indol-1-yl)-8-phenylisoquinoline (18)

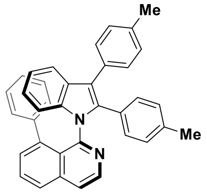

Prepared according to general procedure A at 0.1 mmol scale at 70°C, column chromatography (*n*-Hexane /EtOAc = 15:1) afforded the compound **18** as yellow foam (35.9 mg, 72%), with an enantiomeric ratio of 93:7.

^{1}H NMR (500 MHz, $CDCl_3$) δ 8.65 (d, J = 5.5 Hz, 1H), 7.80 – 7.77 (m, 2H), 7.52 (dd, J = 8.2, 7.1 Hz, 1H), 7.35 (d, J = 8.2 Hz, 1H), 7.32 (d, J = 7.9 Hz, 1H), 7.23 – 7.10 (m, 4H), 7.09 – 7.04 (m, 3H), 7.00 – 6.97 (m, 1H), 6.93 – 6.91 (m, 2H), 6.68 – 6.65 (m, 2H), 6.61 – 6.58 (m, 2H), 6.55 (d, J = 7.4 Hz, 1H), 6.38 – 6.27 (m, 1H), 2.35 (s, 3H), 2.11 (s, 3H).

^{13}C NMR (126 MHz, $CDCl_3$) δ 149.8 (C_q), 141.4 (CH), 140.0 (C_q), 139.9 (C_q), 138.9 (C_q), 138.0 (C_q), 136.3 (C_q), 135.3 (C_q), 135.0 (C_q), 132.4 (C_q), 132.0 (CH), 130.3 (CH), 130.2 (CH), 129.8 (CH), 128.9 (CH), 128.8 (CH), 128.5 (C_q), 128.1 (C_q), 127.9 (CH), 127.4 (CH), 126.8 (CH), 126.5 (CH), 126.4 (CH), 125.7 (CH), 124.7 (C_q), 122.7 (CH), 121.5 (CH), 120.6 (CH), 119.0 (CH), 116.7 (C_q), 111.2 (CH), 21.4 (CH_3), 21.2 (CH_3).

HRMS (ESI): m/z $[M+H]^+$ calculated for $C_{37}H_{29}N_2^+$ 501.2325, found 501.2328.

$[\alpha]^D{}_{20}$ = -105.0 (c = 0.1, DCM).

HPLC Separation (Chiralpak® IA-3, *n*-hexane/*i*-PrOH 70/30, 1.0 mL/min, 250 nm): *t*r(minor) = 6.9 min, *t*r(major) = 18.8 min, 93:7 er.

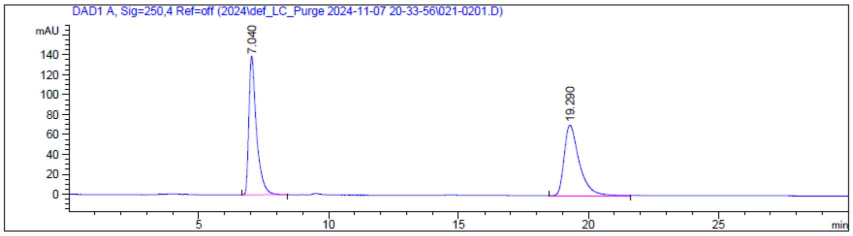

Peak #	RetTime [min]	Type	Width [min]	Area [mAU*s]	Height [mAU]	Area %
1	7.040	MM R	0.3402	2842.55933	139.25143	50.0458
2	19.290	MM R	0.6679	2837.35547	70.80358	49.9542

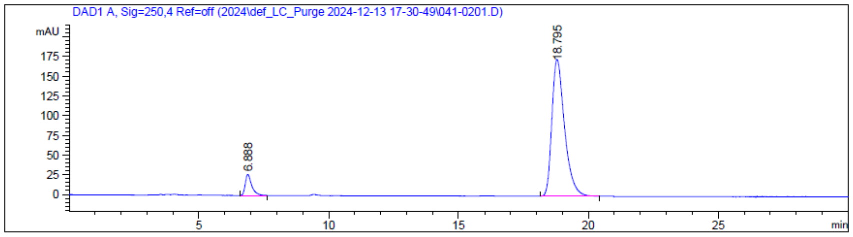

Peak #	RetTime [min]	Type	Width [min]	Area [mAU*s]	Height [mAU]	Area %
1	6.888	BB	0.2559	457.81595	26.46097	7.2178
2	18.795	BB	0.5063	5885.10107	173.25618	92.7822

(S)-1-(2,3-bis(4-methoxyphenyl)-1H-indol-1-yl)-8-phenylisoquinoline (19)

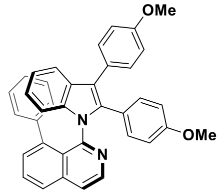

Prepared according to general procedure A at 0.1 mmol scale, column chromatography (DCM /EtOAc = 200:1) afforded the compound **19** as yellow foam (45.6 mg, 86%), with an enantiomeric ratio of 94:6.

^{1}H NMR (400 MHz, $CDCl_3$) δ 8.65 (d, J=5.5, 1H), 7.80 – 7.76 (m, 2H), 7.52 (dd, J=8.2, 7.1, 1H), 7.38 – 7.34 (m, 1H), 7.31 – 7.28 (m, 1H), 7.22 – 7.04 (m, 5H), 7.01 – 6.97 (m, 1H), 6.95 – 6.91 (m, 2H), 6.84 – 6.81 (m, 2H), 6.63 – 6.60 (m, 2H), 6.54 (d, J=7.6, 1H), 6.42 – 6.38 (m, 2H), 6.38 – 6.25 (m, 1H), 3.81 (s, 3H), 3.62 (s, 3H).

^{13}C NMR (101 MHz, $CDCl_3$) δ 158.2 (C_q), 157.8 (C_q), 149.8 (C_q), 141.4 (CH), 140.0 (C_q), 139.9 (C_q), 139.0 (C_q), 137.9 (C_q), 134.6 (C_q), 132.1 (CH), 131.7 (CH), 131.4 (CH), 129.9 (CH), 128.9 (CH), 128.6 (C_q), 127.7 (C_q), 127.4 (CH), 126.8 (CH), 126.5 (CH), 126.4 (CH), 125.8 (CH), 124.6 (C_q), 123.5 (C_q), 122.6 (CH), 121.5 (CH), 120.6 (CH), 118.9 (CH), 116.0 (C_q), 113.7 (CH), 112.7 (CH), 111.1 (CH), 55.3 (CH_3), 55.1 (CH_3).

HRMS (ESI): m/z $[M+H]^+$ calculated for $C_{37}H_{29}N_2O_2^+$ 533.2224, found 533.2227.

$[\alpha]^D{}_{20}$ = -115.0 (c = 0.1, DCM).

HPLC Separation (Chiralpak® IA-3, n-hexane/i-PrOH 50/50, 1.2 mL/min, 250 nm): tr(minor) = 7.8 min, tr(major) = 14.7 min, 94:6 er.

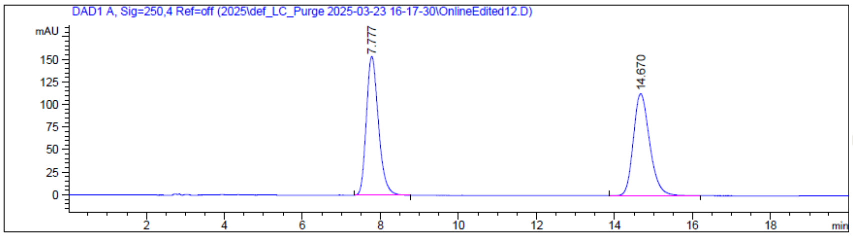

Peak #	RetTime [min]	Type	Width [min]	Area [mAU*s]	Height [mAU]	Area %
1	7.777	BB	0.3276	3298.89502	153.55611	50.0934
2	14.670	MM R	0.4848	3286.59961	112.97987	49.9066

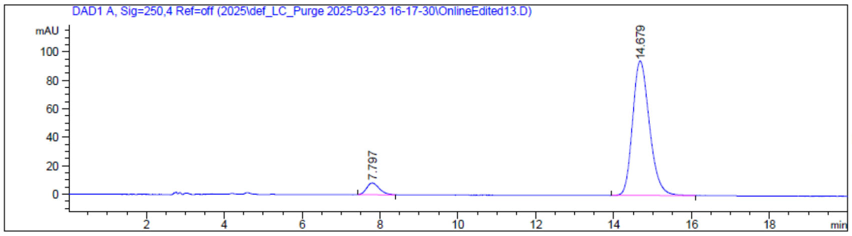

Peak #	RetTime [min]	Type	Width [min]	Area [mAU*s]	Height [mAU]	Area %
1	7.797	BB	0.2747	184.22375	8.21205	6.0791
2	14.679	MM R	0.5020	2846.23364	94.49638	93.9209

(*S*)-1-(2,3-bis(4-(trifluoromethyl)phenyl)-1*H*-indol-1-yl)-8-phenylisoquinoline (20)

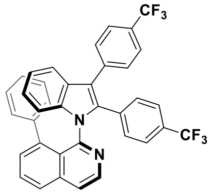

Prepared according to general procedure A at 0.1 mmol scale, column chromatography (*n*-hexane /EtOAc = 1:1) afforded the compound **20** as yellow foam (46.2 mg, 76%), with an enantiomeric ratio of 95:5.

HRMS (ESI): m/z $[M+H]^+$ calculated for $C_{37}H_{23}F_6N_2^+$ 609.1760, found 609.1763.

^{1}H NMR (400 MHz, $CDCl_3$) δ 8.68 (d, *J* = 5.6 Hz, 1H), 7.85 (d, *J* = 5.6 Hz, 1H), 7.82 (dd, *J* = 8.2, 1.1 Hz, 1H), 7.57 – 7.52 (m, 3H), 7.43 (dt, *J* = 8.2, 0.9 Hz, 1H), 7.33 – 7.28 (m, 2H), 7.21 – 7.00 (m, 9H), 6.79 (d, *J* = 8.1 Hz, 2H), 6.54 (d, *J* = 6.3 Hz, 1H), 6.35 (d, *J* = 7.7 Hz, 1H).

^{13}C NMR (101 MHz, $CDCl_3$) δ 148.9 (C_q), 141.5 (CH), 140.0 (C_q), 139.4 (C_q), 138.7 (C_q), 138.6 (C_q), 138.4 (C_q), 134.1 (C_q), 133.9 (C_q), 132.4 (CH), 130.5 (CH), 130.5 (CH), 130.2 (CH), 128.9 (CH), 128.9 (q, $J_{C\text{-}F}$ = 32.7 Hz, C_q), 128.3 (q, $J_{C\text{-}F}$ = 32.4 Hz, C_q), 127.7 (C_q), 127.1 (CH), 126.9 (CH), 126.7 (CH), 126.7 (CH), 126.1 (CH), 125.4 (q, $J_{C\text{-}F}$ = 3.8 Hz, CH), 124.5 (q, $J_{C\text{-}F}$ = 271.8 Hz, C_q), 124.4 (C_q), 124.3 (q, $J_{C\text{-}F}$ = 3.8 Hz, CH), 124.0 (q, $J_{C\text{-}F}$ = 272.2 Hz, C_q), 124.0 (CH), 122.1 (CH), 121.5 (CH), 119.0 (CH), 116.8 (C_q), 111.7 (CH).

^{19}F NMR (377 MHz, $CDCl_3$) δ -62.26, -62.74.

$[\alpha]^D_{20}$ = -157.0 (c = 0.1, DCM).

HPLC Separation (Chiralpak® IA-3, *n*-hexane/*i*-PrOH 75/25, 1.0 mL/min, 250 nm): *t*r(minor) = 8.0 min, *t*r(major) = 19.5 min, 95:5 er.

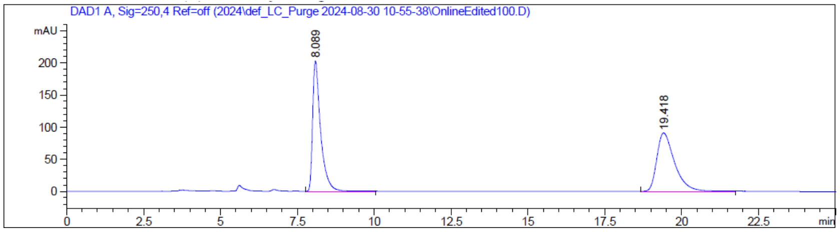

Peak #	RetTime [min]	Type	Width [min]	Area [mAU*s]	Height [mAU]	Area %
1	8.089	MM R	0.3013	3669.80884	202.99809	50.0961
2	19.418	MM R	0.6694	3655.72510	91.01431	49.9039

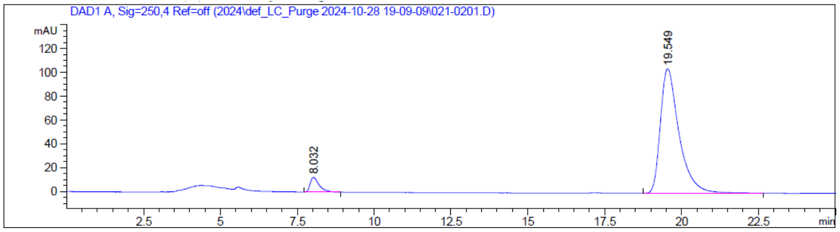

Peak #	RetTime [min]	Type	Width [min]	Area [mAU*s]	Height [mAU]	Area %
1	8.032	BB	0.2907	254.01472	12.41431	5.4388
2	19.549	MM R	0.7042	4416.37305	104.52129	94.5612

Dimethyl 4,4'-(1-(8-phenylisoquinolin-1-yl)-1*H*-indole-2,3-diyl)(*S*)-dibenzoate (21)

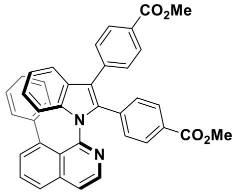

Prepared according to general procedure A at 0.1 mmol scale, column chromatography (*n*-hexane /EtOAc = 3:1) afforded the compound **21** as yellow foam (41.9 mg, 71%), with an enantiomeric ratio of 95:5.

^{1}H NMR (600 MHz, $CDCl_3$) δ 8.68 (d, *J* = 5.5 Hz, 1H), 7.95 – 7.92 (m, 2H), 7.83 (d, *J* = 5.5 Hz, 1H), 7.79 (dd, *J* = 8.3, 1.2 Hz, 1H), 7.54 – 7.50 (m, 3H), 7.46 (dt, *J* = 8.3, 0.9 Hz, 1H), 7.34 (dt, *J* = 8.0, 1.0 Hz, 1H), 7.29 (ddd, *J* = 8.2, 7.0, 1.1 Hz, 1H), 7.19 – 7.12 (m, 3H), 7.06 (d, *J* = 7.5 Hz, 1H), 7.05 – 7.00 (m, 3H), 6.71 – 6.67 (m, 2H), 6.52 (d, *J* = 7.6 Hz, 1H), 6.31 (t, *J* = 7.5 Hz, 1H), 3.92 (s, 3H), 3.80 (s, 3H).

^{13}C NMR (151 MHz, $CDCl_3$) δ 167.3 (C_q), 166.8 (C_q), 149.1 (C_q), 141.5 (CH), 140.0 (C_q), 140.0 (C_q), 139.5 (C_q), 138.7 (C_q), 138.5 (C_q), 135.2 (C_q), 134.4 (C_q), 132.3 (CH), 130.2 (CH), 130.2 (CH), 130.1 (CH), 129.7 (CH), 128.9 (CH), 128.6 (CH), 128.4 (C_q), 127.9 (C_q), 127.7 (C_q), 127.2 (CH), 126.9 (CH), 126.7 (CH), 126.6 (CH), 125.9 (CH), 124.4 (C_q), 123.9 (CH), 122.0 (CH), 121.4 (CH), 119.1 (CH), 117.3 (C_q), 111.7 (CH), 52.2 (CH_3), 52.1 (CH_3).

HRMS (ESI): m/z $[M+H]^+$ calculated for $C_{39}H_{29}N_2O_4^+$ 589.2122, found 589.2126.

$[\alpha]^{D}_{20}$ = -84.0 (c = 0.1, DCM).

HPLC Separation (Chiralpak® IB-3, *n*-hexane/*i*-PrOH 96/4, 1.0 mL/min, 250 nm): *t*r(major) = 14.5 min, *t*r(minor) = 18.6 min, 95:5 er.

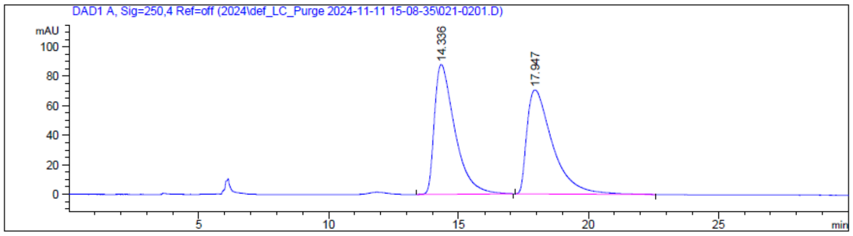

Peak #	RetTime [min]	Type	Width [min]	Area [mAU*s]	Height [mAU]	Area %
1	14.336	MM R	0.9112	4813.27930	88.04135	49.9776
2	17.947	MM R	1.1378	4817.58496	70.56935	50.0224

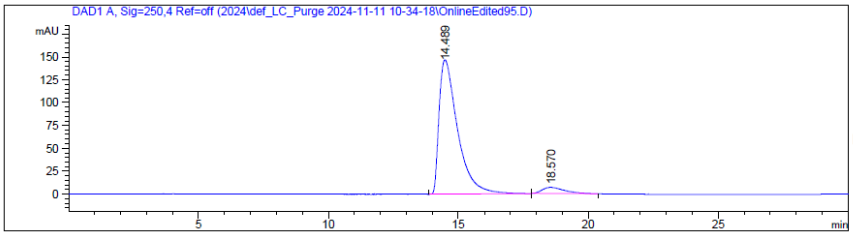

Peak #	RetTime [min]	Type	Width [min]	Area [mAU*s]	Height [mAU]	Area %
1	14.489	MM R	0.8236	7253.88232	146.79333	94.6189
2	18.570	MM R	1.0156	412.53470	6.77027	5.3811

(*S*)-1-(2,3-bis(4-fluorophenyl)-1*H*-indol-1-yl)-8-phenylisoquinoline (22)

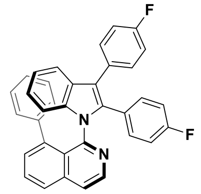

Prepared according to general procedure A at 0.1 mmol scale, column chromatography (*n*-hexane /EtOAc = 15:1) afforded the compound **22** as yellow foam (50.2 mg, 99%), with an enantiomeric ratio of 92:8.

^{1}H NMR (500 MHz, $CDCl_3$) δ 8.67 (d, *J* = 5.5 Hz, 1H), 7.82 – 7.79 (m, 2H), 7.54 (dd, *J* = 8.2, 7.1 Hz, 1H), 7.44 – 7.41 (m, 1H), 7.29 – 7.26 (m, 1H), 7.26 – 7.23 (m, 1H), 7.19 (dd, *J* = 7.2, 1.3 Hz, 2H), 7.13 – 7.06 (m, 2H), 7.02 – 6.90 (m, 5H), 6.65 – 6.61 (m, 2H), 6.60 – 6.51 (m, 3H), 6.37 – 6.26 (m, 1H).

^{13}C NMR (126 MHz, $CDCl_3$) δ 161.6 (d, $J_{C\text{-}F}$ = 247.5 Hz, C_q), 161.5 (d, $J_{C\text{-}F}$ = 245.1 Hz, C_q), 149.4 (C_q), 141.5 (CH), 140.0 (C_q), 139.7 (C_q), 138.8 (C_q), 138.0 (C_q), 134.0 (C_q), 132.2 (CH), 132.1 (d, $J_{C\text{-}F}$ = 7.9 Hz, CH), 131.8 (d, $J_{C\text{-}F}$ = 7.7 Hz, CH), 130.9 (d, $J_{C\text{-}F}$ = 3.3 Hz, C_q), 130.1 (CH), 129.0 (CH), 128.2 (C_q), 127.2 (CH), 126.8 (d, $J_{C\text{-}F}$ = 3.7 Hz, C_q), 126.7 (CH), 126.6 (CH), 126.5 (CH), 125.9 (CH), 124.4 (C_q), 123.2 (CH), 121.8 (CH), 121.0 (CH), 118.9 (CH), 116.1 (C_q), 115.3 (d, $J_{C\text{-}F}$ = 21.2 Hz, CH), 114.4 (d, $J_{C\text{-}F}$ = 21.7 Hz, CH), 111.4 (CH).

HRMS (ESI): m/z $[M+H]^+$ calculated for $C_{35}H_{23}F_2N_2^+$ 509.1824, found 509.1825.

$[\alpha]^D_{20}$ = -119.0 (c = 0.1, DCM).

HPLC Separation (Chiralpak® ID-3, *n*-hexane/*i*-PrOH 80/20, 1.0 mL/min, 273 nm): *t*r(minor) = 6.0 min, *t*r(major) = 11.3 min, 92:8 er.

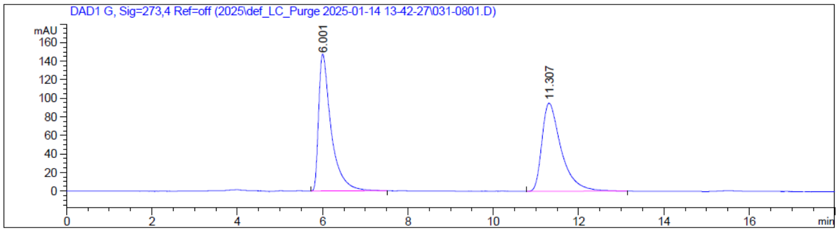

Peak #	RetTime [min]	Type	Width [min]	Area [mAU*s]	Height [mAU]	Area %
1	6.001	MM R	0.3385	3001.67480	147.80368	50.0113
2	11.307	MM R	0.5256	3000.32397	95.13356	49.9887

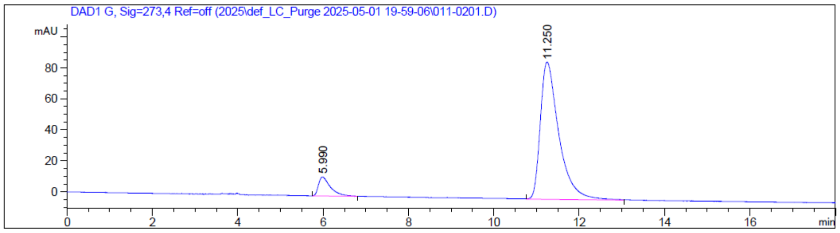

Peak #	RetTime [min]	Type	Width [min]	Area [mAU*s]	Height [mAU]	Area %
1	5.990	BB	0.2342	237.50211	12.19753	8.1919
2	11.250	MM R	0.5009	2661.74170	88.56258	91.8081

(*S*)-1-(2,3-bis(3-methoxyphenyl)-1*H*-indol-1-yl)-8-phenylisoquinoline (23)

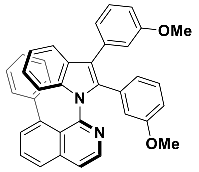

Prepared according to general procedure A at 0.1 mmol scale, column chromatography (*n*-hexane /EtOAc/DCM = 10:1:5) afforded the compound **23** as a yellow foam (37.1mg, 71%), with an enantiomeric ratio of 91.5:8.5.

^{1}H NMR (500 MHz, $CDCl_3$) δ 8.67 (d, J = 5.5 Hz, 1H), 7.81 (d, J = 5.6 Hz, 1H), 7.79 (dd, J = 8.3, 1.3 Hz, 1H), 7.53 (dd, J = 8.2, 7.1 Hz, 1H), 7.42 (d, J = 8.2 Hz, 1H), 7.36 (d, J = 7.9 Hz, 1H), 7.26 – 7.22 (m, 1H), 7.22 – 7.13 (m, 4H), 7.11 – 7.08 (m, 1H), 7.03 – 6.99 (m, 1H), 6.79 – 6.74 (m, 2H), 6.63 – 6.60 (m, 1H), 6.60 – 6.58 (m, 1H), 6.55 (d, J = 6.9 Hz, 1H), 6.48 – 6.45 (m, 1H), 6.36 – 6.27 (m, 2H), 6.25 – 6.22 (m, 1H), 3.70 (s, 3H), 3.36 (s, 3H).

^{13}C NMR (126 MHz, $CDCl_3$) δ 159.4 (C_q), 158.2 (C_q), 149.8 (C_q), 141.4 (CH), 140.0 (C_q), 138.9 (C_q), 138.1 (C_q), 136.6 (C_q), 134.9 (C_q), 132.2 (CH), 132.0 (C_q), 130.0 (CH), 129.1 (CH), 129.0 (CH), 128.2 (C_q), 128.1 (CH), 127.4 (CH), 126.8 (CH), 126.6 (CH), 126.4 (CH), 125.8 (CH), 124.7 (Cq), 123.1 (CH), 123.0 (CH), 123.0 (CH), 121.7 (CH), 120.9 (CH), 119.2 (CH), 117.2 (C_q), 115.7 (CH), 114.8 (CH), 114.0 (CH), 111.9 (CH), 111.3 (CH), 55.2 (CH_3), 54.9 (CH_3).

HRMS (ESI): m/z $[M+H]^+$ calculated for $C_{37}H_{29}N_2O_2^+$ 533.2224, found 533.2225.

$[\alpha]^D{}_{20}$ = -110.0 (c = 0.2, DCM).

HPLC Separation (Chiralpak® ID-3, *n*-hexane/*i*-PrOH 60/40, 1.0 mL/min, 250 nm): *t*r(minor) = 6.8 min, *t*r(major) = 17.5 min, 91.5:8.5 er.

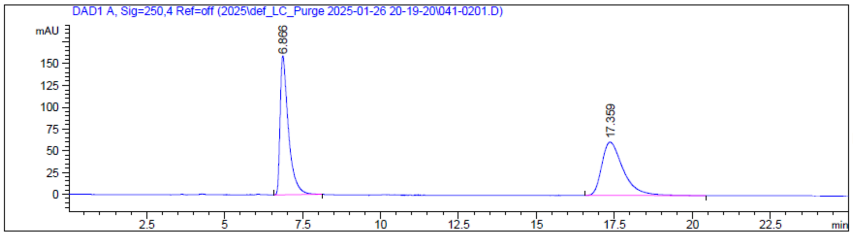

Peak #	RetTime [min]	Type	Width [min]	Area [mAU*s]	Height [mAU]	Area %
1	6.866	BB	0.2699	2949.26758	160.08243	49.9998
2	17.359	MM R	0.7993	2949.29224	61.49483	50.0002

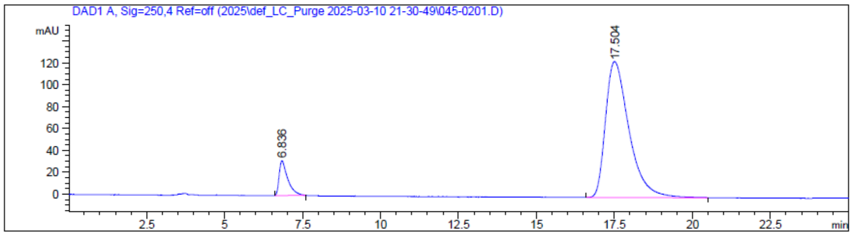

Peak #	RetTime [min]	Type	Width [min]	Area [mAU*s]	Height [mAU]	Area %
1	6.836	MM R	0.3189	606.16180	31.68089	8.5005
2	17.504	MM R	0.8737	6524.76416	124.46062	91.4995

(*S*)-1-(2,3-bis(3-chlorophenyl)-1*H*-indol-1-yl)-8-phenylisoquinoline (24)

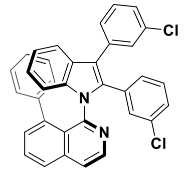

Prepared according to general procedure A at 0.1 mmol scale, column chromatography (n-hexane / EtOAc = 15:1) afforded the compound **24** as a sticky oil (41.8 mg, 77%), with an enantiomeric ratio of 92.5:7.5.

^{1}H-NMR (400 MHz, $CDCl_3$) δ 8.69 (d, *J* = 5.5 Hz, 1H), 7.84 (d, *J* = 5.7 Hz, 1H), 7.82 (dd, *J* = 8.3, 1.3 Hz, 1H), 7.54 (dd, *J* = 8.2, 7.1 Hz, 1H), 7.47 – 7.43 (m, 1H), 7.34 – 7.31 (m, 1H), 7.30 – 7.26 (m, 1H), 7.23 – 7.19 (m, 3H), 7.16 (dd, *J* = 8.0, 0.7 Hz, 1H), 7.15 – 7.11 (m, 1H), 7.08 (d, *J* = 6.8 Hz, 1H), 7.06 – 7.01 (m, 2H), 6.92 – 6.88 (m, 1H), 6.84 – 6.81 (m, 1H), 6.81 – 6.78 (m, 1H), 6.60 – 6.56 (m, 2H), 6.54 (d, *J* = 8.5 Hz, 1H), 6.39 – 6.26 (m, 1H).

^{13}C NMR (101 MHz, $CDCl_3$) δ 149.1 (C_q), 141.4 (CH), 140.0 (C_q), 139.6 (C_q), 138.7 (C_q), 138.1 (C_q), 136.6 (C_q), 134.0 (C_q), 133.7 (C_q), 133.0 (C_q), 132.3 (C_q), 132.2 (CH), 130.3 (CH), 130.1 (CH), 130.1 (CH), 129.6 (CH), 128.9 (CH), 128.5 (CH), 128.5 (CH), 127.7 (C_q), 127.2 (CH), 127.1 (CH), 126.9 (CH), 126.7 (CH), 126.5 (CH), 126.4 (CH), 125.9 (CH), 124.4 (C_q), 123.6 (CH), 121.9 (CH), 121.3 (CH), 119.0 (CH), 116.3 (C_q), 111.5 (CH).

HRMS (ESI): m/z $[M+H]^+$ calculated for $C_{35}H_{23}Cl_2N_2^+$ 541.1233, found 541.1232.

$[\alpha]^D{}_{20}$ = -138.0 (c = 0.1, DCM).

HPLC Separation (Chiralpak® IG-3, *n*-hexane/*i*-PrOH 95/5, 1.0 mL/min, 275 nm): *t*r(minor) = 12.5 min, *t*r(major) = 28.5 min, 92.5:7.5 e.r.

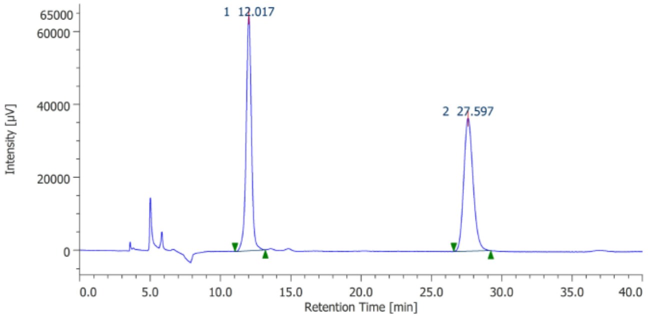

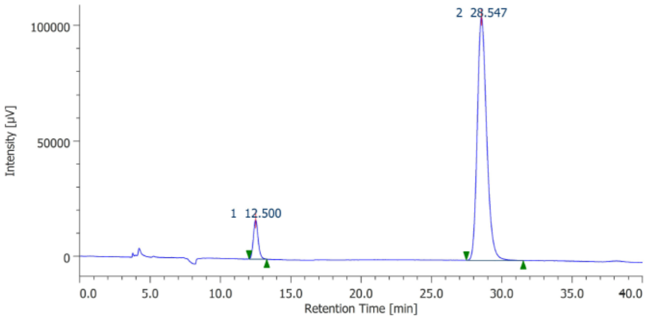

#	Peak Name	CH	tR [min]	Area [μV·sec]	Height [μV]	Area%	Height%	Quantity	NTP	Resolution	Symmetry Factor	Warning
1	Unknown	1	12.027	1680835	64507	49.965	63.789	N/A	5486	16.922	0.956	
2	Unknown	1	27.607	1683157	36619	50.035	36.211	N/A	8513	N/A	1.173	

#	Peak Name	CH	tR [min]	Area [μV·sec]	Height [μV]	Area%	Height%	Quantity	NTP	Resolution	Symmetry Factor	Warning
1	Unknown	9	12.500	399500	17033	7.541	13.913	N/A	6787	17.810	1.132	
2	Unknown	9	28.547	4898473	105388	92.459	86.087	N/A	9057	N/A	1.243	

(*S*)-1-(2,3-bis(2-fluorophenyl)-1*H*-indol-1-yl)-8-phenylisoquinoline (25)

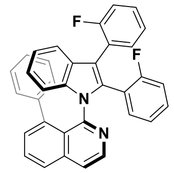

Prepared according to general procedure A at 0.1 mmol scale, column chromatography (*n*-hexane /EtOAc = 15:1) afforded the compound **25** as a white foam (50.6mg, 99%), with an enantiomeric ratio of 95:5.

^{1}H NMR (600 MHz, $CDCl_3$) δ 8.58 (d, *J* = 5.4 Hz, 1H), 7.78 (dd, *J* = 8.2, 1.2 Hz, 1H), 7.76 (d, *J* = 5.5 Hz, 1H), 7.54 (dd, *J* = 8.2, 7.1 Hz, 1H), 7.47 (d, *J* = 8.8 Hz, 1H), 7.29 – 7.17 (m, 6H), 7.11 – 7.08 (m, 1H), 7.04 (s, 2H), 7.00 – 6.91 (m, 3H), 6.86 – 6.81 (m, 1H), 6.76 – 6.63 (m, 2H), 6.61 – 6.57 (m, 1H), 6.37 (s, 1H).

^{13}C NMR (151 MHz, $CDCl_3$) δ 160.2 (d, $J_{C\text{-}F}$ = 248.3 Hz, C_q), 159.5 (d, $J_{C\text{-}F}$ = 250.8 Hz, C_q), 149.2 (C_q), 141.3 (CH), 140.1 (C_q), 140.0 (C_q), 138.7 (C_q), 137.9 (C_q), 132.6 (d, $J_{C\text{-}F}$ = 1.4 Hz, CH), 132.5 (d, $J_{C\text{-}F}$ = 3.8 Hz, CH), 131.8 (CH), 131.3 (d, $J_{C\text{-}F}$ = 1.5 Hz, C_q), 129.8 (CH), 129.5 (d, $J_{C\text{-}F}$ = 8.3 Hz, CH), 129.2 (CH), 128.3 (d, $J_{C\text{-}F}$ = 7.7 Hz, CH), 127.8 (C_q), 127.1 (CH), 126.7 (CH), 126.2 (CH), 125.8 (CH), 123.7 (d, $J_{C\text{-}F}$ = 3.5 Hz, CH), 123.5 (C_q), 123.2 (CH), 123.0 (d, $J_{C\text{-}F}$ = 3.4 Hz, CH), 122.7 (d, $J_{C\text{-}F}$ = 16.2 Hz, C_q), 121.2 (CH), 120.9 (CH), 119.5 (C_q), 119.4 (CH), 115.8 (d, $J_{C\text{-}F}$ = 22.1 Hz, CH), 115.3 (d, $J_{C\text{-}F}$ = 22.6 Hz, CH), 113.3 (C_q), 111.8 (CH).

^{19}F NMR (565 MHz, $CDCl_3$) δ -109.44, -112.76.

HRMS (ESI): m/z $[M+H]^+$ calculated for $C_{35}H_{23}F_2N_2^+$ 509.1824, found 509.1823.

$[\alpha]^{D}_{20}$ = -191.0 (c = 0.1, DCM).

HPLC Separation (Chiralpak® IF-3, *n*-hexane/*i*-PrOH 90/10, 1.0 mL/min, 250 nm): *t*r(minor) = 10.3 min, *t*r(major) = 19.5 min, 95:5 er.

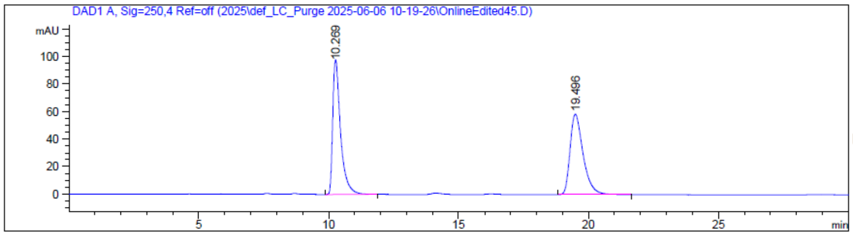

Peak #	RetTime [min]	Type	Width [min]	Area [mAU*s]	Height [mAU]	Area %
1	10.269	MM R	0.3484	2043.21814	97.74573	49.9913
2	19.496	MM R	0.5837	2043.92871	58.36431	50.0087

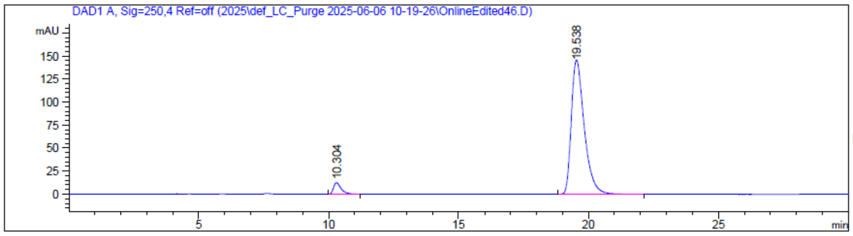

Peak #	RetTime [min]	Type	Width [min]	Area [mAU*s]	Height [mAU]	Area %
1	10.304	MM R	0.3405	254.30692	12.44902	4.8012
2	19.538	MM R	0.5760	5042.42041	145.90747	95.1988

(*S*)-1-(2,3-di(thiophen-2-yl)-1*H*-indol-1-yl)-8-phenylisoquinoline (26)

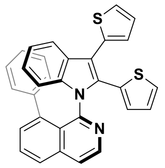

Prepared according to general procedure A at 0.1 mmol scale, column chromatography (*n*-hexane /EtOAc = 15:1) afforded the compound **26** as a yellow foam (35.9mg, 74%), with an enantiomeric ratio of 89:11.

^{1}H-NMR (500 MHz, $CDCl_3$) δ = 8.63 (d, *J* = 5.5 Hz, 1H), 7.90 (dd, *J* = 8.2, 1.3 Hz, 1H), 7.87 (d, *J* = 5.5 Hz, 1H), 7.66 (dd, *J* = 8.2, 7.1 Hz, 1H), 7.35 – 7.29 (m, 3H), 7.17 – 7.08 (m, 3H), 7.07 – 7.03 (m, 3H), 6.97 – 6.93 (m, 1H), 6.93 – 6.90 (m, 1H), 6.86 (dd, *J* = 3.5, 1.2 Hz, 1H), 6.70 (dd, *J* = 5.1, 3.7 Hz, 1H), 6.64 (dd, *J* = 3.7, 1.2 Hz, 1H), 6.58 (d, *J* = 7.6 Hz, 1H), 6.41 (t, *J* = 7.5 Hz, 1H).

^{13}C-NMR (126 MHz, $CDCl_3$) δ = 148.8 (C_q), 141.6 (CH), 140.3 (C_q), 139.9 (C_q), 139.0 (C_q), 137.9 (C_q), 136.0 (C_q), 132.4 (CH), 132.3 (C_q), 130.7 (C_q), 130.0 (CH), 129.4 (CH), 128.3 (C_q), 128.0 (CH), 127.8 (CH), 127.2 (CH), 127.0 (CH), 126.9 (CH), 126.9 (CH), 126.7 (CH), 126.5 (CH), 126.2 (CH), 125.7 (CH), 125.3 (CH), 125.0 (C_q), 123.2 (CH), 122.5 (CH), 121.0 (CH), 119.4 (CH), 111.3 (C_q), 111.1 (CH).

HRMS (ESI): m/z $[M+H]^+$ calculated for $C_{31}H_{21}N_2S_2^+$ 485.1141, found 485.1142.

$[\alpha]^{D}_{20}$ = 38.0 (c = 0.1, DCM).

HPLC Separation (Chiralpak® IA-3, *n*-hexane/*i*-PrOH 80/20, 1.0 mL/min, 250 nm): *t*r(minor) = 9.4 min, *t*r(major) = 15.7 min, 89:11 er.

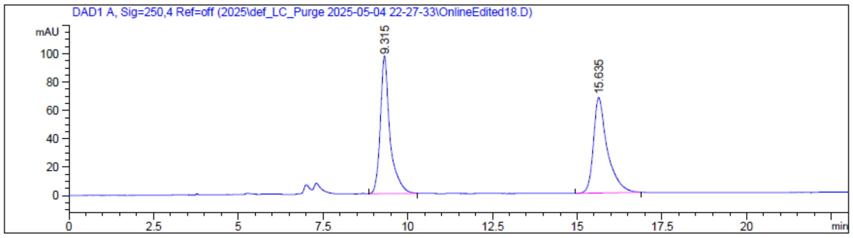

Peak #	RetTime [min]	Type	Width [min]	Area [mAU*s]	Height [mAU]	Area %
1	9.315	BB	0.2830	1923.14343	97.11241	49.9989
2	15.635	MM R	0.4762	1923.22437	67.30780	50.0011

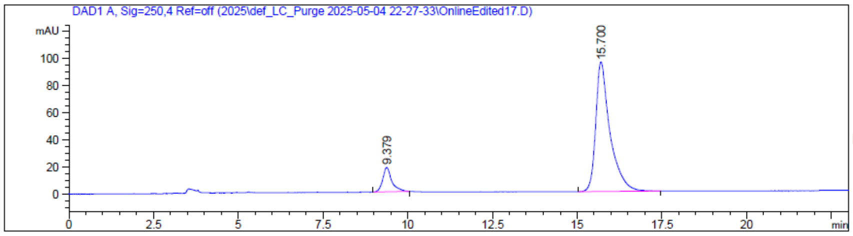

Peak #	RetTime [min]	Type	Width [min]	Area [mAU*s]	Height [mAU]	Area %
1	9.379	BB	0.2564	339.11862	18.11371	10.7907
2	15.700	MM R	0.4887	2803.58081	95.61838	89.2093

(*R*)-1-(2,3-dibutyl-1*H*-indol-1-yl)-8-phenylisoquinoline (27)

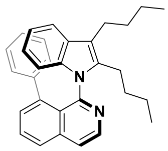

Prepared according to general procedure A at 0.1 mmol scale with 10% mol **Ru3**, column chromatography (*n*-hexane /EtOAc = 15:1) afforded the compound **27** as a yellow oil (31.0 mg, 72%), with an enantiomeric ratio of 97:3.

^{1}H-NMR (600 MHz, $CDCl_3$) δ 8.59 (d, *J* = 5.4 Hz, 1H), 7.94 (dd, *J* = 8.2, 1.3 Hz, 1H), 7.79 (d, *J* = 5.5 Hz, 1H), 7.74 (dd, *J* = 8.2, 7.1 Hz, 1H), 7.42 (dd, *J* = 7.1, 1.3 Hz, 1H), 7.22 – 7.18 (m, 1H), 7.10 (s, 1H), 7.08 – 7.03 (m, 1H), 7.02 – 6.96 (m, 2H), 6.96 – 6.93 (m, 1H), 6.86 – 6.81 (m, 1H), 6.51 (d, *J* = 7.4 Hz, 1H), 6.31 (s, 1H), 2.54 – 2.46 (m, 1H), 2.34 (t, *J* = 7.7 Hz, 2H), 2.16 – 2.08 (m, 1H), 1.48 – 1.31 (m, 4H), 1.12 – 0.97 (m, 4H), 0.95 (t, *J* = 7.2 Hz, 3H), 0.59 (t, *J* = 7.2 Hz, 3H).

^{13}C-NMR (151 MHz, $CDCl_3$) δ 149.7 (C_q), 141.8 (CH), 141.0 (C_q), 140.1 (C_q), 139.6 (C_q), 137.3 (C_q), 136.4 (C_q), 132.1 (CH), 129.9 (CH), 129.1 (C_q), 128.0 (CH), 127.0 (CH), 126.8 (CH), 126.7 (CH), 126.3 (CH), 125.9 (CH), 123.8 (C_q), 121.1 (CH), 120.9 (CH), 119.3 (CH), 117.8 (CH), 114.0 (C_q), 110.4 (CH), 32.9 (CH_2), 31.8 (CH_2), 25.4 (CH_2), 24.2 (CH_2), 23.2 (CH_2), 22.6 (CH_2), 14.2 (CH_3), 13.6 (CH_3).

HRMS (ESI): m/z $[M+H]^+$ calculated for $C_{31}H_{33}N_2^+$ 433.2638, found 433.2637.

$[\alpha]^D_{20}$ = 83.0 (c = 0.1, DCM).

HPLC Separation (Chiralpak® IE-3, *n*-hexane/*i*-PrOH 95/5, 1.0 mL/min, 250 nm): *t*r(minor) = 6.2 min, *t*r(major) = 7.8 min, 97:3 er.

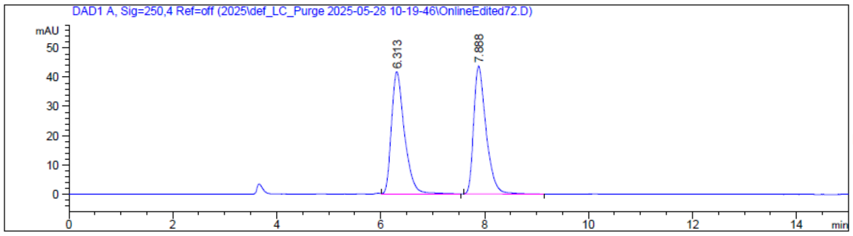

Peak #	RetTime [min]	Type	Width [min]	Area [mAU*s]	Height [mAU]	Area %
1	6.313	MM R	0.2895	724.96808	41.73835	49.9974
2	7.888	BB	0.2496	725.04224	43.68822	50.0026

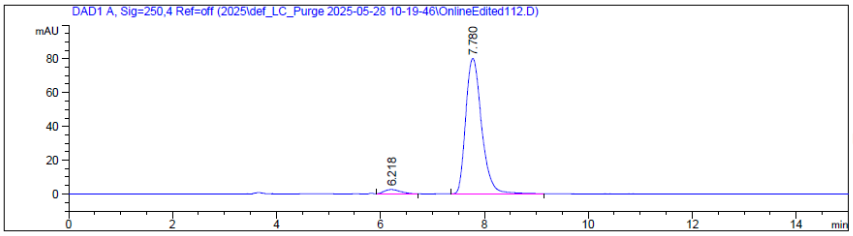

Peak #	RetTime [min]	Type	Width [min]	Area [mAU*s]	Height [mAU]	Area %
1	6.218	MM R	0.3535	52.94823	2.49627	3.0334
2	7.780	MM R	0.3523	1692.56189	80.06824	96.9666

(S)-1-(3-butyl-2-phenyl-1H-indol-1-yl)-8-phenylisoquinoline (29)

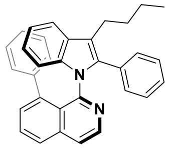

Prepared according to general procedure A at 0.1 mmol scale with 10% mol **Ru3**, column chromatography (n-hexane /EtOAc = 15:1) afforded the compound **29** as a yellow foam (34.4mg, 76%), with an enantiomeric ratio of 91.5:8.5 and a regional selectivity of 9.5:1.

^{1}H-NMR (500 MHz, $CDCl_3$) δ 8.61 (d, J = 5.5 Hz, 1H), 7.75 – 7.69 (m, 2H), 7.51 – 7.46 (m, 2H), 7.38 – 7.34 (m, 1H), 7.30 – 7.15 (m, 4H), 7.12 – 7.08 (m, 1H), 7.01 – 6.95 (m, 3H), 6.91 – 6.86 (m, 1H), 6.81 – 6.74 (m, 2H), 6.56 (s, 1H), 6.24 (s, 1H), 2.58 – 2.34 (m, 2H), 1.48 – 1.23 (m, 4H), 0.90 (t, J = 7.3 Hz, 3H).

^{13}C-NMR (126 MHz, $CDCl_3$) δ 150.0 (C_q), 141.3 (CH), 140.0 (C_q), 140.0 (C_q), 139.0 (C_q), 137.9 (C_q), 135.0 (C_q), 131.7 (CH), 131.7 (C_q), 129.7 (CH), 129.6 (CH), 129.2 (C_q), 127.2 (CH), 127.0 (CH), 126.6 (CH), 126.6 (CH), 126.1 (CH), 125.8 (CH), 124.1 (C_q), 122.6 (CH), 120.9 (CH), 119.9 (CH), 118.9 (CH), 116.4 (C_q), 111.3 (CH), 33.0 (CH_2), 24.4 (CH_2), 23.1 (CH_2), 14.1 (CH_3).

HRMS (ESI): m/z $[M+H]^+$ calculated for $C_{33}H_{29}N_2^+$ 453.2325, found 453.2326.

$[\alpha]^{D}_{20}$ = -55.0 (c = 0.1, DCM).

HPLC Separation (Chiralpak® IE-3, n-hexane/i-PrOH 90/10, 1.0 mL/min, 273 nm): tr(minor) = 9.6 min, tr(major) = 12.9 min, 91.5:8.5 er.

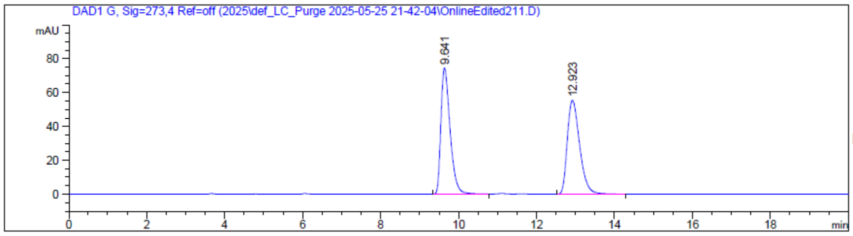

Peak #	RetTime [min]	Type	Width [min]	Area [mAU*s]	Height [mAU]	Area %
1	9.641	MM R	0.2735	1223.27429	74.53806	49.9920
2	12.923	MM R	0.3677	1223.66663	55.46682	50.0080

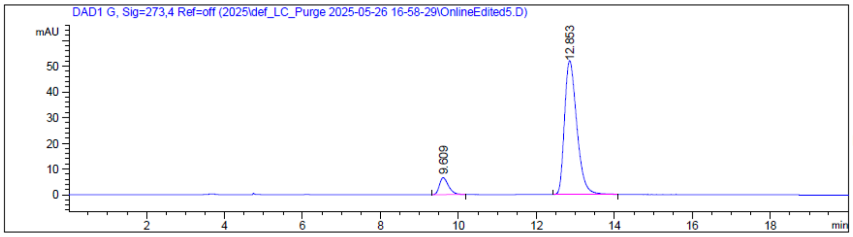

Peak #	RetTime [min]	Type	Width [min]	Area [mAU*s]	Height [mAU]	Area %
1	9.609	BB	0.2429	105.80835	6.56957	8.5136
2	12.853	MM R	0.3640	1137.00317	52.05901	91.4864

(*R*)-2,4,6,7,9-pentaphenyl-2,3-diazaspiro[4.4]nona-3,6,8-trien-1-one (31)

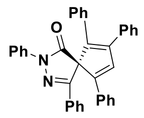

Prepared according to general procedure B at 0.1 mmol scale, column chromatography (*n*-hexane /EtOAc = 20:1) afforded the compound **31** as a yellow solid (36.5 mg, 71%), with an enantiomeric ratio of 96:4.

^{1}H-NMR (300 MHz, $CDCl_3$) δ 7.87 – 7.81 (m, 2H), 7.80 – 7.74 (m, 2H), 7.57 (s, 1H), 7.45 – 7.27 (m, 12H), 7.24 – 7.19 (m, 3H), 7.19 – 7.05 (m, 4H), 7.01 – 6.96 (m, 2H).

^{13}C-NMR (101 MHz, $CDCl_3$) δ 169.4 (C_q), 156.3 (C_q), 145.6 (C_q), 145.4 (C_q), 140.4 (C_q), 138.0 (C_q), 135.5 (CH), 134.5 (C_q), 133.6 (C_q), 133.3 (C_q), 131.2 (C_q), 130.9 (CH), 129.2 (CH), 129.0 (CH), 128.9 (CH), 128.7 (CH), 128.6 (CH), 128.5 (CH), 128.3 (CH), 128.2 (CH), 128.1 (CH), 125.9 (CH), 125.8 (CH), 125.3 (CH), 119.9 (CH), 76.7 (C_q).

$[\alpha]^{D}_{25}$ = 45.0 (c = 0.1, DCM).

HRMS (ESI): m/z $[M+Na]^+$ calculated for $C_{37}H_{26}N_2ONa^+$ 537.1943, found: 537.1937.

HPLC Separation (Chiralpak® IE-3, MeOH/*i*-PrOH 90/10, 0.5 mL/min, 250 nm): *t*r(minor) = 10.0 min, *t*r(major) = 13.8 min, 96:4 er.

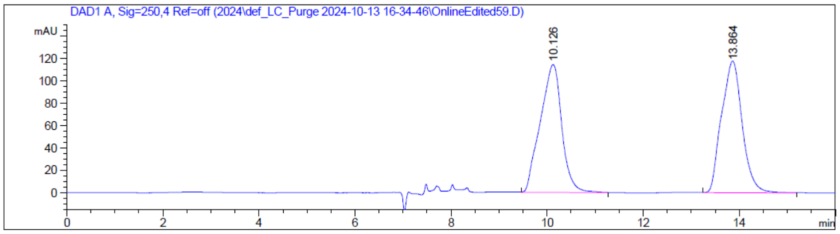

Peak #	RetTime [min]	Type	Width [min]	Area [mAU*s]	Height [mAU]	Area %
1	10.126	MM R	0.5188	3559.11108	114.33093	50.1860
2	13.864	MM R	0.4998	3532.72876	117.80238	49.8140

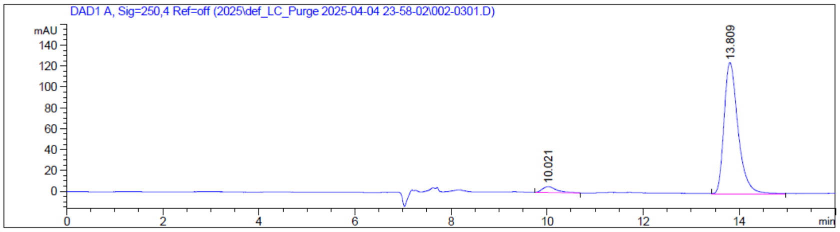

Peak #	RetTime [min]	Type	Width [min]	Area [mAU*s]	Height [mAU]	Area %
1	10.021	BB	0.2503	116.08153	5.70623	4.3599
2	13.809	MM R	0.3374	2546.39990	125.77181	95.6401

(*R*)-6,7-bis(4-fluorophenyl)-2,4,9-triphenyl-2,3-diazaspiro[4.4]nona-3,6,8-trien-1-one (32)

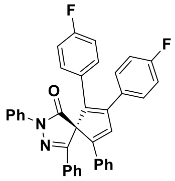

Prepared according to general procedure B at 0.1 mmol scale, column chromatography (*n*-hexane /EtOAc = 20:1) afforded the compound **32** as a yellow solid (29.0 mg, 53%), with an enantiomeric ratio of 93:7.

^{1}H NMR (300 MHz, $CDCl_3$) δ 7.87 – 7.80 (m, 2H), 7.78 – 7.71 (m, 2H), 7.51 (s, 1H), 7.46 – 7.39 (m, 2H), 7.38 – 7.26 (m, 8H), 7.25 – 7.18 (m, 3H), 7.02 – 6.89 (m, 4H), 6.85 – 6.77 (m, 2H).

^{13}C NMR (126 MHz, $CDCl_3$) δ 169.3 (C_q), 162.6 (d, $J_{C\text{-}F}$ = 248.4 Hz, C_q), 162.5 (d, $J_{C\text{-}F}$ = 241.4 Hz, C_q) 156.2 (C_q), 145.8 (C_q), 145.0 (C_q), 139.0 (C_q), 137.9 (C_q), 135.0 (CH), 133.2 (C_q), 131.2 (CH), 131.1 (C_q), 130.9 (CH), 130.8 (CH), 130.4 (C_q), 130.3 (CH), 130.2 (CH), 129.5 (d, $J_{C\text{-}F}$ = 3.4 Hz, C_q), 129.3 (CH), 129.1 (C_q), 129.0 (C_q), 128.5 (CH), 126.1 (CH), 125.9 (CH), 125.3 (CH), 119.8 (CH), 116.0 (d, $J_{C\text{-}F}$ = 21.5 Hz), 115.7 (d, $J_{C\text{-}F}$ = 21.6 Hz), 76.7 (C_q).

^{19}F NMR (471 MHz, $CDCl_3$) δ -112.65 – -112.74 (m), -112.75 – -112.84 (m).

$[\alpha]^{D}_{25}$ = 54.0 (c = 0.1, DCM).

HRMS (ESI): m/z $[M+Na]^+$ calculated for $C_{37}H_{24}F_2N_2ONa^+$ 573.1754, found: 573.1749.

HPLC Separation (Chiralpak® IE-3, MeOH/*i*-PrOH 90/10, 0.5 mL/min, 250 nm): *t*r(minor) = 9.4 min, *t*r(major) = 14.1 min, 93:7 er.

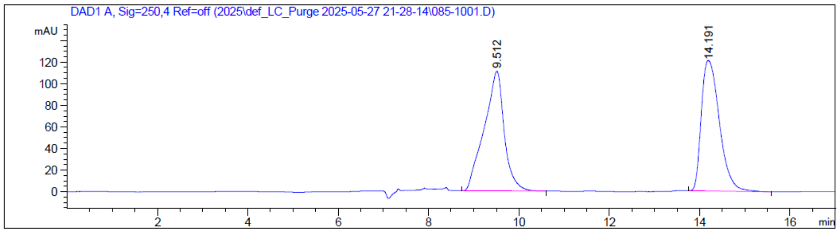

Peak #	RetTime [min]	Type	Width [min]	Area [mAU*s]	Height [mAU]	Area %
1	9.512	BB	0.4294	3420.47925	110.74009	50.4610
2	14.191	BB	0.4399	3357.98218	120.94901	49.5390

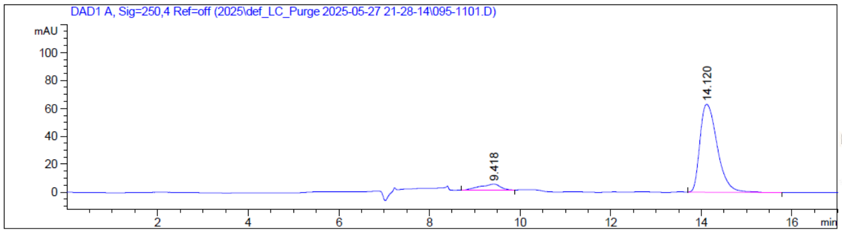

Peak #	RetTime [min]	Type	Width [min]	Area [mAU*s]	Height [mAU]	Area %
1	9.418	BB	0.4026	132.02348	4.23591	7.2998
2	14.120	BB	0.4179	1676.56592	62.91475	92.7002

(*R*)-6,7-bis(3-fluorophenyl)-2,4,9-triphenyl-2,3-diazaspiro[4.4]nona-3,6,8-trien-1-one (33)

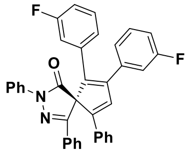

Prepared according to general procedure B at 0.1 mmol scale, column chromatography (*n*-hexane /EtOAc = 20:1) afforded the compound **33** as a yellow solid (32.5 mg, 59%), with an enantiomeric ratio of 95:5.

^{1}H NMR (300 MHz, $CDCl_3$) δ 7.89 – 7.80 (m, 2H), 7.78 – 7.70 (m, 2H), 7.50 (s, 1H), 7.47 – 7.39 (m, 2H), 7.39 – 7.29 (m, 5H), 7.28 – 7.19 (m, 5H), 7.18 – 6.94 (m, 4H), 6.92 – 6.83 (m, 1H), 6.81 – 6.73 (m, 1H), 6.70 – 6.62 (m, 1H).

^{13}C NMR (126 MHz, $CDCl_3$) δ 168.9 (C_q), 162.8 (d, $J_{C\text{-}F}$ = 246.3 Hz, C_q), 162.7 (d, $J_{C\text{-}F}$ = 247.2 Hz, C_q), 155.9 (C_q), 146.2 (C_q), 145.3 (C_q), 139.6 (C_q), 137.8 (C_q), 136.2 (d, $J_{C\text{-}F}$ = 7.9 Hz, C_q), 135.3 (d, $J_{C\text{-}F}$ = 8.1 Hz, C_q), 134.7 (CH), 133.0 (C_q), 131.1 (CH), 131.0 (C_q), 130.4 (d, $J_{C\text{-}F}$ = 8.6 Hz, CH), 130.2 (d, $J_{C\text{-}F}$ = 8.4 Hz, CH), 129.2 (CH), 129.1 (CH), 129.0 (CH), 128.5 (CH), 126.1 (CH), 125.8 (CH), 125.4 (CH), 124.6 (d, $J_{C\text{-}F}$ = 2.8 Hz, CH), 124.3 (d, $J_{C\text{-}F}$ = 2.8 Hz, CH), 119.9 (CH), 115.8 (d, $J_{C\text{-}F}$ = 22.0 Hz, CH), 115.5 (d, $J_{C\text{-}F}$ = 21.2 Hz, CH), 115.4 (CH), 115.3 (CH), 115.2 (CH), 76.7 (C_q).

^{19}F NMR (471 MHz, $CDCl_3$) δ -112.03 – -112.12 (m), -112.34 – -112.43 (m).

$[\alpha]^D_{25}$ = 65.0 (c = 0.1, DCM).

HRMS (ESI): m/z $[M+Na]^+$ calculated for $C_{37}H_{24}F_2N_2ONa^+$ 573.1754, found: 573.1749.

HPLC Separation (Chiralpak® IE-3, MeOH/*i*-PrOH 90/10, 0.5 mL/min, 250 nm): *t*r(minor) = 15.7 min, *t*r(major) = 19.9 min, 95:5 er.

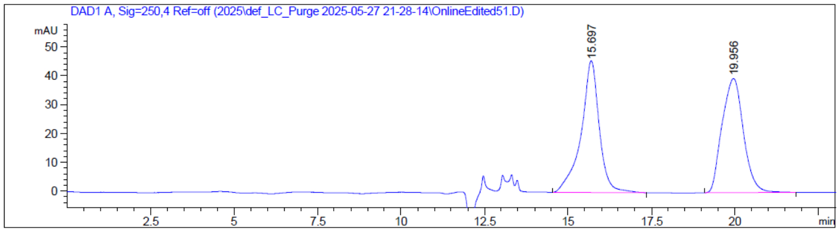

Peak #	RetTime [min]	Type	Width [min]	Area [mAU*s]	Height [mAU]	Area %
1	15.697	BB	0.5288	1738.12964	45.79364	49.6496
2	19.956	BB	0.6078	1762.66223	39.63877	50.3504

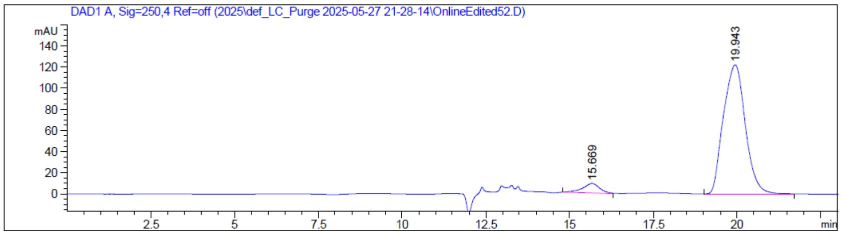

Peak #	RetTime [min]	Type	Width [min]	Area [mAU*s]	Height [mAU]	Area %
1	15.669	MM R	0.5778	309.47003	8.92729	5.2833
2	19.943	BB	0.6571	5548.04736	122.11420	94.7167

(*R*)-6,7-bis(3-chlorophenyl)-2,4,9-triphenyl-2,3-diazaspiro[4.4]nona-3,6,8-trien-1-one (34)

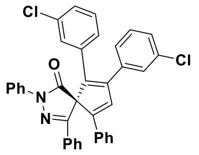

Prepared according to general procedure B at 0.1 mmol scale, column chromatography (*n*-hexane /EtOAc = 20:1) afforded the compound **34** as a yellow solid (32.0 mg, 55%), with an enantiomeric ratio of 94.5:5.5.

^{1}H NMR (300 MHz, $CDCl_3$) δ 7.84 (dd, *J* = 8.7, 1.2 Hz, 2H), 7.75 – 7.67 (m, 2H), 7.50 (s, 1H), 7.47 – 7.40 (m, 3H), 7.37 – 7.32 (m, 4H), 7.27 – 7.13 (m, 9H), 7.09 – 7.00 (m, 1H), 6.98 – 6.92 (m, 1H), 6.88 – 6.81 (m, 1H).

^{13}C NMR (126 MHz, $CDCl_3$) δ 168.9 (C_q), 156.0 (C_q), 146.4 (C_q), 145.3 (C_q), 139.5 (C_q), 137.8 (C_q), 135.9 (C_q), 135.0 (CH), 134.7 (C_q), 134.6 (CH), 134.5 (C_q), 133.0 (C_q), 131.2 (CH), 131.0 (C_q), 130.1 (CH), 130.0 (CH), 129.3 (CH), 129.2 (CH), 129.1 (CH), 128.8 (CH), 128.7 (CH), 128.6 (CH), 128.6 (CH), 128.4 (CH), 127.2 (CH), 126.8 (CH), 126.2 (CH), 125.9 (CH), 125.4 (CH), 120.0 (CH), 76.6 (C_q).

$[\alpha]^{D}{}_{25}$ = 38.0 (c = 0.1, DCM).

HRMS (ESI): m/z $[M+Na]^+$ calculated for $C_{37}H_{24}Cl_2N_2ONa^+$ 605.1163, found: 605.1158.

HPLC Separation (Chiralpak® IE-3, MeOH/*i*-PrOH 90/10, 0.5 mL/min, 250 nm): *t*r(minor) = 9.9 min, *t*r(major) = 12.6 min, 94.5:5.5 er.

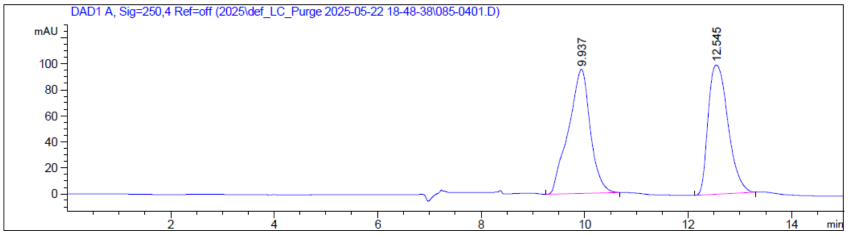

Peak #	RetTime [min]	Type	Width [min]	Area [mAU*s]	Height [mAU]	Area %
1	9.937	MM R	0.4875	2790.75317	95.41931	51.3719
2	12.545	MM R	0.4437	2641.69995	99.22250	48.6281

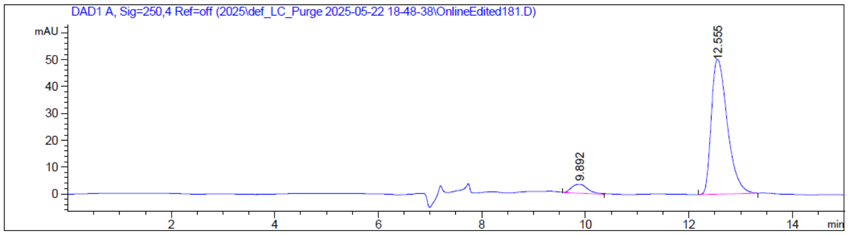

Peak #	RetTime [min]	Type	Width [min]	Area [mAU*s]	Height [mAU]	Area %
1	9.892	MM R	0.3283	62.30370	3.16330	5.4789
2	12.555	MP R	0.3566	1074.85632	50.24139	94.5211

(*R*)-2,4,9-triphenyl-6,7-bis(3-(trifluoromethyl)phenyl)-2,3-diazaspiro[4.4]nona-3,6,8-trien-1-one (35)

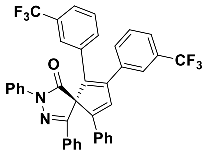

Prepared according to general procedure B at 0.1 mmol scale, column chromatography (*n*-hexane /EtOAc = 20:1) afforded the compound **35** as a yellow solid (32.3 mg, 50%), with an enantiomeric ratio of 95:5.

^{1}H NMR (300 MHz, $CDCl_3$) δ 7.88 – 7.78 (m, 2H), 7.76 – 7.69 (m, 2H), 7.62 (s, 1H), 7.59 – 7.52 (m, 2H), 7.51 – 7.32 (m, 10H), 7.27 – 7.19 (m, 6H), 7.12 (d, *J* = 7.5 Hz, 1H).

^{13}C NMR (126 MHz, $CDCl_3$) δ 168.7 (C_q), 155.9 (C_q), 146.8 (C_q), 145.5 (C_q), 139.6 (C_q), 137.7 (C_q), 134.6 (CH), 134.1 (CH), 133.9 (C_q), 132.8 (C_q), 132.2 (CH), 131.8 (CH), 131.5 (q, J_{C-F} = 32.6 Hz，C_q), 131.4 (q, J_{C-F} = 32.5 Hz，C_q), 131.3(CH), 131.2(CH), 130.8(CH), 129.4(CH), 129.3(CH), 129.2(CH), 129.1(CH), 129.0(CH), 128.8(CH), 126.2(CH), 125.8(CH), 125.7 (q, J_{C-F} = 3.8 Hz，CH), 125.4(CH), 125.2 (q, J_{C-F} = 3.7 Hz，CH), 123.8 (q, J_{C-F} = 272.4 Hz，C_q), 123.6 (q, J_{C-F} = 272.6 Hz，C_q), 119.8(CH), 76.7(C_q).

^{19}F NMR (471 MHz, $CDCl_3$) δ -62.97, -63.10.

$[\alpha]^D_{25}$ = 40.0 (c = 0.1, DCM).

HRMS (ESI): m/z $[M+Na]^+$ calculated for $C_{39}H_{24}F_6N_2ONa^+$ 673.1691, found: 673.1685.

HPLC Separation (Chiralpak® IE-3, *n*-Hexane/*i*-PrOH 60/40, 1.0 mL/min, 250 nm): *t*r(minor) = 5.7 min, *t*r(major) = 15.9 min, 95:5 er.

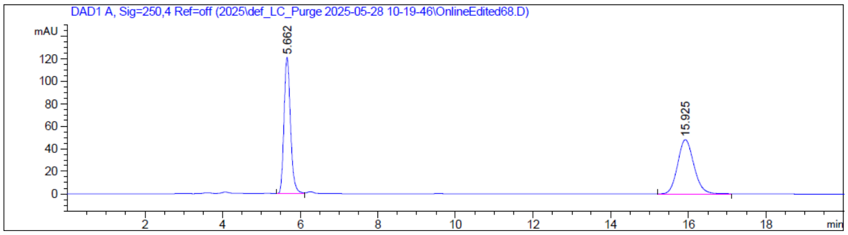

Peak #	RetTime [min]	Type	Width [min]	Area [mAU*s]	Height [mAU]	Area %
1	5.662	MF R	0.1932	1402.59912	121.00856	50.1455
2	15.925	BB	0.4397	1394.46045	48.03445	49.8545

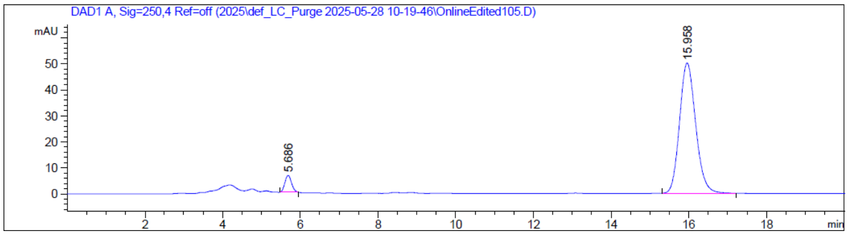

Peak #	RetTime [min]	Type	Width [min]	Area [mAU*s]	Height [mAU]	Area %
1	5.686	MM R	0.1973	76.40298	6.45558	5.0325
2	15.958	BB	0.4383	1441.77441	50.00976	94.9675

(*R*)-1,1'-((4-oxo-1,3,9-triphenyl-2,3-diazaspiro[4.4]nona-1,6,8-triene-6,7-diyl)bis(3,1-phenylene))bis(ethan-1-one) (36)

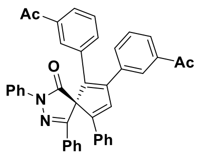

Prepared according to general procedure B at 0.1 mmol scale, column chromatography (*n*-hexane /EtOAc = 20:1) afforded the compound **36** as a yellow solid (43.6 mg, 73%), with an enantiomeric ratio of 93:7.

^{1}H NMR (300 MHz, $CDCl_3$) δ 8.04 – 8.00 (m, 1H), 7.91 – 7.83 (m, 3H), 7.80 – 7.72 (m, 3H), 7.62 (s, 1H), 7.55 – 7.47 (m, 2H), 7.45 – 7.32 (m, 8H), 7.32 – 7.18 (m, 5H), 7.17 – 7.11 (m, 1H), 2.46 (s, 3H), 2.19 (s, 3H).

^{13}C NMR (126 MHz, $CDCl_3$) δ 197.8 (C_q), 197.5 (C_q), 169.0 (C_q), 156.2 (C_q), 146.5 (C_q), 145.6 (C_q), 140.0 (C_q), 137.9 (C_q), 137.6 (C_q), 137.5 (C_q),134.6 (CH), 133.8 (C_q), 133.7 (CH), 133.1 (CH), 133.0 (C_q), 131.3 (CH), 131.0 (C_q), 129.4 (CH), 129.3 (CH), 129.2 (CH), 129.1 (CH), 129.0 (CH), 128.7 (CH), 128.3 (CH), 128.2 (CH), 127.9 (CH), 126.1 (CH), 125.9 (CH), 125.4 (CH), 119.7 (CH), 76.8 (C_q), 26.7 (CH_3), 26.4 (CH_3).

$[\alpha]^{D}_{25}$ = 28.0 (c = 0.1, DCM).

HRMS (ESI): m/z $[M+Na]^+$ calculated for $C_{41}H_{30}N_2O_3Na^+$ 621.2154, found: 621.2149.

HPLC Separation (Chiralpak® IE-3, MeOH/*i*-PrOH 90/10, 0.5 mL/min, 250 nm): *t*r(minor) = 15.2 min, *t*r(major) = 20.9 min, 93:7 er.

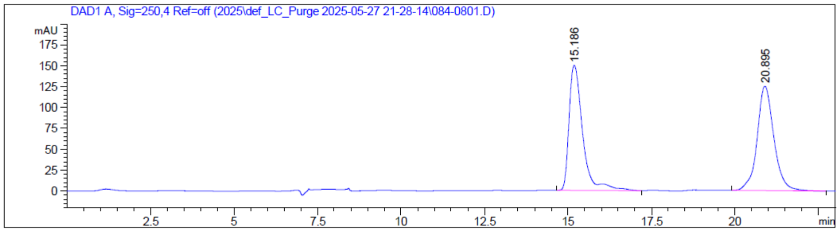

Peak #	RetTime [min]	Type	Width [min]	Area [mAU*s]	Height [mAU]	Area %
1	15.186	BV R	0.4165	4254.98438	149.92542	49.3471
2	20.895	BB	0.5259	4367.57520	125.24195	50.6529

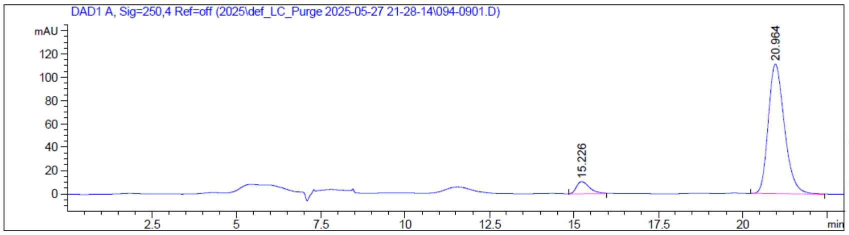

Peak #	RetTime [min]	Type	Width [min]	Area [mAU*s]	Height [mAU]	Area %
1	15.226	MM R	0.4466	280.03497	10.45165	7.0495
2	20.964	MM R	0.5543	3692.36157	111.01266	92.9505

(*R*)-4-(4-fluorophenyl)-2,6,7,9-tetraphenyl-2,3-diazaspiro[4.4]nona-3,6,8-trien-1-one (37)

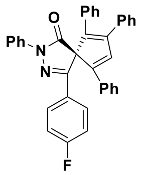

Prepared according to general procedure B at 0.1 mmol scale, column chromatography (*n*-hexane /EtOAc = 20:1) afforded the compound **37** as a yellow solid (36.1 mg, 68%), with an enantiomeric ratio of 91:9.

^{1}H NMR (300 MHz, $CDCl_3$) δ 7.87 – 7.70 (m, 4H), 7.57 (s, 1H), 7.47 – 7.33 (m, 5H), 7.32 – 7.18 (m, 8H), 7.17 – 7.07 (m, 3H), 7.04 – 6.93 (m, 4H).

^{13}C NMR (101 MHz, $CDCl_3$) δ 169.3 (C_q), 164.3 (d, J_{C-F} = 252.0 Hz, C_q), 155.4 (C_q), 145.8 (C_q), 145.3 (C_q), 140.3 (C_q), 137.9 (C_q), 135.6 (CH), 134.3 (C_q), 133.5 (C_q), 133.2 (C_q), 129.2 (CH), 129.1 (CH), 128.8 (CH), 128.7 (CH), 128.6 (CH), 128.5 (CH), 128.3 (CH), 128.2 (CH), 128.0 (d, J_{C-F} = 8.5 Hz, CH), 127.4 (d, J_{C-F} = 3.3 Hz, C_q), 126.0 (CH), 125.2 (CH), 119.9 (CH), 116.2 (d, J_{C-F} = 22.0 Hz, CH), 76.6 (C_q).

^{19}F NMR (377 MHz, $CDCl_3$) δ -108.44 – -108.55 (m).

$[\alpha]^{D}_{25}$ = 48.0 (c = 0.1, DCM).

HRMS (ESI): m/z $[M+Na]^+$ calculated for $C_{37}H_{25}FN_2ONa^+$ 555.1849, found: 555.1843.

HPLC Separation (Chiralpak® IE-3, MeOH/*i*-PrOH 90/10, 0.5 mL/min, 250 nm): *t*r(minor) = 9.9 min, *t*r(major) = 13.7 min, 91:9 er.

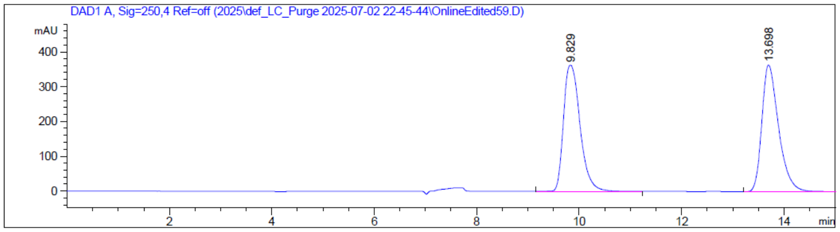

Peak #	RetTime [min]	Type	Width [min]	Area [mAU*s]	Height [mAU]	Area %
1	9.829	BB	0.3510	8158.12500	363.29230	49.6143
2	13.698	BBA	0.3472	8284.96387	364.40359	50.3857

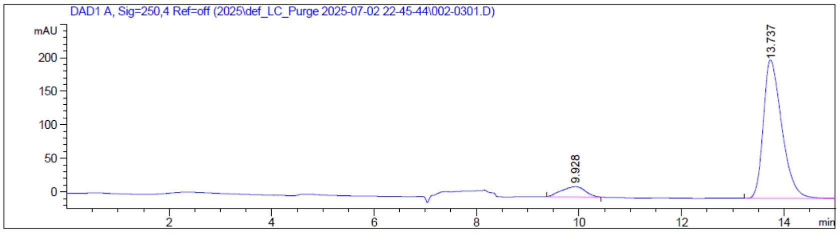

Peak #	RetTime [min]	Type	Width [min]	Area [mAU*s]	Height [mAU]	Area %
1	9.928	MM R	0.5382	491.80444	15.22938	8.6797
2	13.737	BBA	0.3854	5174.33545	206.43343	91.3203

(R)-2,6,7,9-tetraphenyl-4-(4-(trifluoromethyl)phenyl)-2,3-diazaspiro[4.4]nona-3,6,8-trien-1-one (38)

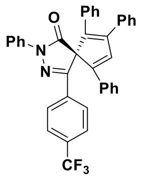

Prepared according to general procedure B at 0.1 mmol scale, column chromatography (*n*-hexane /EtOAc = 20:1) afforded the compound **38** as a yellow solid (29.7mg, 51%), with an enantiomeric ratio of 93:7.

^{1}H NMR (500 MHz, $CDCl_3$) δ 7.87 (d, J = 8.1 Hz, 2H), 7.84 – 7.80 (m, 2H), 7.60 (s, 1H), 7.57 (d, J = 8.3 Hz, 2H), 7.45 – 7.41 (m, 2H), 7.40 – 7.36 (m, 2H), 7.33 – 7.30 (m, 2H), 7.30 – 7.27 (m, 3H), 7.27 – 7.26 (m, 1H), 7.25 – 7.23 (m, 2H), 7.21 – 7.14 (m, 2H), 7.13 – 7.09 (m, 2H), 6.98 – 6.94 (m, 2H).

^{13}C NMR (126 MHz, $CDCl_3$) δ 169.3 (C_q), 155.0 (C_q), 146.1 (C_q), 145.1 (C_q), 140.0 (C_q), 137.8 (C_q), 135.8 (CH), 134.4 (C_q), 134.2 (C_q), 133.4 (Cq), 133.1 (Cq), 132.3 (q, $J_{C\text{-}F}$ = 32.6 Hz, C_q), 129.3 (CH), 129.2 (CH), 128.8 (CH), 128.8 (CH), 128.6 (CH), 128.5 (CH), 128.5 (CH), 128.4 (CH), 128.4 (CH), 126.3 (CH), 126.1 (CH), 126.0 (q, $J_{C\text{-}F}$ = 3.7 Hz, CH), 125.2 (CH), 123.9 (q, $J_{C\text{-}F}$ = 272.2 Hz, C_q), 120.0 (CH), 76.4 (C_q).

^{19}F NMR (471 MHz, $CDCl_3$) δ -63.0.

$[\alpha]^D_{20}$ =54 (c = 0.1, DCM).

HPLC Separation (Chiralpak® IB-3, MeOH/*i*-PrOH 70/30, 0.5 mL/min, 250 nm): *t*r(major) = 4.8 min, *t*r(minor) = 10.9 min, 93:7 er.

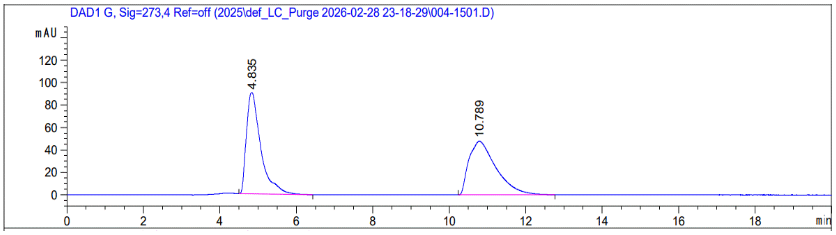

Peak #	RetTime [min]	Type	Width [min]	Area [mAU*s]	Height [mAU]	Area %
1	4.835	BB	0.3862	2350.94507	90.17467	50.0154
2	10.789	BB	0.6307	2349.49902	47.69521	49.9846

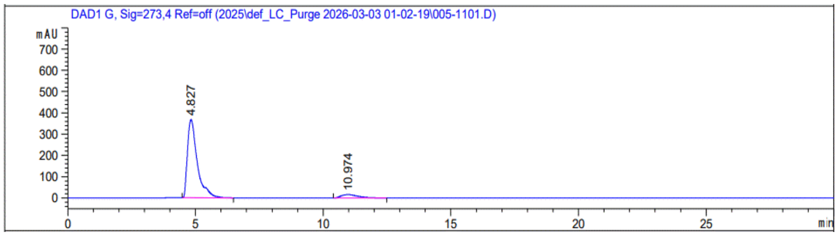

Peak #	RetTime [min]	Type	Width [min]	Area [mAU*s]	Height [mAU]	Area %
1	4.827	BB	0.3823	9773.29980	366.22775	93.1672
2	10.974	BB	0.5561	716.76825	15.98610	6.8328

(*R*)-4-(4-methoxyphenyl)-2,6,7,9-tetraphenyl-2,3-diazaspiro[4.4]nona-3,6,8-trien-1-one (39)

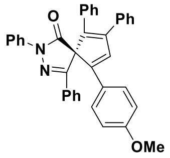

Prepared according to general procedure B at 0.1 mmol scale, column chromatography (*n*-hexane /EtOAc = 20:1) afforded the compound **39** as a yellow solid (38 mg, 70%), with an enantiomeric ratio of 90:10.

^{1}H NMR (400 MHz, $CDCl_3$) δ 7.9 (dd, *J* = 8.8, 1.1 Hz, 2H), 7.8 (dd, *J* = 8.3, 1.5 Hz, 2H), 7.4 (s, 1H), 7.4 – 7.4 (m, 4H), 7.4 – 7.2 (m, 8H), 7.2 – 7.2 (m, 1H), 7.2 – 7.1 (m, 3H), 7.0 – 7.0 (m, 2H), 6.8 – 6.7 (m, 2H), 3.7 (s, 3H).

^{13}C NMR (101 MHz, $CDCl_3$) δ 169.7 (C_q), 159.6 (C_q), 156.6 (C_q), 145.8 (C_q), 145.2 (C_q), 139.5 (C_q), 138.1 (C_q), 134.7 (C_q), 133.8 (C_q), 133.6 (CH), 131.3 (C_q), 130.9 (CH), 129.1 (CH), 129.0 (CH), 128.9 (CH), 128.7 (CH), 128.6 (CH), 128.5 (CH), 128.2 (CH), 128.1 (CH), 126.7 (CH), 126.3 (C_q), 126.0 (CH), 125.8 (CH), 119.9 (CH), 114.7 (CH), 76.7 (C_q), 55.3 (CH_3).

$[\alpha]^D{}_{25}$ = 52.0 (c = 0.1, DCM).

HRMS (ESI): m/z $[M+Na]^+$ calculated for $C_{38}H_{28}N_2O_2Na^+$ 567.2048, found: 567.2043.

HPLC Separation (Chiralpak® IE-3, MeOH/*i*-PrOH 90/10, 0.5 mL/min, 250 nm): *t*r(minor) = 12.4 min, *t*r(major) = 25.4 min, 90:10 er.

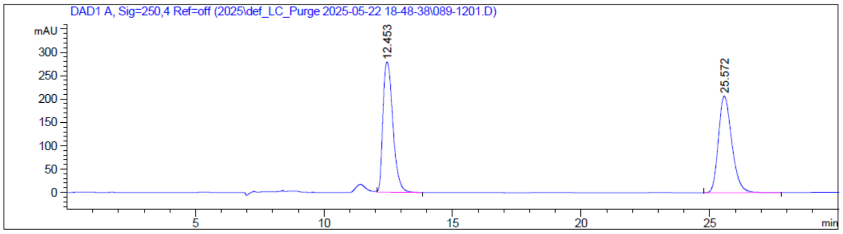

Peak #	RetTime [min]	Type	Width [min]	Area [mAU*s]	Height [mAU]	Area %
1	12.453	MM R	0.4494	7520.12549	278.87961	50.1372
2	25.572	BB	0.5568	7478.95508	206.39052	49.8628

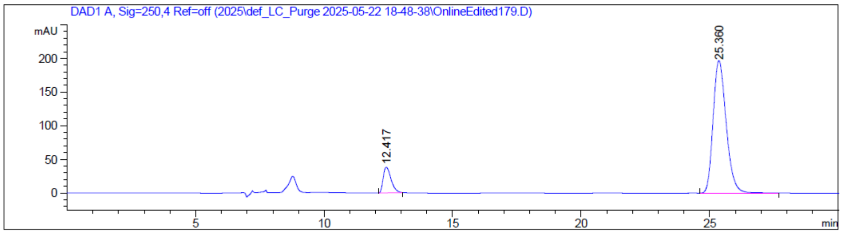

Peak #	RetTime [min]	Type	Width [min]	Area [mAU*s]	Height [mAU]	Area %
1	12.417	MM R	0.3536	808.97748	38.12715	10.3933
2	25.360	BB	0.5511	6974.68701	196.96906	89.6067

(*R*)-9-(benzo[d][1,3]dioxol-5-yl)-2,4,6,7-tetraphenyl-2,3-diazaspiro[4.4]nona-3,6,8-trien-1-one (40)

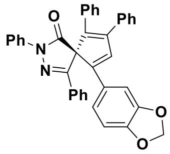

Prepared according to general procedure B at 0.1 mmol scale, column chromatography (*n*-hexane /EtOAc = 20:1) afforded the compound **40** as a yellow solid (41.3 mg, 74%), with an enantiomeric ratio of 88:12.

^{1}H NMR (300 MHz, $CDCl_3$) δ 7.85 – 7.72 (m, 4H), 7.45 – 7.30 (m, 9H), 7.24 (d, *J* = 8.0 Hz, 3H), 7.16 – 7.04 (m, 3H), 6.96 (d, *J* = 6.7 Hz, 2H), 6.91 (d, *J* = 1.7 Hz, 1H), 6.73 (dd, *J* = 8.1, 1.8 Hz, 1H), 6.64 (d, *J* = 8.2 Hz, 1H), 5.89 (s, 2H).

^{13}C NMR (126 MHz, $CDCl_3$) δ 169.5 (C_q), 156.4 (C_q), 148.4 (C_q), 147.8 (C_q), 145.6 (C_q), 145.0 (C_q), 139.7 (C_q), 137.9 (C_q), 134.5 (C_q), 134.2 (CH), 133.7 (C_q), 131.2 (C_q), 130.9 (CH), 129.1 (CH), 129.0 (CH), 128.6 (CH), 128.5 (CH), 128.4 (CH), 128.1 (CH), 127.8 (C_q), 126.0 (CH), 125.9 (CH), 119.9 (CH), 119.2 (CH), 108.9 (CH), 105.8 (CH), 101.3 (C_q), 76.7 (C_q), 29.8 (CH_2).

$[\alpha]^{D}_{25}$ = 40 (c = 0.1, DCM).

HRMS (ESI): m/z $[M+Na]^+$ calculated for $C_{38}H_{26}N_2O_3Na^+$ 581.1841, found: 581.1836.

HPLC Separation (Chiralpak® IE-3, MeOH/*i*-PrOH 90/10, 0.5 mL/min, 250 nm): *t*r(minor) = 12.5 min, *t*r(major) = 26.4 min, 88:12 er.

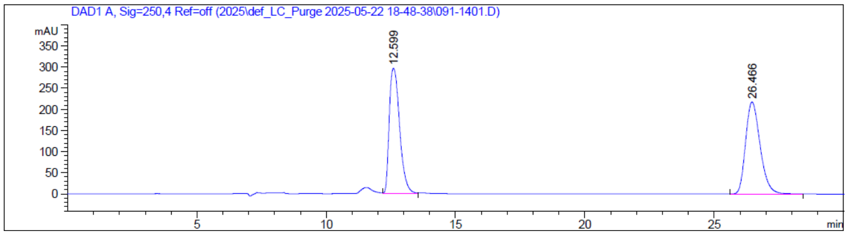

Peak #	RetTime [min]	Type	Width [min]	Area [mAU*s]	Height [mAU]	Area %
1	12.599	MM R	0.4615	8192.78320	295.87152	49.1156
2	26.466	BB	0.6041	8487.83398	217.59514	50.8844

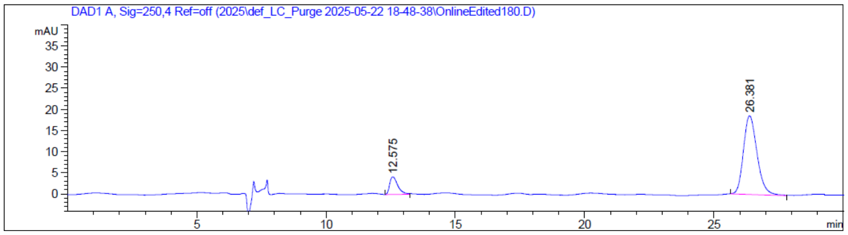

Peak #	RetTime [min]	Type	Width [min]	Area [mAU*s]	Height [mAU]	Area %
1	12.575	MM R	0.3679	92.74715	4.20155	11.9725
2	26.381	MM R	0.6096	681.91840	18.64492	88.0275

(*R*)-4-methyl-2,6,7,9-tetraphenyl-2,3-diazaspiro[4.4]nona-3,6,8-trien-1-one (41)

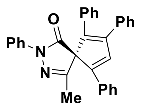

Prepared according to general procedure B at 0.1 mmol scale, column chromatography (*n*-hexane /EtOAc = 20:1) afforded the compound **41** as a yellow solid (24.7 mg, 55%), with an enantiomeric ratio of 91:9.

^{1}H NMR (300 MHz, $CDCl_3$) δ 7.81 – 7.72 (m, 2H), 7.45 (s, 1H), 7.43 – 7.26 (m, 12H), 7.20 (tt, *J* = 5.2, 2.7 Hz, 4H), 7.13 (td, *J* = 5.3, 3.3 Hz, 2H), 1.94 (s, 3H).

^{13}C NMR (126 MHz, $CDCl_3$) δ 169.8 (C_q), 159.7 (C_q), 146.4 (C_q), 142.6 (C_q), 138.0 (C_q), 137.7 (C_q), 136.1 (CH), 134.4 (C_q), 133.8 (C_q), 133.2 (C_q), 129.3 (CH), 129.1 (CH), 128.9 (CH), 128.7 (CH), 128.6 (CH), 128.5 (CH), 128.3 (CH), 128.2 (CH), 128.1 (CH), 125.7 (CH), 125.1 (CH), 119.8 (CH), 78.1 (C_q), 14.3 (CH_3).

$[\alpha]^D{}_{25}$ = -114.0 (c = 0.05, DCM).

HRMS (ESI): m/z $[M+Na]^+$ calculated for $C_{32}H_{24}N_2ONa^+$ 475.1786, found: 475.1781.

HPLC Separation (Chiralpak® IE-3, MeOH/*i*-PrOH 90/10, 0.5 mL/min, 250 nm): *t*r(minor) = 10.3 min, *t*r(major) = 11.9 min, 91:9 er.

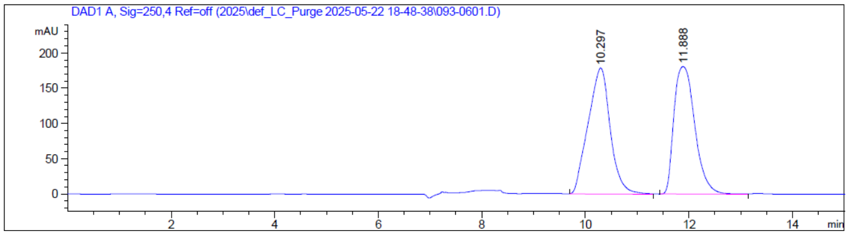

Peak #	RetTime [min]	Type	Width [min]	Area [mAU*s]	Height [mAU]	Area %
1	10.297	MM R	0.4959	5313.88867	178.58258	51.1660
2	11.888	MM R	0.4672	5071.68945	180.93259	48.8340

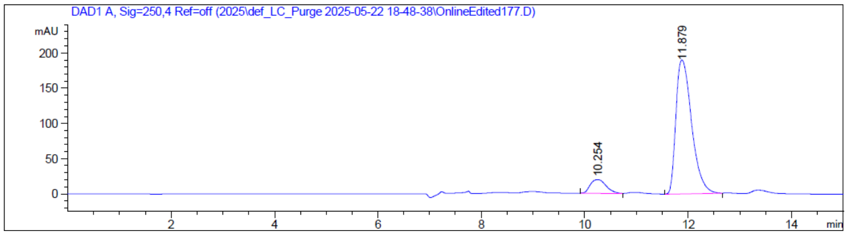

Peak #	RetTime [min]	Type	Width [min]	Area [mAU*s]	Height [mAU]	Area %
1	10.254	MM R	0.3502	405.87534	19.31717	9.1986
2	11.879	BB	0.3299	4006.48242	190.07661	90.8014

(*S*)-2,4,9-triphenyl-6,7-dipropyl-2,3-diazaspiro[4.4]nona-3,6,8-trien-1-one (42)

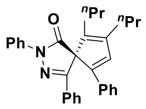

Prepared according to general procedure B at 0.1 mmol scale, column chromatography (*n*-hexane /EtOAc = 20:1) afforded the compound **42** as a yellow solid (24.5 mg, 55%), with an enantiomeric ratio of 74:26.

^{1}H NMR (500 MHz, $CDCl_3$) δ 8.07 – 8.01 (m, 2H), 7.72 – 7.65 (m, 2H), 7.52 – 7.43 (m, 2H), 7.36 – 7.31 (m, 1H), 7.30 – 7.27 (m, 2H), 7.24 (t, *J* = 1.1 Hz, 1H), 7.23 – 7.19 (m, 2H), 7.19 – 7.14 (m, 2H), 7.13 (s, 1H), 7.11 – 7.07 (m, 1H), 2.43 (t, *J* = 7.4 Hz, 2H), 2.14 (ddd, *J* = 14.6, 9.4, 7.0 Hz, 1H), 2.06 (ddd, *J* = 14.7, 9.3, 6.3 Hz, 1H), 1.77 – 1.61 (m, 2H), 1.36 – 1.21 (m, 2H), 1.01 (t, *J* = 7.4 Hz, 3H), 0.77 (t, *J* = 7.3 Hz, 3H).

^{13}C NMR (126 MHz, $CDCl_3$) δ 170.4 (C_q), 157.9 (C_q), 146.2 (C_q), 143.7 (C_q), 141.7 (C_q), 138.4 (C_q), 135.7 (CH), 133.7 (C_q), 131.1 (C_q), 130.8 (CH), 129.1 (CH), 129.1 (CH), 128.8 (CH), 127.5 (CH), 126.0 (CH), 125.6 (CH), 124.9 (CH), 119.4 (CH), 75.0 (C_q), 29.7 (CH_2), 28.6 (CH_2), 22.8 (CH_2), 22.4 (CH_2), 14.5 (CH_3), 14.1 (CH_3).

$[\alpha]^{D}_{20}$ = 69 (c = 0.1, DCM).

HRMS (ESI): m/z $[M+Na]^+$ $C_{31}H_{30}N_2ONa^+$calculated for 469.2256, found: 469.2250.

HPLC Separation (Chiralpak® IE-3, MeOH /*i*-PrOH 90/10, 0.5 mL/min, 273 nm): t_r(minor) = 9.3 min, t_r(major) = 15.9 min, 74:26 er.

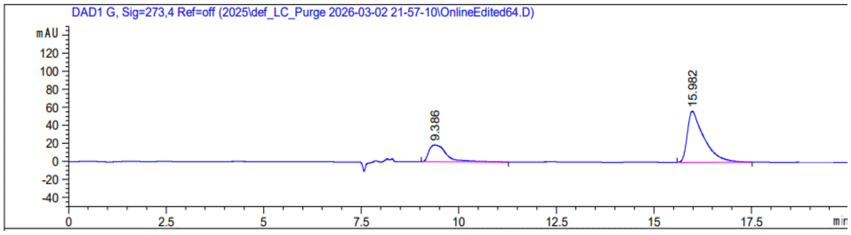

Peak #	RetTime [min]	Type	Width [min]	Area [mAU*s]	Height [mAU]	Area %
1	9.386	BB	0.4352	584.60577	18.57178	25.7874
2	15.982	BB	0.4082	1682.41321	56.86533	74.2126

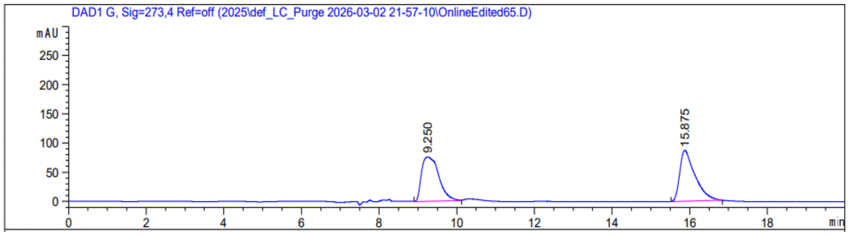

Peak #	RetTime [min]	Type	Width [min]	Area [mAU*s]	Height [mAU]	Area %
1	9.250	MM R	0.5153	2355.03174	76.17107	49.7624
2	15.875	MM R	0.4544	2377.52515	87.20679	50.2376

(*R*)-6-(tert-butyl)-7-butyl-2,4,9-triphenyl-2,3-diazaspiro[4.4]nona-3,6,8-trien-1-one (43)

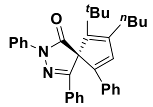

Prepared according to general procedure B at 0.1 mmol scale, column chromatography (*n*-hexane /EtOAc = 20:1) afforded the compound **43** as a yellow solid (29.7 mg, 51%), with an enantiomeric ratio of 85:15 and a regional selectivity over 20:1.

^{1}H NMR (500 MHz, $CDCl_3$) δ 7.91 (d, *J* = 7.6 Hz, 2H), 7.73 (d, *J* = 7.0 Hz, 2H), 7.46 – 7.41 (m, 2H), 7.39 – 7.35 (m, 1H), 7.34 – 7.30 (m, 2H), 7.25 – 7.21 (m, 1H), 7.18 – 7.09 (m, 5H), 6.84 (s, 1H), 2.71 (ddd, *J* = 14.0, 10.1, 6.0 Hz, 1H), 2.62 (ddd, *J* = 14.0, 10.1, 5.6 Hz, 1H), 1.80 – 1.61 (m, 2H), 1.51-1.60 (m, 2H), 1.13 (s, 9H), 1.02 (t, *J* = 7.3 Hz, 3H).

^{13}C NMR (126 MHz, CDCl3) δ 170.7 (C_q), 157.8 (C_q), 147.7 (C_q), 147.5 (C_q), 143.9 (C_q), 138.4 (C_q), 137.9 (CH), 133.9 (C_q), 131.6 (C_q), 130.8 (CH), 129.1 (CH), 128.7 (CH), 128.7 (CH), 127.8 (CH), 126.2 (CH), 126.1 (CH), 125.7 (CH), 119.7 (CH), 76.3 (C_q), 35.2 (C_q), 31.5 (CH_2), 30.4 (CH_3), 30.0 (CH_2), 23.2 (CH_2), 14.3 (CH_3).

$[\alpha]^{D}_{20}$ = 63 (c = 0.1, DCM).

HRMS (ESI): m/z $[M+Na]^+$ $C_{33}H_{34}N_2ONa^+$calculated for 497.2569, found: 497.2563.

HPLC Separation (Chiralpak® IE-3, *n*-Hexane/*i*-PrOH 60/40, 1.0 mL/min, 273 nm): t_r(minor) = 4.7 min, t_r(major) = 21.8 min, 85:15 er.

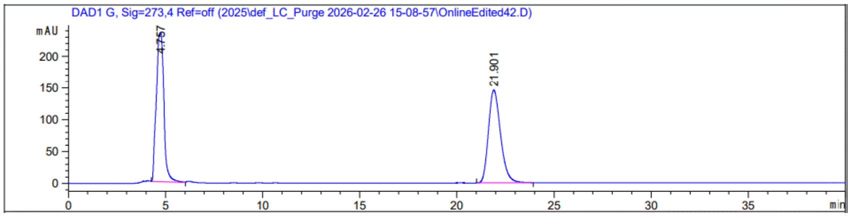

Peak #	RetTime [min]	Type	Width [min]	Area [mAU*s]	Height [mAU]	Area %
1	4.757	MM R	0.4469	6289.25439	234.52841	49.9293
2	21.901	BB	0.6375	6307.06982	145.55814	50.0707

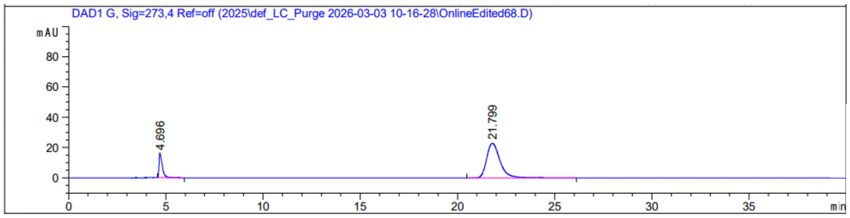

Peak #	RetTime [min]	Type	Width [min]	Area [mAU*s]	Height [mAU]	Area %
1	4.696	MM R	0.1907	186.05313	16.26066	14.6113
2	21.799	MM R	0.7923	1087.29895	22.87314	85.3887

5.Mechanistic studies

Preliminary isotope labelling experiments

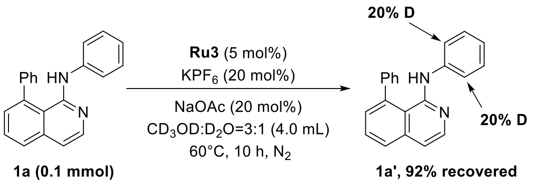

The electrolysis was carried out in an undivided cell setup. A GF anode (20 mm × 10 mm × 4 mm) and a platinum plate cathode (10 mm × 15 mm × 0.25 mm) with electrode holders made of stainless steel were used. A 10 mL Schlenk tube for electrolysis was charged with 1a (0.1 mmol, 1.0 equiv.), **Ru3** (6.3 mg, 0.005 mmol, 5 mol%), KPF6 (3.7 mg, 0.02 mmol, 20 mol%), NaOAc (1.6 mg, 0.02 mmol, 20 mol%). Then a mixture of CD_3OD (3.0 mL) and D_2O (1.0 mL) were added. After sealing, the tube was charged with N_2. The resulting reaction mixture was then stirred at 60℃ with a constant current of 2.0 mA for 10 h. After completion of the reaction, the solvent was then removed under vacuum, and the residue was purified by column chromatography on silica gel to give corresponding product. 92% of the starting material was recovered and found H/D exchange occurred on the phenyl ring at the ortho-position (20% D).

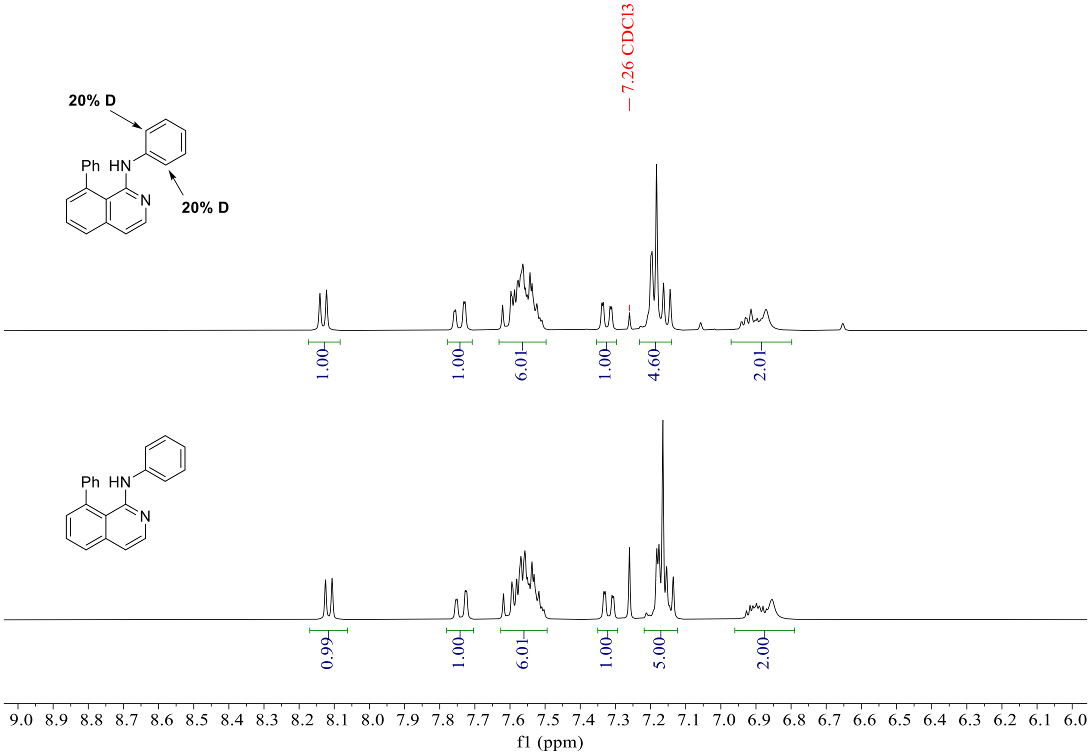

Figure S2. ^{1}H NMR of the product 1a-D of this reaction

Kinetic isotope effect (KIE) experiments by parallel reactions

The electrolysis was carried out in an undivided cell setup. A GF anode (20 mm × 10 mm × 4 mm) and a platinum plate cathode (10 mm × 15 mm × 0.25 mm) with electrode holders made of stainless steel were used. A 10 mL Schlenk tube for electrolysis was charged with **1a** (0.3 mmol, 3.0 equiv.), **2a** (0.1 mmol, 1.0 equiv.), **Ru3** (6.3 mg, 0.005 mmol, 5 mol%), KPF6 (3.7 mg, 0.02 mmol, 20 mol%), NaOAc (1.6 mg, 0.02 mmol, 20 mol%). Then a mixture of HFIP (3.0 mL) and H_2O (1.0 mL) were added. After sealing, the tube was charged with N_2. The resulting reaction mixture was then stirred at 60℃ with a constant current of 2.0 mA for 30 min. After removal of the solvent, the residue was purified by flash chromatography on silica gel to provide product **3** (24.0 mg, yield 51%).

The electrolysis was carried out in an undivided cell setup. A GF anode (20 mm × 10 mm × 4 mm) and a platinum plate cathode (10 mm × 15 mm × 0.25 mm) with electrode holders made of stainless steel were used. A 10 mL Schlenk tube for electrolysis was charged with **1a’** (0.3 mmol, 3.0 equiv.), **2a** (0.1 mmol, 1.0 equiv.), **Ru3** (6.3 mg, 0.005 mmol, 5 mol%), KPF6 (3.7 mg, 0.02 mmol, 20 mol%), NaOAc (1.6 mg, 0.02 mmol, 20 mol%). Then a mixture of HFIP (3.0 mL) and H_2O (1.0 mL) were added. After sealing, the tube was charged with N_2. The resulting reaction mixture was then stirred at 60℃ with a constant current of 2.0 mA for 30 min. After removal of the solvent, the residue was purified by flash chromatography on silica gel to provide product **3’** (10.5 mg, yield 22%).

A kinetic isotopic effect of these two reactions was determined to be k_H/k_D =2.3.

Capture of the C−H activation intermediate

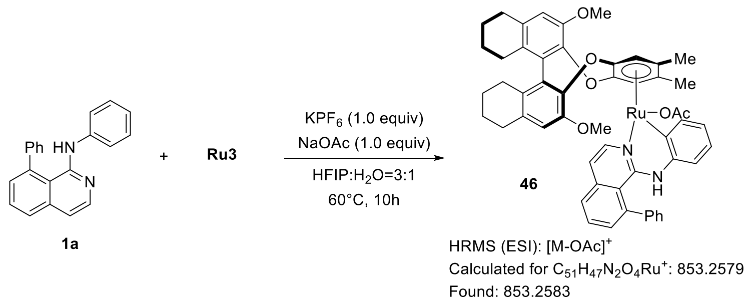

To a mixture of **1a** (0.02 mmol), **Ru3** (0.01 mmol, 0.5 equiv.), KPF_6 (0.02 mmol, 1.0 equiv.), NaOAc (0.02 mmol, 1.0 equiv.), 1.0 mL of the MeOH was added. Then the tube was sealed and the resulting mixture was stirred at 60℃ for 10

h. The reaction mixture was filtered and diluted with MeOH. Then the sample was analyzed *via* HRMS.

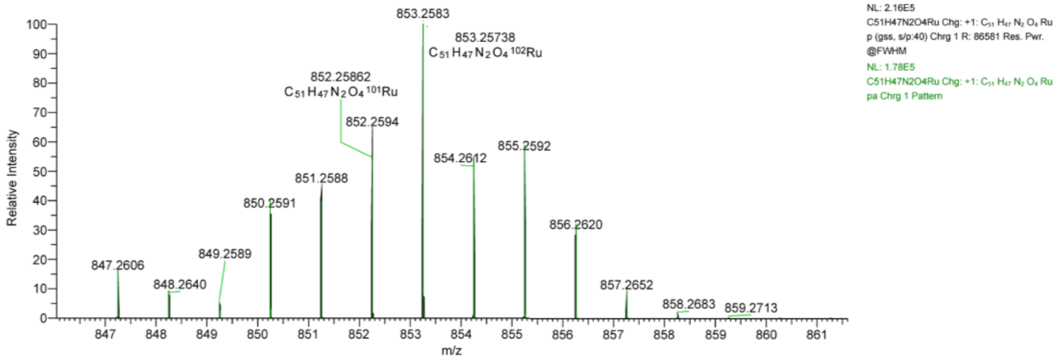

Figure S3. HRMS analysis of the C−H activation intermediate.

Cyclic Voltammetry studies

CV measurements were conducted with a Metrohm Autolab PGSTAT204 potentiostat and Nova 2.1 software. A glassy carbon working electrode (disk, diameter: 3mm), a coiled platinum wire counter electrode, and a SCE reference electrode were employed. The voltammograms were recorded at room temperature in a mixture of HFIP and H_2O with the ration of 3:1, at a substrate concentration of 5 mM KPF_6 and 5 mM NaOAc as supporting electrolytes. All solutions were degassed with N_2 before the measurement and an overpressure of protective gas was maintained throughout the experiment. The scan rate is 100 mV/s. The respective figures and descriptions indicate deviations from the general experimental conditions. For the curve 1a+Ru in Figure S4 and the curve 1a:Ru = 1:1 in Figure S5, the voltammograms were recorded with concentration of 5 mM **1a** and 2.5 mM **Ru3**, with a ratio of the substrate **1a** to the ruthenium catalyst monomer is 1:1. For the curve 1a:Ru = 2:1 in Figure S5, the voltammograms were recorded with concentration of 6 mM **1a** and 1.5 mM **Ru3**, with a ratio of the substrate **1a** to the ruthenium catalyst monomer is 2:1.

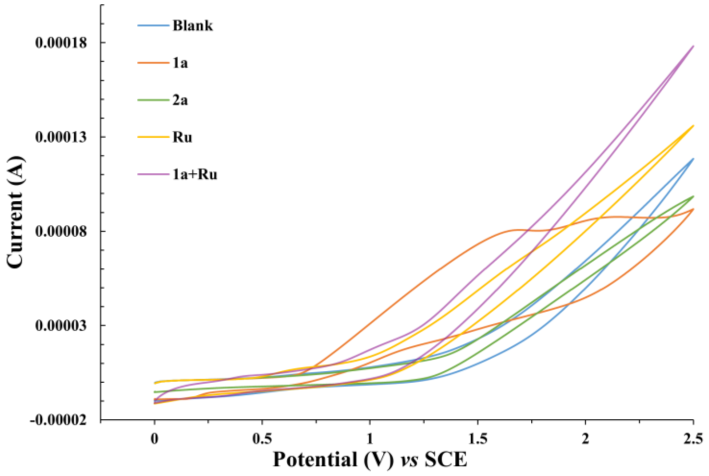

Figure S4. Cyclic voltammograms for ruthenium(II)-catalyzed electrolytic synthesis of atroposelective indole.

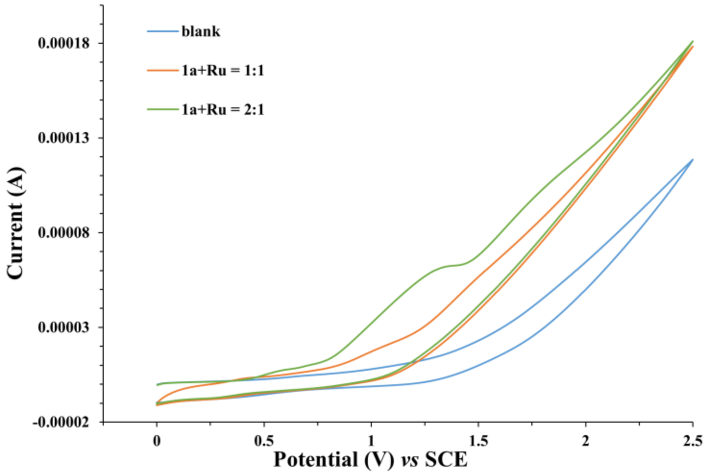

Figure S5. Cyclic voltammograms for different ratio of 1a/Ru.

Cathodic Process Study

In a reaction conducted under standard condition of the synthesis of atroposelective indole, after completion of the reaction, 1 mL sample of the headspace was carefully collected using a gas syringe. The sample was then analyzed via gas chromatography. The H_2 peak was observed at a retention time of 1.56 min, as confirmed by the comparison with pure hydrogen gas as reference sample.

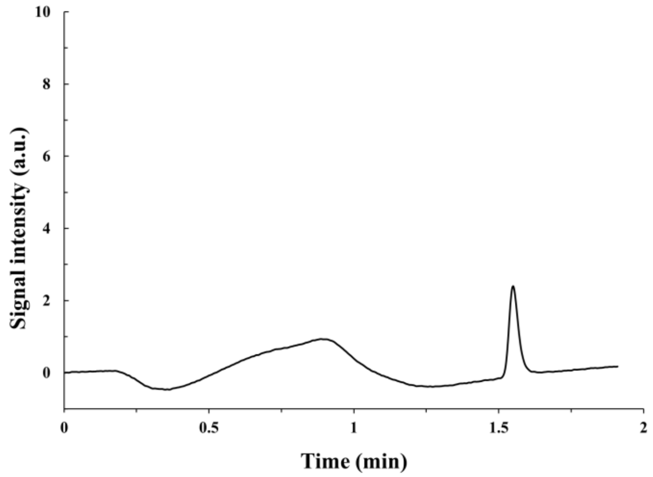

Figure S6. GC analysis of the reaction of the synthesis of atroposelective indole.

Proposed mechanism for the synthesis of chiral spiropyrazolones

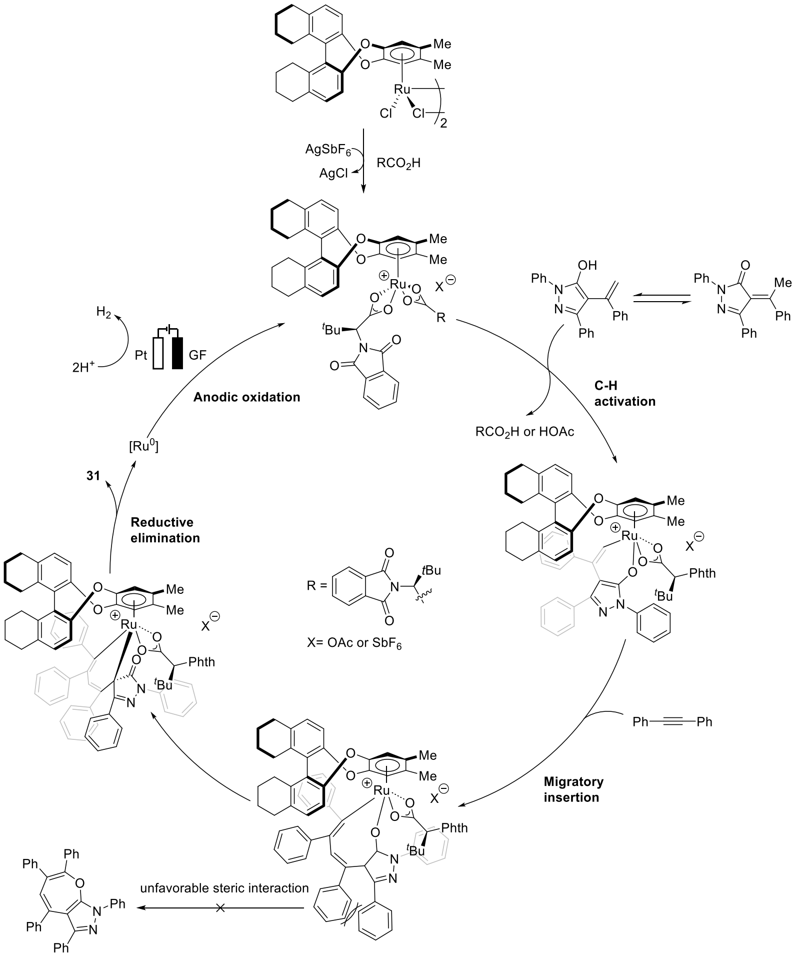

Figure S7. Proposed mechanism for the synthesis of chiral spiropyrazolone 31.

6. Computational Studies

All density functional theory calculations were performed using the ORCA 6.0.1.[7,8] Geometry optimizations of all the stationary points were carried out at the PBE0[9,10] level of theory. Dispersion corrections were included through the D3 formalism with a Becke-Johnson damping scheme (D3(BJ)).[11,12] Ruthenium was described with a def2-TZVP[13-14] basis set in combination with an SDD[15,16] pseudopotential, while all remaining atoms were described with a def2-SVP basis set.[13-15] All stationary points were characterized as intermediates (no imaginary frequency) or transition states (single imaginary frequency) through analytical frequency calculations carried out at the same level of theory. Thermal and non-thermal corrections to the free energy in the gas phase were calculated at 333.15 K and 1 atm. Electronic energies were further refined by single-point calculations on the optimized geometries with a def2-TZVP basis set for all atoms in combination with an SDD pseudopotential for ruthenium, where solvent effects were included through the implicit solvent model SMD[18] for HFIP, the solvent employed in the experiments. As HFIP is not included in ORCA's default solvents, solvent parameters were defined using published literature values.[19-21] The latter encompass the static dielectric constant (epsilon = 16.7)[22-24] and the index of refraction at optical frequencies of 293 K (soln = 1.275)[25] and 298 K (soln25 = 1.2752),[26] with an Abhrahm's hydrogen bond acidity of sola = 1.96, an Abhrahm's hydrogen bond basicity of solb = 0.00, a relative macroscopic surface tension of solg = 23.23,[27,28] an aromaticity of solc = 0.00, fraction of non-hydrogenic solvent atoms that are aromatic carbon atoms, and electronegative halogenicity of solh = 0.60, which represents the fraction of non-hydrogenic solvent atoms that are F, Cl, or Br. These parameters were specified in the single-point energy calculations by including the keyword "smd true" in the %cpcm block with SMDsolvent "2-PROPANOL". Non-covalent interactions analysis (NCIs) was performed using the program NCIPLOT,[29] where the respective NCI isosurfaces were plotted using the VMD 2.0.0a8 program.[30]

Effect of isoquinoline's substituents at the 8th position on the rotational barrier

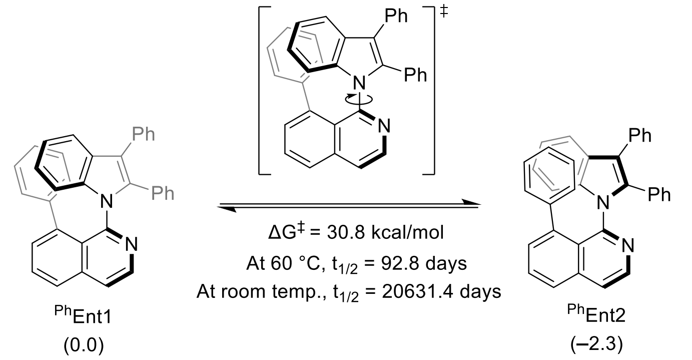

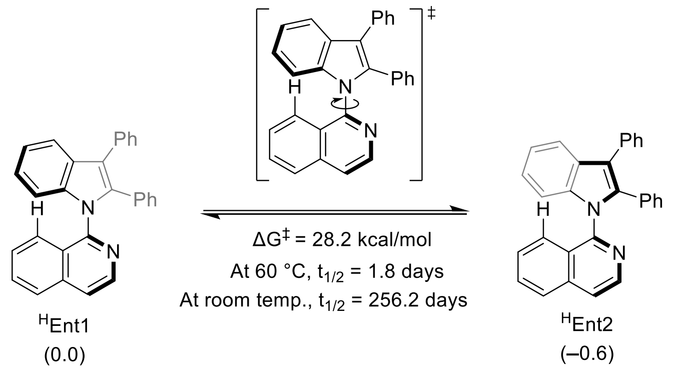

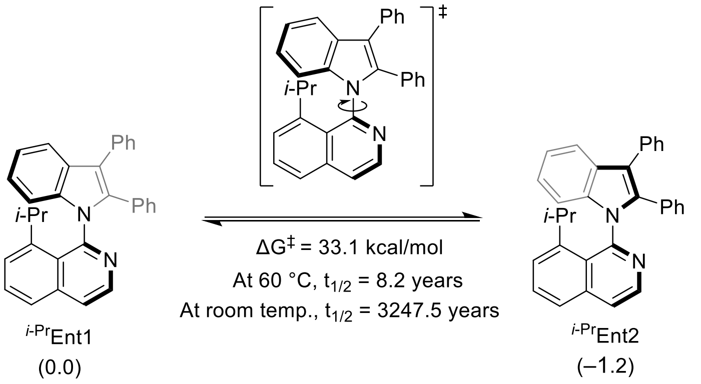

Table S1. Calculated electronic and Gibbs free energies (in Hartree) for all the reported structures along the pathway for enantiomer 1 (in Hartree) at the PBE0-D3(BJ)/def2-TZVP,SDD(Ru)+SMD(HFIP)//PBE0-D3(BJ)/def2-SVP,def2-TZVP(Ru),SDD(Ru) level of theory.[a]

Structure	Electronic Energy	Total Gibbs Free Energy
**^{ent1}Int-1**	-3471.671485	-3470.606683
**^{ent1}TS(1-2)**	-3471.656352	-3470.591735
**^{ent1}Int-2**	-3471.697776	-3470.630497
**^{ent2}Int-1**	-3471.667406	-3470.601613
**^{ent2}TS(1-2)**	-3471.646279	-3470.579325
**^{ent2}Int-2**	-3471.702018	-3470.631998

[a] Superscripts correspond to the respective enantiomer pathway.

Table S2. Calculated electronic and Gibbs free energies (in Hartree) for all the reported structures involved in the determination of rotation barriers with products bearing Ph, H and *i*-Pr substituents at the isoquinolines' 8th position at the PBE0-D3(BJ)/def2-TZVP+SMD(HFIP)//PBE0-D3(BJ)/def2-SVP level of theory.

Structure	Electronic Energy	Total Gibbs Free Energy
**^{Ph}Ent1**	-1456.589685	-1456.158589
**^{Ph}TSrot**	-1456.540301	-1456.109475
**^{Ph}Ent2**	-1456.593193	-1456.162206
**^{H}Ent1**	-1225.718099	-1225.363931
**^{H}TSrot**	-1225.673978	-1225.319040
**^{H}Ent2**	-1225.719082	-1225.364842
**$^{i\text{-}Pr}$Ent1**	-1343.557930	-1343.123688
**$^{i\text{-}Pr}$TSrot**	-1343.505498	-1343.070905
**$^{i\text{-}Pr}$Ent2**	-1343.557924	-1343.123685

7. Cartesian coordinates of the optimized structure

^{ent1}Int-1

Lowest frequency = 5.95 cm^{-1}

Charge = 0, Multiplicity = 1

Ru	6.050296000	22.938262000	10.087014000
O	6.568382000	21.799854000	7.251627000
O	7.420616000	18.498132000	10.683196000
O	5.765651000	19.511316000	8.925465000
O	5.284493000	23.071615000	5.282142000
C	6.405614000	19.726002000	6.101698000
C	6.128892000	21.080468000	6.164847000
C	4.904888000	23.843593000	4.172409000
H	5.519454000	23.602403000	3.289981000
H	5.092352000	24.890855000	4.439114000
H	3.838968000	23.699413000	3.921696000
C	5.873959000	18.946329000	5.051718000
C	6.068072000	17.448838000	5.054267000
H	7.112476000	17.212673000	4.782616000
H	5.946208000	17.071380000	6.081822000
C	5.130913000	16.724300000	4.099115000
H	5.434601000	15.669850000	4.007211000
H	4.105280000	16.721916000	4.509320000
C	5.124445000	17.412916000	2.743400000
H	4.532358000	16.843635000	2.009812000
H	6.156530000	17.455010000	2.352210000
C	4.568910000	18.822107000	2.879082000
H	4.716161000	19.391382000	1.946921000
H	3.474262000	18.767742000	3.025748000
C	5.166893000	19.593886000	4.031221000
C	7.846577000	18.506299000	9.395790000
C	5.644790000	21.894769000	8.237675000
C	4.813658000	23.037181000	8.319062000
C	3.892522000	23.235344000	9.390984000
C	3.887086000	22.281716000	10.429480000
C	4.580359000	21.034746000	10.226755000
C	5.366654000	20.776627000	9.113615000
C	5.437185000	21.744533000	5.139076000
C	7.343480000	19.164355000	7.106150000
C	6.997537000	19.103309000	8.451619000
C	8.211814000	17.838251000	11.632475000

H	9.209517000	18.300572000	11.732934000
H	8.339271000	16.770550000	11.384150000
H	7.690514000	17.923070000	12.594560000
C	9.112136000	19.051421000	5.298068000
H	9.309586000	20.141154000	5.279049000
H	8.310333000	18.899444000	4.563474000
C	8.627810000	18.740692000	6.691535000
C	9.477033000	18.146205000	7.633770000
C	11.397242000	18.328732000	5.998779000
H	11.633547000	19.383425000	6.220758000
H	12.335924000	17.842392000	5.689119000
C	10.356791000	18.279555000	4.892115000
H	10.103843000	17.224544000	4.677015000
H	10.760496000	18.701878000	3.958761000
C	9.063137000	17.998841000	8.962691000
H	9.746733000	17.528678000	9.671132000
C	4.980904000	20.984787000	4.068290000
H	4.445282000	21.466411000	3.248118000
C	10.851973000	17.654886000	7.248250000
H	11.538744000	17.791461000	8.099935000
H	10.799620000	16.562949000	7.079380000
H	4.950061000	23.790425000	7.543390000
H	4.471752000	20.231718000	10.956304000
C	2.954864000	24.398259000	9.365227000
H	1.989126000	24.063999000	8.949051000
H	2.760199000	24.803033000	10.365723000
H	3.342083000	25.208568000	8.736201000
C	3.005841000	22.434961000	11.633123000
H	1.946161000	22.326836000	11.349839000
H	3.235925000	21.675096000	12.389962000
H	3.128080000	23.421160000	12.101763000
N	8.053928000	22.658422000	9.641921000
N	8.067833000	24.327077000	8.005558000
C	10.058790000	23.047921000	8.339700000
C	10.594628000	21.816701000	8.797363000
C	8.689445000	23.353340000	8.689022000
C	10.880885000	23.923449000	7.568625000
C	11.839353000	21.367792000	8.310458000
C	9.861696000	21.098143000	9.779088000
C	12.086341000	23.431458000	7.089743000
C	12.547068000	22.148135000	7.423467000

C	8.658213000	21.572964000	10.199807000
H	12.225692000	20.403070000	8.647427000
H	10.267106000	20.180739000	10.202853000
H	12.715039000	24.091122000	6.488047000
H	13.501934000	21.798401000	7.023535000
H	8.087897000	21.066041000	10.977686000
C	10.608455000	25.371093000	7.379410000
C	10.374563000	26.179828000	8.500873000
C	10.679697000	25.973277000	6.118852000
C	10.217908000	27.554565000	8.365597000
C	10.515362000	27.350644000	5.983070000
C	10.289667000	28.146695000	7.104262000
H	10.312626000	25.722766000	9.490316000
H	10.815529000	25.362432000	5.225653000
H	10.029186000	28.162753000	9.253460000
H	10.552750000	27.786619000	4.982473000
H	10.161340000	29.226651000	6.995674000
C	7.137540000	25.221328000	8.513979000
C	7.045820000	26.482034000	7.903695000
C	6.322759000	24.904250000	9.609583000
C	6.205287000	27.454991000	8.432391000
C	5.521706000	25.910535000	10.149333000
C	5.461870000	27.184752000	9.580269000
H	7.647531000	26.684230000	7.015226000
H	6.148578000	28.434541000	7.952077000
H	4.901369000	25.685199000	11.022626000
H	4.818639000	27.951744000	10.019460000
C	6.392535000	22.771764000	12.226753000
C	7.074855000	23.779058000	11.920197000
C	8.143437000	24.740108000	12.030094000
C	7.906417000	26.111858000	12.203720000
C	9.471505000	24.279801000	11.988611000
C	8.970749000	26.999549000	12.318488000
C	10.530141000	25.173441000	12.104773000
C	10.284388000	26.536689000	12.264774000
H	6.882994000	26.480531000	12.238655000
H	9.667096000	23.214787000	11.857406000
H	8.769193000	28.065532000	12.445089000
H	11.555680000	24.800456000	12.059681000
H	11.117920000	27.238328000	12.343718000
C	5.870516000	21.869804000	13.229643000

C	5.291532000	22.403163000	14.391956000
C	5.952477000	20.475998000	13.087481000
C	4.793208000	21.560983000	15.381417000
C	5.457273000	19.640217000	14.083755000
C	4.870395000	20.177117000	15.229989000
H	5.233582000	23.487623000	14.504475000
H	6.398610000	20.048782000	12.186645000
H	4.340689000	21.989761000	16.278635000
H	5.522940000	18.556299000	13.960411000
H	4.475433000	19.516835000	16.005345000
H	8.311248000	24.389091000	6.974156000
C	8.731914000	23.110979000	4.917366000
O	9.140159000	22.190736000	5.621605000
O	8.315502000	24.235959000	5.383984000
C	8.676999000	22.967577000	3.408066000
H	8.992580000	23.902957000	2.922662000
H	9.303644000	22.128251000	3.080708000
H	7.638300000	22.756943000	3.106205000
H	7.819281000	25.337124000	4.640839000
O	7.385923000	26.213095000	4.246528000
O	9.266031000	26.369545000	3.040820000
C	8.202139000	26.818796000	3.419377000
C	7.697232000	28.176136000	2.993726000
H	7.774756000	28.865335000	3.849303000
H	8.298750000	28.560512000	2.161833000
H	6.635425000	28.125317000	2.714104000

^{ent1}TS(1-2)

Lowest frequency = -217.32 cm^{-1}

Charge = 0, Multiplicity = 1

Ru	5.931130000	22.819677000	9.929022000
O	6.454147000	21.824019000	7.013458000
O	7.080499000	18.472606000	10.526635000
O	5.595901000	19.544465000	8.661727000
O	5.134926000	22.989185000	5.009566000
C	6.421438000	19.724173000	5.902156000
C	6.071907000	21.061857000	5.936132000
C	4.634137000	23.703471000	3.910283000
H	5.217913000	23.494949000	2.998143000
H	4.750699000	24.766984000	4.152219000

H	3.572559000	23.465153000	3.720180000
C	5.969467000	18.903645000	4.845092000
C	6.254291000	17.420971000	4.867754000
H	7.317476000	17.248182000	4.622171000
H	6.132854000	17.045661000	5.896036000
C	5.386337000	16.630865000	3.899500000
H	5.755529000	15.595954000	3.827309000
H	4.353301000	16.570654000	4.285945000
C	5.370808000	17.303774000	2.536092000
H	4.831783000	16.692503000	1.795409000
H	6.407640000	17.403961000	2.168787000
C	4.728385000	18.677999000	2.642911000
H	4.864103000	19.246041000	1.708203000
H	3.635671000	18.558954000	2.763628000
C	5.249344000	19.496058000	3.800653000
C	7.618127000	18.502918000	9.283202000
C	5.511587000	21.925439000	7.980155000
C	4.757920000	23.106647000	8.102686000
C	3.855857000	23.317506000	9.191015000
C	3.770457000	22.330321000	10.203530000
C	4.428296000	21.072837000	9.983605000
C	5.218600000	20.819806000	8.862012000
C	5.363099000	21.670328000	4.887542000
C	7.307529000	19.202343000	6.971867000
C	6.854871000	19.126325000	8.284263000
C	7.792375000	17.812147000	11.535593000
H	8.771856000	18.284995000	11.728098000
H	7.954342000	16.748283000	11.290297000
H	7.186932000	17.882176000	12.447984000
C	9.221801000	19.144044000	5.320158000
H	9.416543000	20.234102000	5.344175000
H	8.483015000	19.008919000	4.518113000
C	8.624708000	18.795864000	6.660691000
C	9.392297000	18.181130000	7.658107000
C	11.441315000	18.406333000	6.193146000
H	11.656449000	19.455769000	6.457446000
H	12.403915000	17.928308000	5.951151000
C	10.497889000	18.382751000	5.001731000
H	10.264975000	17.333347000	4.741839000
H	10.978185000	18.826492000	4.115870000
C	8.865002000	17.998106000	8.942628000

H	9.483896000	17.507676000	9.695611000
C	4.972062000	20.871888000	3.819500000
H	4.422430000	21.308446000	2.983716000
C	10.797792000	17.704384000	7.379079000
H	11.410854000	17.828893000	8.287111000
H	10.767990000	16.615582000	7.186490000
H	4.948932000	23.887318000	7.365598000
H	4.306542000	20.256351000	10.696045000
C	2.993250000	24.539391000	9.194910000
H	2.026860000	24.281695000	8.729388000
H	2.784678000	24.911022000	10.204612000
H	3.448139000	25.352471000	8.616622000
C	2.914231000	22.498335000	11.422130000
H	1.853158000	22.317363000	11.184556000
H	3.216096000	21.789637000	12.204573000
H	2.998302000	23.509426000	11.841611000
N	7.962776000	22.488415000	9.586329000
N	8.068593000	24.329854000	8.158657000
C	10.053562000	23.009366000	8.485908000
C	10.587047000	21.773691000	8.943219000
C	8.664456000	23.277081000	8.769401000
C	10.906232000	23.909152000	7.774997000
C	11.875508000	21.365024000	8.537710000
C	9.805726000	20.995949000	9.834064000
C	12.149970000	23.452855000	7.369122000
C	12.624010000	22.177222000	7.717955000
C	8.547151000	21.402459000	10.156314000
H	12.258179000	20.401407000	8.881790000
H	10.209345000	20.075631000	10.253840000
H	12.794282000	24.134561000	6.809979000
H	13.612556000	21.858268000	7.378942000
H	7.920627000	20.840739000	10.847963000
C	10.614009000	25.350047000	7.561838000
C	10.424528000	26.178514000	8.676283000
C	10.619470000	25.922087000	6.286341000
C	10.238802000	27.547089000	8.515896000
C	10.418239000	27.292092000	6.124983000
C	10.232696000	28.108852000	7.238390000
H	10.425753000	25.740813000	9.676609000
H	10.736224000	25.291259000	5.404963000
H	10.090634000	28.177006000	9.396218000

H	10.386208000	27.701004000	5.113031000
H	10.076985000	29.183266000	7.111888000
C	7.163070000	25.200570000	8.721244000
C	7.029958000	26.471916000	8.141481000
C	6.342924000	24.850476000	9.819218000
C	6.162552000	27.410789000	8.688835000
C	5.496632000	25.823208000	10.367115000
C	5.405992000	27.102479000	9.821085000
H	7.619199000	26.706775000	7.252936000
H	6.089148000	28.399141000	8.229098000
H	4.891270000	25.556385000	11.237467000
H	4.738631000	27.844245000	10.265629000
C	6.374464000	22.937948000	11.962455000
C	7.025014000	23.975827000	11.574027000
C	8.244626000	24.727386000	11.840214000
C	8.355536000	26.121944000	11.739045000
C	9.356670000	23.988733000	12.282752000
C	9.538226000	26.758336000	12.098812000
C	10.544803000	24.629882000	12.617100000
C	10.638269000	26.018056000	12.531514000
H	7.508219000	26.704942000	11.378246000
H	9.278897000	22.902195000	12.344918000
H	9.605031000	27.846293000	12.027517000
H	11.404307000	24.039057000	12.941461000
H	11.571257000	26.522990000	12.792033000
C	6.078416000	22.163376000	13.144494000
C	5.963061000	22.802868000	14.392197000
C	5.913796000	20.769011000	13.086429000
C	5.686773000	22.069038000	15.540568000
C	5.646107000	20.039668000	14.240944000
C	5.525698000	20.684719000	15.471804000
H	6.088083000	23.886616000	14.443705000
H	6.010730000	20.262160000	12.123920000
H	5.595502000	22.582862000	16.500614000
H	5.521382000	18.955402000	14.178943000
H	5.305195000	20.111023000	16.374806000
H	8.285735000	24.443301000	7.121162000
C	8.648541000	23.237563000	5.040598000
O	9.163492000	22.343222000	5.705573000
O	8.195557000	24.334211000	5.544627000
C	8.520403000	23.117112000	3.534596000

H	8.978125000	23.997889000	3.058720000
H	8.989209000	22.192584000	3.176348000
H	7.456512000	23.115659000	3.254616000
H	7.555993000	25.335982000	4.784059000
O	7.020670000	26.122945000	4.322296000
O	8.950186000	26.542183000	3.264577000
C	7.794054000	26.814560000	3.521155000
C	7.095579000	28.020654000	2.938971000
H	6.833123000	28.717146000	3.749867000
H	7.747380000	28.522344000	2.214638000
H	6.153757000	27.718014000	2.458211000

^{ent1}Int-2

Lowest frequency = 14.17 cm^{-1}

Charge = 0, Multiplicity = 1

Ru	5.803344000	22.862185000	9.755503000
O	6.254023000	21.920068000	6.817890000
O	6.863126000	18.411788000	10.345674000
O	5.490793000	19.646218000	8.522914000
O	4.940889000	22.992487000	4.781685000
C	6.350168000	19.800848000	5.745447000
C	5.928620000	21.117862000	5.755519000
C	4.457987000	23.674535000	3.654997000
H	5.099639000	23.497200000	2.775124000
H	4.500613000	24.742298000	3.901225000
H	3.422318000	23.378266000	3.411301000
C	5.970532000	18.951833000	4.679578000
C	6.326476000	17.484680000	4.720541000
H	7.403589000	17.362642000	4.508436000
H	6.192565000	17.109261000	5.747126000
C	5.529164000	16.646700000	3.732181000
H	5.951611000	15.631139000	3.680190000
H	4.488660000	16.536854000	4.086324000
C	5.523842000	17.308689000	2.363642000
H	5.038063000	16.667421000	1.611639000
H	6.565485000	17.456399000	2.027131000
C	4.812817000	18.650454000	2.440454000
H	4.950558000	19.219498000	1.506605000
H	3.723815000	18.479461000	2.527419000
C	5.255375000	19.500067000	3.608086000

C	7.449531000	18.499153000	9.128331000
C	5.321268000	22.003820000	7.799567000
C	4.570813000	23.180136000	7.961216000
C	3.738332000	23.387015000	9.109646000
C	3.701925000	22.404470000	10.137084000
C	4.389204000	21.171790000	9.907317000
C	5.114528000	20.923570000	8.724447000
C	5.225555000	21.682245000	4.678885000
C	7.221485000	19.306656000	6.842260000
C	6.735458000	19.197732000	8.141103000
C	7.524378000	17.692294000	11.348966000
H	8.492517000	18.151517000	11.616166000
H	7.697361000	16.643567000	11.051646000
H	6.873406000	17.711503000	12.231876000
C	9.195950000	19.315696000	5.262162000
H	9.385508000	20.403891000	5.343710000
H	8.491108000	19.205583000	4.425906000
C	8.550661000	18.910376000	6.563712000
C	9.282537000	18.253001000	7.560209000
C	11.387204000	18.572728000	6.200084000
H	11.574091000	19.611218000	6.524426000
H	12.367612000	18.121637000	5.978986000
C	10.497350000	18.588015000	4.967462000
H	10.296003000	17.547704000	4.650649000
H	11.007791000	19.079009000	4.124389000
C	8.702687000	17.998512000	8.809350000
H	9.289282000	17.464878000	9.558759000
C	4.909160000	20.860666000	3.604992000
H	4.368346000	21.263959000	2.747094000
C	10.703816000	17.804104000	7.320107000
H	11.274333000	17.893274000	8.259180000
H	10.697850000	16.725868000	7.073588000
H	4.700601000	23.962520000	7.212978000
H	4.324743000	20.351805000	10.623200000
C	2.871489000	24.604956000	9.172122000
H	1.845416000	24.318601000	8.887038000
H	2.829953000	25.045750000	10.174687000
H	3.217168000	25.375979000	8.474510000
C	2.918942000	22.583820000	11.401175000
H	1.856338000	22.334904000	11.245049000
H	3.314771000	21.930047000	12.190056000

H	2.974475000	23.616214000	11.768561000
N	7.861548000	22.378634000	9.518826000
N	8.191464000	24.312932000	8.302030000
C	10.111487000	22.908638000	8.773963000
C	10.561892000	21.658243000	9.281237000
C	8.709934000	23.195083000	8.894918000
C	11.059005000	23.789495000	8.165670000
C	11.897799000	21.246163000	9.079655000
C	9.640006000	20.860307000	9.997484000
C	12.350317000	23.334002000	7.966733000
C	12.768942000	22.062843000	8.400135000
C	8.341137000	21.254865000	10.109394000
H	12.215494000	20.274793000	9.465300000
H	9.964620000	19.922543000	10.446599000
H	13.066027000	24.004087000	7.486033000
H	13.799192000	21.745186000	8.223042000
H	7.613829000	20.662949000	10.657356000
C	10.759487000	25.193394000	7.787881000
C	10.483707000	26.138680000	8.784039000
C	10.774717000	25.593590000	6.450513000
C	10.197208000	27.454377000	8.440078000
C	10.473468000	26.910159000	6.103627000
C	10.182489000	27.841038000	7.097696000
H	10.476852000	25.830935000	9.831956000
H	10.976386000	24.852928000	5.675040000
H	9.976741000	28.181079000	9.226275000
H	10.428931000	27.186475000	5.048158000
H	9.938678000	28.871744000	6.827791000
C	7.175031000	25.038202000	8.809858000
C	6.634150000	26.036203000	7.942326000
C	6.642337000	24.917653000	10.145087000
C	5.661670000	26.899923000	8.369511000
C	5.578422000	25.816462000	10.501034000
C	5.113204000	26.799650000	9.669835000
H	7.040428000	26.116069000	6.934880000
H	5.304270000	27.671944000	7.683015000
H	5.186956000	25.711437000	11.516031000
H	4.330389000	27.485421000	9.998855000
C	6.543759000	23.176888000	11.662234000
C	7.260962000	24.289258000	11.376381000
C	8.441243000	24.905185000	11.990154000

C	8.679904000	26.284119000	11.875289000
C	9.409652000	24.109304000	12.626351000
C	9.835920000	26.853382000	12.402744000
C	10.565981000	24.679110000	13.145382000
C	10.784013000	26.054400000	13.039916000
H	7.948313000	26.912773000	11.361453000
H	9.251085000	23.030302000	12.684444000
H	10.002704000	27.929179000	12.306820000
H	11.313808000	24.043038000	13.625243000
H	11.696690000	26.499133000	13.443148000
C	6.474341000	22.419724000	12.906275000
C	6.628786000	23.069615000	14.147511000
C	6.188536000	21.042562000	12.927391000
C	6.527653000	22.371048000	15.344354000
C	6.092862000	20.341769000	14.126711000
C	6.263541000	21.000085000	15.343232000
H	6.825404000	24.143682000	14.157721000
H	6.041463000	20.517441000	11.982188000
H	6.651043000	22.902747000	16.291258000
H	5.871413000	19.271175000	14.111645000
H	6.180214000	20.452704000	16.284982000
H	8.325545000	24.383355000	7.230918000
C	8.683134000	23.272097000	5.204110000
O	9.340762000	22.481034000	5.875319000
O	8.120150000	24.321426000	5.699472000
C	8.494523000	23.077778000	3.714876000
H	8.823880000	23.987975000	3.190667000
H	9.051954000	22.202267000	3.360584000
H	7.424805000	22.940621000	3.498960000
H	7.372711000	25.248390000	4.904670000
O	6.791395000	26.002972000	4.458648000
O	8.655461000	26.472887000	3.308741000
C	7.509782000	26.719479000	3.621848000
C	6.750516000	27.905089000	3.077378000
H	6.474754000	28.578195000	3.903169000
H	7.365487000	28.443566000	2.347460000
H	5.813485000	27.569870000	2.608498000

^{ent2}Int-1

Lowest frequency = 11.64 cm^{-1}

Charge = 0, Multiplicity = 1

Ru	5.295695000	22.991633000	9.854944000
O	6.351503000	22.431434000	6.766920000
O	7.036133000	18.735298000	9.955904000
O	5.659299000	19.923841000	8.106770000
O	4.899225000	23.534685000	4.825972000
C	6.698127000	20.454157000	5.481510000
C	6.142048000	21.715654000	5.609520000
C	4.113599000	24.147940000	3.840492000
H	4.670391000	24.272797000	2.895794000
H	3.840903000	25.138333000	4.226074000
H	3.192542000	23.574234000	3.638423000
C	6.416277000	19.675059000	4.335330000
C	6.909315000	18.250168000	4.250796000
H	7.990704000	18.246070000	4.026070000
H	6.821427000	17.779613000	5.242254000
C	6.183653000	17.428991000	3.195661000
H	6.692904000	16.462177000	3.062570000
H	5.159068000	17.200320000	3.538308000
C	6.115170000	18.197813000	1.886027000
H	5.684437000	17.580883000	1.082210000
H	7.138379000	18.463407000	1.565595000
C	5.287846000	19.460357000	2.069753000
H	5.378452000	20.119303000	1.190703000
H	4.218212000	19.187214000	2.128466000
C	5.643080000	20.239784000	3.313603000
C	7.684821000	18.962446000	8.814304000
C	5.337995000	22.312663000	7.671253000
C	4.477931000	23.426810000	7.916880000
C	3.343336000	23.363552000	8.769914000
C	3.165463000	22.161658000	9.513844000
C	4.106869000	21.105238000	9.375237000
C	5.083538000	21.099543000	8.350279000
C	5.365429000	22.289024000	4.591141000
C	7.550550000	19.931228000	6.577563000
C	6.983673000	19.667630000	7.818256000
C	7.555462000	17.779657000	10.859740000
H	8.535803000	18.087498000	11.255327000
H	7.647145000	16.793933000	10.376637000
H	6.847537000	17.707431000	11.688783000
C	9.615640000	20.161308000	5.125237000

H	9.830621000	21.234307000	5.287711000
H	8.942473000	20.138168000	4.256770000
C	8.916623000	19.642884000	6.360104000
C	9.623326000	18.944495000	7.347652000
C	11.778279000	19.346647000	6.070378000
H	11.983035000	20.367251000	6.440108000
H	12.756089000	18.892021000	5.846781000
C	10.916232000	19.434980000	4.820724000
H	10.697200000	18.415083000	4.455703000
H	11.452279000	19.950461000	4.008442000
C	8.990041000	18.570150000	8.537626000
H	9.552834000	18.015289000	9.289077000
C	5.140629000	21.544127000	3.441069000
H	4.545640000	21.955946000	2.624424000
C	11.065477000	18.542975000	7.147020000
H	11.601546000	18.615141000	8.107173000
H	11.088563000	17.471462000	6.875413000
H	4.672122000	24.336126000	7.347962000
H	4.015285000	20.216565000	10.014144000
C	2.380689000	24.508820000	8.816021000
H	1.530731000	24.327111000	8.138069000
H	1.973569000	24.659452000	9.823257000
H	2.872123000	25.440315000	8.503377000
C	2.001757000	21.911314000	10.414872000
H	1.215776000	21.426669000	9.810843000
H	2.287141000	21.216349000	11.216891000
H	1.587434000	22.824377000	10.853199000
N	7.036149000	22.142156000	10.608536000
N	5.982508000	21.127089000	12.433439000
C	8.333569000	20.805567000	12.157353000
C	9.401853000	20.733712000	11.224923000
C	7.082122000	21.389120000	11.716470000
C	8.532167000	20.325572000	13.486190000
C	10.553552000	19.981288000	11.542173000
C	9.264654000	21.458025000	10.012711000
C	9.666244000	19.568098000	13.748405000
C	10.652557000	19.361126000	12.769556000
C	8.126860000	22.169673000	9.787039000
H	11.356307000	19.904704000	10.804986000
H	10.055642000	21.452848000	9.263028000
H	9.821929000	19.193760000	14.762284000

H	11.533095000	18.761619000	13.012506000
H	8.015414000	22.746515000	8.871884000
C	7.702417000	20.766102000	14.635909000
C	7.202286000	19.864304000	15.579036000
C	7.497254000	22.139311000	14.838144000
C	6.494598000	20.326262000	16.688048000
C	6.796189000	22.597627000	15.946903000
C	6.289690000	21.690448000	16.878023000
H	7.321362000	18.790737000	15.426022000
H	7.902038000	22.850792000	14.113745000
H	6.091780000	19.603326000	17.401222000
H	6.639668000	23.670281000	16.083684000
H	5.730547000	22.048835000	17.745472000
C	4.900604000	21.969609000	12.652302000
C	4.185761000	21.761759000	13.844720000
C	4.557111000	23.006063000	11.782292000
C	3.161185000	22.620584000	14.207055000
C	3.545200000	23.881875000	12.194647000
C	2.851143000	23.706774000	13.387565000
H	4.467683000	20.917476000	14.476945000
H	2.617504000	22.453141000	15.139666000
H	3.285262000	24.732919000	11.561268000
H	2.069438000	24.415284000	13.672089000
C	6.529470000	24.693042000	9.436169000
C	5.896192000	24.930044000	10.510997000
C	5.672172000	25.874749000	11.583334000
C	5.984944000	25.562790000	12.915219000
C	5.169465000	27.149480000	11.283167000
C	5.806741000	26.509212000	13.917063000
C	4.983738000	28.090544000	12.292416000
C	5.301331000	27.773264000	13.611948000
H	6.350703000	24.564442000	13.156803000
H	4.926279000	27.392766000	10.246519000
H	6.053403000	26.253137000	14.950130000
H	4.589393000	29.079219000	12.045521000
H	5.153291000	28.510768000	14.404270000
C	7.623818000	25.119785000	8.583538000
C	8.796715000	25.568954000	9.219152000
C	7.572263000	25.138166000	7.181674000
C	9.880345000	26.021440000	8.475533000
C	8.661437000	25.591816000	6.442693000

C	9.818340000	26.032803000	7.082160000
H	8.843377000	25.548669000	10.309763000
H	6.688614000	24.779016000	6.657448000
H	10.782036000	26.364132000	8.988680000
H	8.602915000	25.596227000	5.351658000
H	10.671431000	26.383040000	6.496291000
H	5.902807000	20.184971000	12.889764000
C	4.275099000	18.383649000	12.456611000
O	5.301647000	18.639835000	13.196443000
O	3.738915000	19.200101000	11.708809000
C	3.745753000	16.965425000	12.547451000
H	4.532748000	16.260920000	12.236251000
H	3.499156000	16.723042000	13.592413000
H	2.856517000	16.847728000	11.916762000
H	5.829290000	17.602817000	14.058750000
O	6.222794000	16.933312000	14.767707000
O	6.882005000	15.686447000	13.027855000
C	6.810922000	15.895719000	14.222093000
C	7.409656000	14.966764000	15.249792000
H	6.643571000	14.676514000	15.983329000
H	7.824467000	14.078478000	14.760043000
H	8.202714000	15.491011000	15.804536000

^{ent2}TS(1-2)

Lowest frequency = -247.95 cm^{-1}

Charge = 0, Multiplicity = 1

Ru	5.236574000	22.845024000	9.687042000
O	6.347460000	22.343773000	6.660648000
O	6.976503000	18.684243000	9.888389000
O	5.621113000	19.845213000	8.000685000
O	4.928120000	23.444492000	4.690871000
C	6.692703000	20.354575000	5.390684000
C	6.148683000	21.623376000	5.503217000
C	4.155370000	24.054220000	3.693935000
H	4.720805000	24.167993000	2.752845000
H	3.885856000	25.049556000	4.069006000
H	3.231958000	23.485354000	3.487970000
C	6.413051000	19.567878000	4.248861000
C	6.885948000	18.134958000	4.184557000
H	7.969879000	18.110776000	3.973790000

H	6.777963000	17.675925000	5.179468000
C	6.160926000	17.313770000	3.128934000
H	6.657847000	16.338500000	3.011429000
H	5.129201000	17.102965000	3.461573000
C	6.118499000	18.070471000	1.811184000
H	5.689160000	17.451511000	1.008142000
H	7.148993000	18.319237000	1.500694000
C	5.306159000	19.345589000	1.973218000
H	5.416395000	19.995087000	1.089373000
H	4.232290000	19.087417000	2.021281000
C	5.657163000	20.131759000	3.214019000
C	7.632301000	18.881256000	8.745033000
C	5.305532000	22.237421000	7.542664000
C	4.449892000	23.355614000	7.763617000
C	3.318308000	23.295611000	8.620687000
C	3.098790000	22.078353000	9.344116000
C	3.985199000	20.992274000	9.176518000
C	5.024522000	21.020466000	8.217303000
C	5.387350000	22.194887000	4.471707000
C	7.528908000	19.828449000	6.497781000
C	6.945751000	19.582310000	7.734850000
C	7.509515000	17.782922000	10.837489000
H	8.493855000	18.114862000	11.203883000
H	7.599426000	16.771018000	10.411388000
H	6.813965000	17.757010000	11.679455000
C	9.609245000	20.010426000	5.058220000
H	9.846092000	21.080264000	5.210437000
H	8.940881000	19.992447000	4.186022000
C	8.892530000	19.517975000	6.293736000
C	9.580375000	18.816972000	7.292445000
C	11.750351000	19.164875000	6.024264000
H	11.972178000	20.185735000	6.383394000
H	12.720723000	18.689607000	5.811425000
C	10.896771000	19.255163000	4.768985000
H	10.659592000	18.235545000	4.414672000
H	11.446902000	19.750548000	3.953615000
C	8.933041000	18.466113000	8.482280000
H	9.483740000	17.912379000	9.243339000
C	5.167683000	21.442215000	3.325436000
H	4.585106000	21.853164000	2.499443000
C	11.016429000	18.387405000	7.105868000

H	11.547795000	18.461197000	8.068582000
H	11.021833000	17.312453000	6.847232000
H	4.656559000	24.264180000	7.197291000
H	3.874269000	20.096115000	9.796055000
C	2.384613000	24.466936000	8.670652000
H	1.649105000	24.401323000	7.851982000
H	1.824475000	24.514793000	9.611189000
H	2.937464000	25.409637000	8.553305000
C	1.947047000	21.859479000	10.270475000
H	1.129249000	21.393868000	9.695630000
H	2.241571000	21.163805000	11.069471000
H	1.568941000	22.782961000	10.718934000
N	7.001728000	22.014461000	10.413562000
N	6.049067000	21.271168000	12.412063000
C	8.377063000	20.860371000	12.033860000
C	9.414908000	20.742364000	11.069270000
C	7.113128000	21.415048000	11.600400000
C	8.623426000	20.439330000	13.376190000
C	10.582378000	20.012473000	11.384286000
C	9.239002000	21.392413000	9.821429000
C	9.768026000	19.697874000	13.632819000
C	10.725243000	19.450420000	12.633661000
C	8.069176000	22.039425000	9.566164000
H	11.359155000	19.903560000	10.623811000
H	10.022876000	21.369425000	9.064559000
H	9.955262000	19.365465000	14.656013000
H	11.615490000	18.864951000	12.875183000
H	7.913810000	22.560873000	8.623136000
C	7.827221000	20.906929000	14.539889000
C	7.317583000	20.018778000	15.490081000
C	7.665775000	22.285218000	14.745364000
C	6.636586000	20.497575000	16.608696000
C	6.998256000	22.760205000	15.868301000
C	6.475168000	21.866468000	16.803840000
H	7.406819000	18.942687000	15.336772000
H	8.073796000	22.987192000	14.013607000
H	6.222855000	19.784031000	17.324998000
H	6.888653000	23.837244000	16.015868000
H	5.941762000	22.239799000	17.681201000
C	4.961558000	22.106433000	12.574764000
C	4.188079000	21.870924000	13.727156000

C	4.597859000	23.131896000	11.682626000
C	3.084052000	22.652222000	14.020089000
C	3.471926000	23.907455000	12.012472000
C	2.720367000	23.687944000	13.156553000
H	4.484840000	21.047517000	14.379672000
H	2.509068000	22.456444000	14.927865000
H	3.204529000	24.737893000	11.355508000
H	1.862567000	24.326902000	13.377621000
C	6.395116000	24.537265000	9.794532000
C	5.831540000	24.635336000	10.951825000
C	5.726636000	25.636568000	12.009059000
C	5.770158000	25.329788000	13.377200000
C	5.661512000	26.985114000	11.621098000
C	5.765655000	26.345824000	14.325370000
C	5.653309000	27.998592000	12.575188000
C	5.706644000	27.683137000	13.931669000
H	5.796157000	24.286301000	13.693792000
H	5.619135000	27.229006000	10.557407000
H	5.798645000	26.089199000	15.386988000
H	5.597673000	29.041834000	12.255067000
H	5.694643000	28.477348000	14.681861000
C	7.431862000	25.217583000	9.039478000
C	8.386818000	25.978059000	9.742445000
C	7.540718000	25.138855000	7.642426000
C	9.401176000	26.645841000	9.068582000
C	8.554797000	25.817576000	6.971190000
C	9.487392000	26.574396000	7.676985000
H	8.324325000	26.029806000	10.831152000
H	6.847988000	24.520649000	7.073382000
H	10.134618000	27.225213000	9.634660000
H	8.619975000	25.742638000	5.882960000
H	10.285565000	27.099852000	7.147352000
H	5.946379000	20.345210000	12.897977000
C	4.302980000	18.539970000	12.469677000
O	5.324828000	18.800106000	13.216289000
O	3.764264000	19.358676000	11.726856000
C	3.783964000	17.117185000	12.549589000
H	4.579175000	16.416663000	12.250919000
H	3.522879000	16.871963000	13.590447000
H	2.904528000	16.994212000	11.906244000
H	5.826314000	17.766881000	14.101405000

O	6.197061000	17.105454000	14.829631000
O	6.886594000	15.822408000	13.127871000
C	6.788659000	16.053302000	14.316101000
C	7.355089000	15.138023000	15.373835000
H	6.571822000	14.872579000	16.098530000
H	7.767900000	14.234908000	14.910210000
H	8.144426000	15.663450000	15.932802000

^{ent2}Int-2

Lowest frequency = 16.63 cm^{-1}

Charge = 0, Multiplicity = 1

Ru	4.955167000	22.694876000	9.531707000
O	6.250867000	22.195470000	6.646925000
O	6.986118000	18.795183000	10.118865000
O	5.589971000	19.701242000	8.059848000
O	4.852833000	23.106670000	4.550157000
C	6.756253000	20.172634000	5.502158000
C	6.140166000	21.412206000	5.518581000
C	4.084419000	23.609825000	3.492697000
H	4.678226000	23.706125000	2.567163000
H	3.741789000	24.605862000	3.799925000
H	3.205380000	22.974070000	3.287788000
C	6.581495000	19.309180000	4.397733000
C	7.153196000	17.912279000	4.435422000
H	8.246687000	17.956306000	4.283577000
H	7.021475000	17.496897000	5.446630000
C	6.546058000	16.990121000	3.388582000
H	7.118221000	16.050632000	3.343717000
H	5.516783000	16.717737000	3.681724000
C	6.514784000	17.679063000	2.033610000
H	6.174564000	16.991595000	1.243657000
H	7.538456000	17.991830000	1.760730000
C	5.603191000	18.894783000	2.091975000
H	5.703260000	19.503531000	1.178467000
H	4.550415000	18.557808000	2.112232000
C	5.842818000	19.768245000	3.300474000
C	7.657048000	18.994904000	8.981215000
C	5.166676000	22.072793000	7.479364000
C	4.255547000	23.160979000	7.590516000
C	3.101505000	23.096866000	8.415769000

C	2.894170000	21.889767000	9.162216000
C	3.775415000	20.787539000	9.020705000
C	4.891825000	20.845604000	8.171187000
C	5.393308000	21.879229000	4.425461000
C	7.573791000	19.775030000	6.673696000
C	6.947368000	19.548079000	7.895293000
C	7.640861000	18.130771000	11.181626000
H	8.562644000	18.652549000	11.481890000
H	7.889746000	17.093173000	10.904085000
H	6.955576000	18.133161000	12.033036000
C	9.687609000	20.083557000	5.308935000
H	9.787180000	21.182954000	5.382744000
H	9.073784000	19.918480000	4.412337000
C	8.969138000	19.601805000	6.547664000
C	9.677201000	19.052138000	7.622963000
C	11.856501000	19.565108000	6.434756000
H	11.937265000	20.626398000	6.731501000
H	12.886208000	19.197875000	6.302049000
C	11.068621000	19.470731000	5.137516000
H	10.971619000	18.408839000	4.847163000
H	11.601716000	19.973034000	4.315134000
C	9.009089000	18.717121000	8.804232000
H	9.579570000	18.284893000	9.626306000
C	5.271557000	21.049838000	3.316960000
H	4.705017000	21.379030000	2.444477000
C	11.158944000	18.775702000	7.531557000
H	11.627799000	18.966035000	8.510638000
H	11.296744000	17.694917000	7.343342000
H	4.490435000	24.062897000	7.024187000
H	3.640764000	19.901756000	9.646891000
C	2.153879000	24.255571000	8.471426000
H	1.288009000	24.082131000	7.811401000
H	1.770214000	24.418155000	9.486341000
H	2.653139000	25.178344000	8.145339000
C	1.715293000	21.695063000	10.061111000
H	0.881247000	21.295833000	9.460058000
H	1.959194000	20.964058000	10.843878000
H	1.377724000	22.626068000	10.527547000
N	6.835328000	22.078003000	10.260596000
N	5.961179000	21.533264000	12.344107000
C	8.361156000	21.358470000	11.999259000

C	9.420209000	21.342154000	11.044073000
C	7.044448000	21.687324000	11.517797000
C	8.655454000	21.019342000	13.357256000
C	10.696233000	20.856894000	11.404888000
C	9.153658000	21.815540000	9.738382000
C	9.912496000	20.517860000	13.656057000
C	10.923647000	20.411353000	12.685595000
C	7.887976000	22.186609000	9.405087000
H	11.484791000	20.837067000	10.649203000
H	9.944281000	21.850028000	8.988689000
H	10.123761000	20.237708000	14.689758000
H	11.900566000	20.011697000	12.967255000
H	7.654191000	22.537111000	8.403276000
C	7.721529000	21.231926000	14.489131000
C	7.319763000	20.151831000	15.276928000
C	7.278473000	22.521754000	14.807170000
C	6.449851000	20.349645000	16.347915000
C	6.421395000	22.717583000	15.883600000
C	5.998373000	21.631227000	16.652236000
H	7.666014000	19.151667000	15.010515000
H	7.601695000	23.371258000	14.202039000
H	6.108616000	19.488590000	16.926215000
H	6.078861000	23.727757000	16.119106000
H	5.312349000	21.786700000	17.488529000
C	4.852137000	22.318002000	12.344673000
C	3.763674000	21.875436000	13.142080000
C	4.746856000	23.570293000	11.659390000
C	2.614887000	22.618712000	13.244671000
C	3.493760000	24.259576000	11.741950000
C	2.452562000	23.819181000	12.519632000
H	3.886041000	20.938303000	13.683018000
H	1.809153000	22.259994000	13.889920000
H	3.434113000	25.208233000	11.203734000
H	1.526983000	24.393840000	12.592979000
C	5.883402000	24.500832000	9.867495000
C	5.859169000	24.487305000	11.219237000
C	6.670694000	25.177029000	12.229268000
C	6.125970000	25.557270000	13.466056000
C	8.041126000	25.394464000	12.005690000
C	6.920562000	26.159122000	14.438387000
C	8.834110000	25.986039000	12.982277000

C	8.277670000	26.374638000	14.202608000
H	5.066449000	25.374427000	13.661555000
H	8.478888000	25.070044000	11.058749000
H	6.478257000	26.456075000	15.392769000
H	9.900435000	26.134967000	12.795816000
H	8.903286000	26.834490000	14.971001000
C	6.490269000	25.505891000	8.997066000
C	6.987334000	25.202526000	7.717879000
C	6.536322000	26.852820000	9.411418000
C	7.540457000	26.190536000	6.906995000
C	7.084212000	27.838465000	8.599705000
C	7.596882000	27.512800000	7.342714000
H	6.934748000	24.179778000	7.345404000
H	6.126172000	27.118596000	10.387754000
H	7.923807000	25.920702000	5.919579000
H	7.106990000	28.873940000	8.948906000
H	8.026210000	28.288385000	6.703928000
H	5.829217000	20.598856000	12.807096000
C	4.279369000	18.621658000	12.332113000
O	5.175558000	19.018133000	13.171271000
O	3.626243000	19.376611000	11.613121000
C	4.066808000	17.121877000	12.237553000
H	4.933158000	16.673978000	11.725354000
H	4.007732000	16.665790000	13.235529000
H	3.160966000	16.903153000	11.659449000
H	5.539179000	18.055892000	14.243018000
O	5.658840000	17.349972000	14.999110000
O	7.661149000	16.951719000	14.081751000
C	6.794983000	16.702873000	14.897749000
C	6.935131000	15.613184000	15.930177000
H	6.083940000	14.920031000	15.863721000
H	7.877839000	15.074175000	15.783407000
H	6.910128000	16.055699000	16.937388000

^{Ph}Ent1

Lowest frequency = 22.33 cm^{-1}

Charge = 0, Multiplicity = 1

N	6.235938000	23.793518000	9.953402000
N	7.572965000	24.168478000	8.115941000
C	8.602919000	23.346556000	10.210959000

C	8.318144000	22.759542000	11.487418000
C	7.451612000	23.790069000	9.471342000
C	9.968991000	23.398335000	9.770657000
C	9.351184000	22.156759000	12.245336000
C	6.990832000	22.795545000	11.976791000
C	10.933555000	22.769989000	10.541274000
C	10.634465000	22.137498000	11.762762000
C	6.007639000	23.353012000	11.202575000
H	9.103189000	21.709693000	13.210545000
H	6.764070000	22.378153000	12.960060000
H	11.970520000	22.811760000	10.201350000
H	11.435576000	21.664588000	12.335291000
H	4.974788000	23.422054000	11.558098000
C	10.463978000	24.130442000	8.576864000
C	10.288274000	25.512652000	8.454220000
C	11.226580000	23.457483000	7.611733000
C	10.852764000	26.201633000	7.383400000
C	11.775241000	24.144079000	6.534749000
C	11.591874000	25.521202000	6.419003000
H	9.716044000	26.053086000	9.211590000
H	11.358022000	22.376896000	7.694133000
H	10.717164000	27.282587000	7.301746000
H	12.343812000	23.598521000	5.778513000
H	12.027903000	26.066077000	5.578370000
C	8.041360000	23.314526000	7.138606000
C	8.357386000	21.956688000	7.219417000
C	8.131534000	24.041544000	5.931785000
C	8.807703000	21.336456000	6.062731000
C	8.571115000	23.384729000	4.772571000
C	8.917285000	22.044241000	4.851446000
H	8.262629000	21.406755000	8.157224000
H	9.070258000	20.276332000	6.091596000
H	8.630127000	23.921031000	3.823518000
H	9.264388000	21.522319000	3.956761000
C	7.322052000	25.414214000	7.547014000
C	7.669748000	25.373813000	6.208838000
C	7.594227000	26.479960000	5.247278000
C	8.661301000	26.723442000	4.368792000
C	6.471644000	27.319288000	5.180265000
C	8.610205000	27.775729000	3.457769000
C	6.424330000	28.373194000	4.273596000

C	7.492725000	28.607039000	3.407719000
H	9.548260000	26.089209000	4.425025000
H	5.628075000	27.134512000	5.848230000
H	9.453952000	27.951316000	2.785584000
H	5.540593000	29.014972000	4.237660000
H	7.452345000	29.433383000	2.694028000
C	6.865007000	26.590326000	8.307466000
C	5.675658000	26.592673000	9.050291000
C	7.606006000	27.779163000	8.224042000
C	5.254861000	27.747962000	9.704476000
C	7.182659000	28.931673000	8.877441000
C	6.005716000	28.919274000	9.624379000
H	5.088672000	25.676735000	9.109779000
H	8.517394000	27.793503000	7.625164000
H	4.324235000	27.733010000	10.277046000
H	7.775350000	29.846160000	8.799793000
H	5.671251000	29.823594000	10.138503000

^{Ph}TSrot

Lowest frequency = -30.17 cm^{-1}

Charge = 0, Multiplicity = 1

N	8.707065000	26.093921000	9.680757000
N	8.476081000	24.920694000	7.754699000
C	8.964467000	23.750245000	10.010225000
C	8.266724000	23.909994000	11.255155000
C	8.818384000	24.908091000	9.136344000
C	9.763218000	22.571513000	9.858357000
C	8.028843000	22.774346000	12.067702000
C	7.935897000	25.211333000	11.718946000
C	9.514906000	21.490650000	10.696677000
C	8.588962000	21.560932000	11.749004000
C	8.358971000	26.271785000	10.963221000
H	7.439058000	22.899775000	12.978564000
H	7.452377000	25.342552000	12.688170000
H	10.131589000	20.595732000	10.598795000
H	8.412138000	20.684955000	12.376862000
H	8.334529000	27.304101000	11.324308000
C	11.003319000	22.551464000	9.051530000
C	11.742459000	23.728610000	8.863083000
C	11.513459000	21.355757000	8.518288000

C	12.926487000	23.718269000	8.131920000
C	12.697286000	21.346674000	7.790015000
C	13.406400000	22.530667000	7.585849000
H	11.387294000	24.665420000	9.296114000
H	10.948442000	20.428277000	8.632837000
H	13.477946000	24.650104000	7.988665000
H	13.063610000	20.408522000	7.366399000
H	14.331093000	22.525316000	7.004625000
C	9.061415000	24.390399000	6.589371000
C	9.797269000	23.233260000	6.350448000
C	8.661774000	25.200007000	5.498405000
C	10.205829000	22.949476000	5.051470000
C	9.068526000	24.897538000	4.194312000
C	9.863406000	23.780971000	3.978513000
H	10.027675000	22.530974000	7.138034000
H	10.787332000	22.041691000	4.875228000
H	8.735256000	25.522131000	3.362985000
H	10.190804000	23.529394000	2.967194000
C	7.611542000	25.961735000	7.341967000
C	7.729845000	26.177663000	5.995536000
C	6.957773000	27.111975000	5.162494000
C	7.622503000	27.960889000	4.265598000
C	5.557985000	27.168385000	5.225408000
C	6.909312000	28.847225000	3.461874000
C	4.846981000	28.056470000	4.424020000
C	5.518800000	28.899853000	3.539639000
H	8.713660000	27.933755000	4.215538000
H	5.027508000	26.503861000	5.909979000
H	7.445071000	29.505578000	2.773580000
H	3.756470000	28.086135000	4.487237000
H	4.958616000	29.595464000	2.910282000
C	6.594919000	26.543195000	8.230881000
C	5.751712000	25.703414000	8.970986000
C	6.397594000	27.927700000	8.292221000
C	4.743952000	26.238548000	9.768155000
C	5.387352000	28.459282000	9.086455000
C	4.557523000	27.618439000	9.829239000
H	5.894591000	24.621180000	8.920187000
H	7.055164000	28.583968000	7.719514000
H	4.097318000	25.571009000	10.342851000
H	5.247532000	29.542152000	9.129181000

H	3.766011000	28.039303000	10.453792000

PhEnt2

Lowest frequency = 19.88 cm^{-1}

Charge = 0, Multiplicity = 1

N	8.780174000	26.813656000	10.413728000
N	9.025828000	25.719750000	8.409272000
C	9.427073000	24.474661000	10.502357000
C	9.590274000	24.607512000	11.920380000
C	9.067398000	25.686487000	9.818728000
C	9.651812000	23.188486000	9.899452000
C	10.055624000	23.514754000	12.691341000
C	9.268144000	25.842759000	12.532447000
C	10.125182000	22.162970000	10.701332000
C	10.341812000	22.319083000	12.083872000
C	8.839228000	26.887237000	11.754415000
H	10.180940000	23.645648000	13.768487000
H	9.363120000	25.950311000	13.615074000
H	10.298349000	21.188543000	10.239571000
H	10.709474000	21.473763000	12.669993000
H	8.556211000	27.846955000	12.197946000
C	9.387090000	22.839944000	8.477347000
C	10.430050000	22.349501000	7.679510000
C	8.100856000	22.904392000	7.931573000
C	10.203382000	21.985173000	6.356611000
C	7.871627000	22.529841000	6.610369000
C	8.922991000	22.081414000	5.814943000
H	11.437926000	22.294358000	8.095222000
H	7.266832000	23.246023000	8.545786000
H	11.036086000	21.637507000	5.741537000
H	6.862652000	22.594318000	6.197926000
H	8.743380000	21.807884000	4.772899000
C	10.127959000	25.552530000	7.600531000
C	11.461034000	25.319226000	7.943266000
C	9.703332000	25.687841000	6.259926000
C	12.366993000	25.157208000	6.904976000
C	10.634917000	25.490077000	5.229497000
C	11.954802000	25.223079000	5.561127000
H	11.774240000	25.250644000	8.986518000
H	13.418029000	24.969052000	7.136327000

H	10.322755000	25.546608000	4.184846000
H	12.690836000	25.072391000	4.768055000
C	7.924015000	26.032716000	7.624210000
C	8.299298000	25.993167000	6.291756000
C	7.433633000	26.149714000	5.116095000
C	7.841628000	26.940461000	4.031374000
C	6.190155000	25.503508000	5.035845000
C	7.033292000	27.081549000	2.905563000
C	5.380893000	25.649276000	3.914173000
C	5.799201000	26.437588000	2.841949000
H	8.796890000	27.467257000	4.086203000
H	5.860523000	24.882653000	5.870917000
H	7.368104000	27.706960000	2.074277000
H	4.415885000	25.138000000	3.874062000
H	5.164296000	26.549343000	1.959853000
C	6.610461000	26.362554000	8.190386000
C	6.048136000	25.661863000	9.268103000
C	5.875094000	27.417278000	7.627765000
C	4.786962000	25.993644000	9.754199000
C	4.614152000	27.745732000	8.112276000
C	4.061918000	27.033862000	9.176414000
H	6.598001000	24.845155000	9.738064000
H	6.309778000	27.986059000	6.804498000
H	4.368344000	25.431612000	10.592367000
H	4.060984000	28.571694000	7.659121000
H	3.071894000	27.293235000	9.558755000

^{H}Ent1

Lowest frequency = 23.40 cm^{-1}
Charge = 0, Multiplicity = 1

N	6.438256000	24.348071000	9.771858000
N	7.688161000	24.800308000	7.871263000
C	8.813931000	23.928290000	9.862279000
C	8.683136000	23.443550000	11.196293000
C	7.615113000	24.342285000	9.207093000
C	10.098672000	24.010679000	9.269975000
C	9.844929000	23.010342000	11.884391000
C	7.392923000	23.449747000	11.778844000
C	11.207356000	23.593367000	9.968155000
C	11.077540000	23.080385000	11.279902000

C	6.330507000	23.921329000	11.045660000
H	9.744304000	22.628851000	12.903237000
H	7.251493000	23.096353000	12.802488000
H	12.196292000	23.659533000	9.509448000
H	11.968148000	22.747162000	11.817855000
H	5.325420000	23.958124000	11.477333000
C	7.960685000	23.993009000	6.780400000
C	8.428086000	22.677783000	6.729077000
C	7.652880000	24.729352000	5.612812000
C	8.626238000	22.117672000	5.473776000
C	7.883872000	24.146504000	4.357457000
C	8.367880000	22.847720000	4.299661000
H	8.633947000	22.111973000	7.639077000
H	8.993728000	21.091676000	5.399179000
H	7.683052000	24.709324000	3.443500000
H	8.548003000	22.380848000	3.328687000
C	7.180469000	26.012741000	7.418607000
C	7.160821000	26.013759000	6.037495000
C	6.781019000	27.126602000	5.156447000
C	7.260198000	28.426712000	5.377428000
C	5.930647000	26.906602000	4.062445000
C	6.892194000	29.473273000	4.537986000
C	5.567703000	27.954041000	3.219019000
C	6.045198000	29.242048000	3.454195000
H	7.931767000	28.610347000	6.218627000
H	5.534516000	25.903476000	3.888522000
H	7.276109000	30.478606000	4.727919000
H	4.899934000	27.762902000	2.375313000
H	5.759263000	30.064139000	2.793817000
C	6.799407000	27.080684000	8.348536000
C	5.602752000	27.783972000	8.157081000
C	7.625890000	27.432022000	9.425315000
C	5.245430000	28.816115000	9.018021000
C	7.263452000	28.461761000	10.288354000
C	6.072805000	29.158437000	10.087199000
H	4.950350000	27.507635000	7.326946000
H	8.568638000	26.901837000	9.579307000
H	4.308172000	29.353800000	8.856787000
H	7.919808000	28.725476000	11.121057000
H	5.789204000	29.967543000	10.764376000
H	10.193695000	24.407631000	8.257997000

^{H}TSrot

Lowest frequency = -46.84 cm^{-1}

Charge = 0, Multiplicity = 1

N	6.860146000	25.011852000	9.704754000
N	8.142945000	24.928828000	7.831535000
C	8.733041000	23.557025000	9.945927000
C	8.562578000	23.645321000	11.368569000
C	7.913272000	24.457218000	9.159900000
C	9.601055000	22.546807000	9.470384000
C	9.395584000	22.877934000	12.222199000
C	7.509690000	24.423980000	11.906167000
C	10.364935000	21.779626000	10.321469000
C	10.297930000	21.975801000	11.714263000
C	6.635280000	24.999840000	11.024903000
H	9.272055000	22.993086000	13.301712000
H	7.365870000	24.487776000	12.986201000
H	11.012415000	21.002611000	9.909077000
H	10.923377000	21.381731000	12.384478000
H	5.723728000	25.505949000	11.359352000
C	8.583787000	24.276111000	6.656183000
C	9.624893000	23.381249000	6.393934000
C	7.899978000	24.858138000	5.560180000
C	9.851445000	22.963514000	5.084295000
C	8.140541000	24.438526000	4.248475000
C	9.095692000	23.459005000	4.017255000
H	10.329879000	23.071487000	7.156732000
H	10.667896000	22.263765000	4.892139000
H	7.606503000	24.912353000	3.422252000
H	9.298947000	23.118890000	2.999524000
C	7.354586000	26.030358000	7.410116000
C	7.148675000	25.981247000	6.059312000
C	6.450899000	26.964017000	5.215781000
C	6.753281000	28.331517000	5.283162000
C	5.475145000	26.536738000	4.303897000
C	6.089053000	29.244862000	4.470459000
C	4.812874000	27.452066000	3.489008000
C	5.116173000	28.809776000	3.570845000
H	7.518300000	28.674813000	5.982129000
H	5.223878000	25.474619000	4.250181000

H	6.337168000	30.306875000	4.537201000
H	4.050508000	27.101666000	2.788912000
H	4.596473000	29.528442000	2.932706000
C	7.002054000	27.152412000	8.293598000
C	5.709621000	27.690051000	8.293938000
C	7.993064000	27.766032000	9.071323000
C	5.412126000	28.805515000	9.069199000
C	7.691344000	28.879532000	9.849635000
C	6.399562000	29.404162000	9.852273000
H	4.935066000	27.213676000	7.690580000
H	9.006910000	27.358319000	9.063747000
H	4.397500000	29.211096000	9.064293000
H	8.473597000	29.345280000	10.454056000
H	6.162997000	30.279734000	10.461398000
H	9.614614000	22.313157000	8.414806000

^{H}Ent2

Lowest frequency = 20.43 cm^{-1}

Charge = 0, Multiplicity = 1

N	10.457731000	25.470820000	9.511454000
N	8.945605000	25.389988000	7.758864000
C	8.392091000	24.271817000	9.871332000
C	8.765019000	24.025030000	11.224204000
C	9.294219000	25.054000000	9.086917000
C	7.185804000	23.725286000	9.370042000
C	7.888640000	23.281209000	12.053800000
C	10.018413000	24.514172000	11.665553000
C	6.362743000	22.996885000	10.195601000
C	6.710384000	22.784563000	11.549403000
C	10.822197000	25.189376000	10.779807000
H	8.169534000	23.103857000	13.094608000
H	10.344404000	24.338693000	12.693055000
H	5.433567000	22.577411000	9.804178000
H	6.040121000	22.210926000	12.193898000
H	11.809418000	25.551562000	11.083510000
C	9.606546000	24.918083000	6.641718000
C	10.787860000	24.179806000	6.545351000
C	8.855335000	25.296464000	5.504814000
C	11.218496000	23.832250000	5.272735000
C	9.325883000	24.948822000	4.228507000

C	10.498885000	24.216856000	4.125477000
H	11.357504000	23.914214000	7.436352000
H	12.142458000	23.260519000	5.159123000
H	8.776873000	25.255547000	3.335792000
H	10.877087000	23.938284000	3.139201000
C	7.806594000	26.078481000	7.355300000
C	7.717210000	26.040557000	5.975532000
C	6.706794000	26.699058000	5.136363000
C	6.344305000	28.037665000	5.349903000
C	6.085927000	25.998771000	4.091015000
C	5.382774000	28.650248000	4.552731000
C	5.127836000	26.615123000	3.289403000
C	4.770116000	27.942419000	3.518673000
H	6.831348000	28.599240000	6.149718000
H	6.348613000	24.951933000	3.921283000
H	5.114551000	29.693736000	4.735342000
H	4.652790000	26.051121000	2.482876000
H	4.017698000	28.425814000	2.891081000
C	6.926895000	26.735786000	8.327699000
C	5.533989000	26.625097000	8.209187000
C	7.458856000	27.479777000	9.391269000
C	4.695533000	27.240862000	9.131858000
C	6.616360000	28.088808000	10.316787000
C	5.233229000	27.971225000	10.191524000
H	5.113171000	26.044409000	7.385912000
H	8.541290000	27.591767000	9.482311000
H	3.612446000	27.143547000	9.026942000
H	7.045224000	28.666210000	11.139163000
H	4.573533000	28.450024000	10.918927000
H	6.919970000	23.884257000	8.324234000

$^{i\text{-Pr}}$Ent1

Lowest frequency = 16.32 cm^{-1}

Charge = 0, Multiplicity = 1

N	5.838265000	23.431104000	9.616005000
N	7.195880000	24.078922000	7.882285000
C	8.231122000	23.153708000	9.941068000
C	7.937686000	22.419951000	11.141586000
C	7.069806000	23.570863000	9.201098000

C	9.608150000	23.384435000	9.593777000
C	8.978758000	21.844716000	11.907076000
C	6.591491000	22.288405000	11.558266000
C	10.577164000	22.782377000	10.378225000
C	10.281428000	22.004196000	11.511821000
C	5.594207000	22.839188000	10.798635000
H	8.722938000	21.281037000	12.806880000
H	6.363501000	21.751988000	12.481876000
H	11.626874000	22.941363000	10.121549000
H	11.095158000	21.556131000	12.086805000
H	4.545373000	22.783654000	11.105937000
C	7.594316000	23.295028000	6.822472000
C	7.796484000	21.913937000	6.764595000
C	7.789971000	24.139887000	5.706065000
C	8.202926000	21.380862000	5.549763000
C	8.179304000	23.569241000	4.483231000
C	8.388662000	22.200075000	4.419339000
H	7.640601000	21.282192000	7.640833000
H	8.372718000	20.304949000	5.466987000
H	8.309194000	24.195266000	3.598124000
H	8.696030000	21.744766000	3.475179000
C	7.126293000	25.402201000	7.470358000
C	7.489817000	25.478858000	6.135866000
C	7.549028000	26.693334000	5.313425000
C	8.643044000	26.926181000	4.465983000
C	6.523573000	27.650258000	5.355576000
C	8.710687000	28.079673000	3.688275000
C	6.595424000	28.805028000	4.583355000
C	7.688284000	29.025169000	3.745107000
H	9.459088000	26.200317000	4.436621000
H	5.660665000	27.475931000	6.001405000
H	9.573549000	28.244352000	3.038143000
H	5.786122000	29.537779000	4.631044000
H	7.741750000	29.930911000	3.136479000
C	6.842389000	26.504613000	8.399909000
C	5.703235000	26.513541000	9.216946000
C	7.724754000	27.594188000	8.454086000
C	5.460531000	27.585818000	10.071904000
C	7.477251000	28.663603000	9.308022000
C	6.344449000	28.662156000	10.121515000
H	5.013185000	25.669835000	9.174124000

H	8.610284000	27.594911000	7.815553000
H	4.567661000	27.581501000	10.701755000
H	8.175417000	29.503323000	9.339967000
H	6.150469000	29.501775000	10.793307000
C	10.109901000	24.318004000	8.500640000
C	10.802696000	25.531062000	9.128190000
H	10.131565000	26.054474000	9.825532000
H	11.712439000	25.246099000	9.680063000
H	11.097187000	26.244591000	8.342616000
C	11.013654000	23.609570000	7.493103000
H	11.307398000	24.311274000	6.697489000
H	11.938312000	23.232290000	7.958475000
H	10.495167000	22.764270000	7.017966000
H	9.264137000	24.714765000	7.938707000

$^{i\text{-Pr}}$TSrot

Lowest frequency = -38.33 cm^{-1}

Charge = 0, Multiplicity = 1

N	8.930275000	26.026608000	9.563499000
N	8.542771000	24.878269000	7.641109000
C	8.880113000	23.665105000	9.887725000
C	8.176640000	23.903108000	11.114132000
C	8.914763000	24.836468000	9.024095000
C	9.500567000	22.390744000	9.722282000
C	7.737866000	22.793724000	11.879400000
C	8.039125000	25.234473000	11.590900000
C	9.041261000	21.332878000	10.489874000
C	8.096435000	21.517710000	11.516904000
C	8.609609000	26.236096000	10.850176000
H	7.147705000	22.979128000	12.779799000
H	7.559731000	25.426613000	12.552193000
H	9.489257000	20.345484000	10.359855000
H	7.744839000	20.659023000	12.093455000
H	8.712112000	27.260146000	11.220186000
C	9.101009000	24.391288000	6.448343000
C	9.864019000	23.262027000	6.152704000
C	8.644849000	25.215978000	5.388697000
C	10.229985000	23.016878000	4.832929000
C	9.010384000	24.950552000	4.063939000
C	9.823416000	23.860381000	3.792147000

H	10.132464000	22.540655000	6.908519000
H	10.824102000	22.127304000	4.610918000
H	8.632056000	25.585351000	3.260080000
H	10.116958000	23.638322000	2.763804000
C	7.659968000	25.920435000	7.286353000
C	7.721521000	26.169132000	5.940792000
C	6.911443000	27.119666000	5.164485000
C	7.531991000	27.989724000	4.256105000
C	5.516084000	27.172821000	5.294790000
C	6.780881000	28.891079000	3.505388000
C	4.767296000	28.076552000	4.547065000
C	5.395511000	28.939337000	3.649462000
H	8.619708000	27.967344000	4.155031000
H	5.018877000	26.492616000	5.988954000
H	7.283092000	29.564967000	2.806904000
H	3.680990000	28.102942000	4.662599000
H	4.805495000	29.646709000	3.061880000
C	6.693660000	26.488463000	8.237770000
C	5.880794000	25.639717000	9.000430000
C	6.519606000	27.873387000	8.345498000
C	4.922622000	26.166194000	9.862726000
C	5.559690000	28.396150000	9.205029000
C	4.758410000	27.545816000	9.968405000
H	6.003969000	24.557626000	8.913518000
H	7.155383000	28.535929000	7.755612000
H	4.298336000	25.491341000	10.453347000
H	5.436979000	29.479089000	9.283049000
H	4.006311000	27.959853000	10.644143000
C	10.859457000	22.285830000	9.056366000
C	11.920255000	22.542561000	10.135729000
H	11.761984000	23.515101000	10.624536000
H	11.882619000	21.763967000	10.913471000
H	12.928304000	22.540418000	9.691843000
C	11.128547000	20.960868000	8.349702000
H	12.073550000	21.011832000	7.788083000
H	11.228536000	20.136549000	9.072120000
H	10.326258000	20.687033000	7.648231000
H	10.965571000	23.113475000	8.342733000

$^{i\text{-Pr}}$Ent2

Lowest frequency = 15.94 cm^{-1}

Charge = 0, Multiplicity = 1

N	9.103505000	26.645128000	10.186318000
N	8.936307000	25.515686000	8.196591000
C	9.375187000	24.230825000	10.261526000
C	9.841678000	24.364528000	11.612617000
C	9.118335000	25.479837000	9.597927000
C	9.210083000	22.912745000	9.710038000
C	10.279264000	23.227247000	12.331757000
C	9.829144000	25.644559000	12.217928000
C	9.668633000	21.843831000	10.460375000
C	10.224009000	21.988689000	11.745647000
C	9.400149000	26.727531000	11.494754000
H	10.651350000	23.356797000	13.350445000
H	10.150256000	25.753736000	13.256100000
H	9.564398000	20.835842000	10.052563000
H	10.571304000	21.104511000	12.285289000
H	9.329825000	27.722347000	11.945443000
C	9.929567000	25.204450000	7.292291000
C	11.227825000	24.738575000	7.513432000
C	9.425010000	25.455974000	5.995764000
C	12.012714000	24.485329000	6.396858000
C	10.232593000	25.167254000	4.884086000
C	11.515572000	24.684690000	5.094991000
H	11.605125000	24.569868000	8.523271000
H	13.032643000	24.118352000	6.532199000
H	9.853426000	25.322457000	3.871934000
H	12.155296000	24.459976000	4.238593000
C	7.831768000	26.002459000	7.513423000
C	8.087101000	25.960022000	6.153710000
C	7.158471000	26.301012000	5.067484000
C	7.581341000	27.099661000	3.994440000
C	5.836423000	25.830808000	5.068086000
C	6.708647000	27.421968000	2.957634000
C	4.963817000	26.158507000	4.035442000
C	5.395753000	26.954868000	2.974851000
H	8.602543000	27.487646000	3.988901000
H	5.497983000	25.197894000	5.891143000
H	7.055865000	28.049937000	2.133426000
H	3.937870000	25.782734000	4.055205000
H	4.710350000	27.209085000	2.162902000

C	6.617154000	26.439271000	8.210106000
C	6.055787000	25.682175000	9.247995000
C	5.982736000	27.628043000	7.824264000
C	4.888959000	26.100792000	9.880075000
C	4.814835000	28.043059000	8.455143000
C	4.263093000	27.281973000	9.485213000
H	6.530399000	24.748766000	9.555605000
H	6.420083000	28.228562000	7.024796000
H	4.464009000	25.496382000	10.684891000
H	4.334560000	28.973500000	8.143412000
H	3.346909000	27.610551000	9.981465000
C	8.451288000	22.580698000	8.430361000
C	9.282624000	21.773275000	7.434656000
H	10.208434000	22.299083000	7.164315000
H	9.550910000	20.782714000	7.835642000
H	8.707022000	21.610711000	6.510826000
C	7.150048000	21.849330000	8.776740000
H	6.550900000	21.691188000	7.866675000
H	7.345407000	20.863176000	9.226434000
H	6.537846000	22.422542000	9.489483000
H	8.162107000	23.509766000	7.932007000

8. References

(1) Li, J.; Wang, G.; Guo, W.; Jiang, J.; Wang, J. H8-BINOL-derived chiral η^6-benzene ligands: New opportunities for the ruthe-nium-catalyzed asymmetric C–H activation. *Angew. Chem., Int. Ed.* **2024**, *63*, No. e202405782.

(2) Bi, W.-Z.; Sun, K.; Qu, C.; Chen, X.-L.; Qu, L.-B.; Zhu, S.-H.; Li, X.; Wu, H.-T.; Duan, L.-K.; Zhao, Y.-F. A direct metal-free C2–H functionalization of quinoline N-oxides: a highly selective amination and alkylation strategy toward 2-substituted quinolines. *Org. Chem. Front.* **2017**, *4*, 1595–1600.

(3) Sun, L.-C.; Chen, H.-H.; Liu, B.-X.; Chang, J.-B.; Kong, L.-H.; Wang, F.; Lan, Y.; Li, X.-W. Rhodium-catalyzed atroposelective construction of indoles via C–H bond activation. *Angew. Chem., Int. Ed.* **2021**, *60*, 8391–8395.

(4) Huang, Y.-Q.; Wu, Z.-J.; Zhu, L.; Gu, Q.; Lu, X.; You, S.-L.; Mei, T.-S. Electrochemical rhodium-catalyzed enantioselec-tive C–H annulation with alkynes. *CCS Chem.* **2022**, *4*, 3181–3189.

(5) Yang, W; Sun, W; Zhang, C; Wang, Q; Guo, Z; Mao, B; Liao, J; Guo, H. Lewis-base-catalyzed asymmetric [3+3] annulation reaction of morita-baylis-hillman carbonates: Enantioselective synthesis of spirocyclohexenes. *ACS Catal.* **2017**, *7*, 3142-3146.

(6) Shen, S.-J.; Du, X.-L.; Xu, X.-L.; Zhao, M.-G.; Liang, J.-Y. Regioselective N-addition/substitution reaction of α-alkylidene pyrazolinones with propargyl sulfonium salts to construct allylthio-containing pyrazolones. *J. Org. Chem.* **2019**, *84*, 12520–12531.

(7) Neese, F. The ORCA Program System. *Wiley Interdiscip. Rev.: Comput. Mol. Sci.* **2012**, *2 (1)*, 73–78.

(8) Neese, F. Software Update: The ORCA Program System─Version 6.0. *Wiley Interdiscip. Rev.: Comput. Mol. Sci.* **2025**, *15 (2)*, e70019.

(9) Adamo, C.; Barone, V. Toward reliable density functional methods without adjustable parameters: The PBE0 model. *J. Chem. Phys.* **1999**, *110*, 6158–6170

(10) Ernzerhof, M.; Scuseria, G. E. Assessment of the Perdew–Burke–Ernzerhof exchange-correlation functional. *J. Chem. Phys.* **1999**, *110*, 5029–5036.

(11) Grimme, S.; Antony, J.; Ehrlich, S.; Krieg, H. A consistent and accurate ab initio parametrization of density functional dispersion correction (DFT-D) for the 94 elements H-Pu. *J. Chem. Phys.* **2010**, *132*, 154104.

(12) Grimme, S.; Ehrlich, S.; Goerigk, L. Effect of the damping function in dispersion corrected density functional theory. *J. Comput. Chem.* **2011**, *32*, 1456–1465.

(13) Weigend, F. Accurate Coulomb-fitting basis sets for H to Rn. *Phys. Chem. Chem. Phys.* **2006**, *8*, 1057−1065.

(14) Weigend, F.; Ahlrichs, R. Balanced basis sets of split valence, triple zeta valence and quadruple zeta valence quality for H to Rn: Design and assessment of accuracy. *Phys. Chem. Chem. Phys.* **2005**, *7*, 3297–3305.

(15) Schäfer, A.; Huber, C.; Ahlrichs, R. Fully optimized contracted Gaussian basis sets of triple zeta valence quality for atoms Li to Kr. *J. Chem. Phys.* **1994**, *100*, 5829−5835.

(16) Martin, J. M. L.; Sundermann, A. Correlation consistent valence basis sets for use with the Stuttgart–Dresden–Bonn relativistic effective core potentials: The atoms Ga–Kr and In–Xe. *J. Chem. Phys.* **2001**, *114*, 3408–3420.

(17) Dolg, M.; Wedig, U.; Stoll, H.; Preuss, H. Energy-adjusted ab initio pseudopotentials for the first row transition elements. *J. Chem. Phys.* **1987**, *86*, 866–872.

(18) Marenich, A. V.; Cramer, C. J.; Truhlar, D. G. Universal solvation model based on solute electron density and on a continuum model of the solvent defined by the bulk dielectric constant and atomic surface tensions. *J. Phys. Chem. B* **2009**, *113*, 6378–6396.

(19) Zhang, X.; Paton, R. S. Stereoretention in styrene heterodimerisation promoted by one-electron oxidants. *Chem. Sci.* **2020**, *11*, 9309–9324.

(20) Goswami, N.; Kumar, N.; Gupta, P.; Maiti, D. Surpassing the Limited Coordination Affinity of Native Amides by Introducing Pyridone-Pd-AgOAc Clusters to Promote Distal γ-C(sp^3)–H Arylation. *ACS Catal.* **2024**, *14 (6)*, 3798–3811.

(21) Fan, Q.-K.; Bai, Z.-Q.; He, G.; Chen, G.; Wang, H. Iridium-Catalyzed Nitrene-Mediated Enantioselective 1,2-Hydride Shift Enabled by Attractive Noncovalent Interactions for Chiral δ-Lactam Synthesis. *J. Am. Chem. Soc.* **2025**, *147*, 20680–20692.

(22) Eberson, L.; Hartshorn, M. P.; Persson, O.; Radner, F. Making Radical Cations Live Longer. *Chem. Commun.* **1996**, 2105–2112.

(23) Gu, X.; Song, X.; Shao, C.; Zeng, P.; Lu, X.; Shen, X.; Yang, Q. Electrospinning of Poly(Butylene-Carbonate): Effect of Solvents on the Properties of the Nanofibers Film. *Int. J. Electrochem. Sci.* **2014**, *9 (12)*, 8045–8056.

(24) Carraro, M.; Gardan, M.; Sartorel, A.; Maccato, C.; Bonchio, M. Hydrogen Peroxide Activation by Fluorophilic Polyoxotungstates for Fast and Selective Oxygen Transfer Catalysis. *Dalton Trans.* **2016**, *45 (37)*, 14544–14548.

(25) Sigma-Aldrich. 1,1,1,3,3,3-Hexafluoro-2-propanol https://www.sigmaaldrich.com/catalog/product/aldrich/105228 (2017).

(26) Parrish Jr., J. R.; Blout, E. R. Spectroscopic Studies of Random Chain and α-Helical Polypeptides in Hexafluoroisopropanol. *Biopolymers* **1971**, *10 (9)*, 1491–1512.

(27) Weast, R. C. *CRC Handbook of Chemistry and Physics,* 76th ed.; CRC Press: Boca Raton, FL, 1995.

(28) Richmond, E.; Yi, J.; D. Vuković, V.; Sajadi, F.; N. Rowley, C.; Moran, J. Ring-Opening Hydroarylation of Monosubstituted Cyclopropanes Enabled by Hexafluoroisopropanol. *Chem. Sci.* **2018**, *9 (30)*, 6411–6416.

(29) Contreras-García, J.; Johnson, E. R.; Keinan, S.; Chaudret, R.; Piquemal, J. P.; Beratan, D. N.; Yang, W. NCIPLOT: a program for plotting non-covalent interaction regions. *J. Chem. Theory Comput.* **2011**, *7*, 625–632.

(30) Humphrey, W.; Dalke, A.; Schulten, K. VMD - Visual Molecular Dynamics. *J. Molec. Graphics* **1996**, *14 (1)*, 33–38. http://www.ks.uiuc.edu/Research/vmd/

9. NMR Spectra

8-phenyl-*N*-(*o*-tolyl)isoquinolin-1-amine (1b)

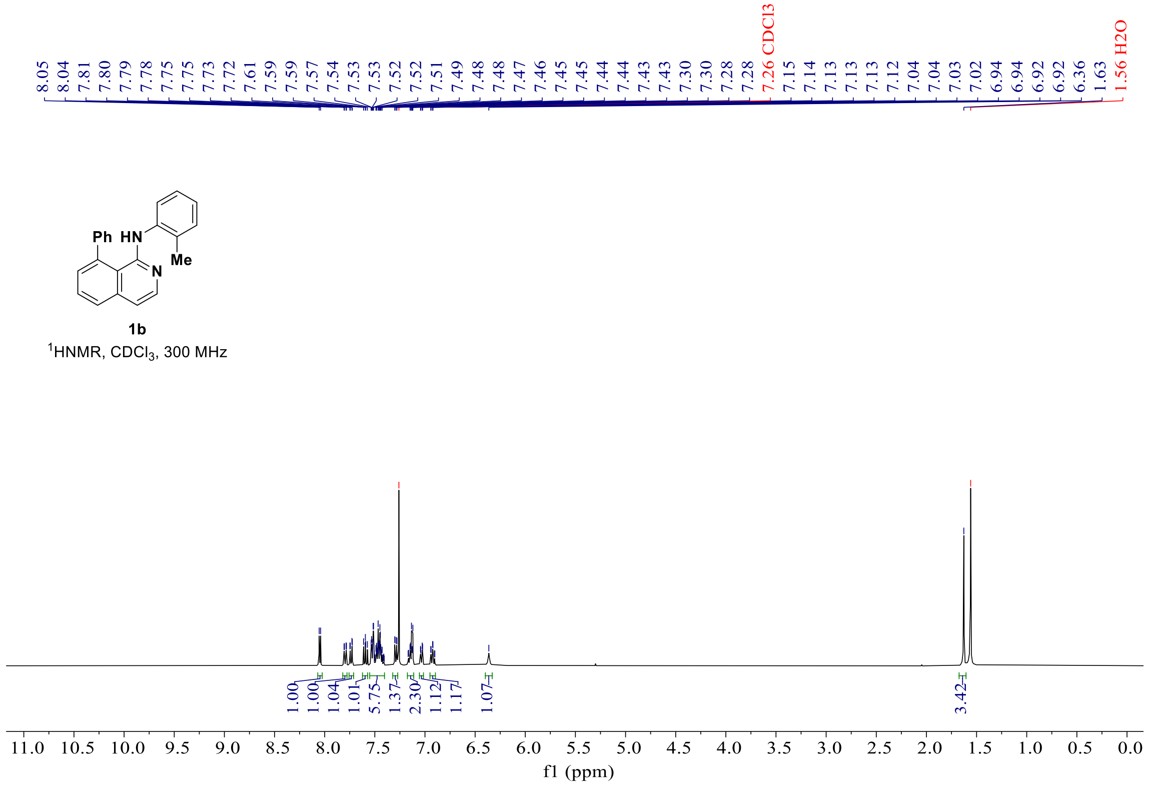

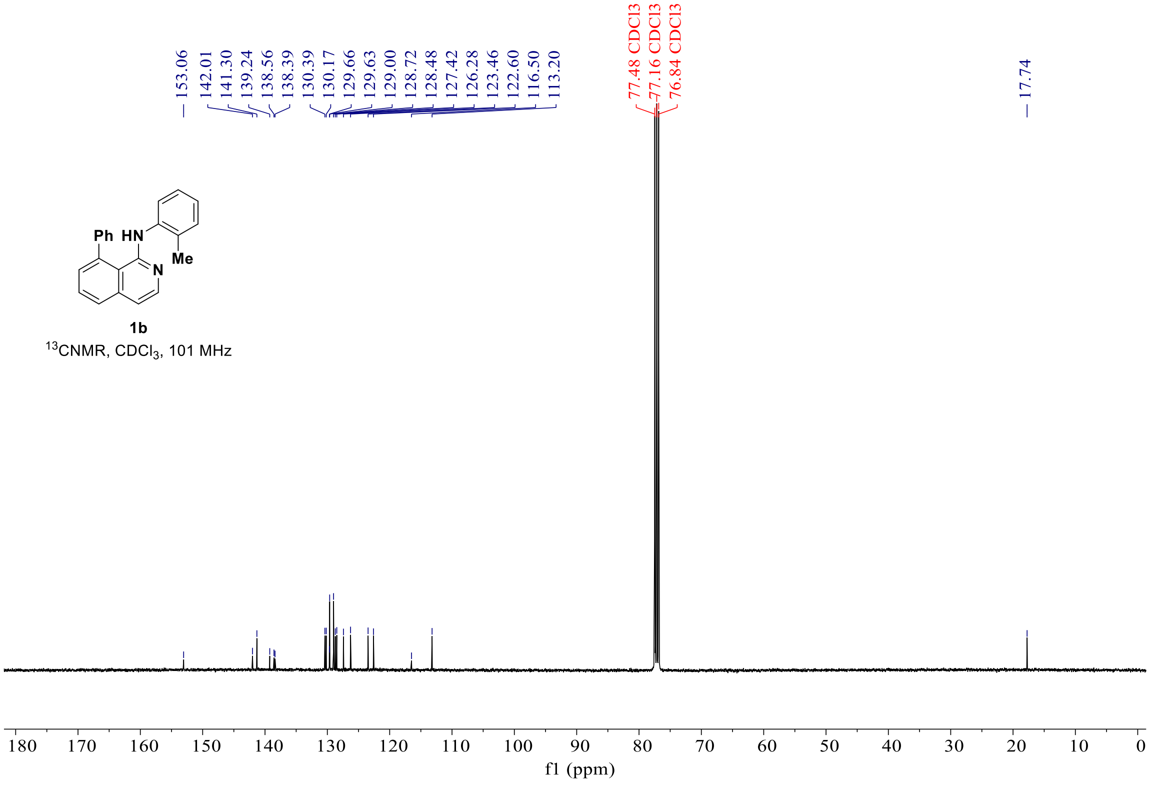

N-(2-fluorophenyl)-8-phenylisoquinolin-1-amine (1c)

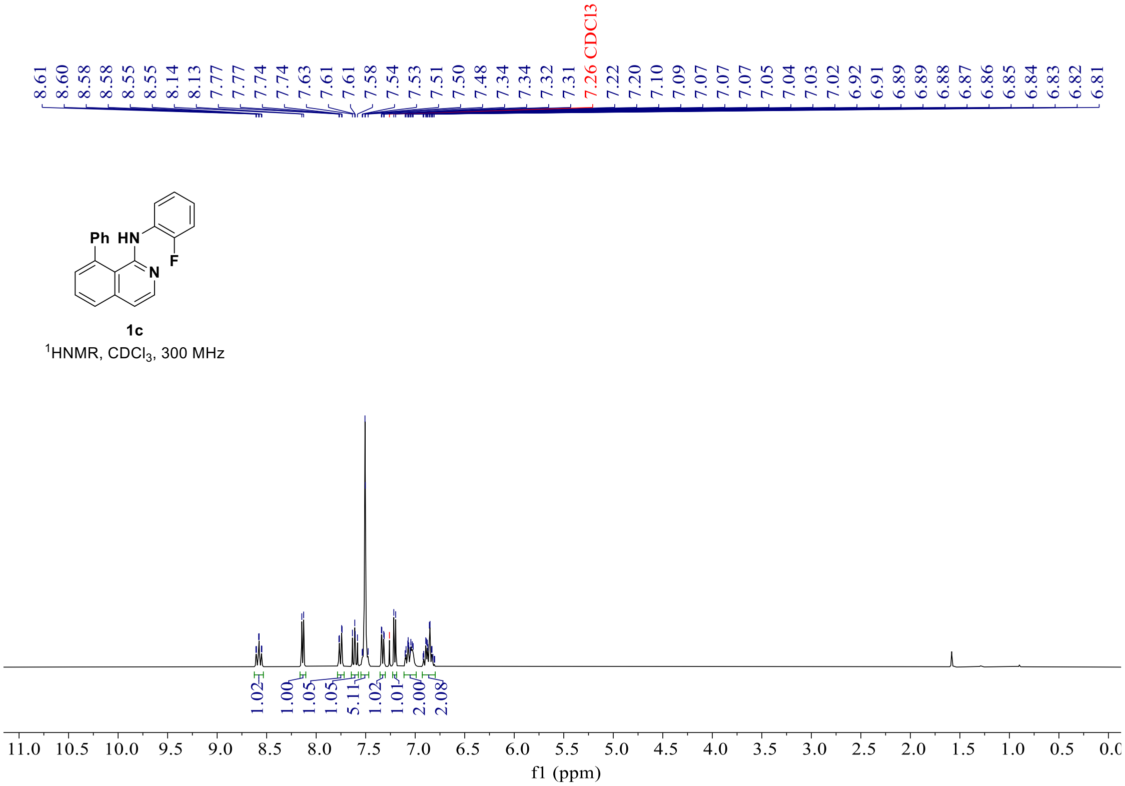

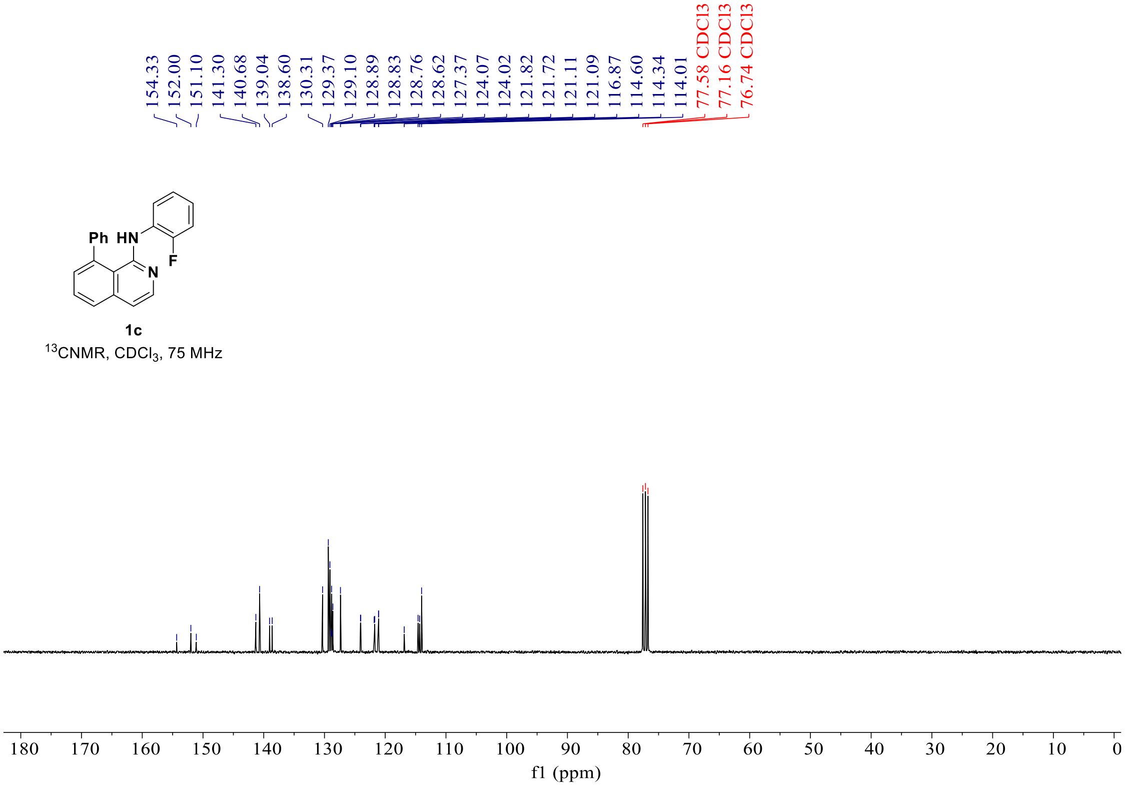

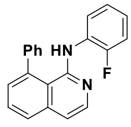

1c

^{19}FNMR, $CDCl_3$, 377 MHz

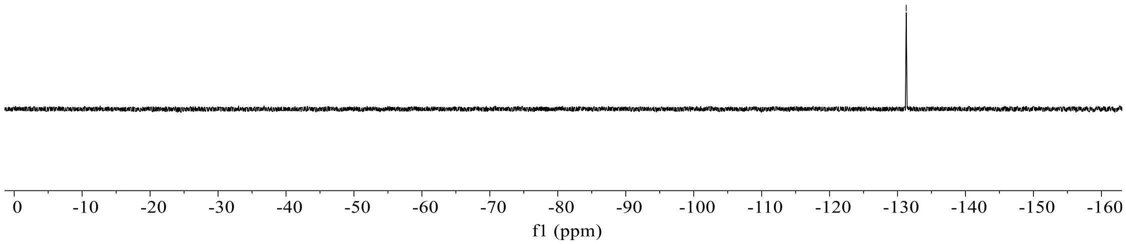

8-phenyl-*N*-(*m*-tolyl)isoquinolin-1-amine (1d)

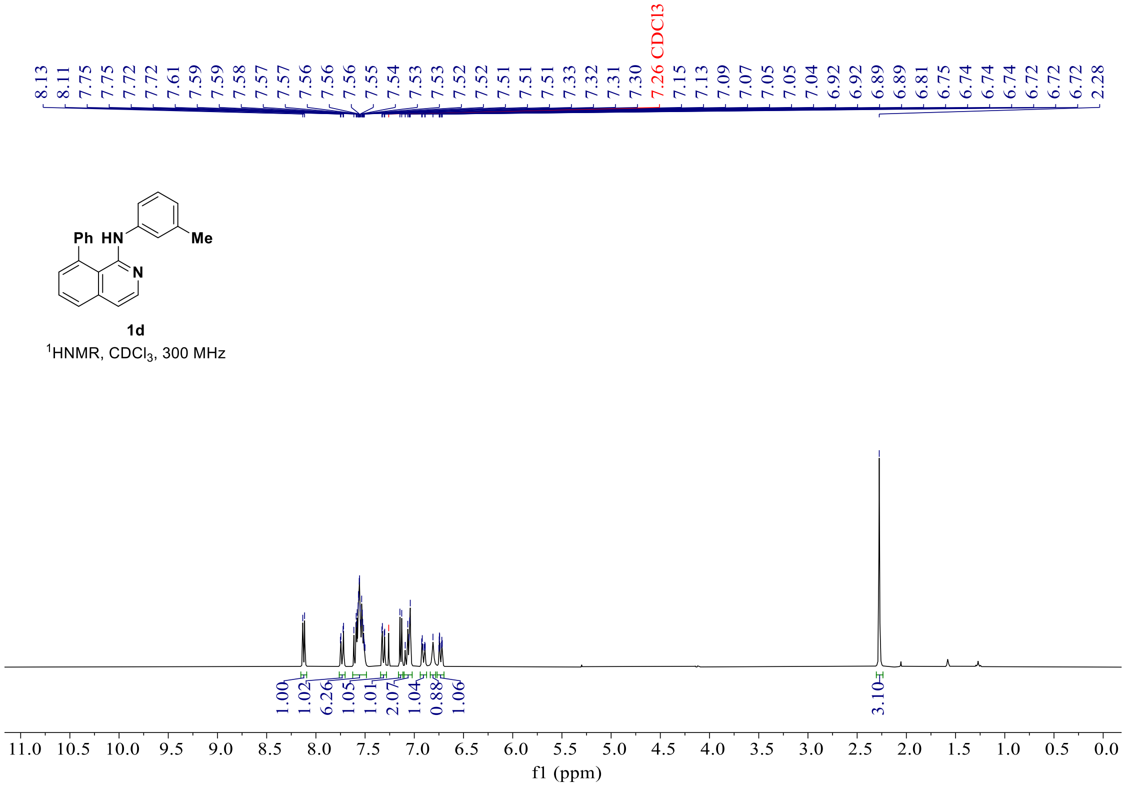

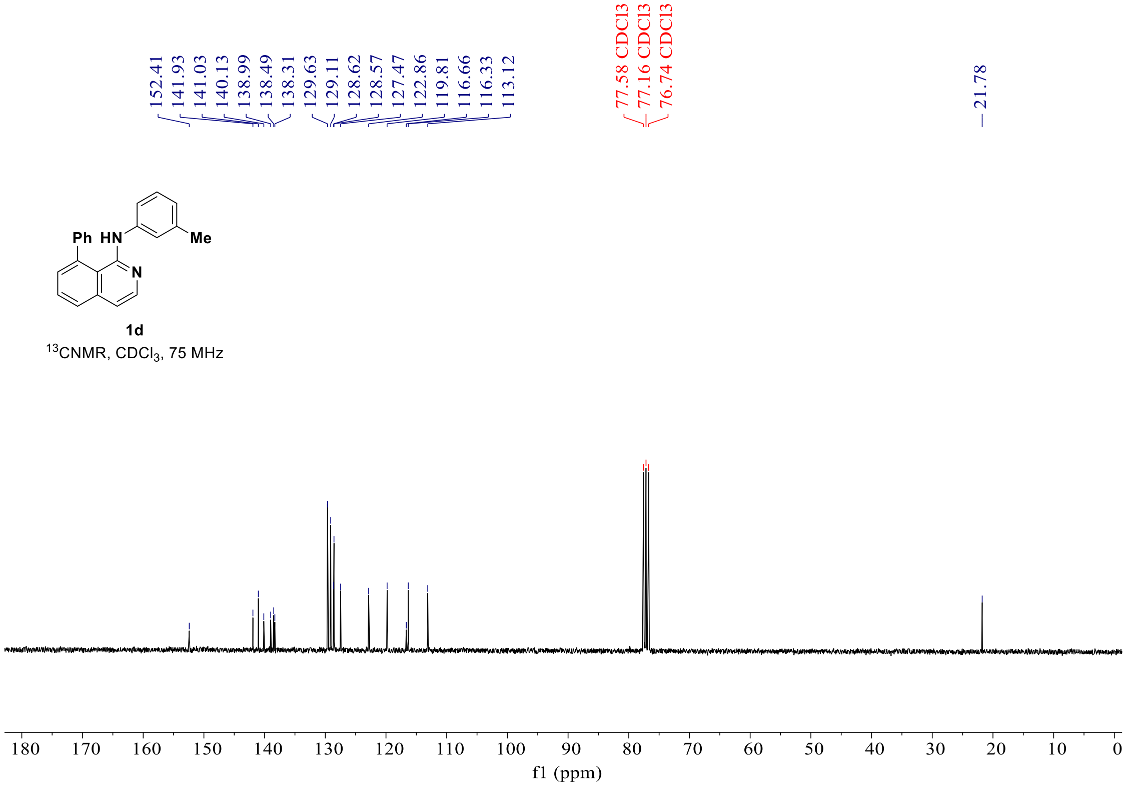

Methyl 3-((8-phenylisoquinolin-1-yl)amino)benzoate (1e)

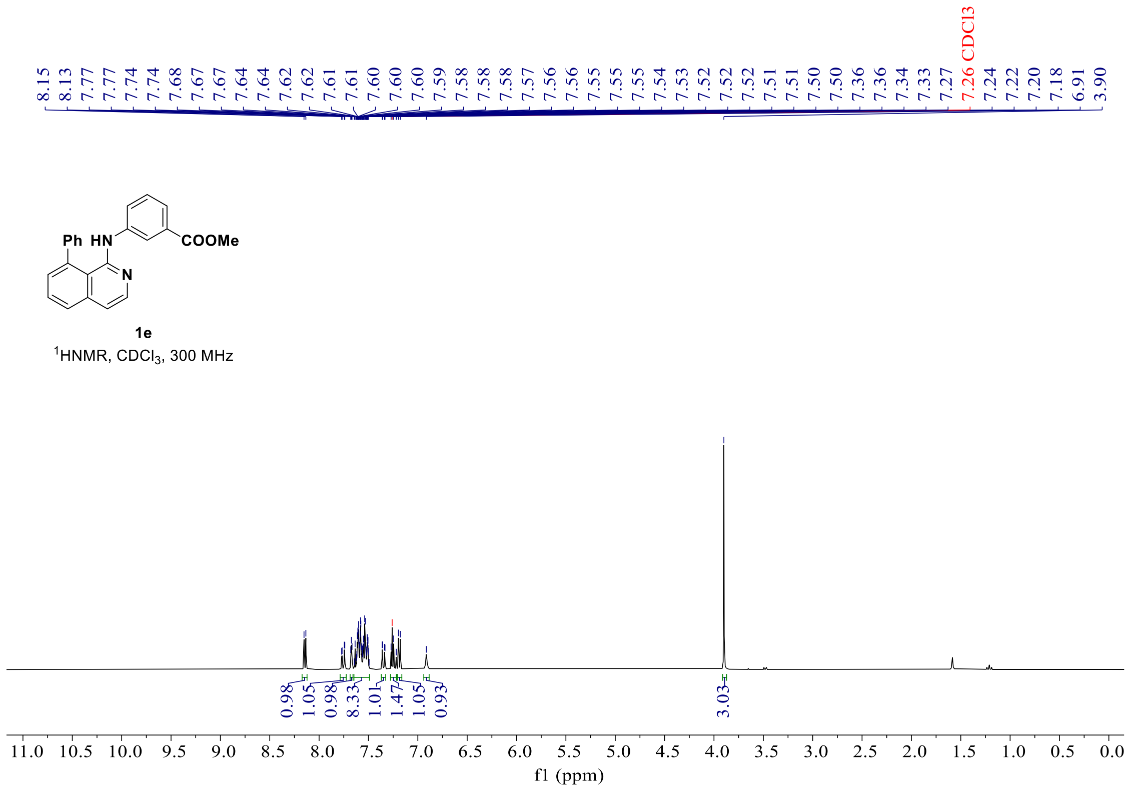

1e

^{1}HNMR, $CDCl_3$, 300 MHz

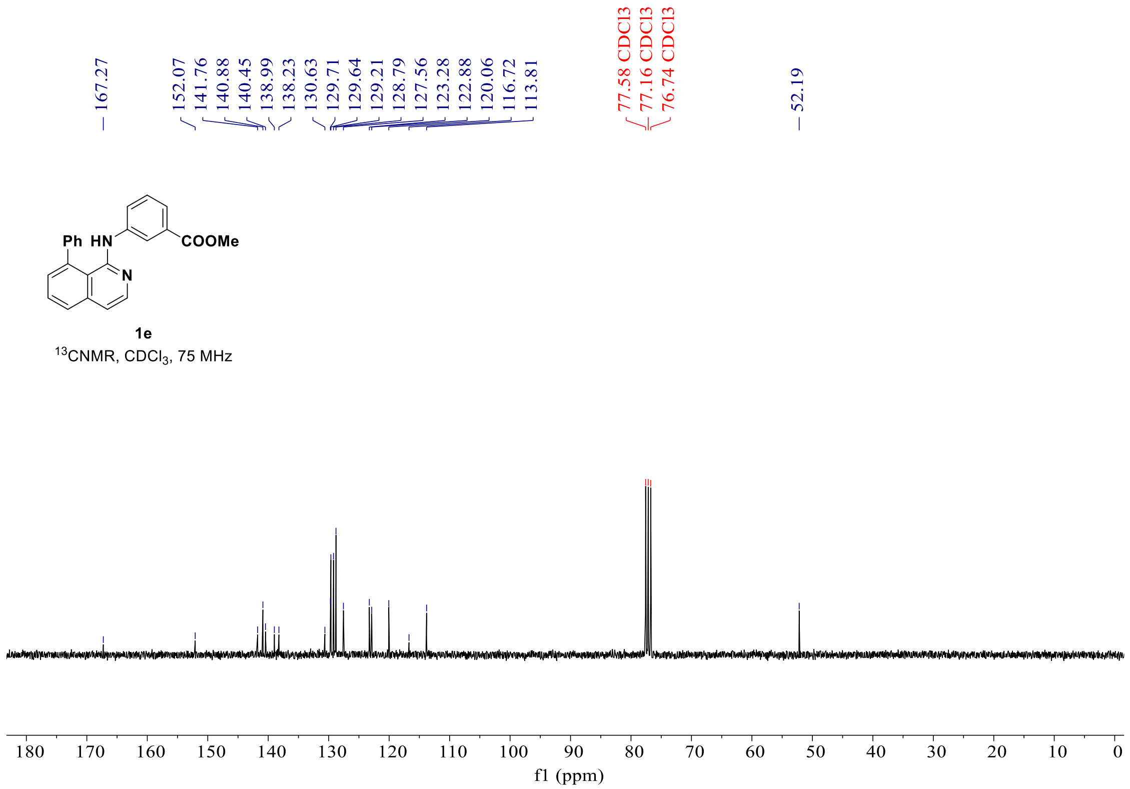

1e

^{13}CNMR, $CDCl_3$, 75 MHz

N-(3-chlorophenyl)-8-phenylisoquinolin-1-amine (1f)

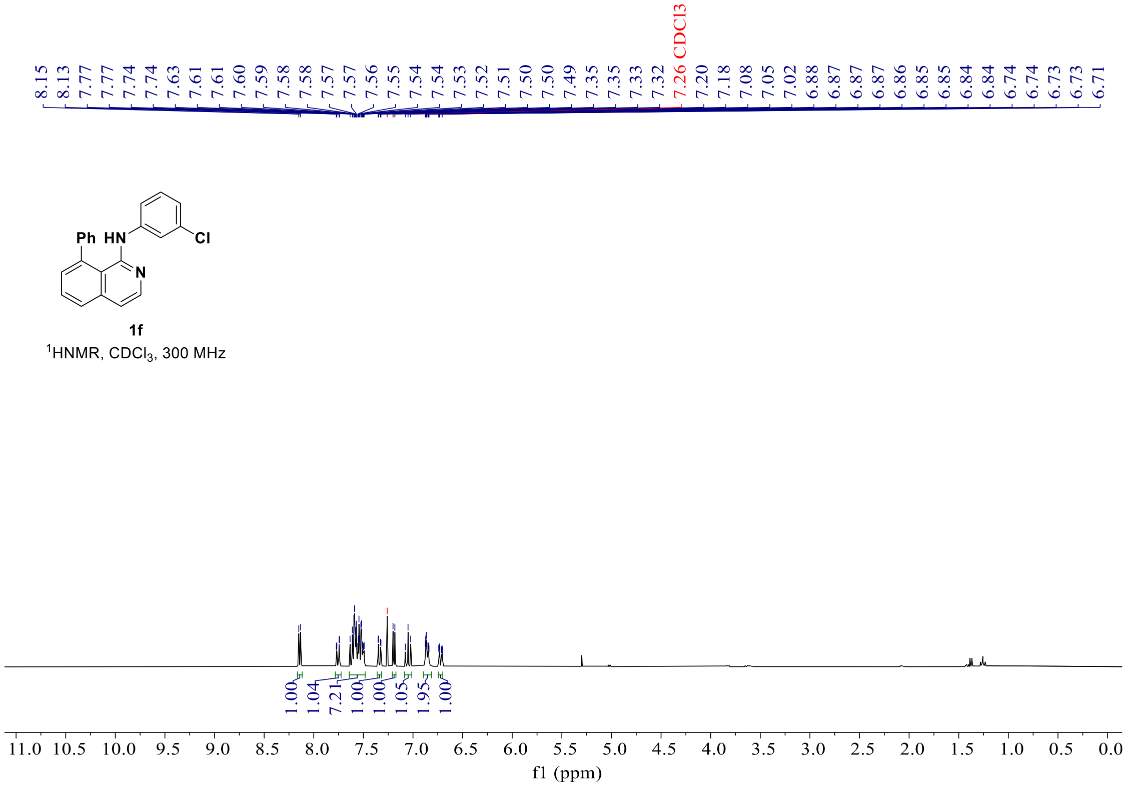

1f

^{1}HNMR, $CDCl_3$, 300 MHz

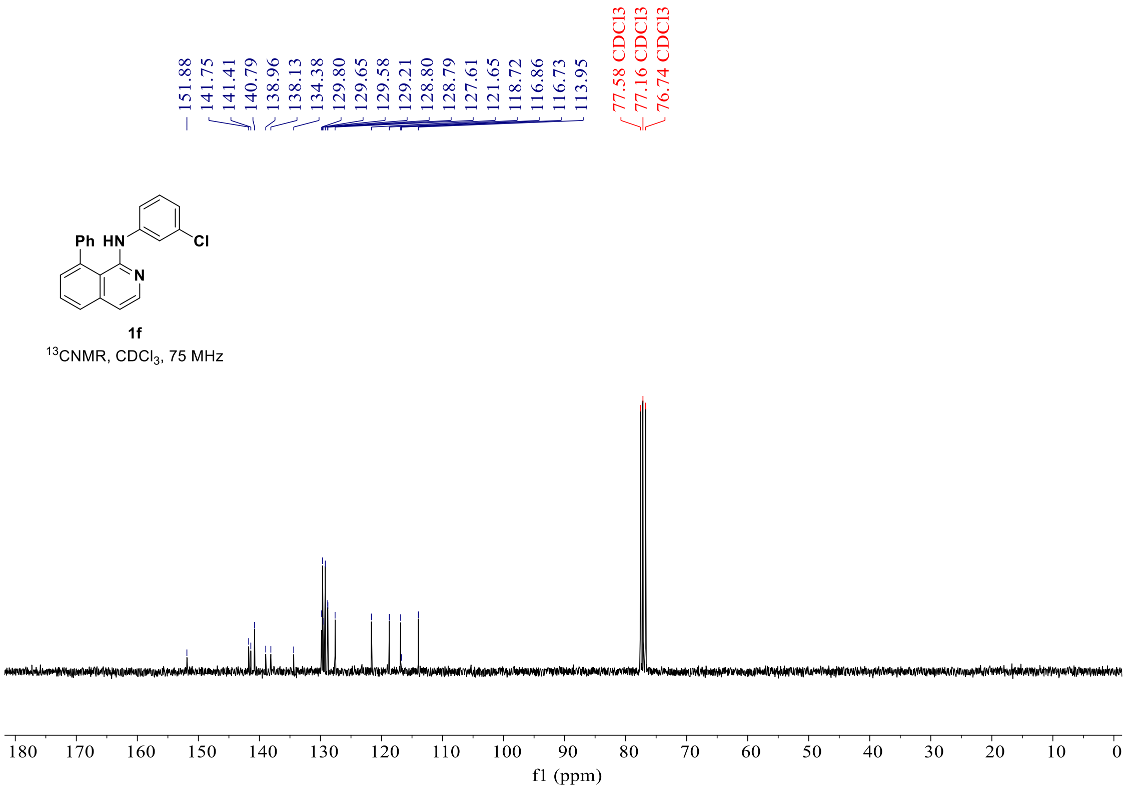

1f

^{13}CNMR, $CDCl_3$, 75 MHz

N-(4-methoxyphenyl)-8-phenylisoquinolin-1-amine (1g)

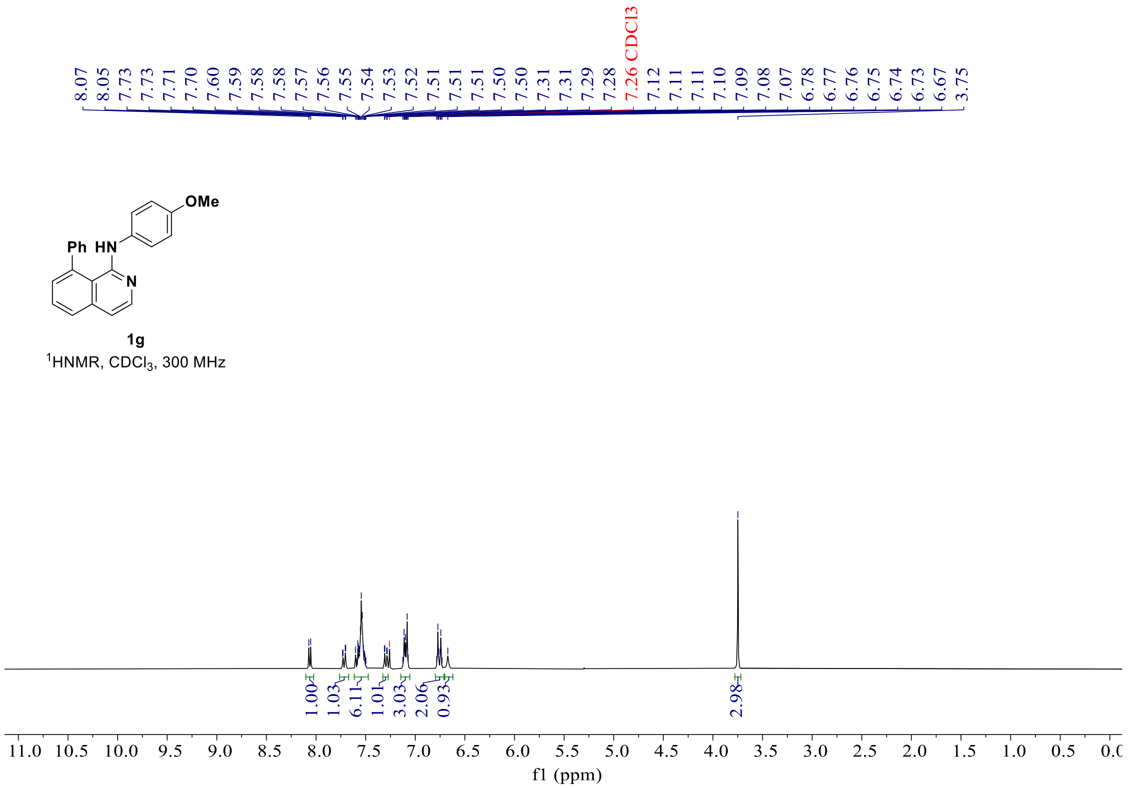

1g

^{1}HNMR, $CDCl_3$, 300 MHz

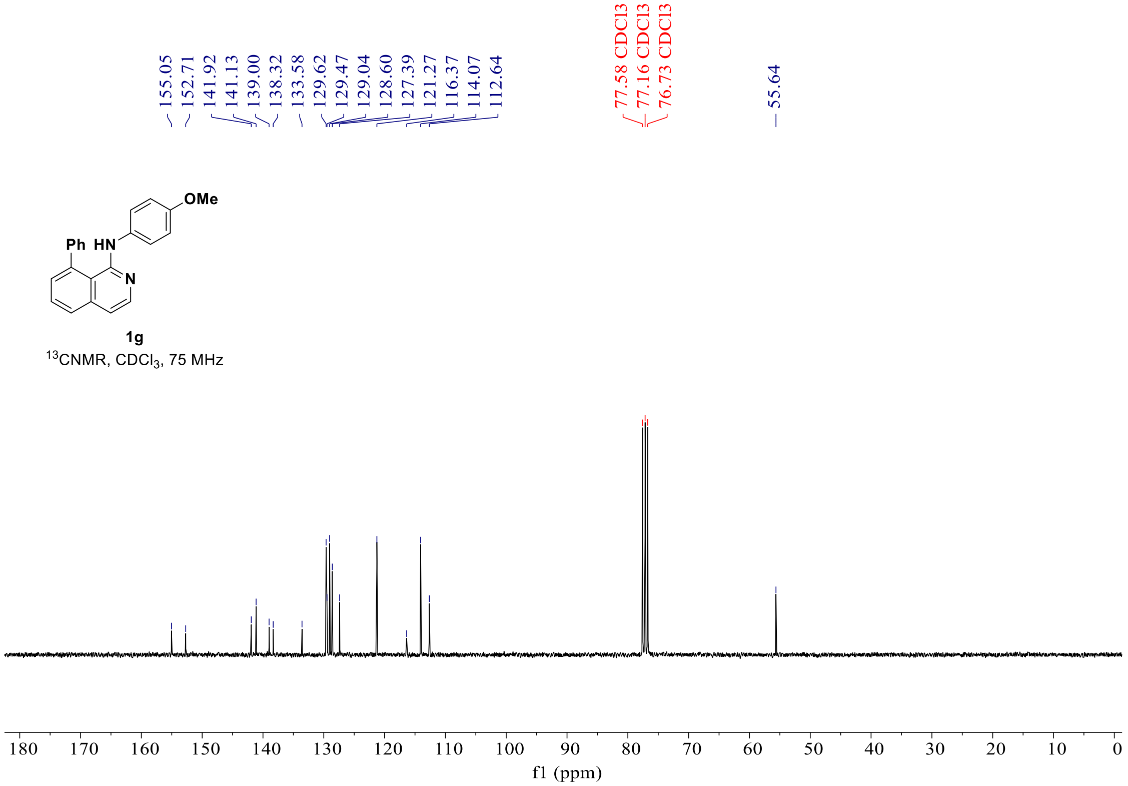

1g

^{13}CNMR, $CDCl_3$, 75 MHz

8-phenyl-*N*-(4-(trifluoromethyl)phenyl)isoquinolin-1-amine (1h)

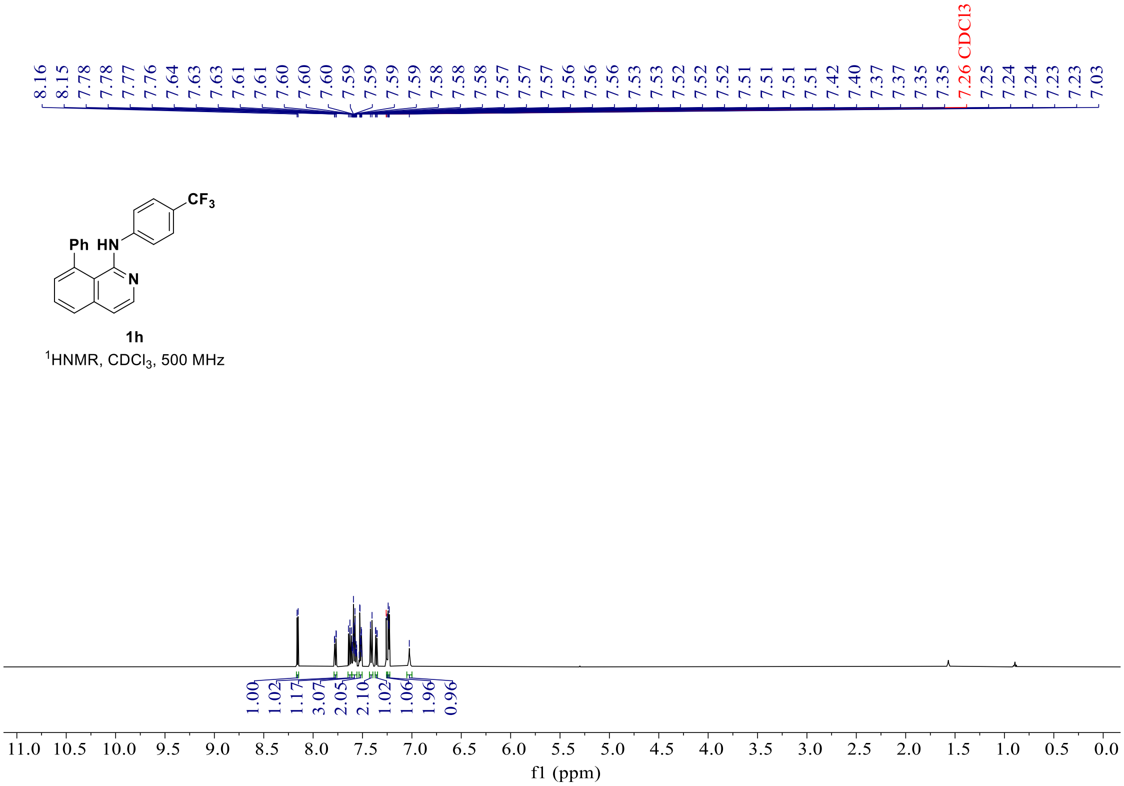

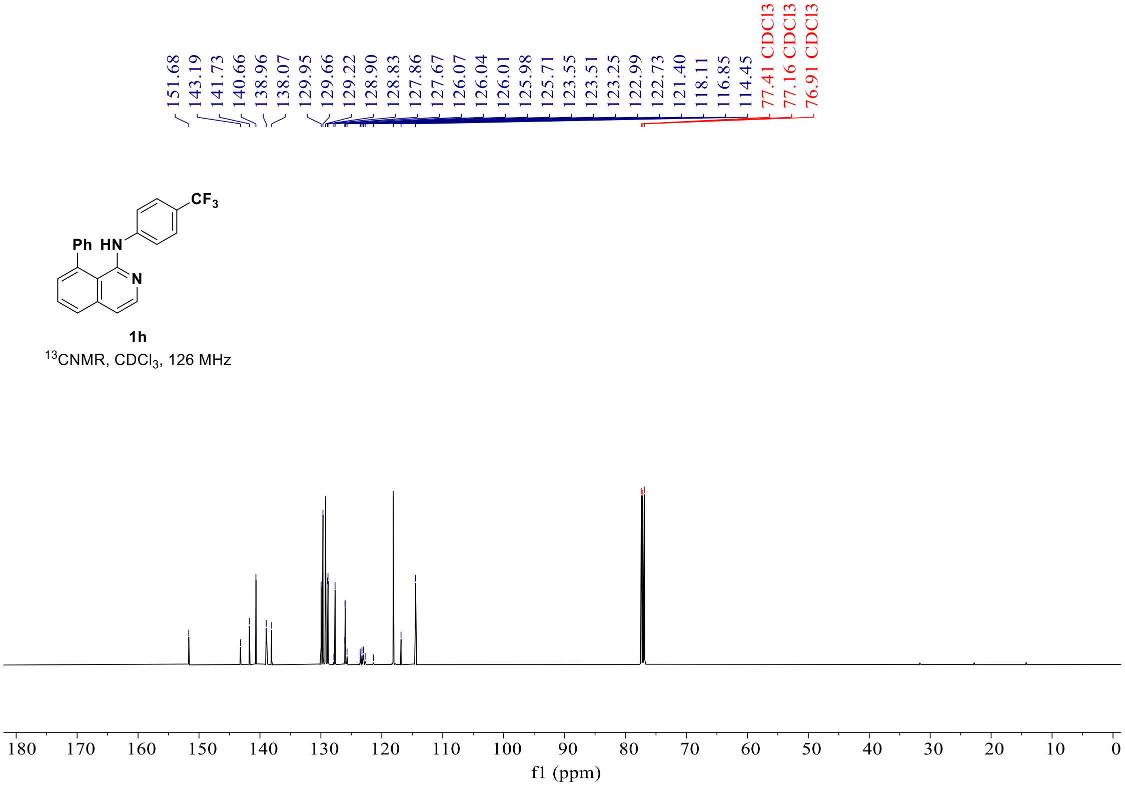

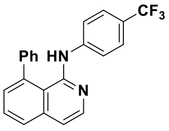

1h

^{19}FNMR, $CDCl_3$, 471 MHz

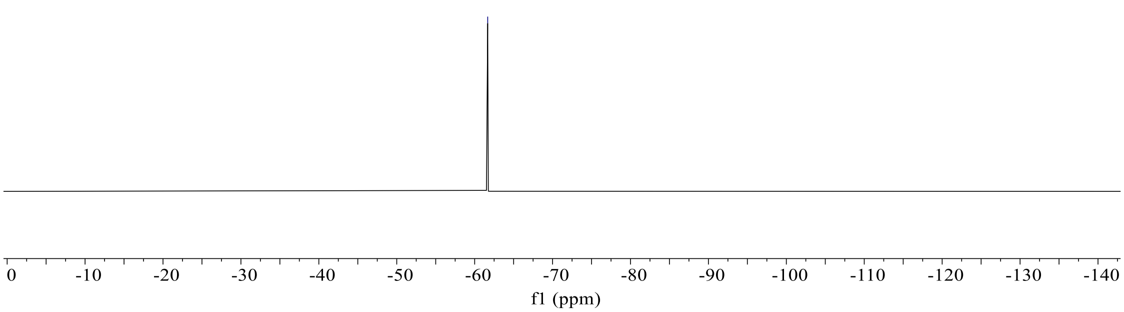

N-(4-bromophenyl)-8-phenylisoquinolin-1-amine (1l)

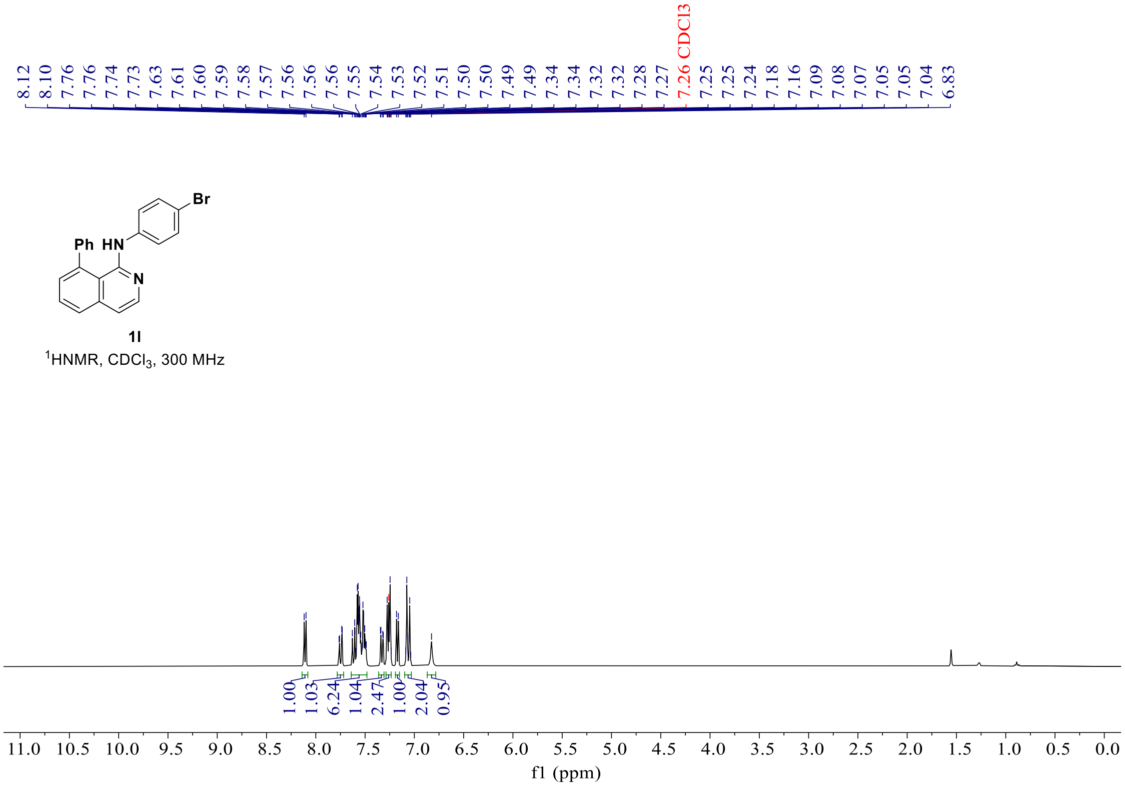

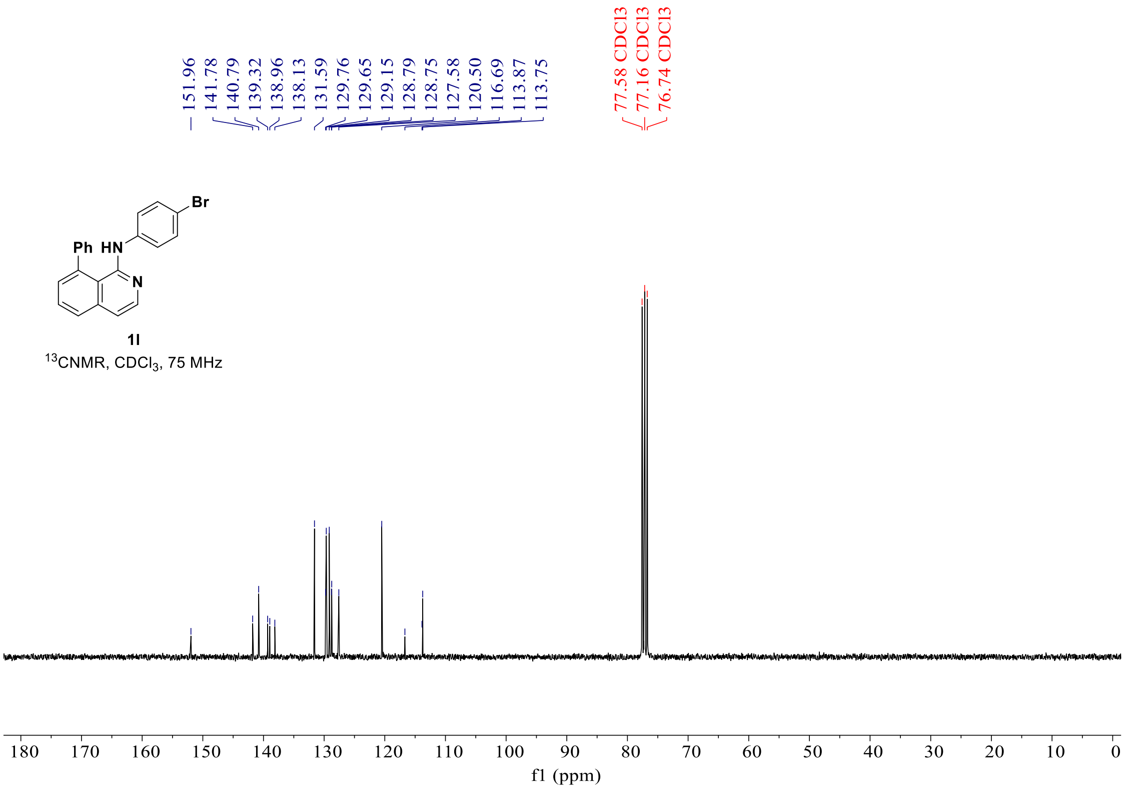

N-(2,4-dimethylphenyl)-8-phenylisoquinolin-1-amine (1j)

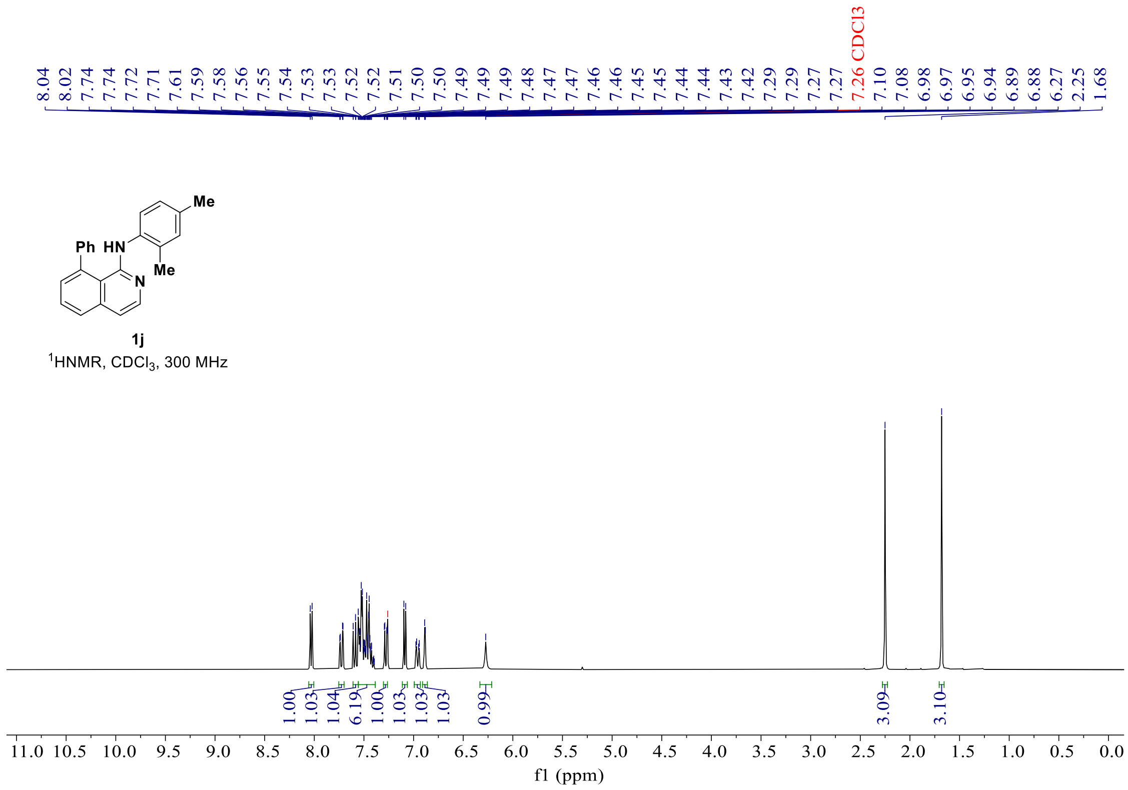

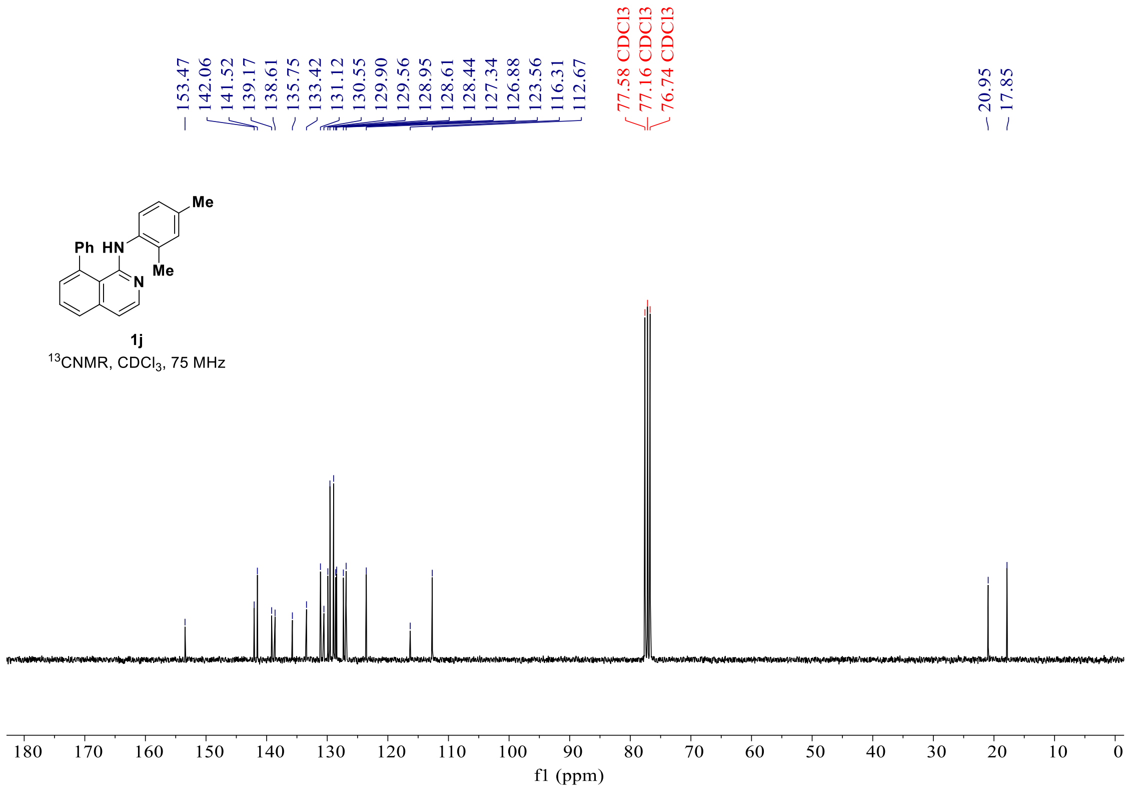

***N*-(naphthalen-1-yl)-8-phenylisoquinolin-1-amine (1k)**

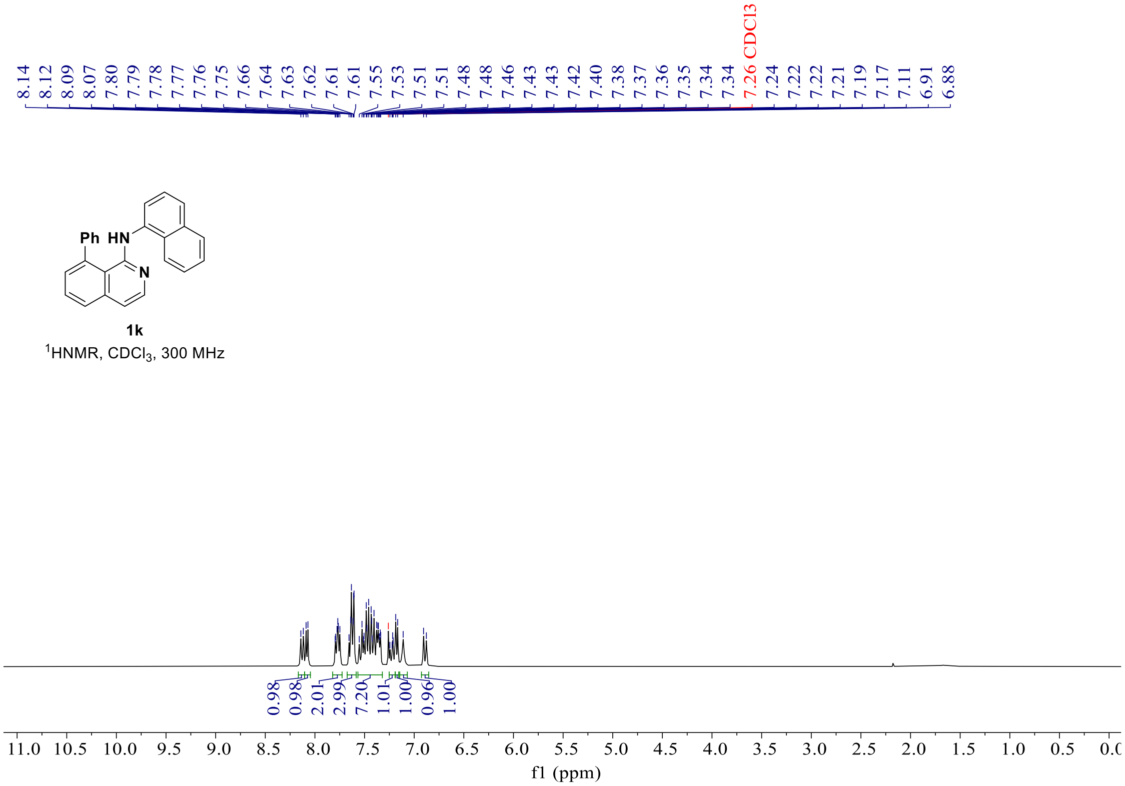

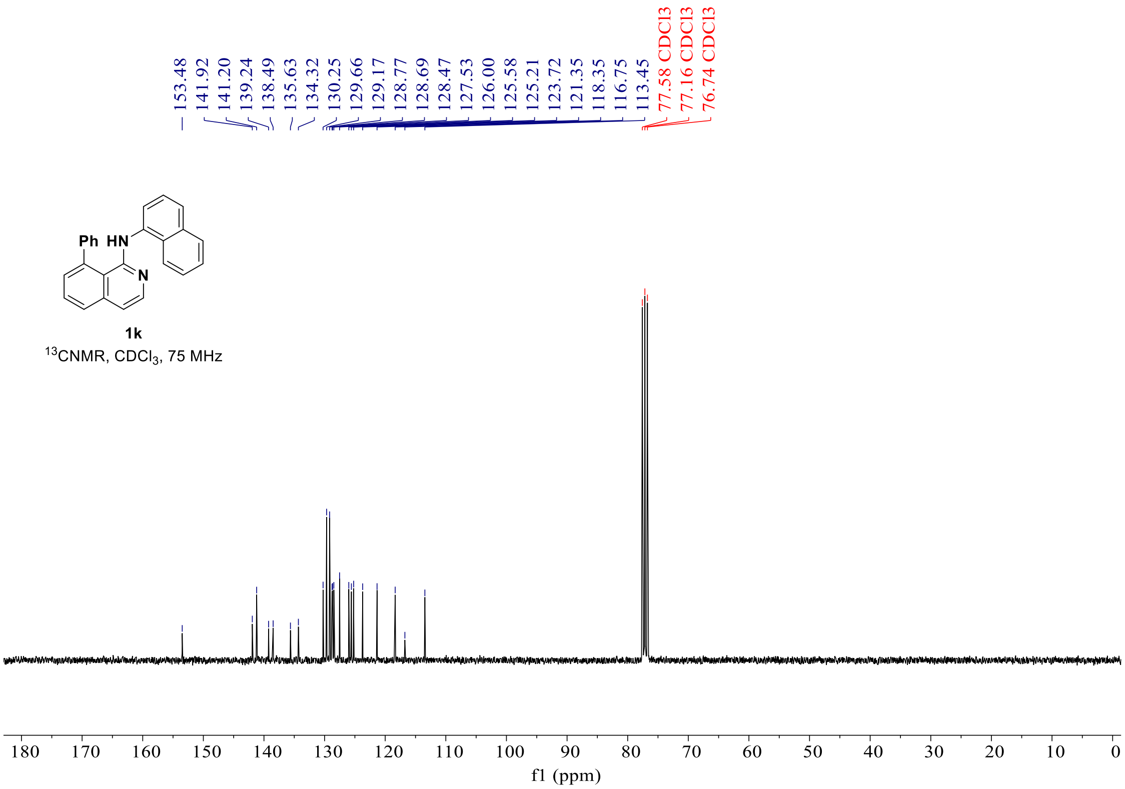

8-(4-methoxyphenyl)-*N*-phenylisoquinolin-1-amine (1l)

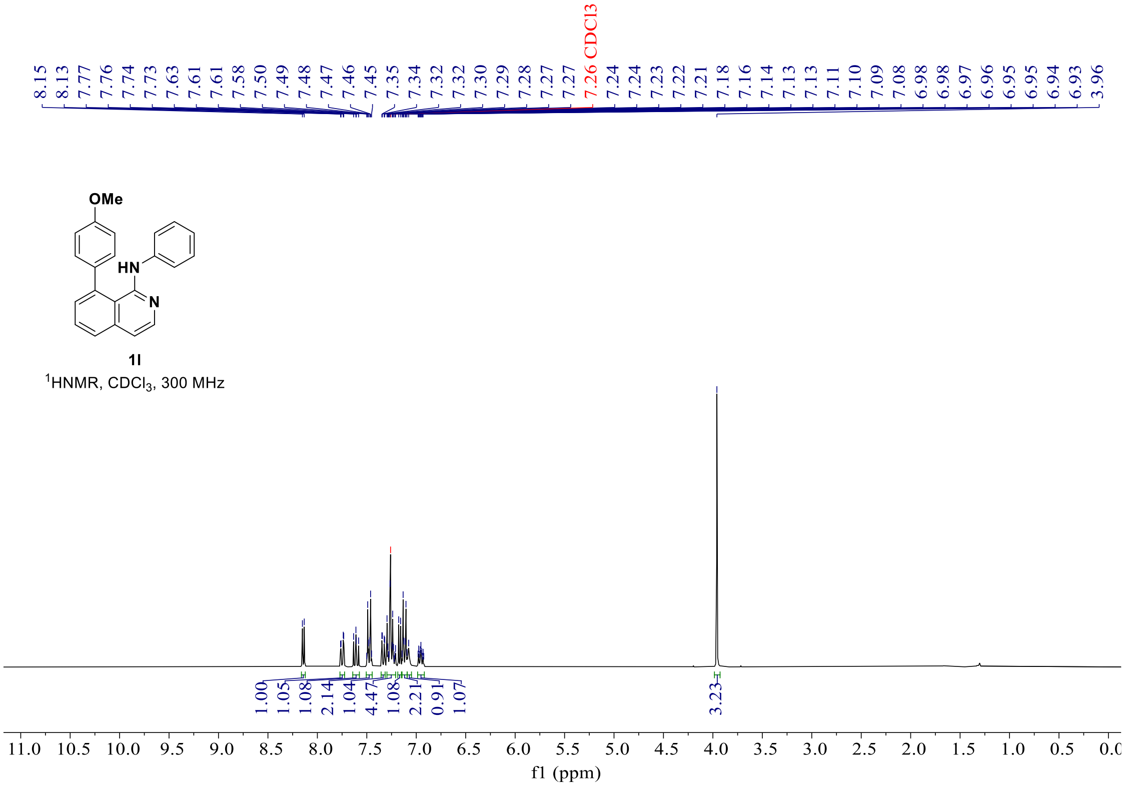

1l

^{1}HNMR, $CDCl_3$, 300 MHz

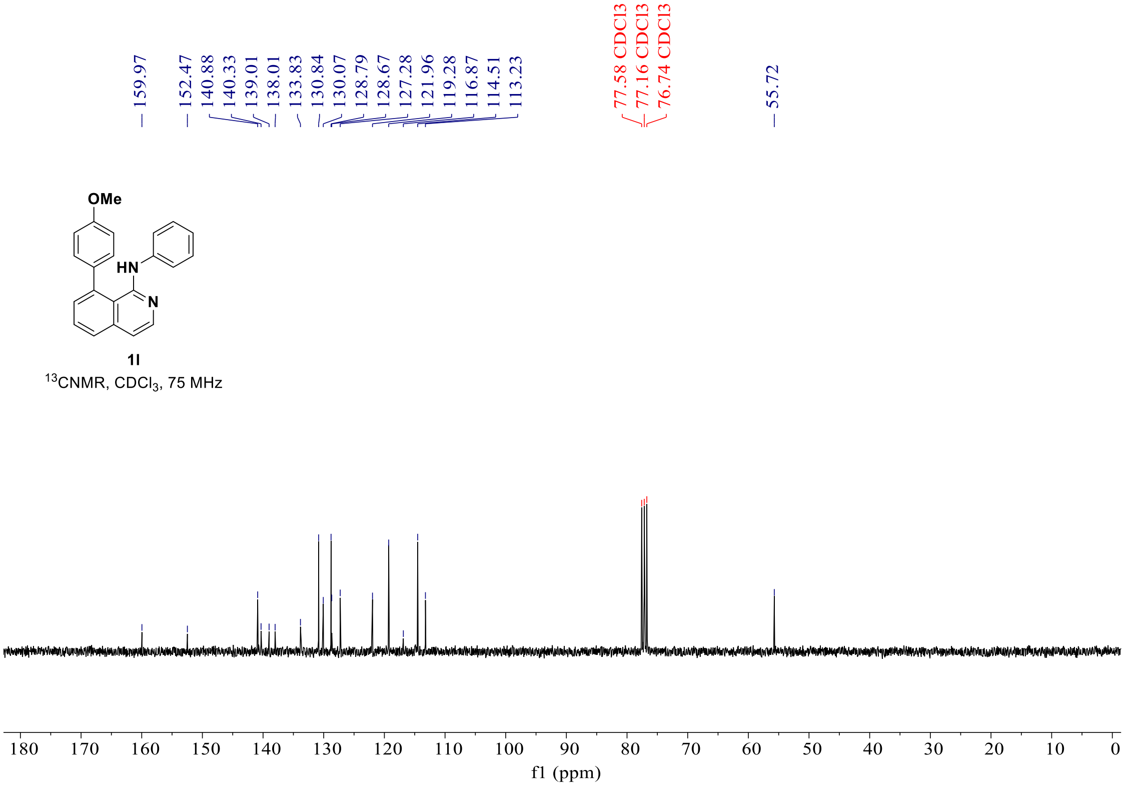

1l

^{13}CNMR, $CDCl_3$, 75 MHz

8-(4-fluorophenyl)-*N*-phenylisoquinolin-1-amine (1m)

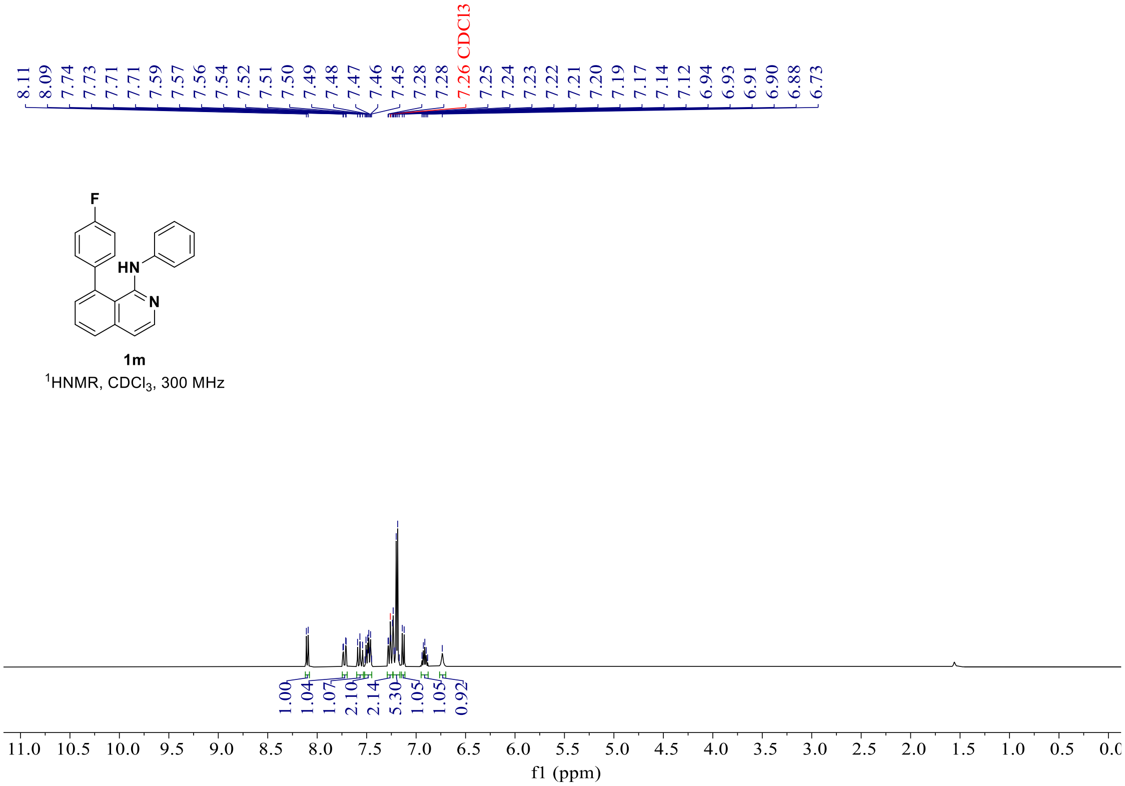

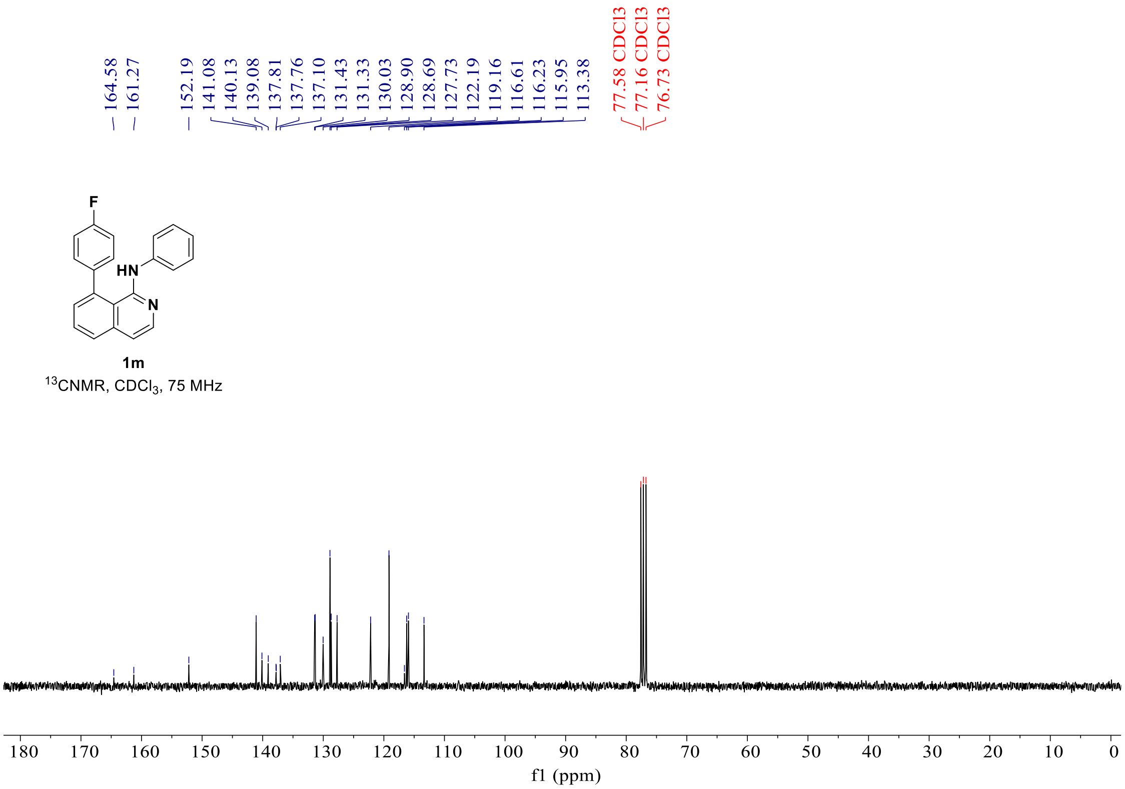

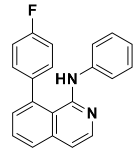

1m

^{19}FNMR, $CDCl_3$, 282 MHz

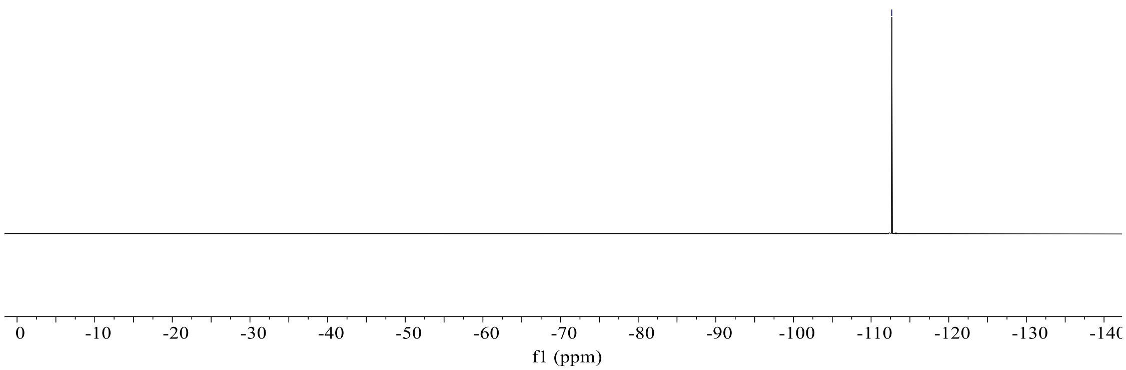

(*S*)-1-(2,3-diphenyl-1*H*-indol-1-yl)-8-phenylisoquinoline (3)

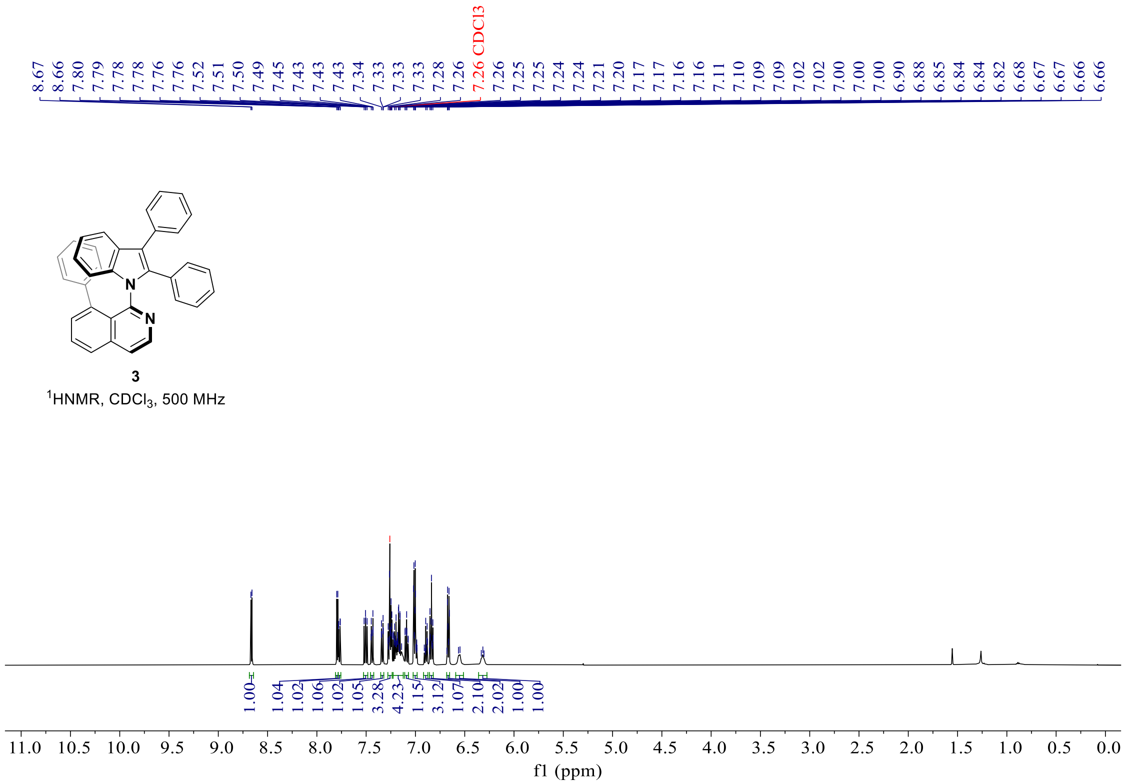

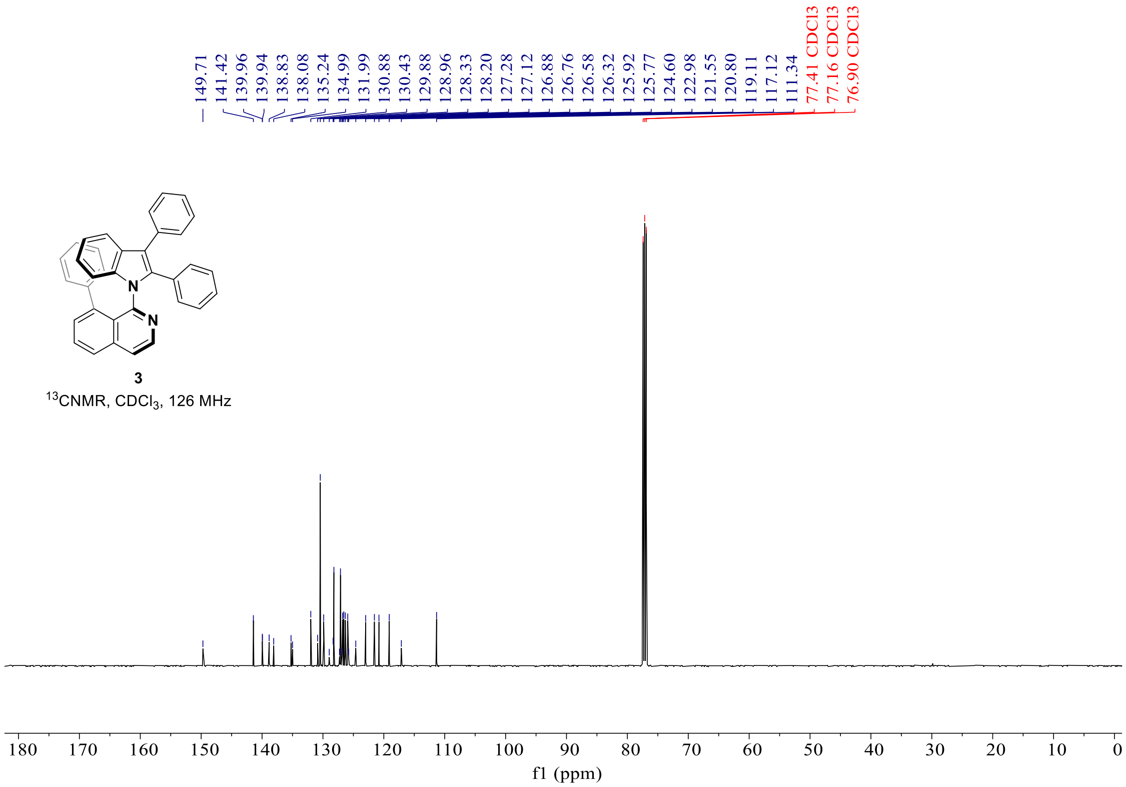

(*S*)-1-(7-methyl-2,3-diphenyl-1*H*-indol-1-yl)-8-phenylisoquinoline (4)

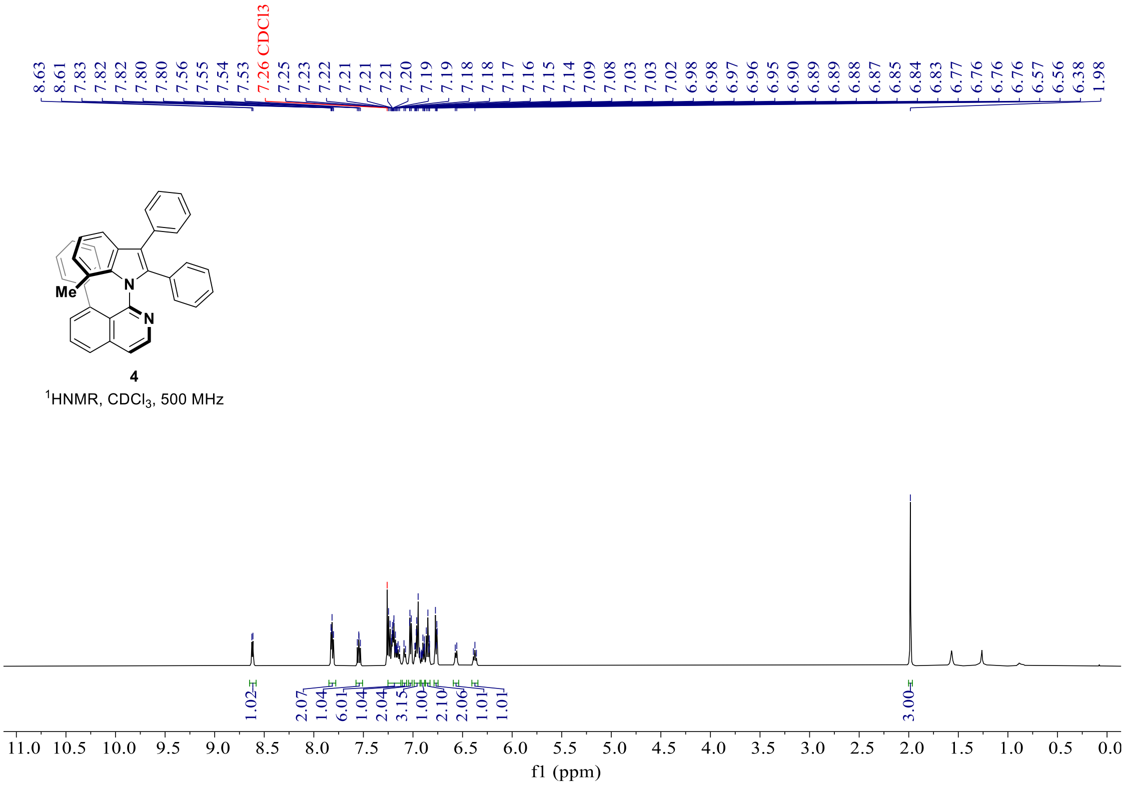

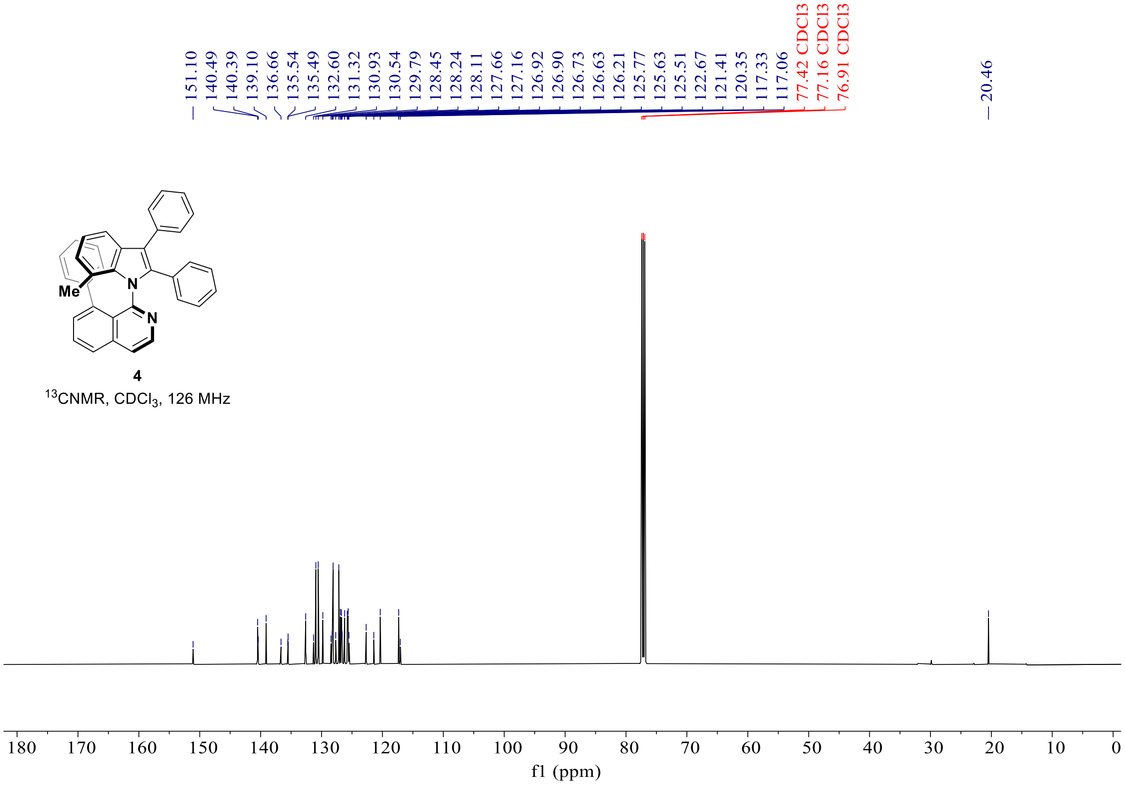

(*R*)-1-(7-fluoro-2,3-diphenyl-1*H*-indol-1-yl)-8-phenylisoquinoline (5)

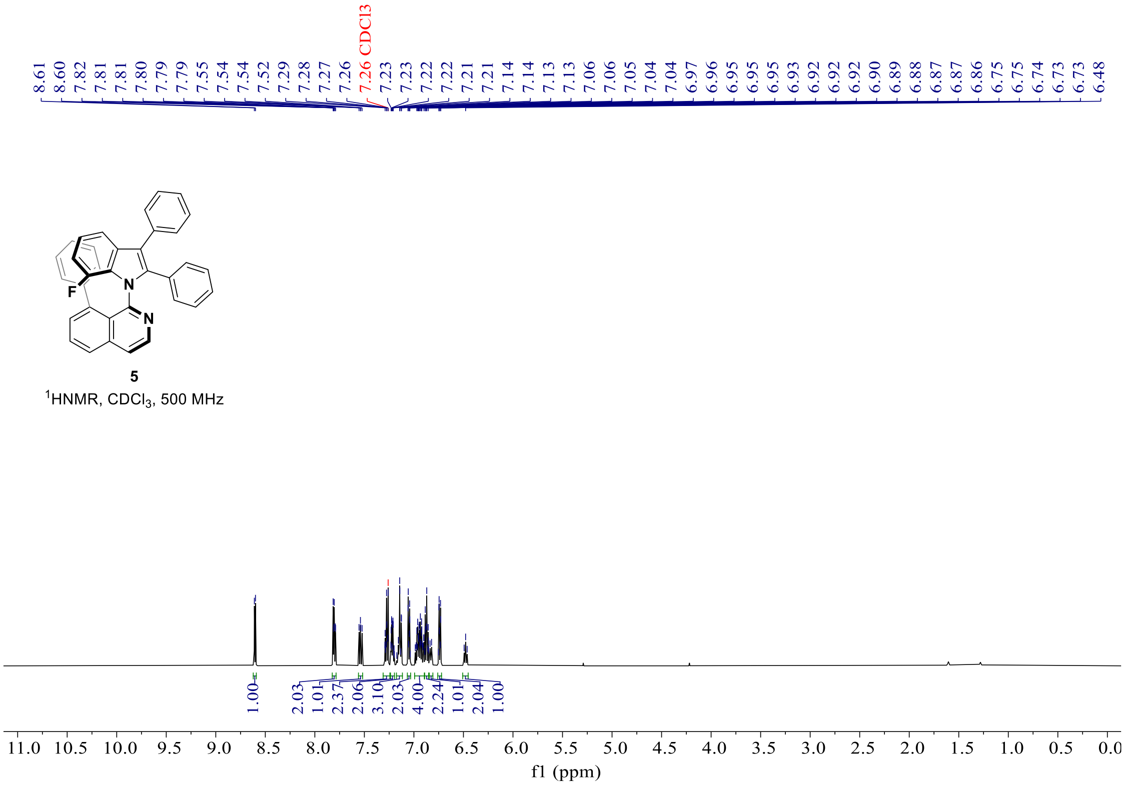

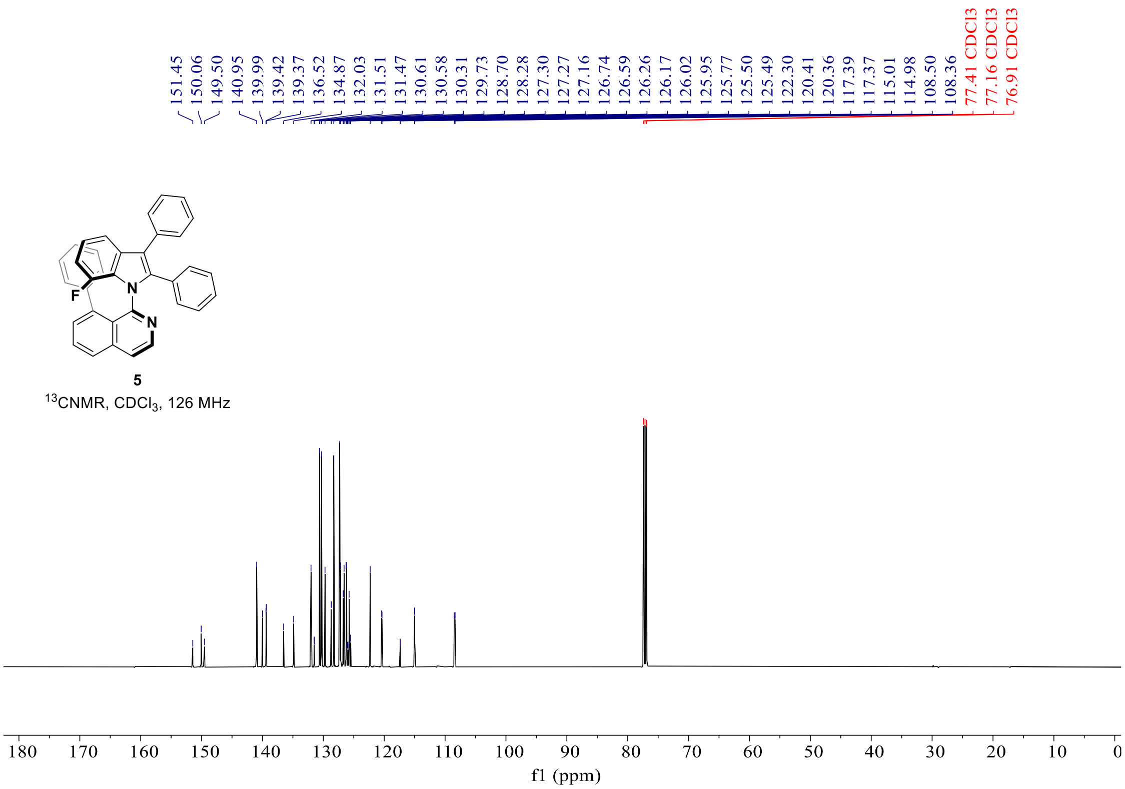

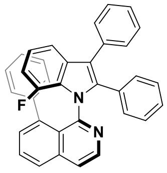

5

^{19}FNMR, $CDCl_3$, 471 MHz

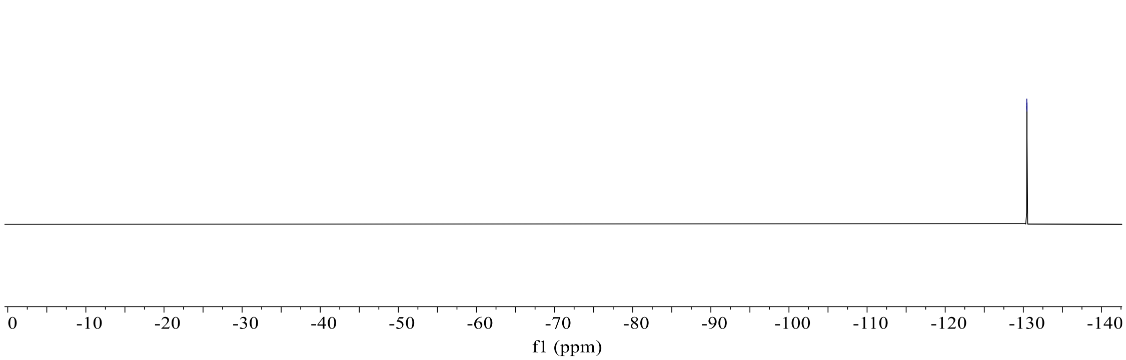

(*S*)-1-(6-methyl-2,3-diphenyl-1*H*-indol-1-yl)-8-phenylisoquinoline (6)

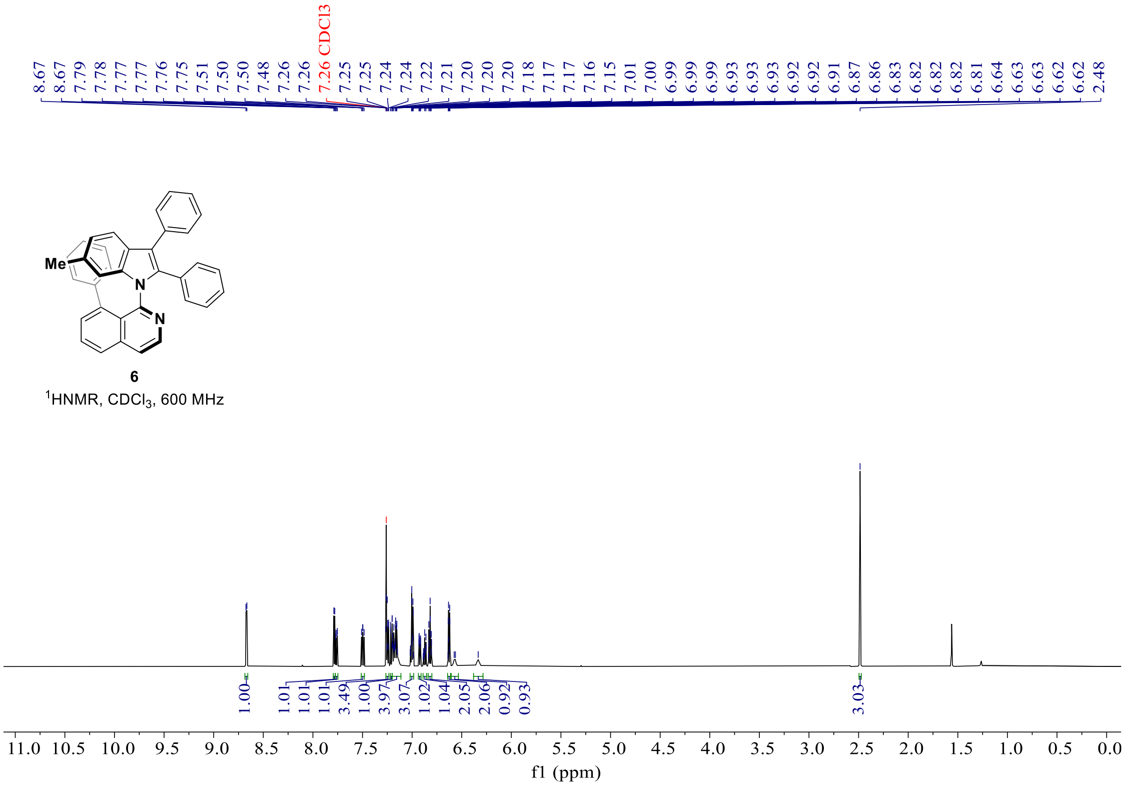

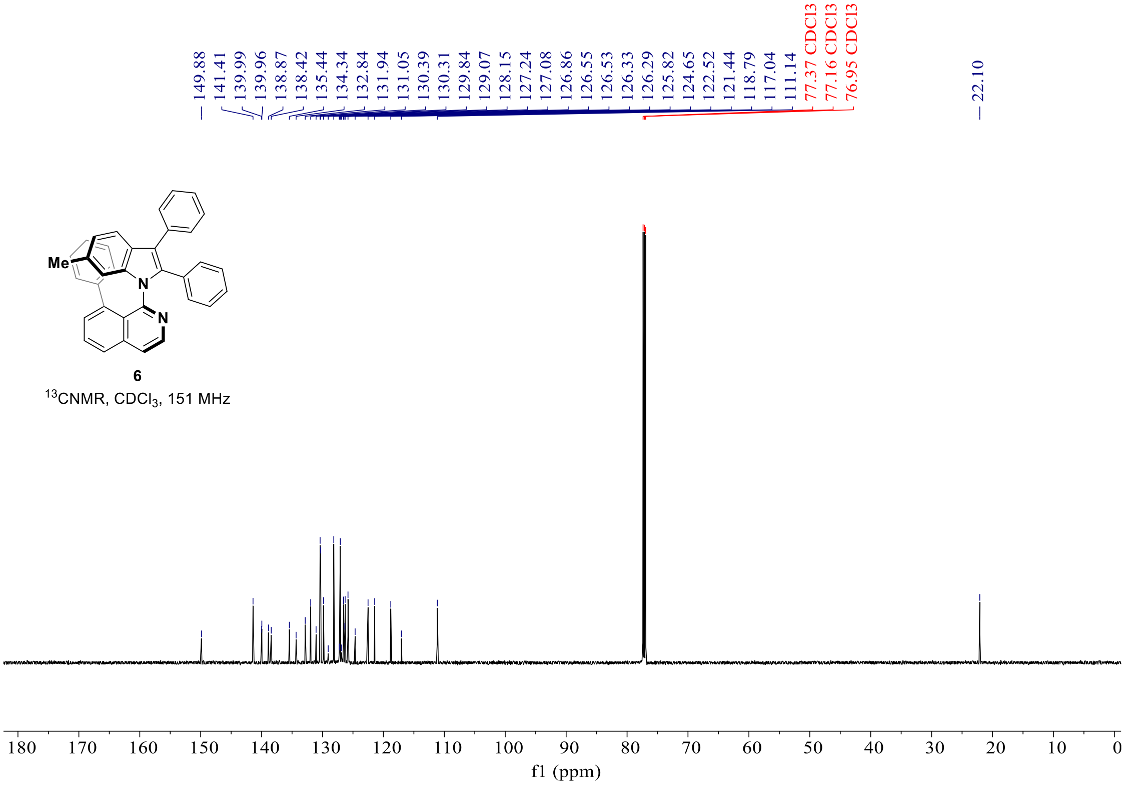

Methyl (*S*)-2,3-diphenyl-1-(8-phenylisoquinolin-1-yl)-1*H*-indole-6-carboxylate (7)

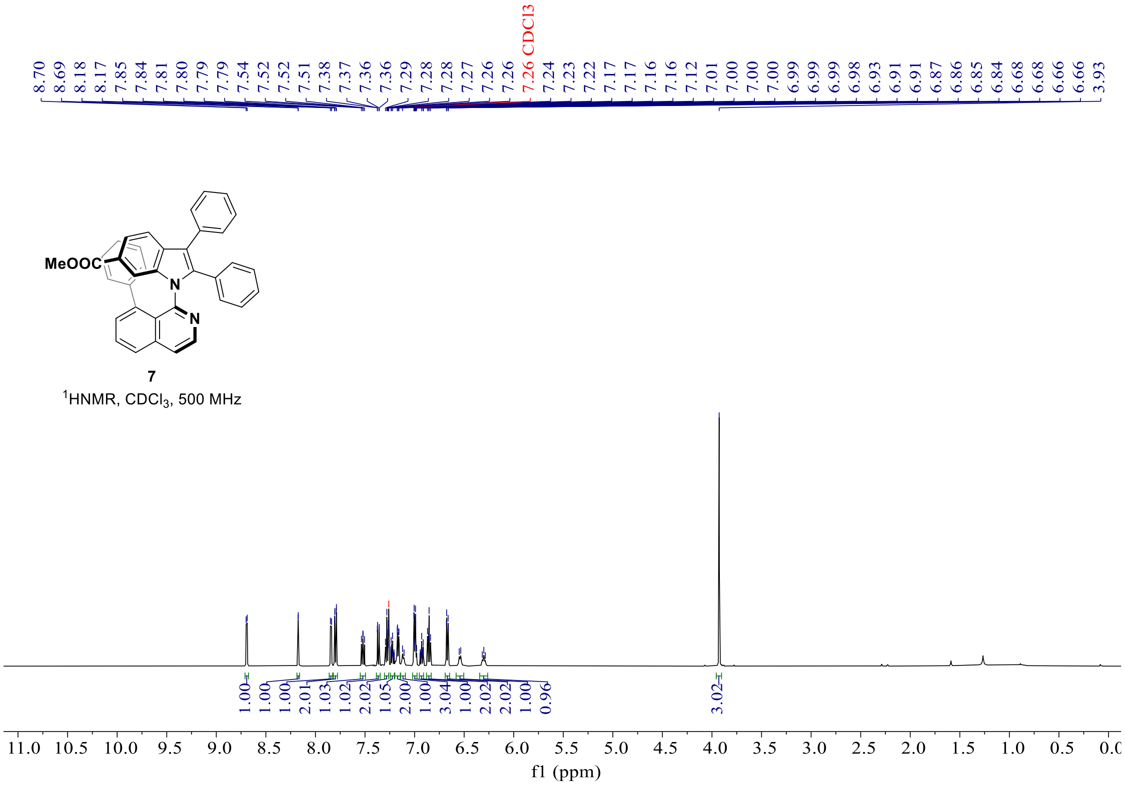

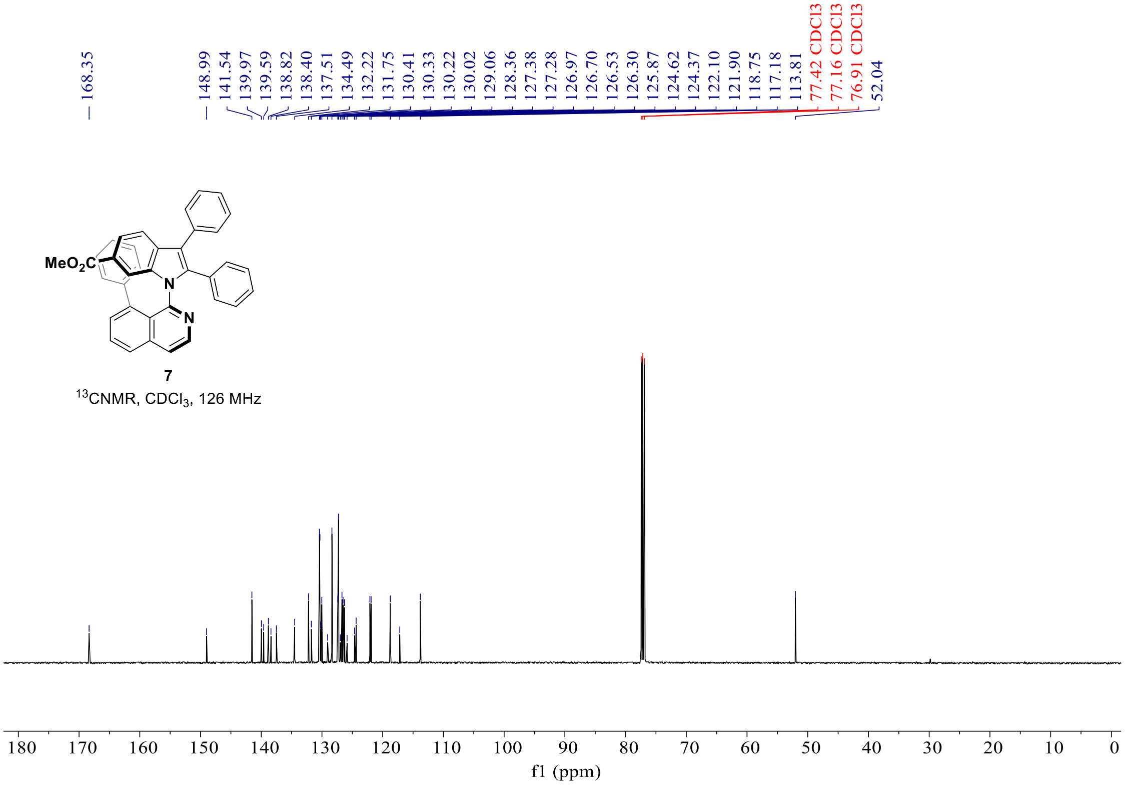

(*S*)-1-(6-chloro-2,3-diphenyl-1*H*-indol-1-yl)-8-phenylisoquinoline (8)

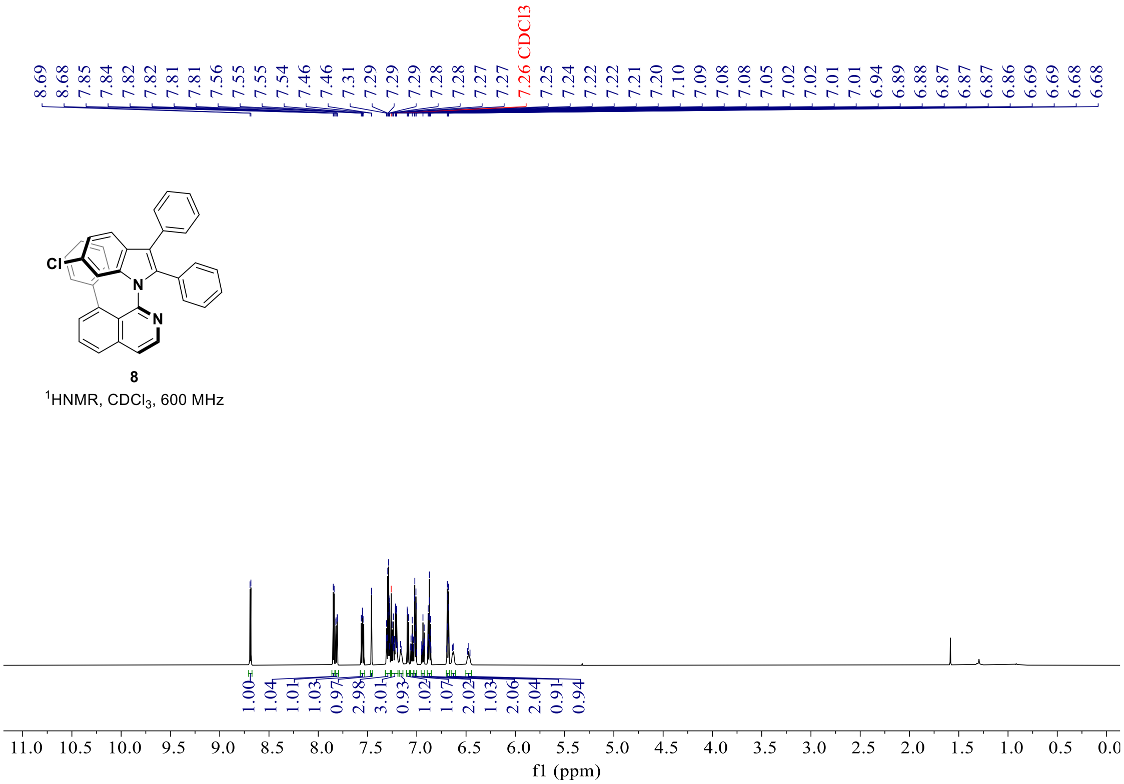

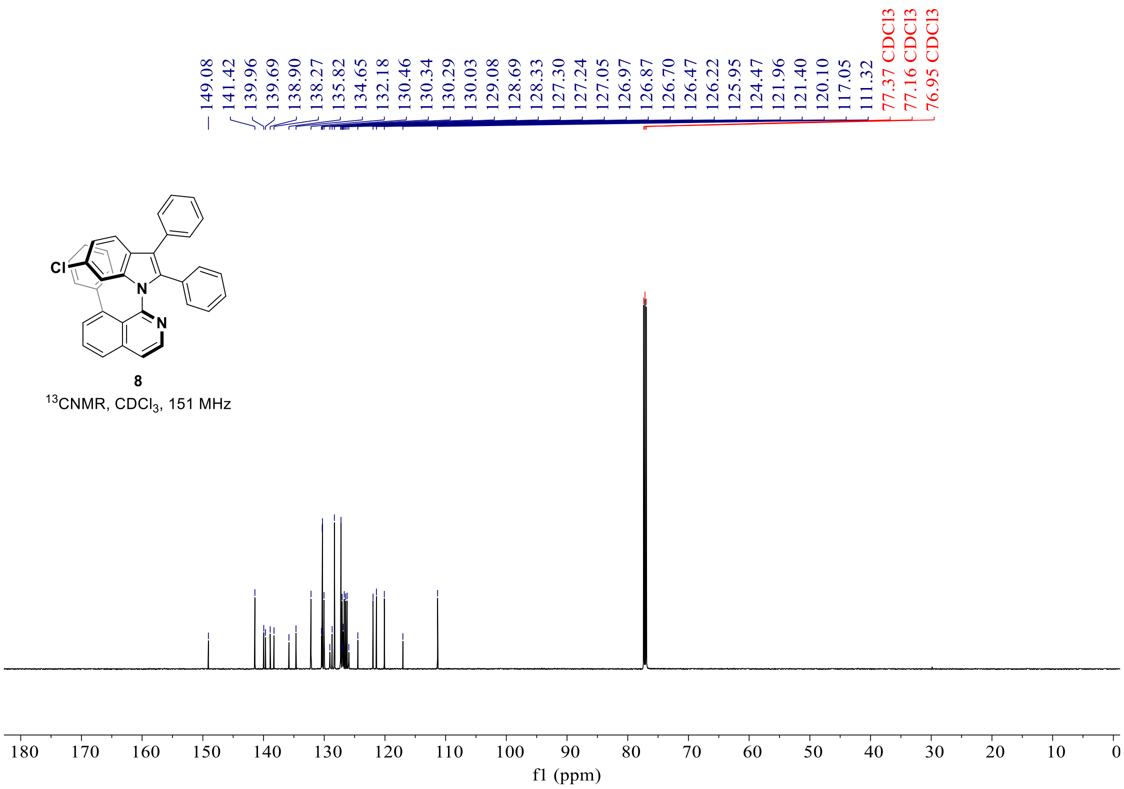

(*S*)-1-(5-methoxy-2,3-diphenyl-1*H*-indol-1-yl)-8-phenylisoquinoline (9)

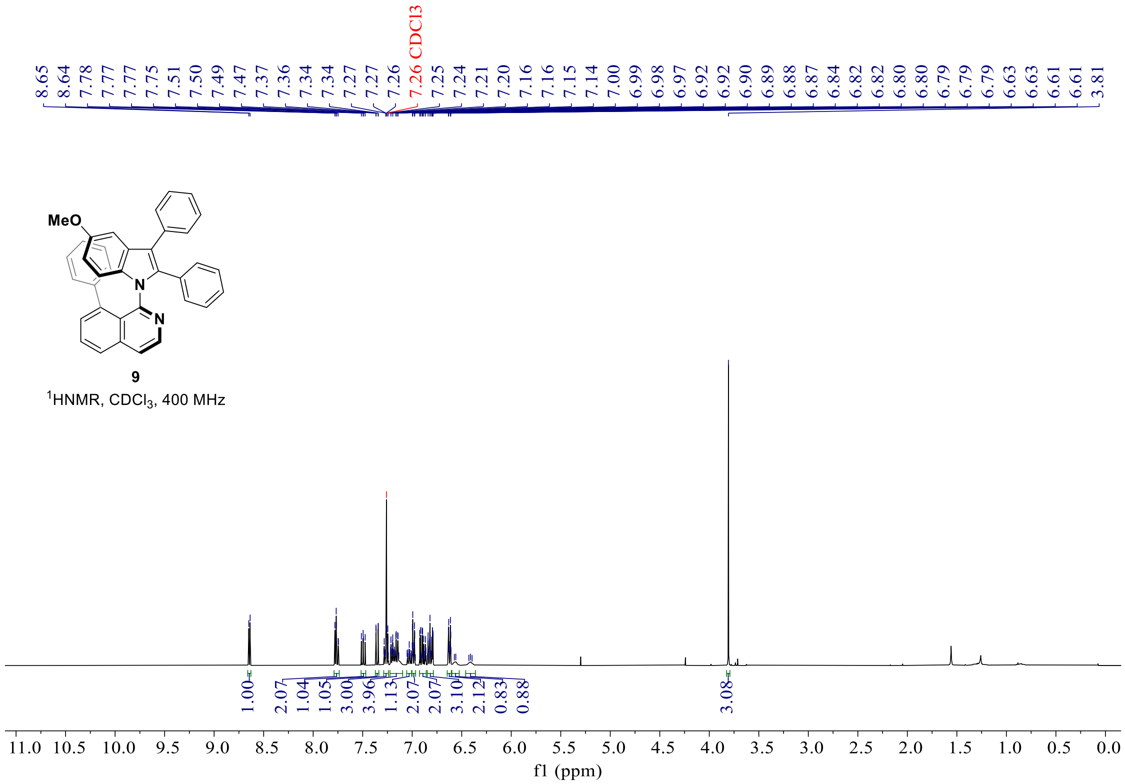

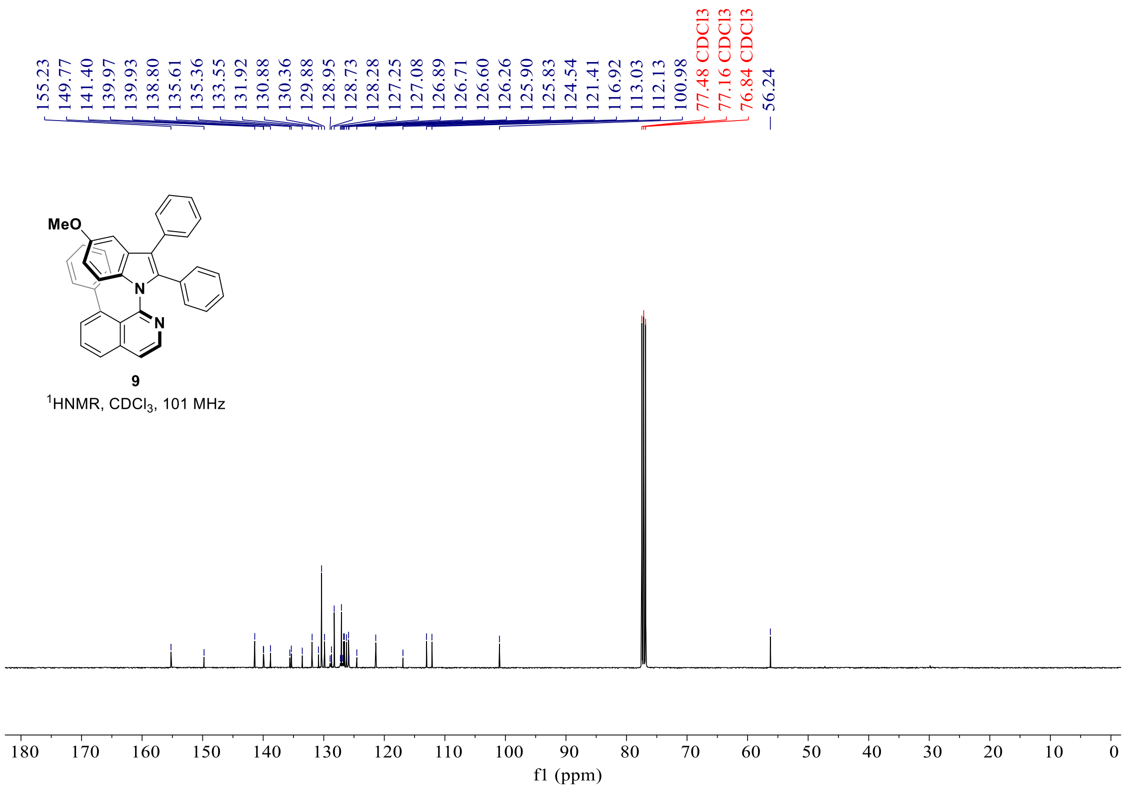

(*S*)-1-(2,3-diphenyl-5-(trifluoromethyl)-1H-indol-1-yl)-8-phenylisoquinoline (10)

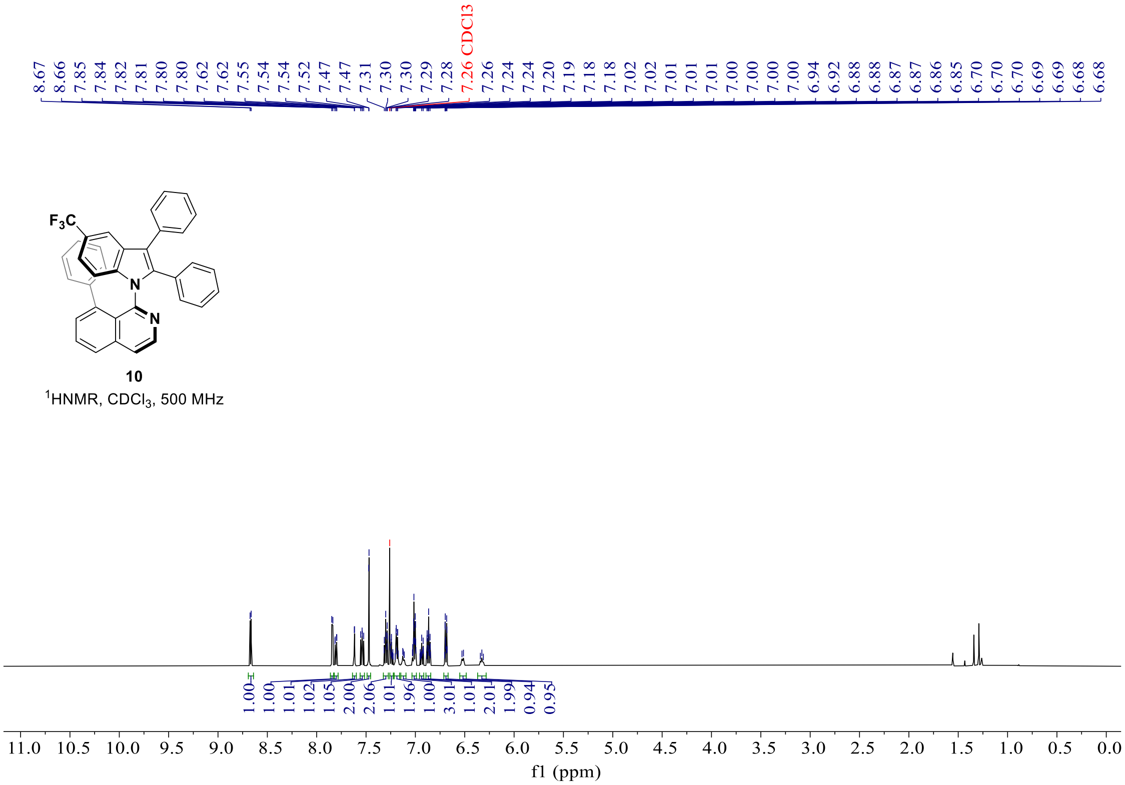

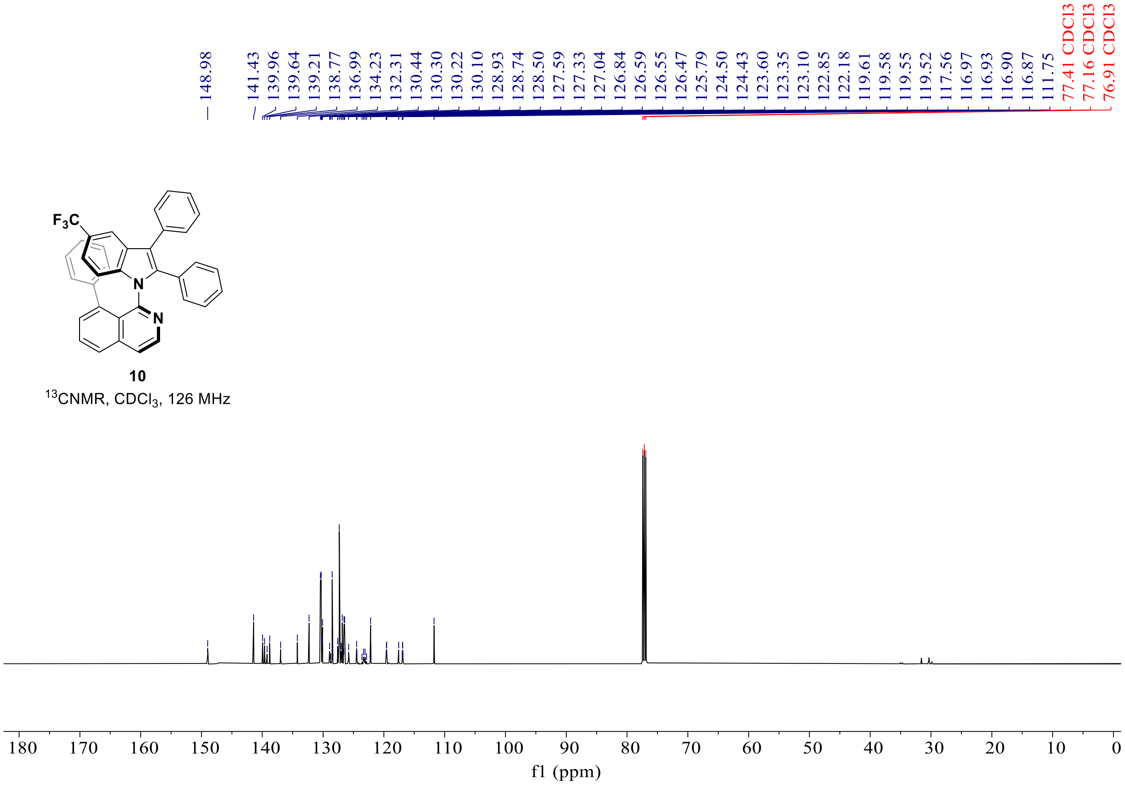

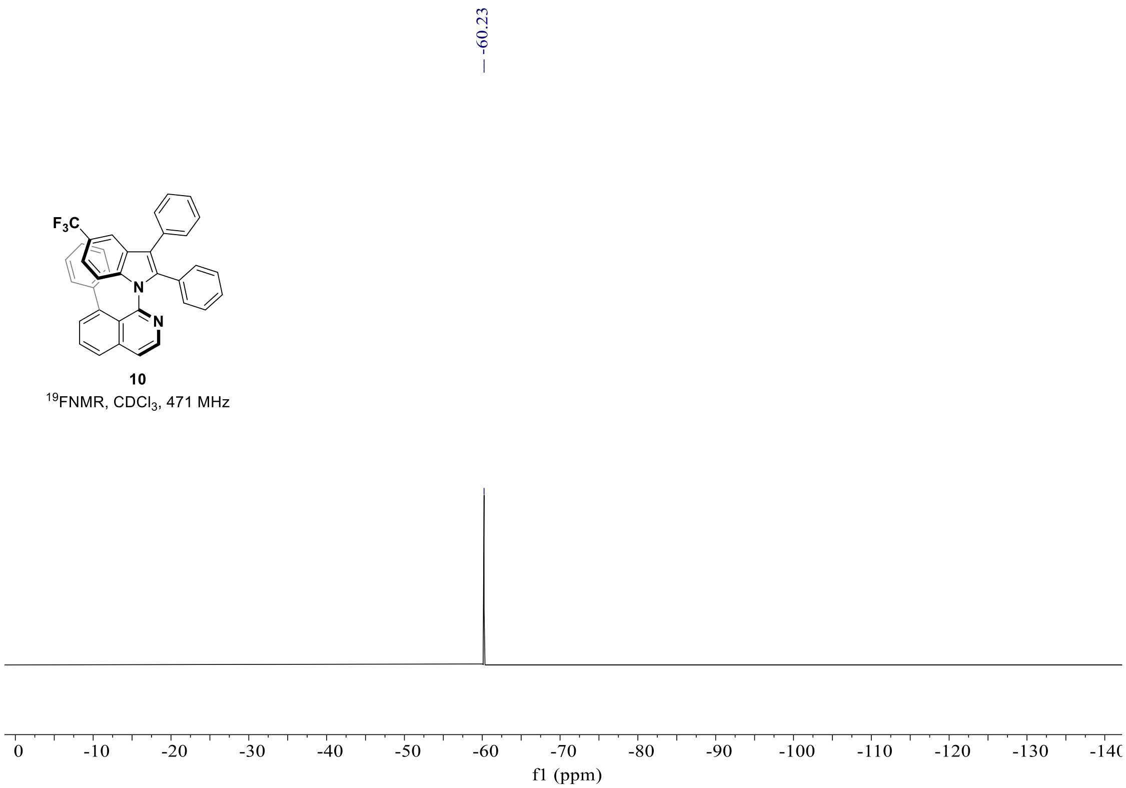

-60.23
F3C
N
N
10
19FNMR, CDCl3, 471 MHz
0
-10
-20
-30
-40
-50
-60
-70
-80
-90
-100
-110
-120
-130
f1 (ppm)

(*S*)-1-(5-bromo-2,3-diphenyl-1*H*-indol-1-yl)-8-phenylisoquinoline (11)

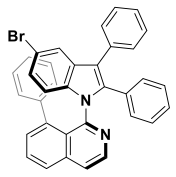

11

^{1}HNMR, $CDCl_3$, 500 MHz

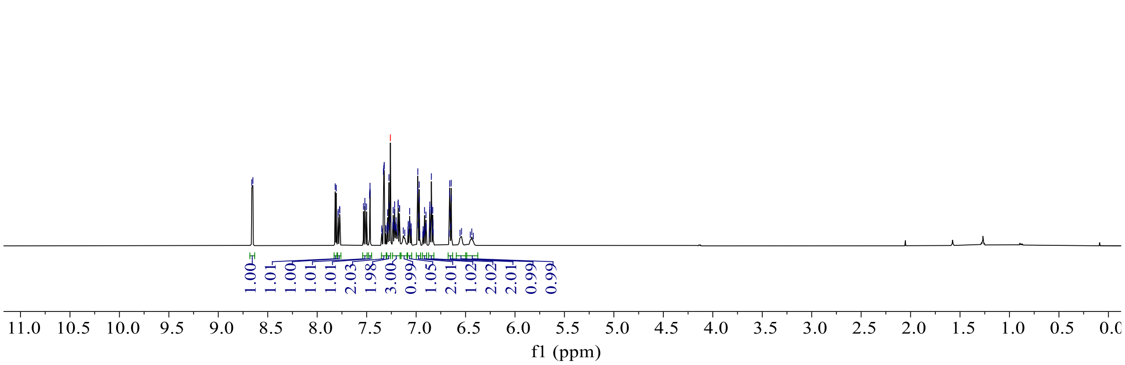

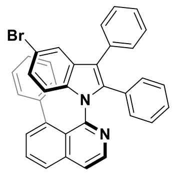

11

^{13}CNMR, $CDCl_3$, 126 MHz

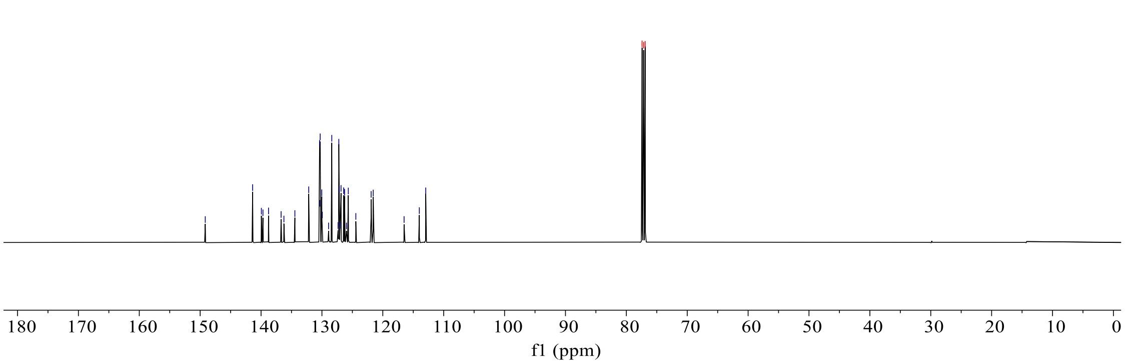

(*S*)-1-(5,7-dimethyl-2,3-diphenyl-1*H*-indol-1-yl)-8-phenylisoquinoline (12)

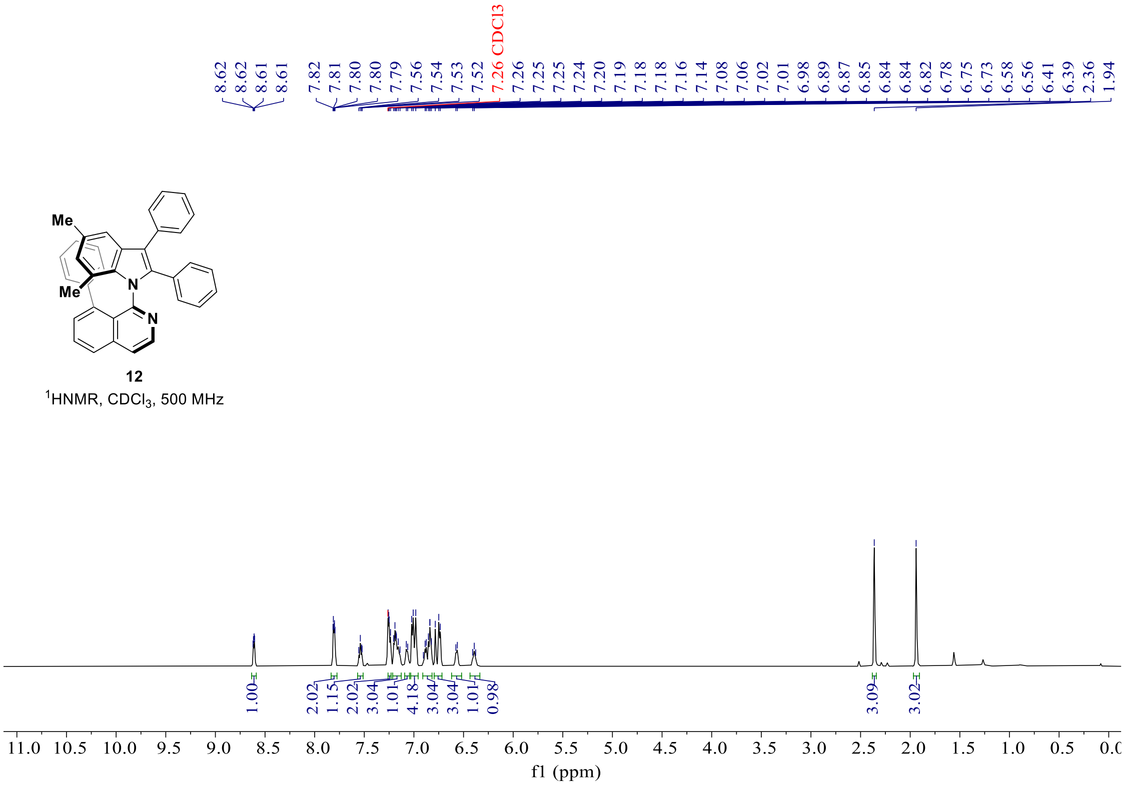

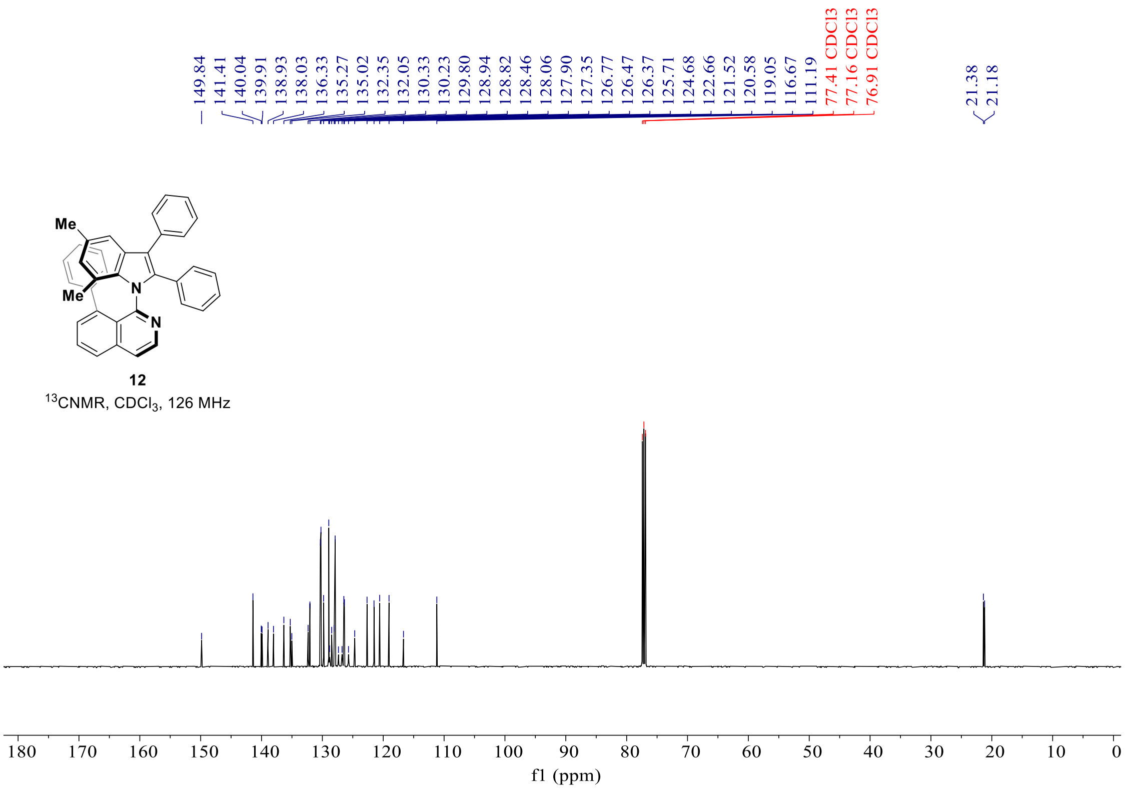

(*S*)-2,3-diphenyl-1-(8-phenylisoquinolin-1-yl)-1*H*-benzo[*g*]indole (13)

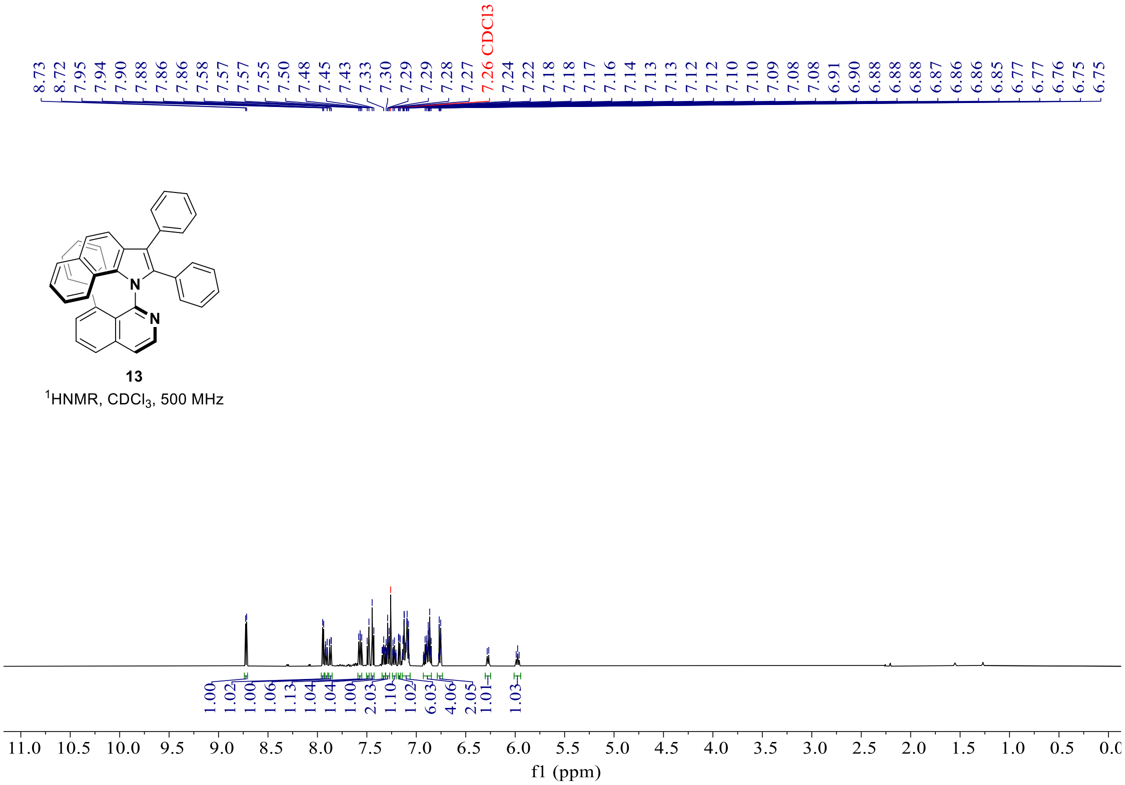

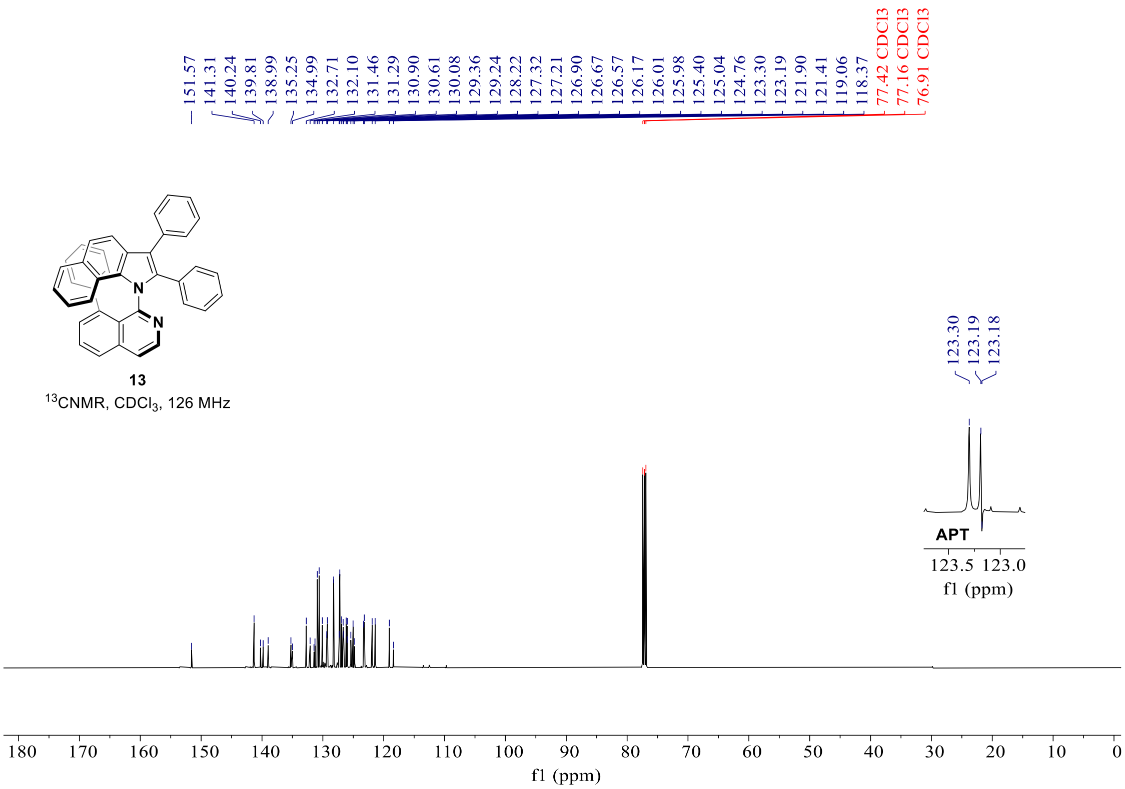

(*S*)-1-(2,3-diphenyl-1*H*-indol-1-yl)-8-(4-methoxyphenyl)isoquinoline (14)

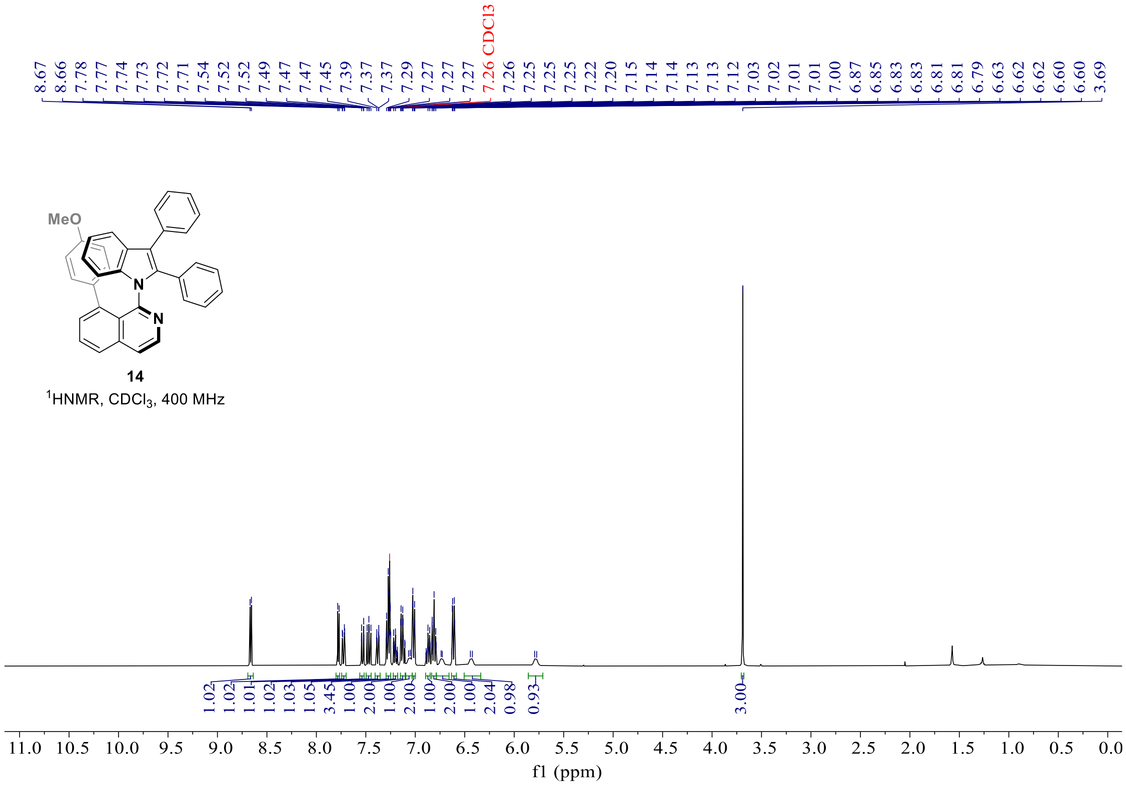

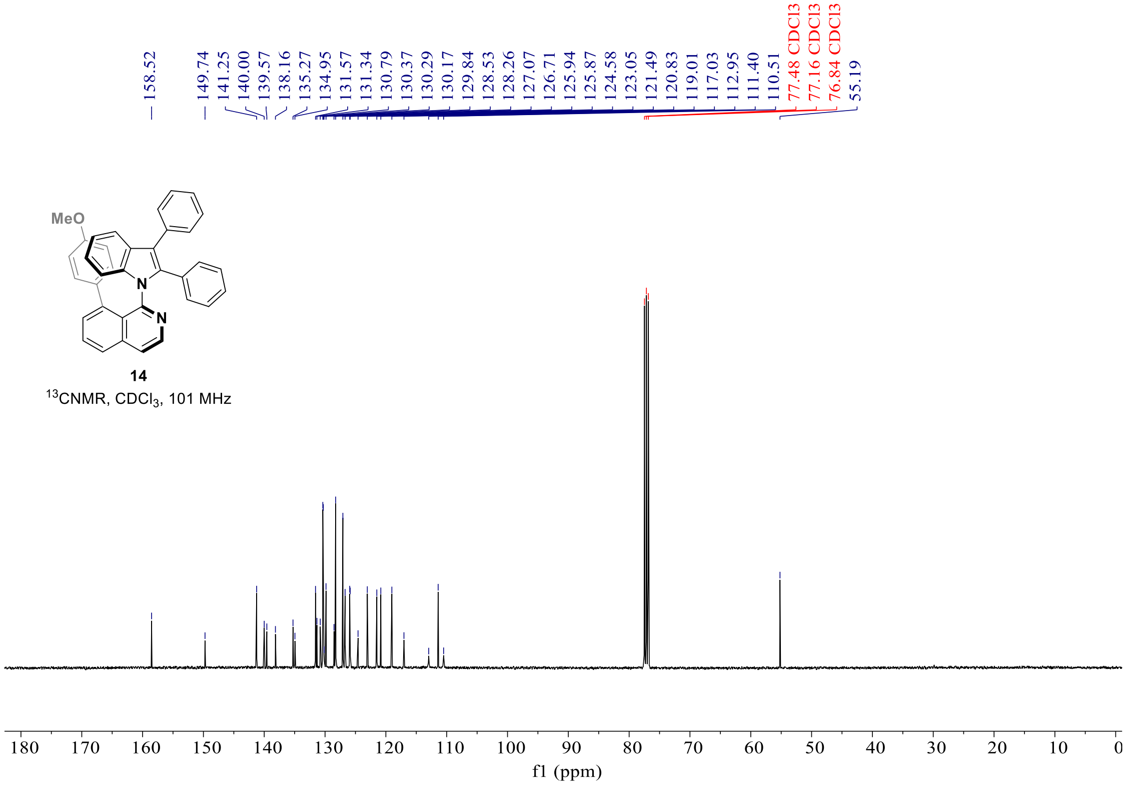

(*S*)-1-(2,3-diphenyl-1*H*-indol-1-yl)-8-(4-fluorophenyl)isoquinoline (15)

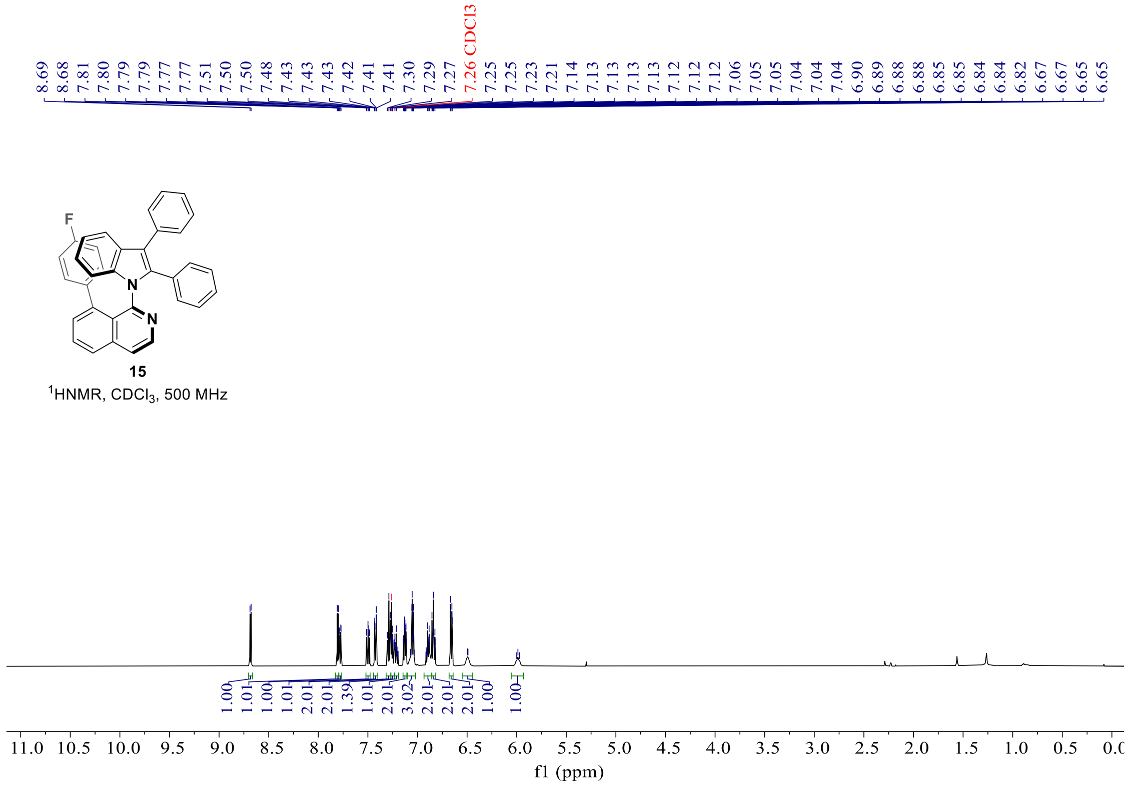

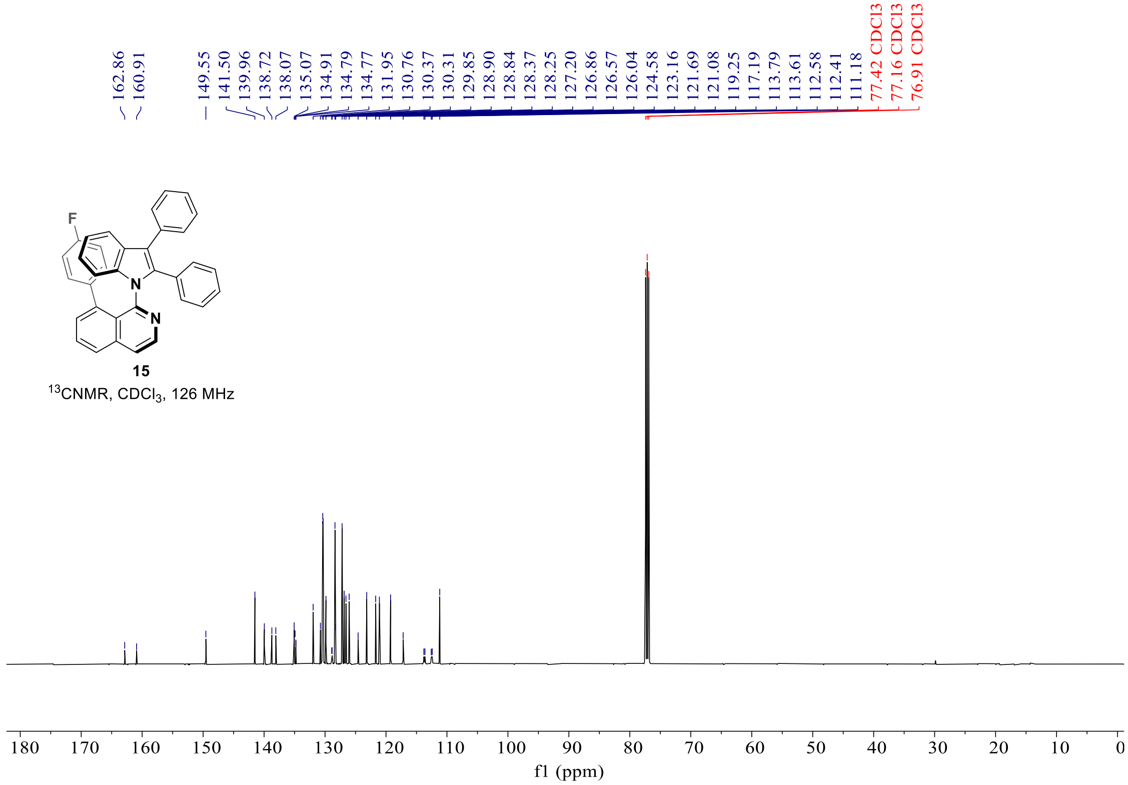

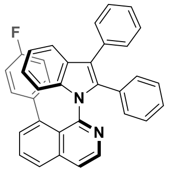

15

^{19}FNMR, $CDCl_3$, 377 MHz

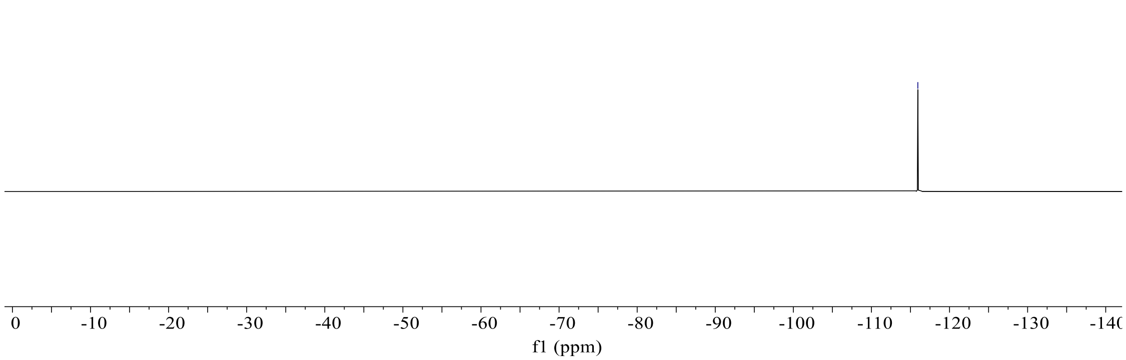

(*S*)-1-(2,3-diphenyl-1*H*-indol-1-yl)-8-methoxyisoquinoline (16)

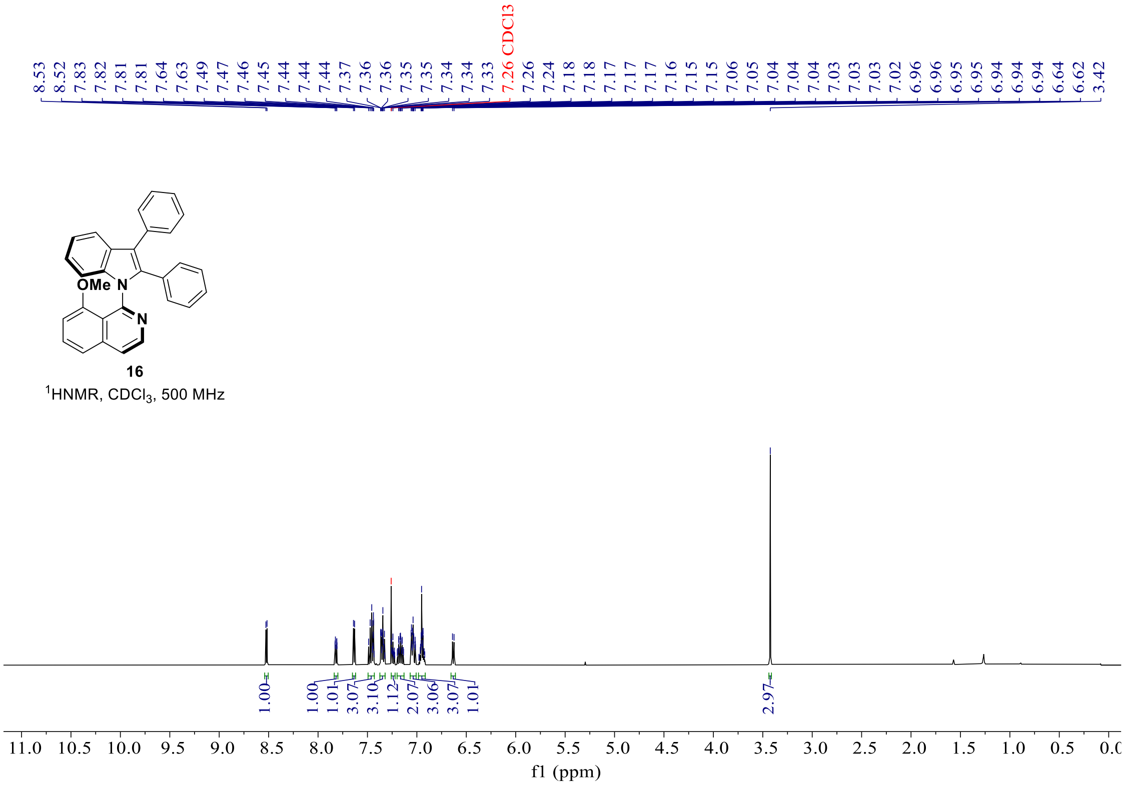

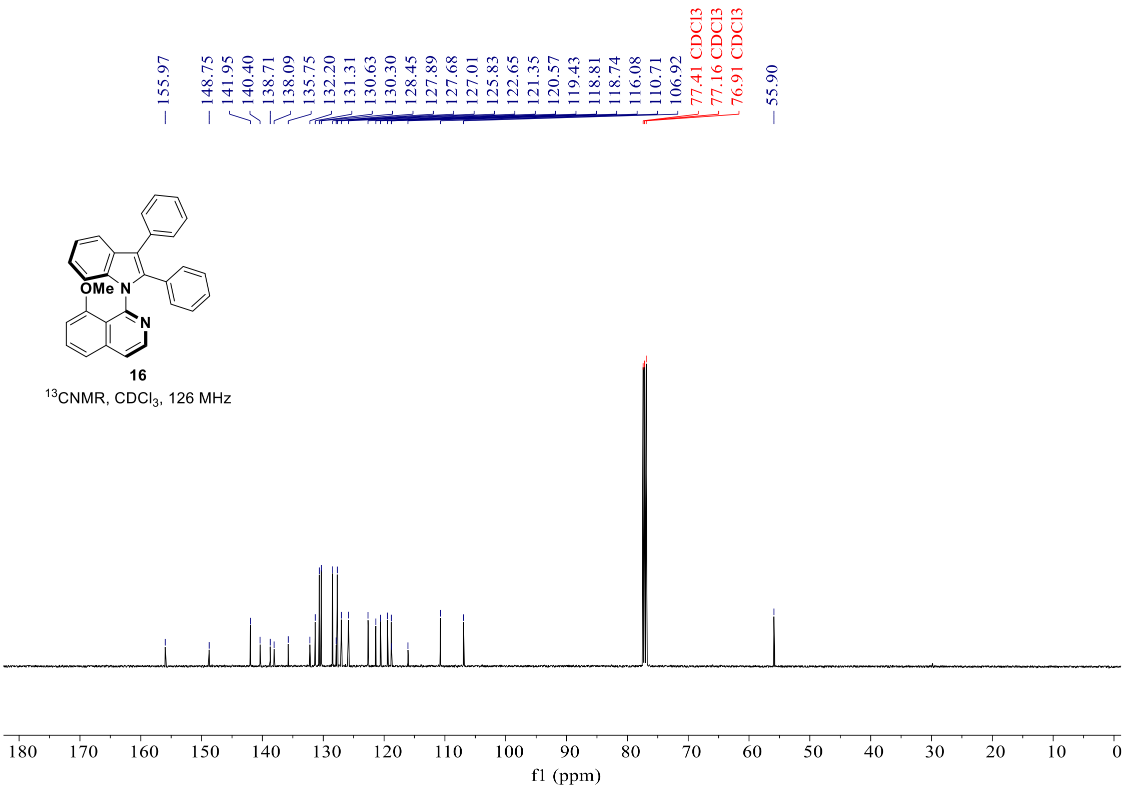

(*S*)-8-bromo-1-(2,3-diphenyl-1*H*-indol-1-yl)isoquinoline (17)

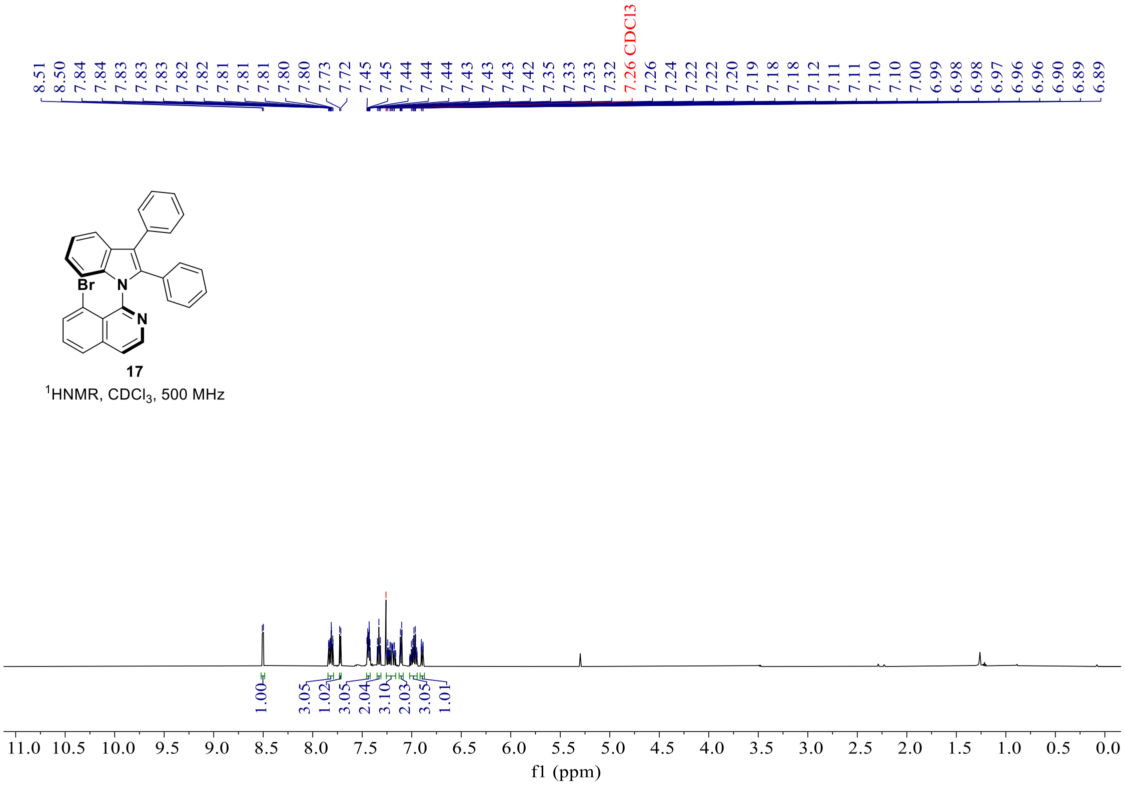

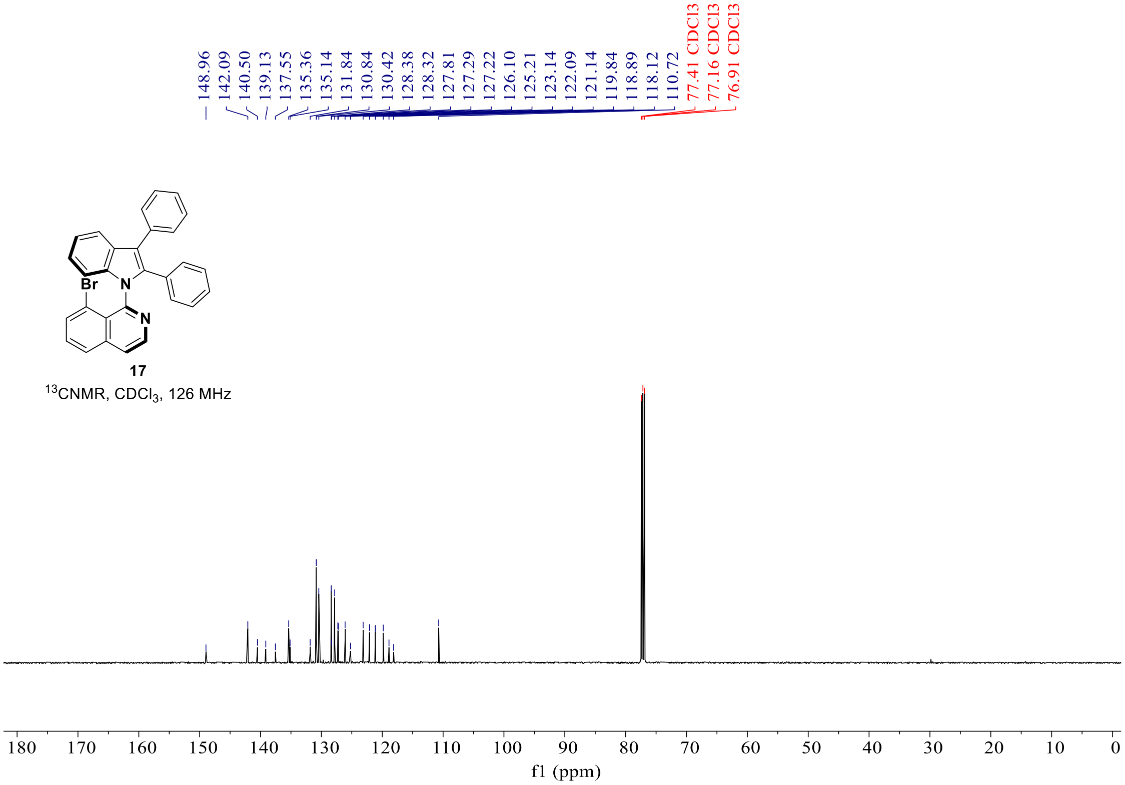

(*S*)-1-(2,3-di-*p*-tolyl-1*H*-indol-1-yl)-8-phenylisoquinoline (18)

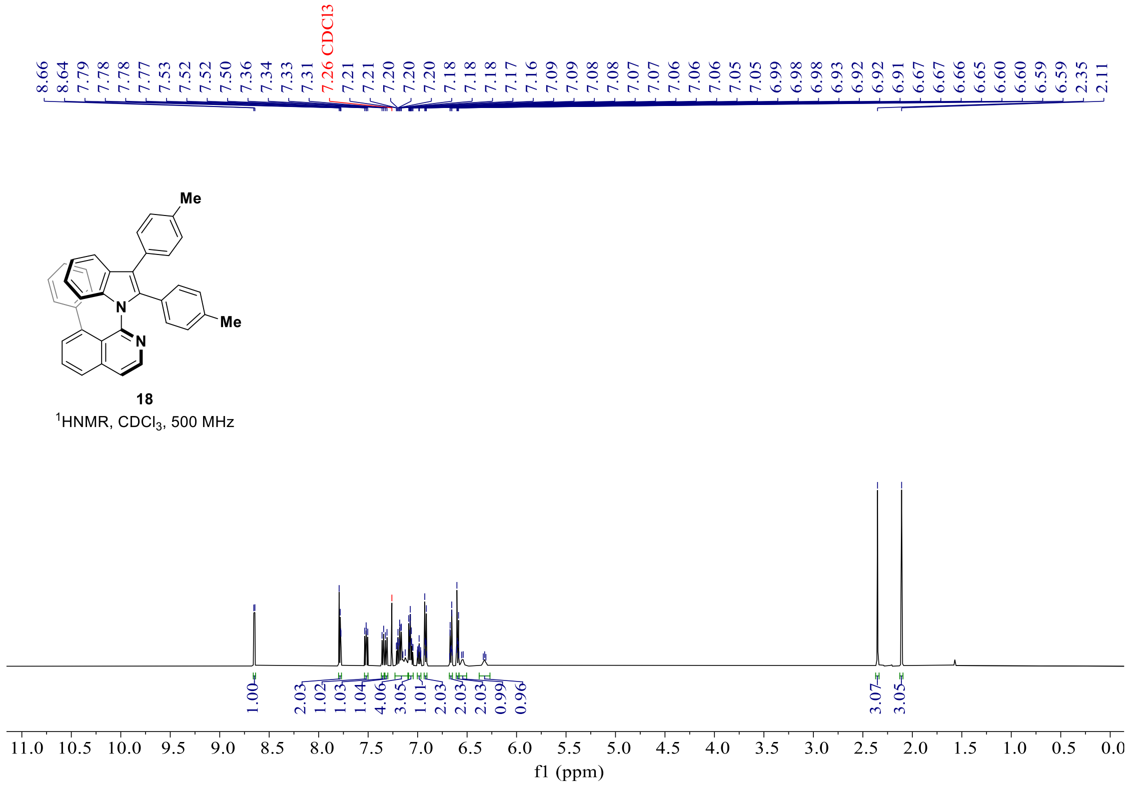

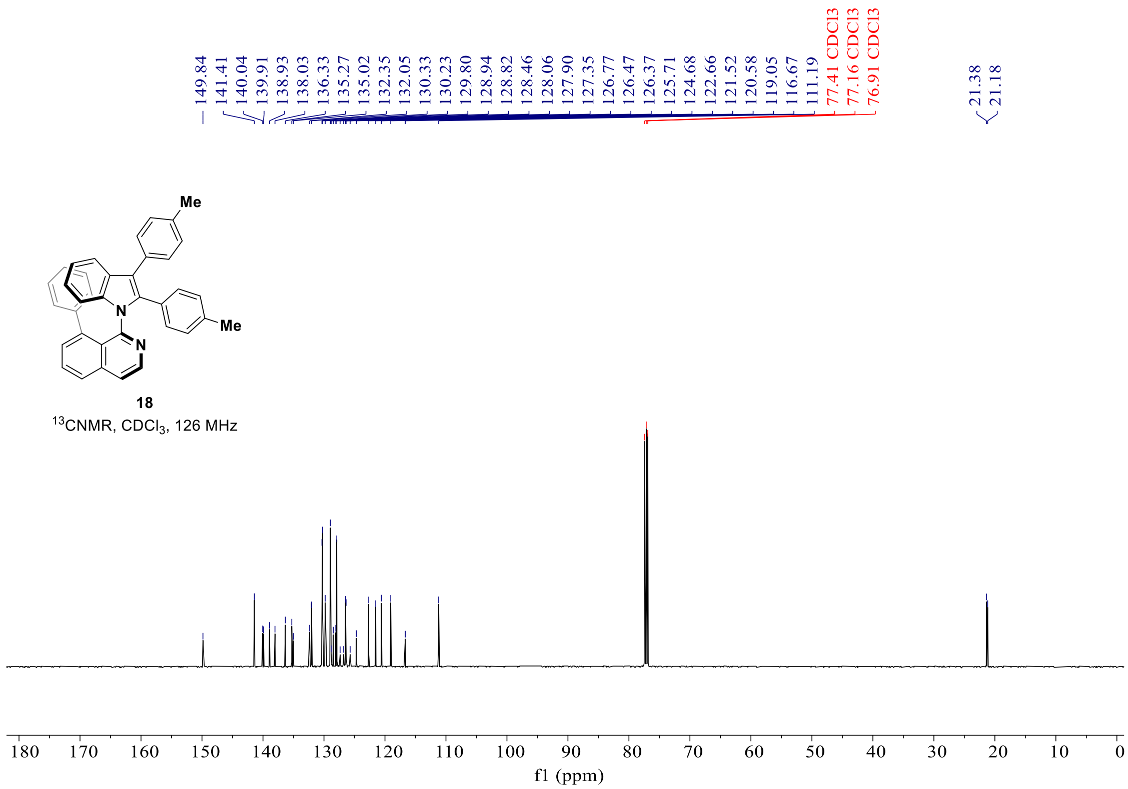

(*S*)-1-(2,3-bis(4-methoxyphenyl)-1*H*-indol-1-yl)-8-phenylisoquinoline (19)

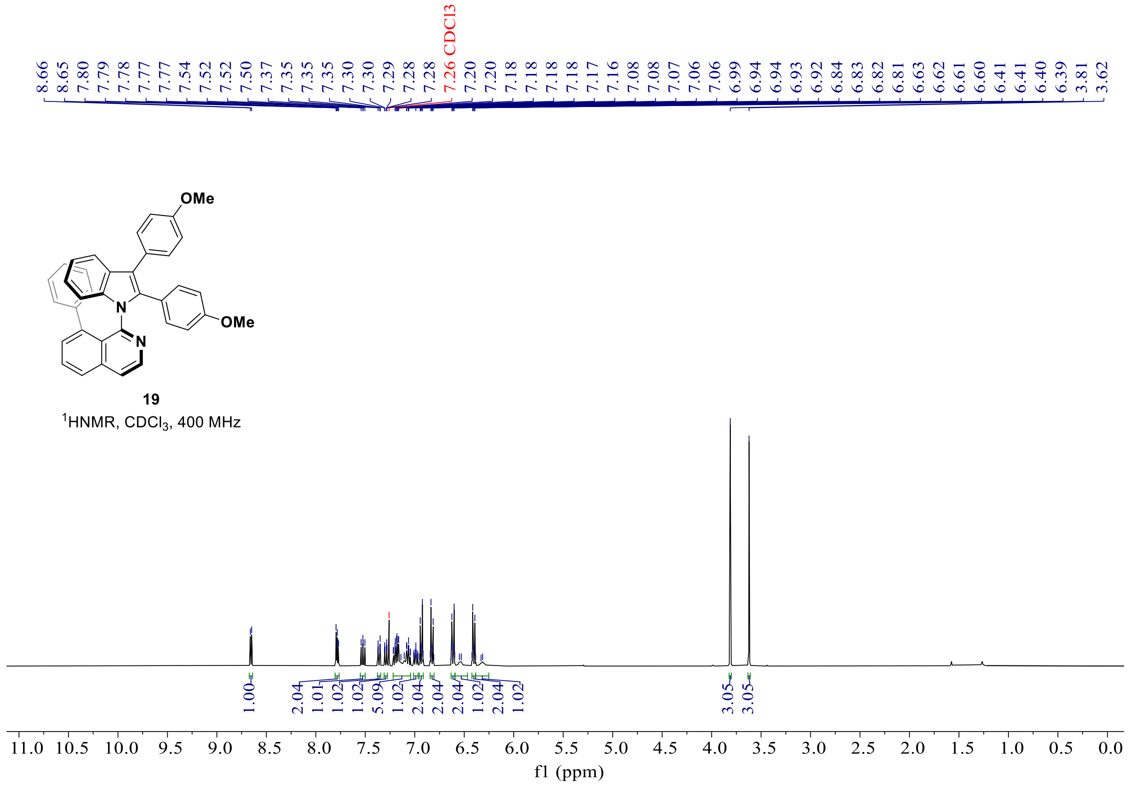

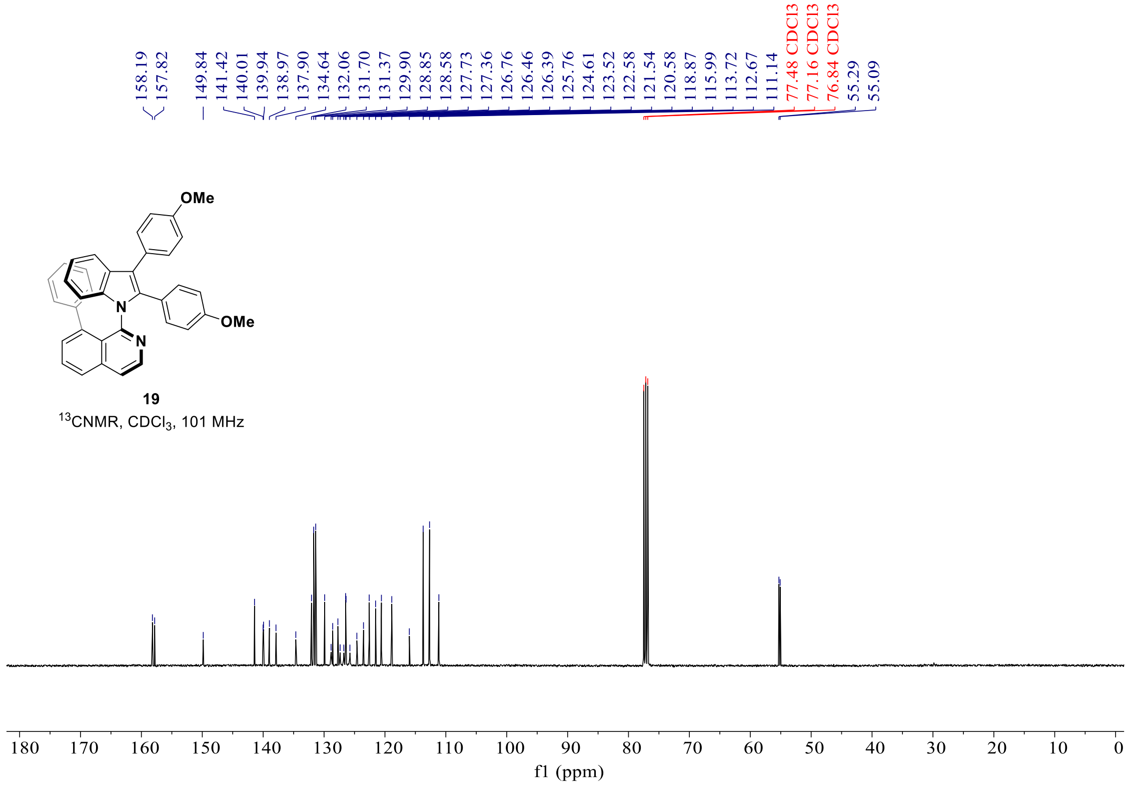

(*S*)-1-(2,3-bis(4-(trifluoromethyl)phenyl)-1*H*-indol-1-yl)-8-phenylisoquinoline (20)

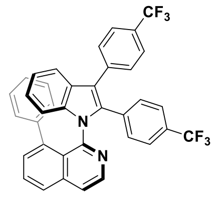

20

^{1}HNMR, $CDCl_3$, 400 MHz

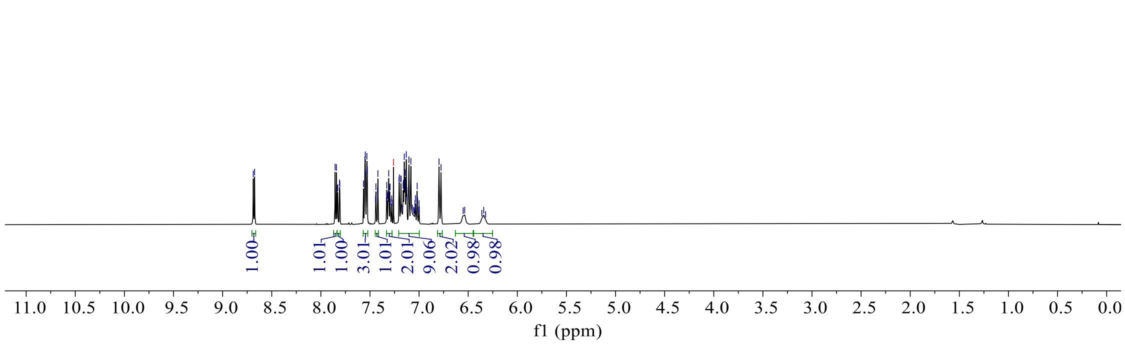

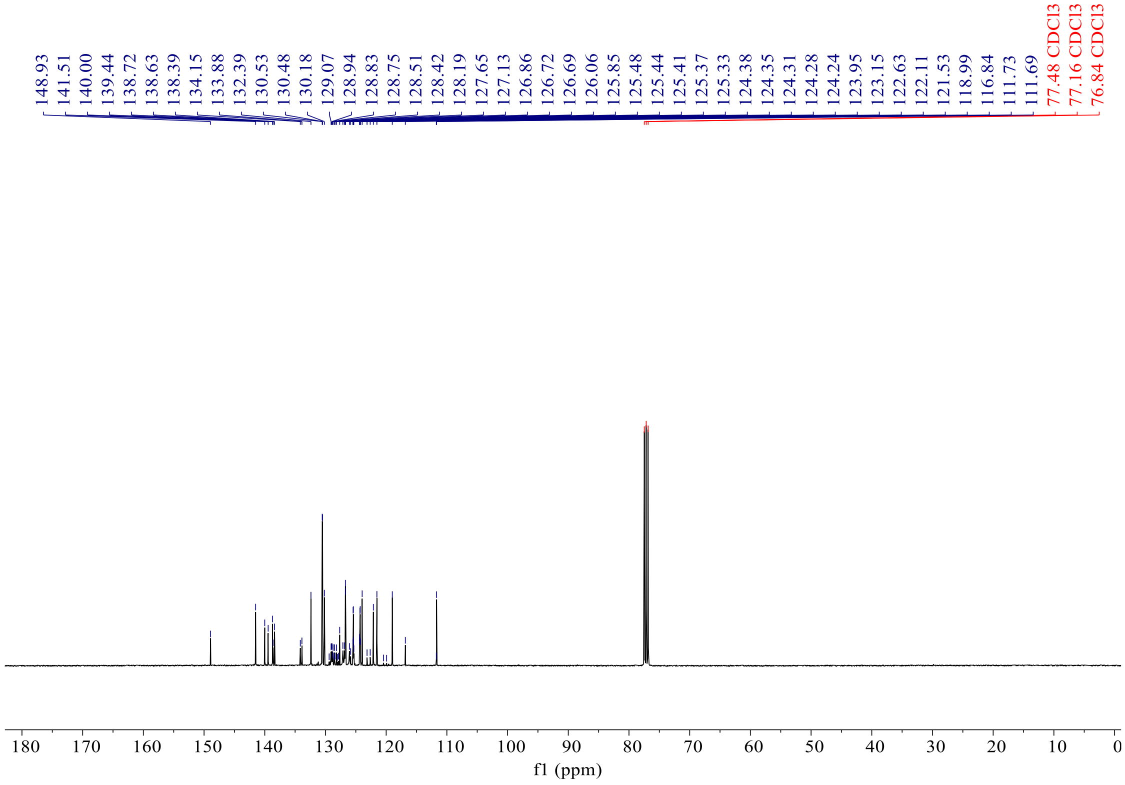

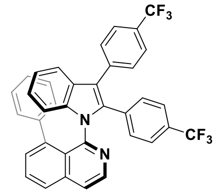

20

^{19}FNMR, $CDCl_3$, 377 MHz

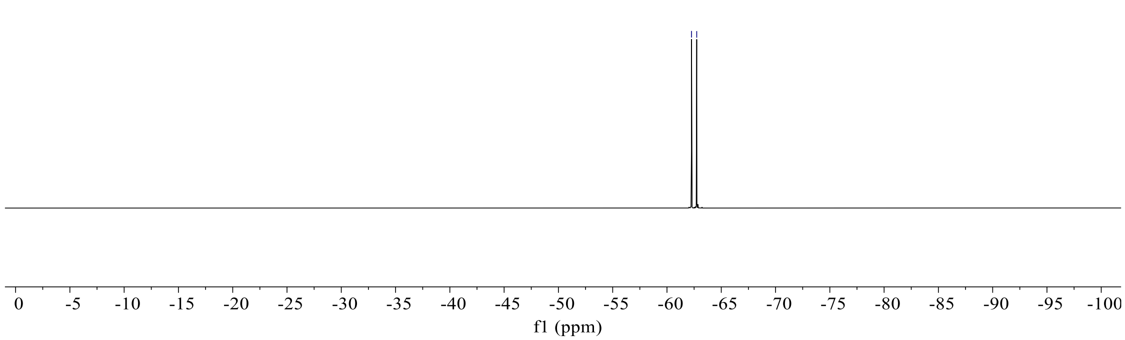

Dimethyl 4,4'-(1-(8-phenylisoquinolin-1-yl)-1*H*-indole-2,3-diyl) (*S*)-dibenzoate (21)

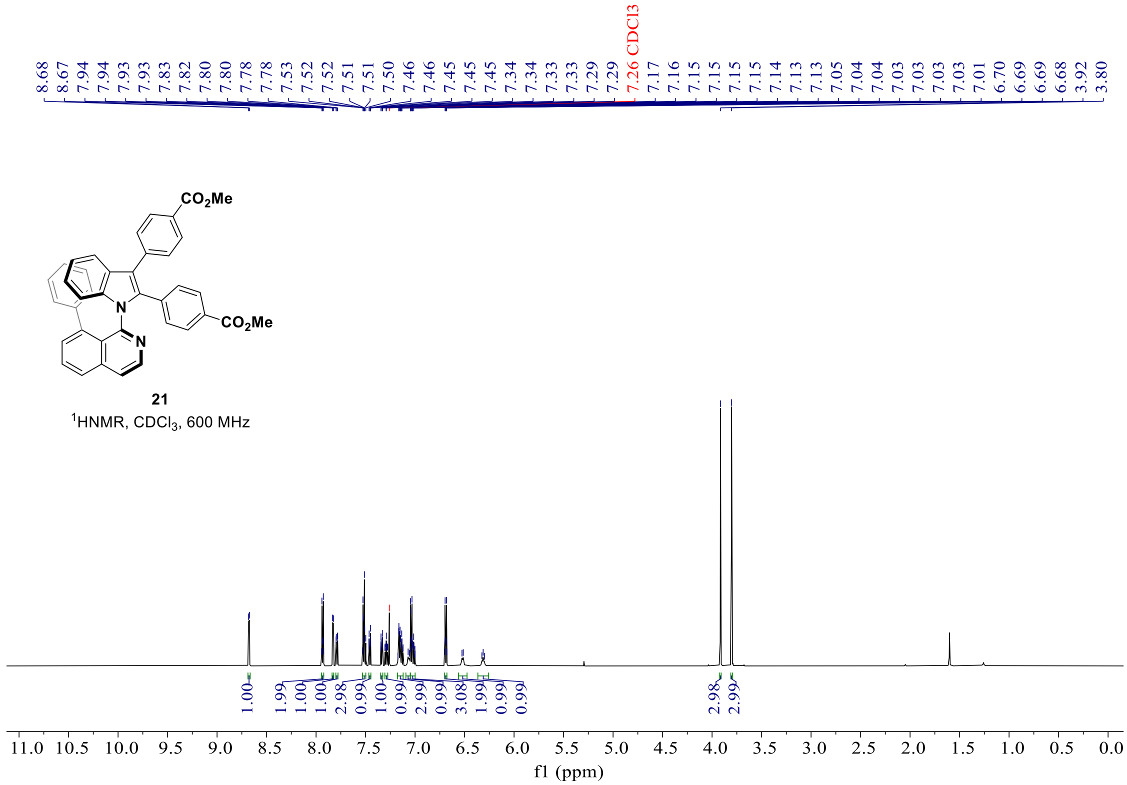

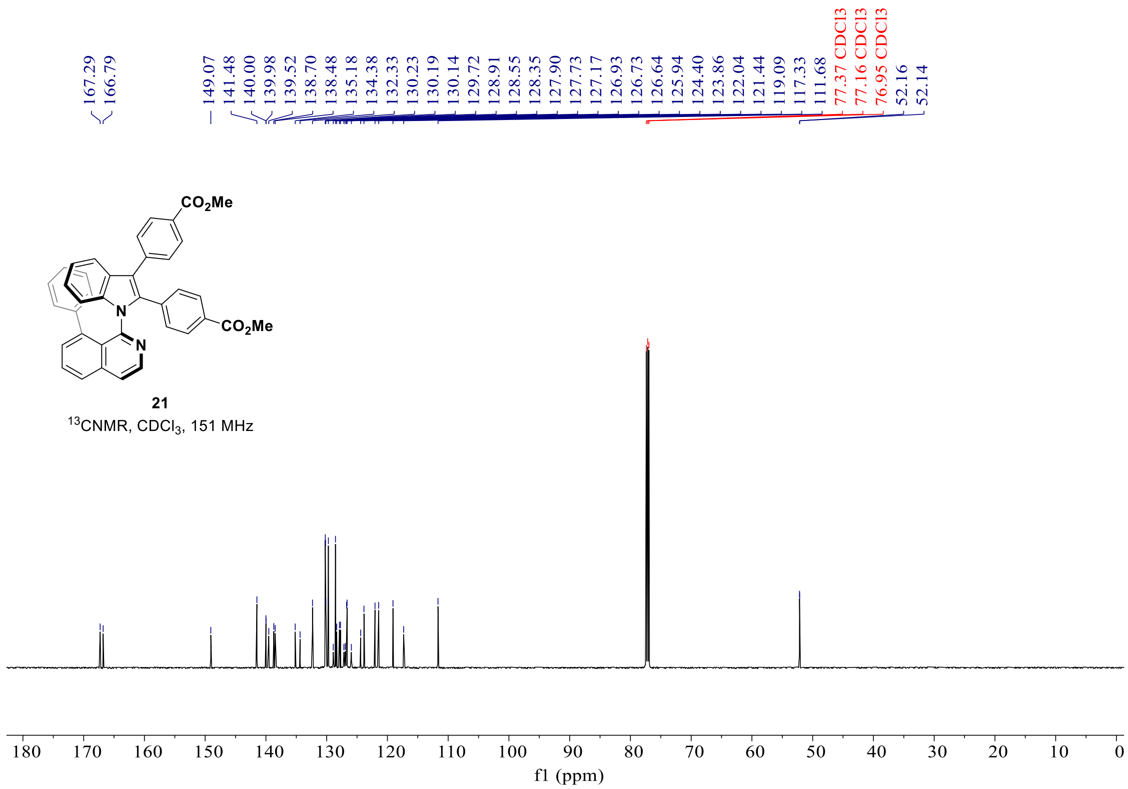

(*S*)-1-(2,3-bis(4-fluorophenyl)-1*H*-indol-1-yl)-8-phenylisoquinoline (22)

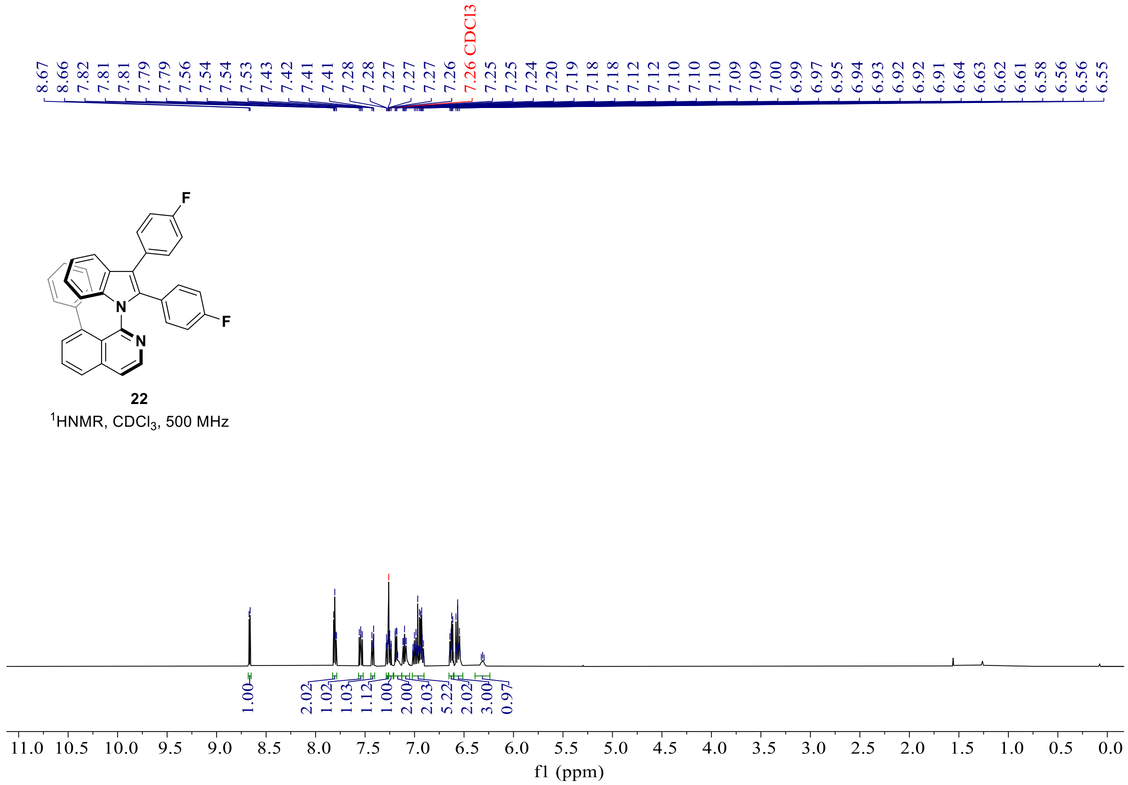

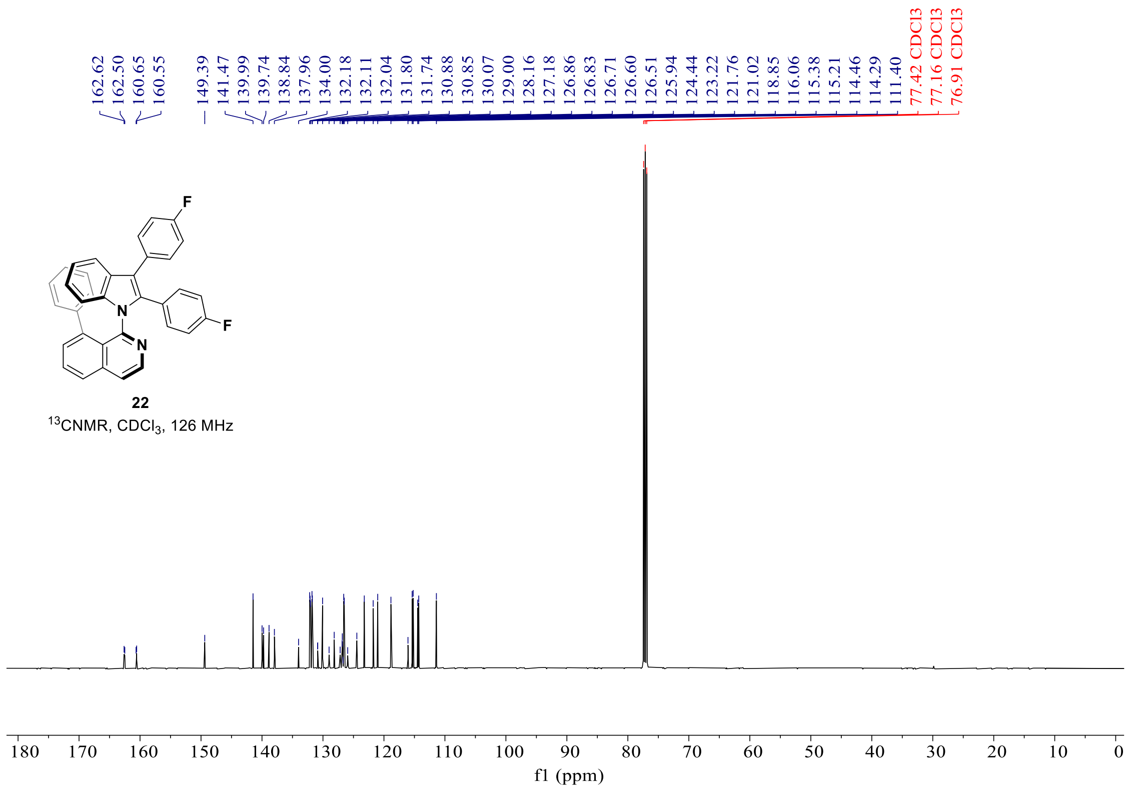

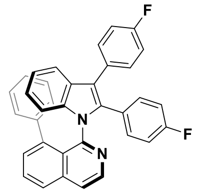

22

^{19}FNMR, $CDCl_3$, 282 MHz

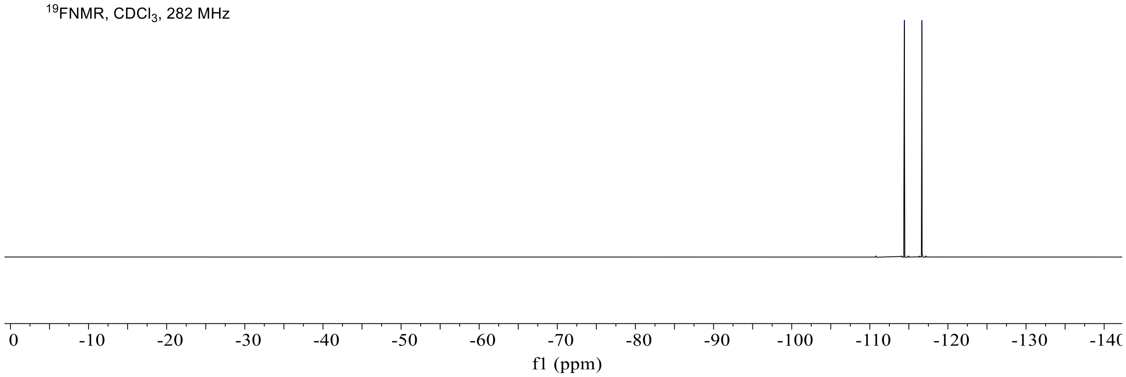

(*S*)-1-(2,3-bis(3-methoxyphenyl)-1*H*-indol-1-yl)-8-phenylisoquinoline (23)

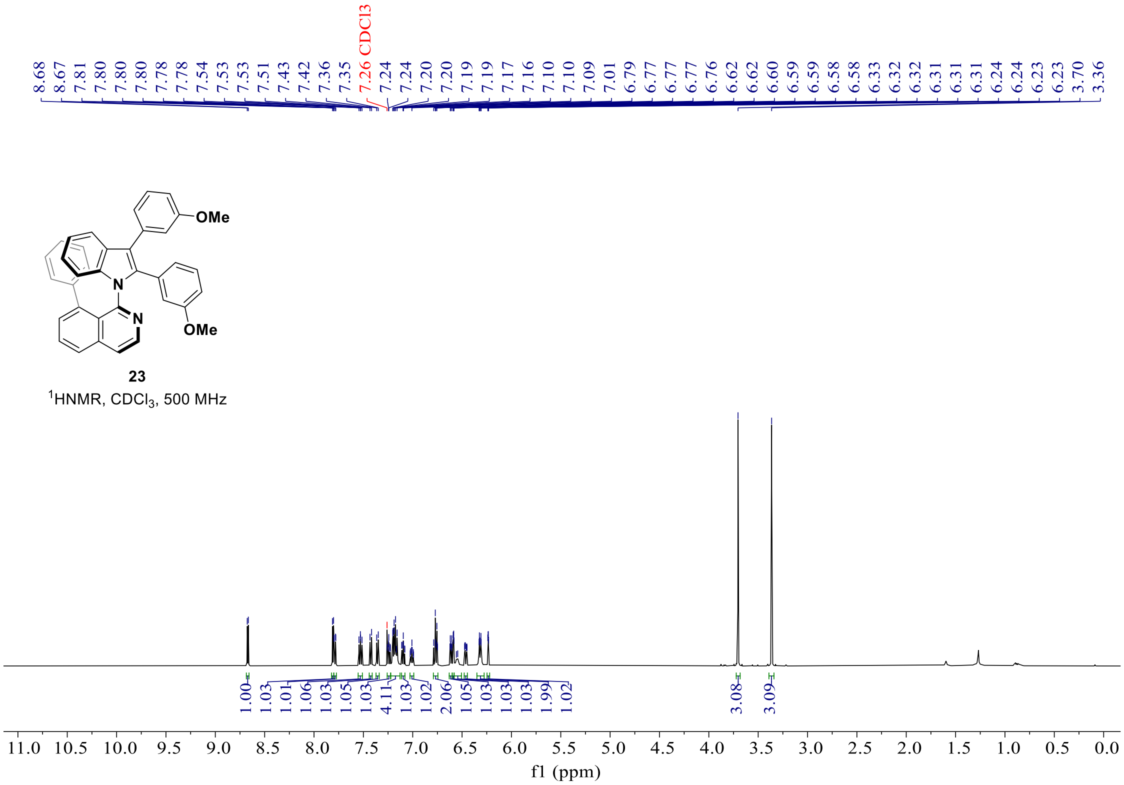

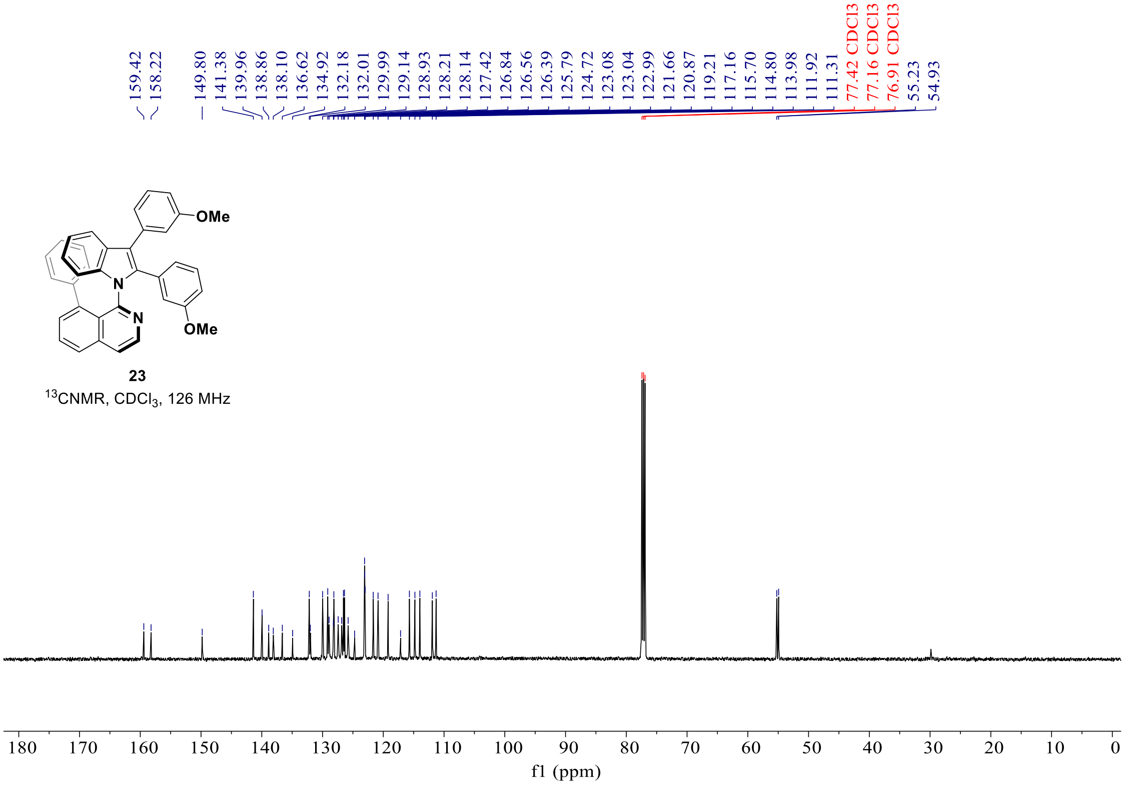

(*S*)-1-(2,3-bis(3-chlorophenyl)-1*H*-indol-1-yl)-8-phenylisoquinoline (24)

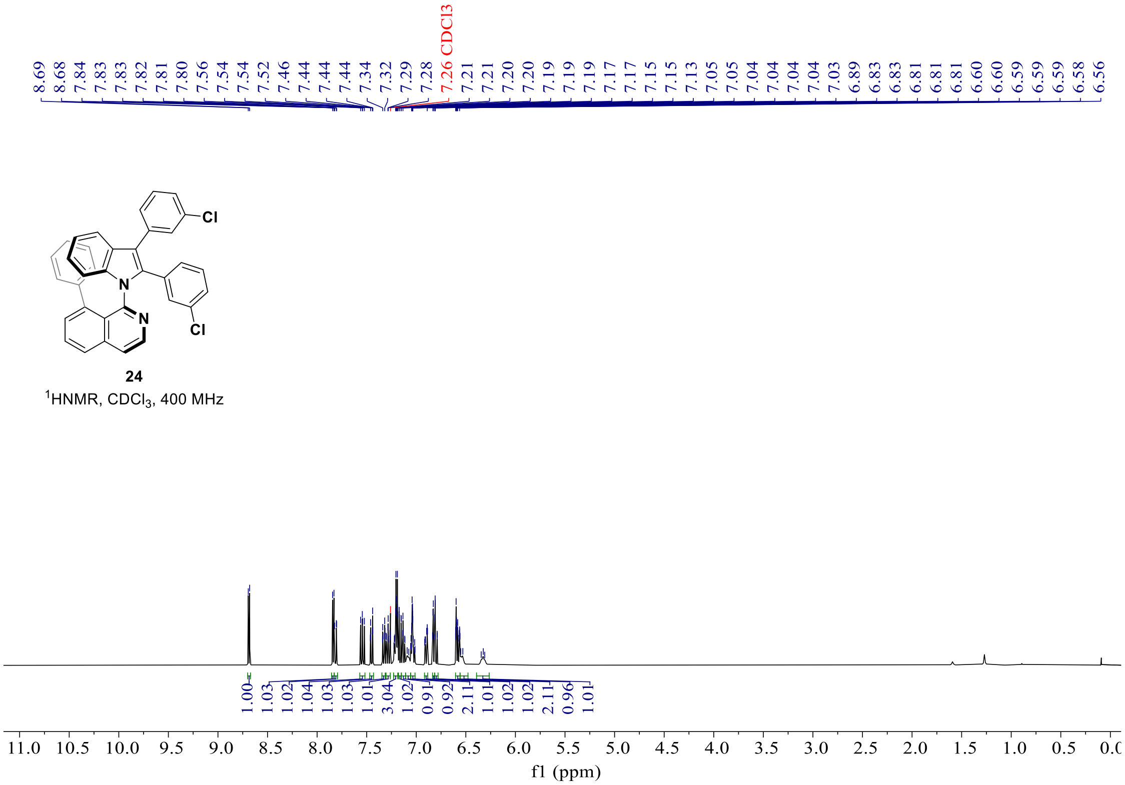

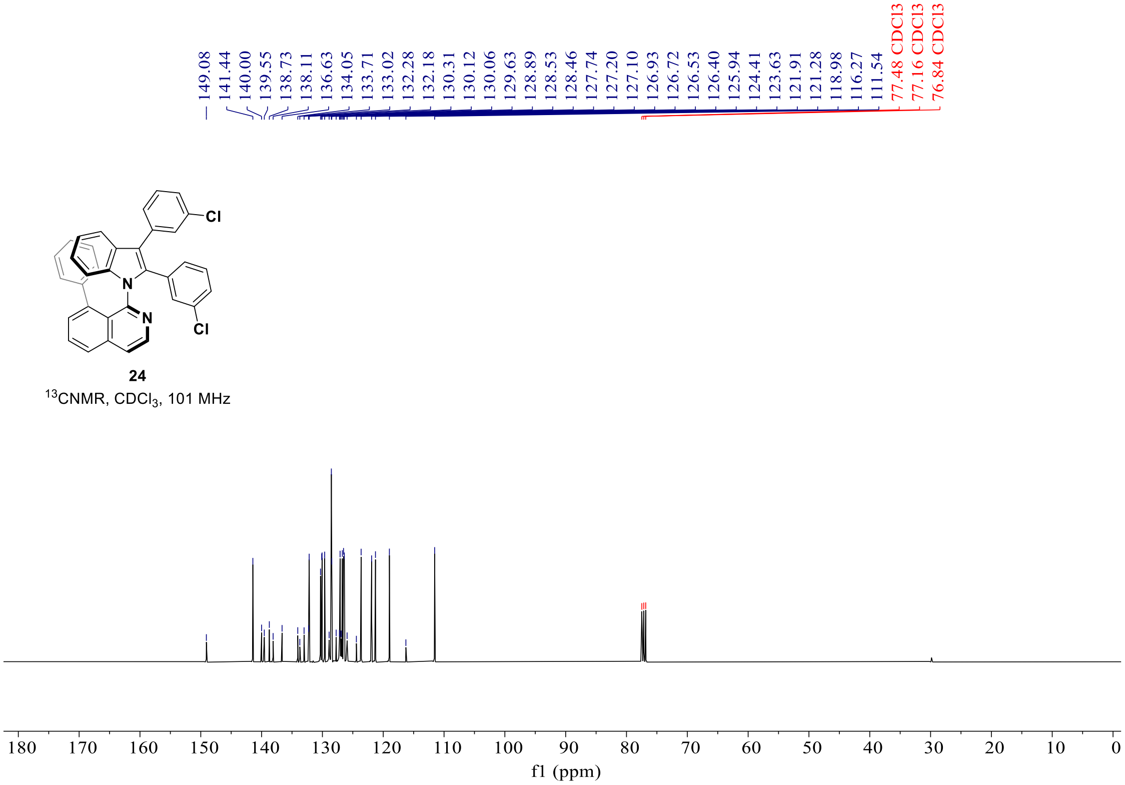

(*S*)-1-(2,3-bis(2-fluorophenyl)-1*H*-indol-1-yl)-8-phenylisoquinoline (25)

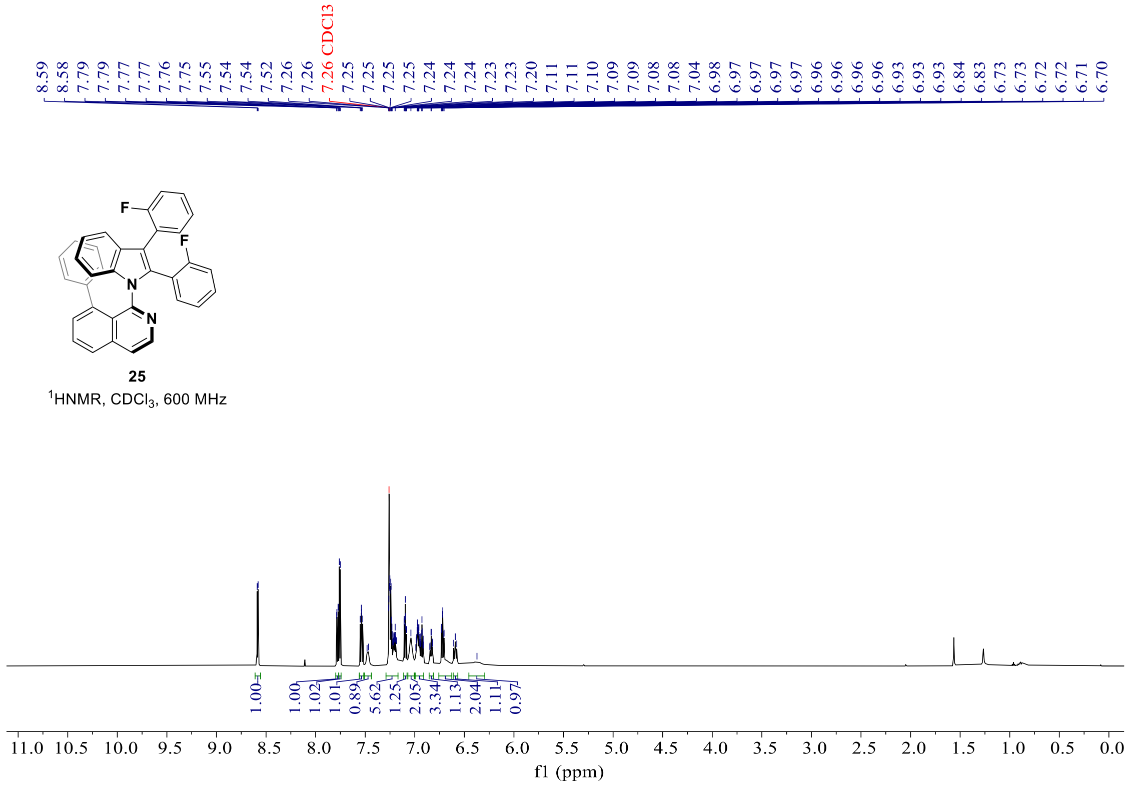

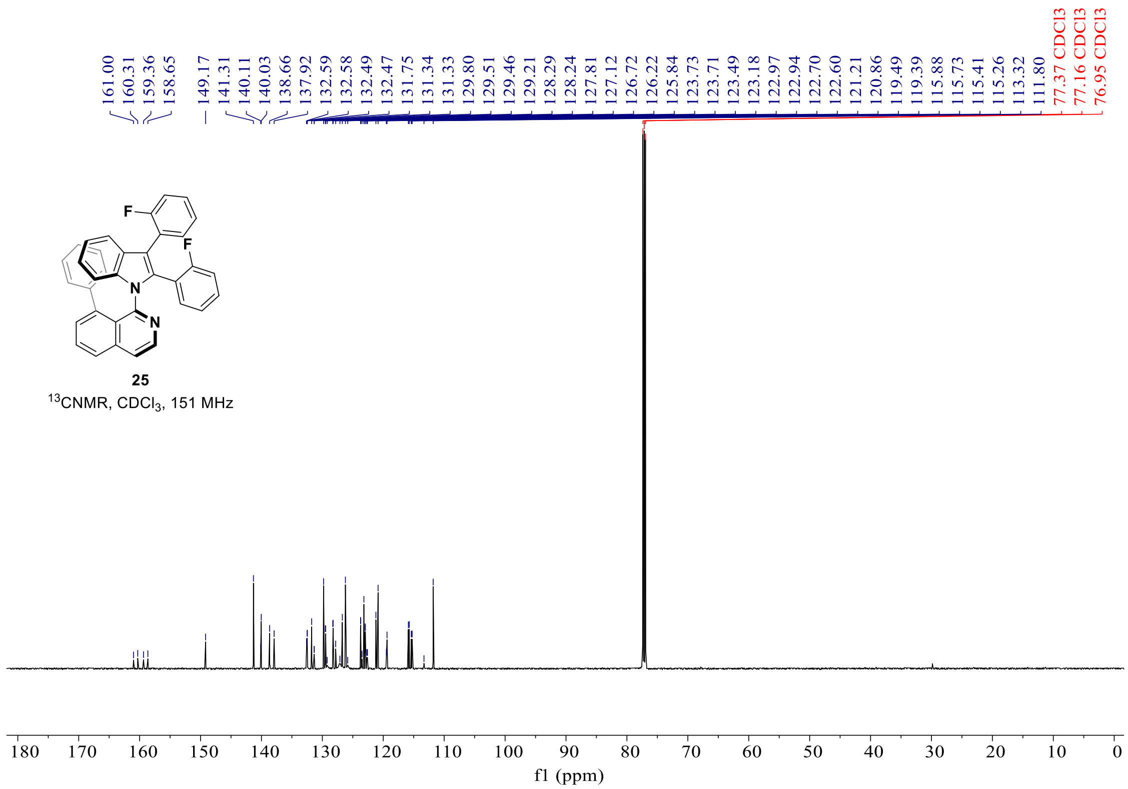

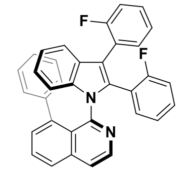

25

^{19}FNMR, $CDCl_3$, 565 MHz

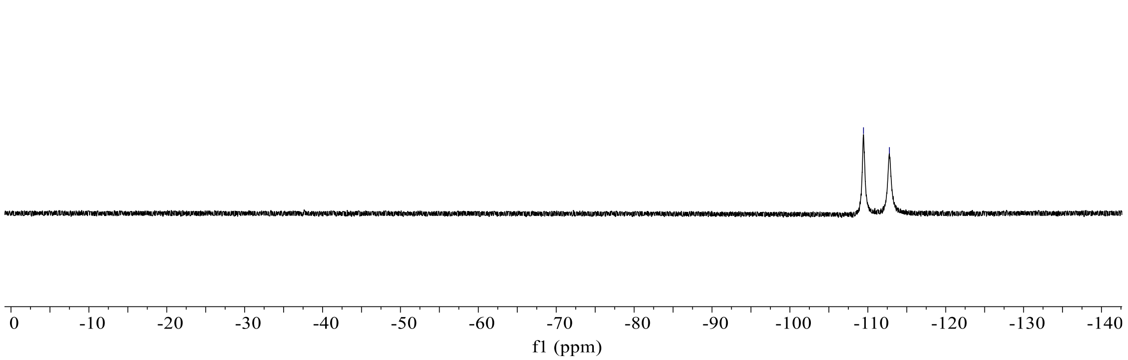

(*S*)-1-(2,3-di(thiophen-2-yl)-1*H*-indol-1-yl)-8-phenylisoquinoline (26)

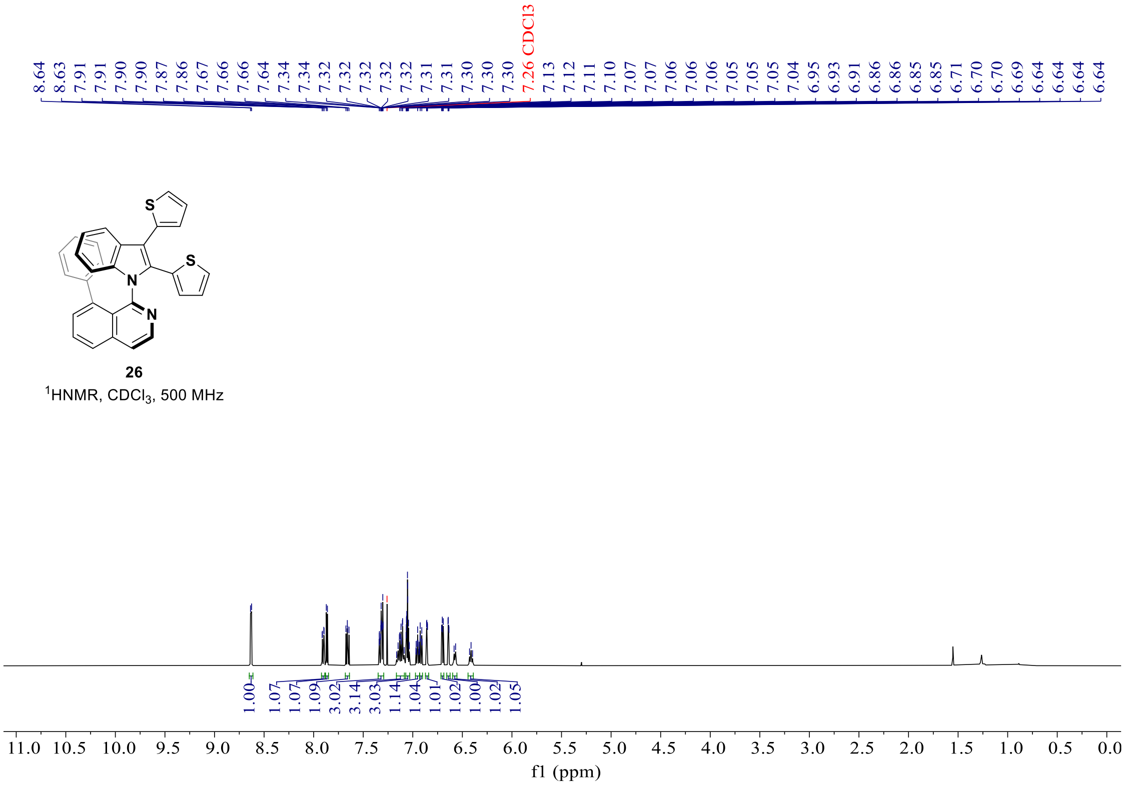

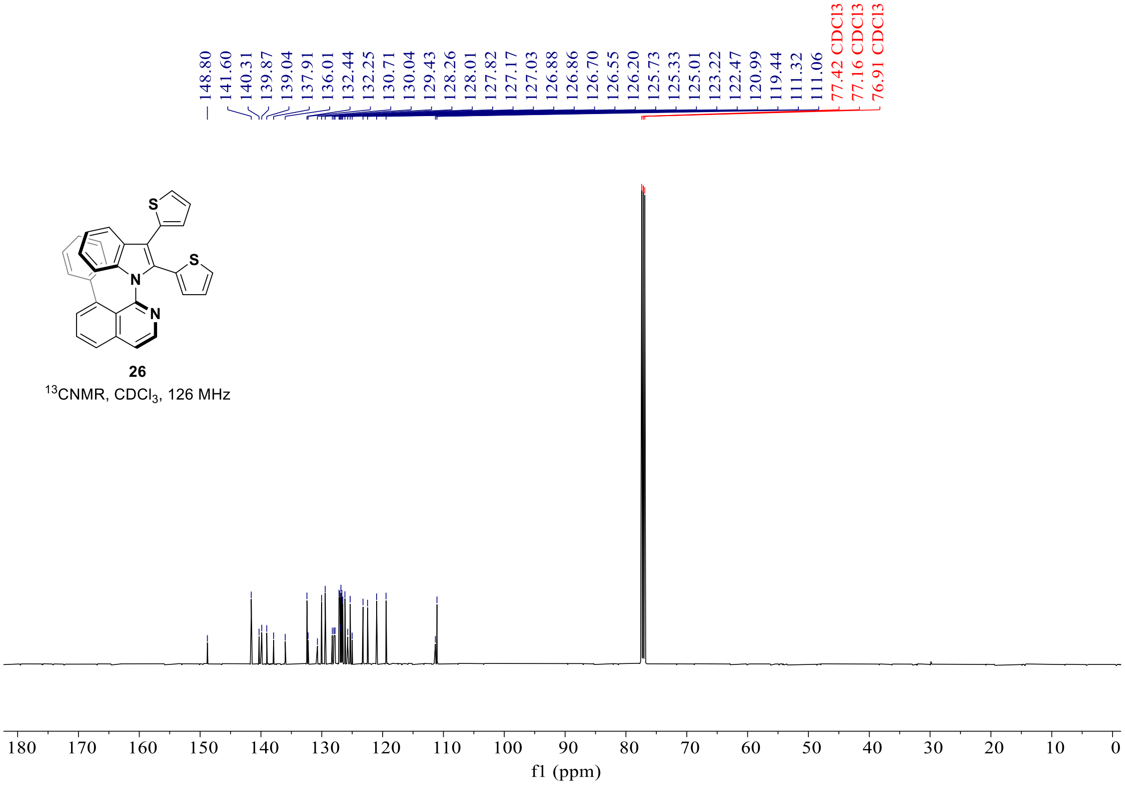

(*R*)-1-(2,3-dibutyl-1*H*-indol-1-yl)-8-phenylisoquinoline (27)

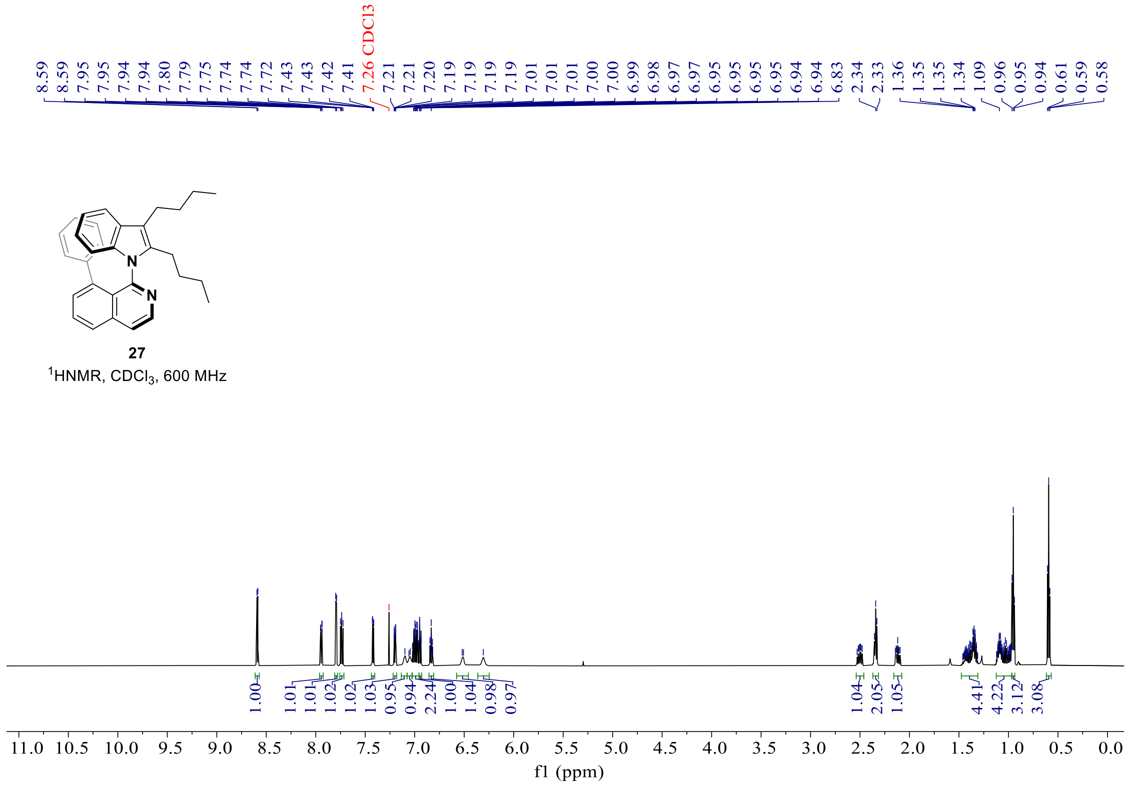

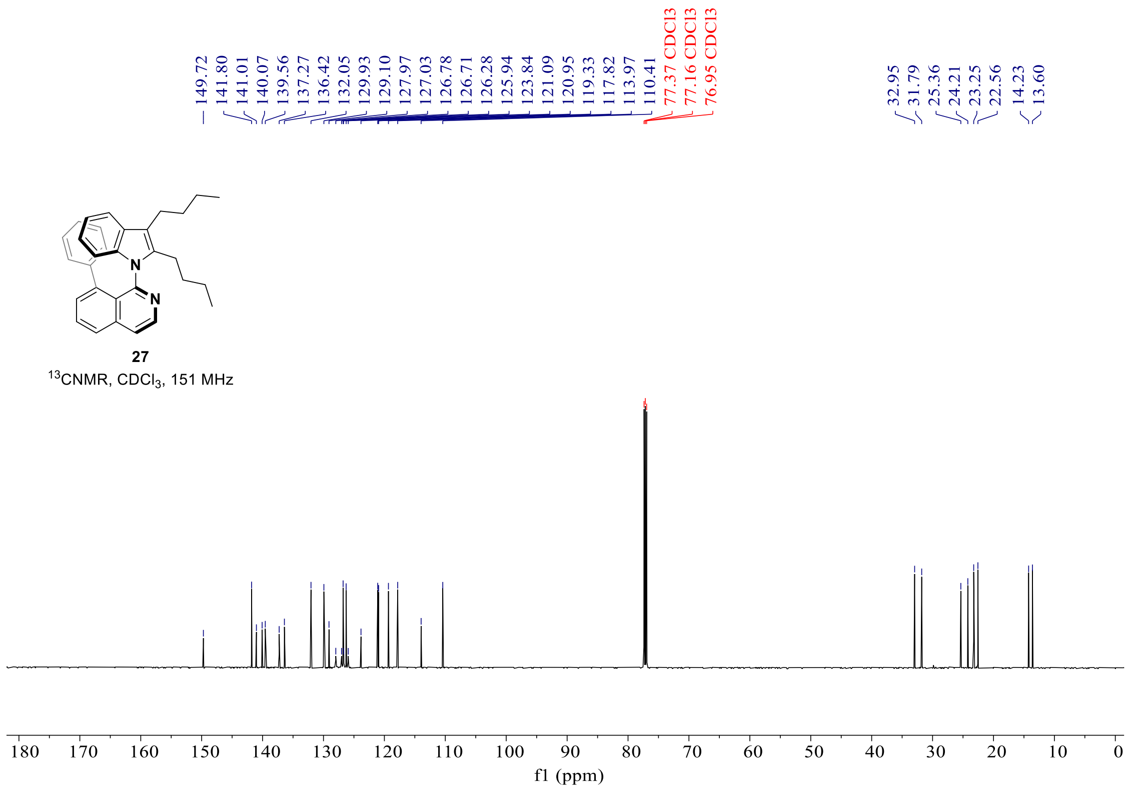

(*S*)-1-(3-butyl-2-phenyl-1*H*-indol-1-yl)-8-phenylisoquinoline (29)

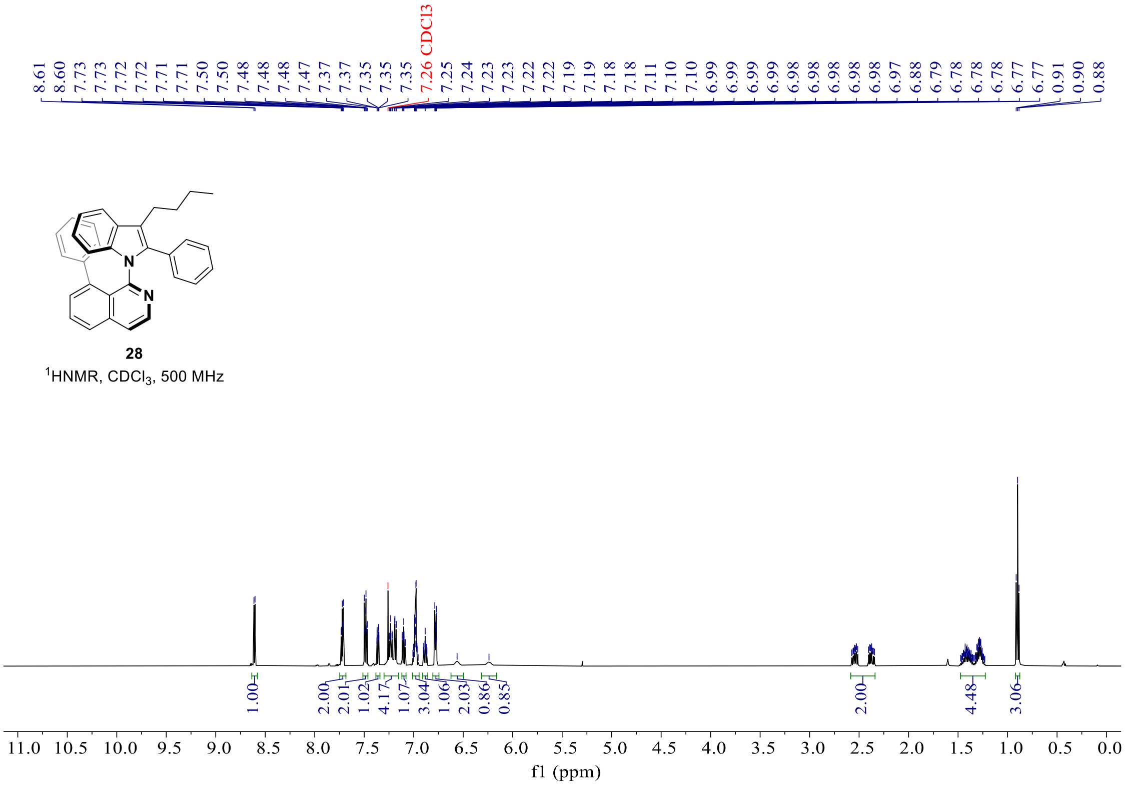

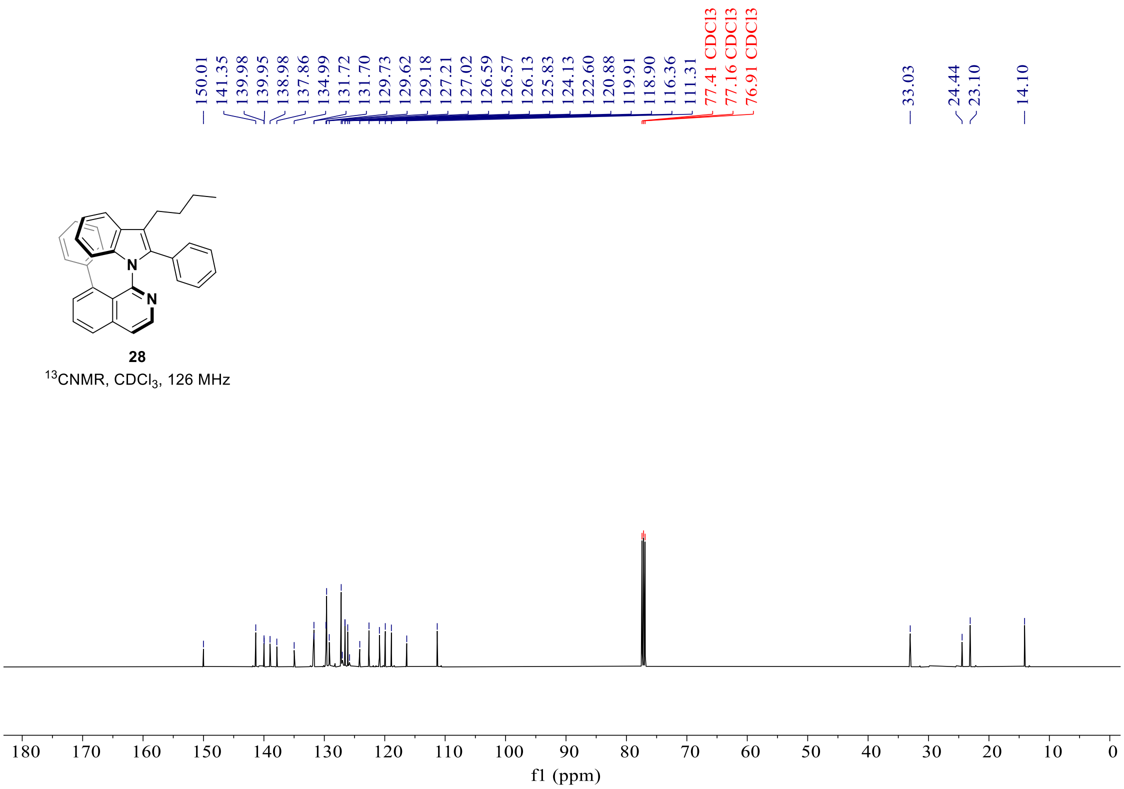

(*R*)-2,4,6,7,9-pentaphenyl-2,3-diazaspiro[4.4]nona-3,6,8-trien-1-one (31)

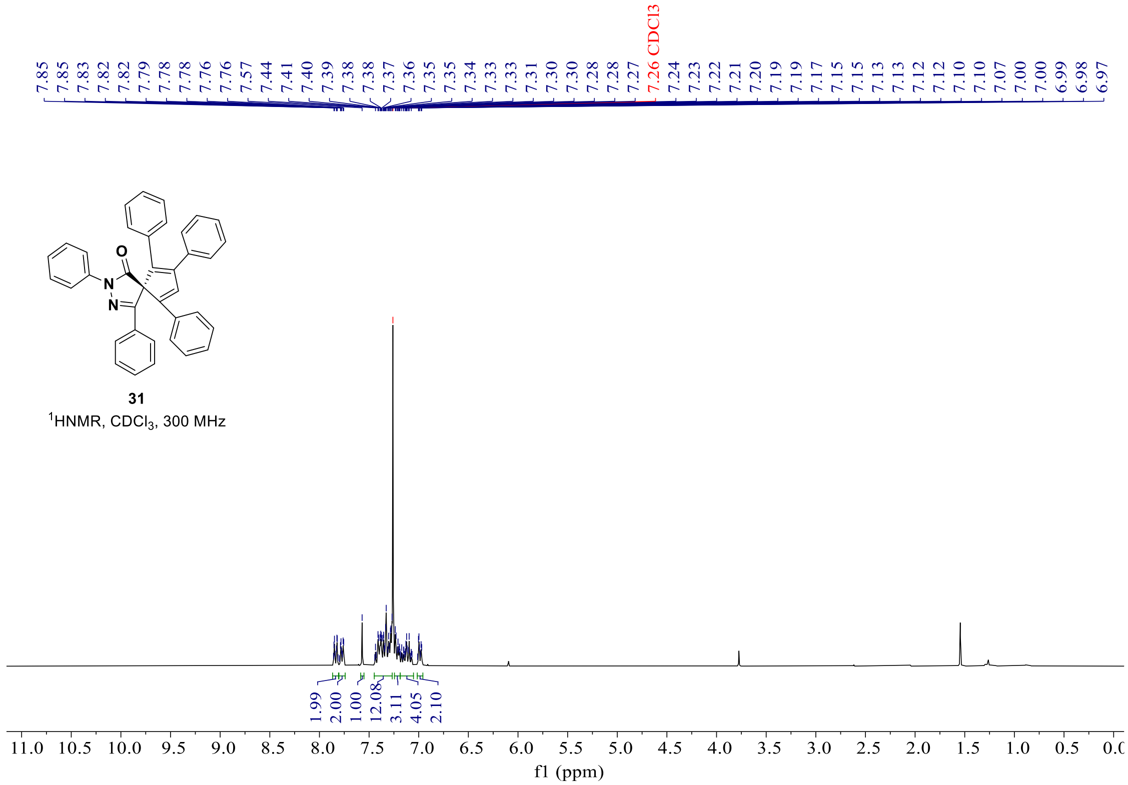

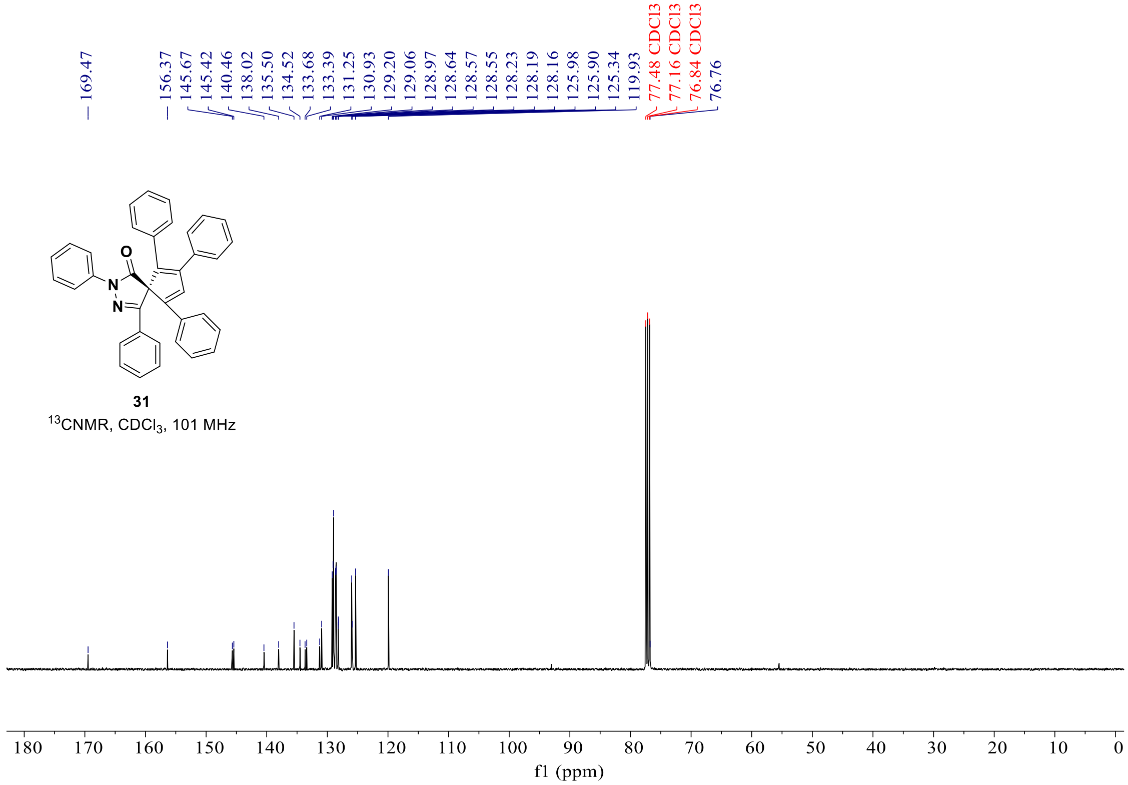

(*R*)-6,7-bis(4-fluorophenyl)-2,4,9-triphenyl-2,3-diazaspiro[4.4]nona-3,6,8-trien-1-one (32)

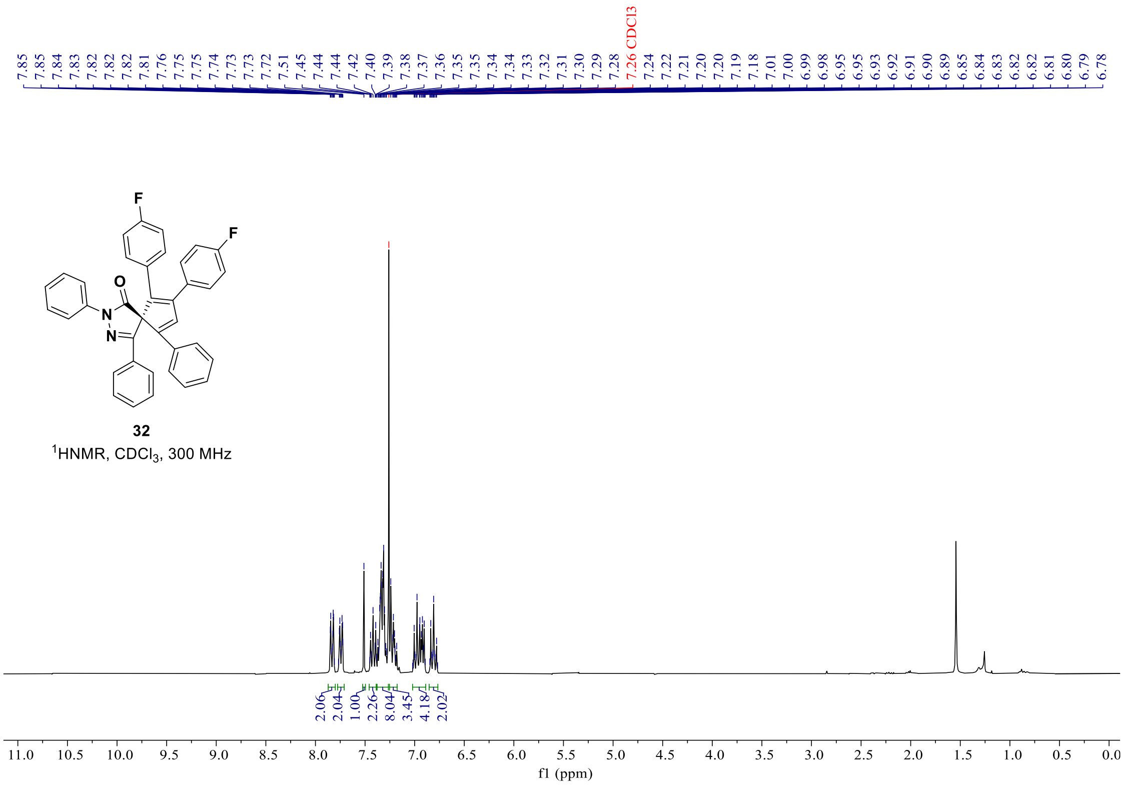

32

^{1}HNMR, $CDCl_3$, 300 MHz

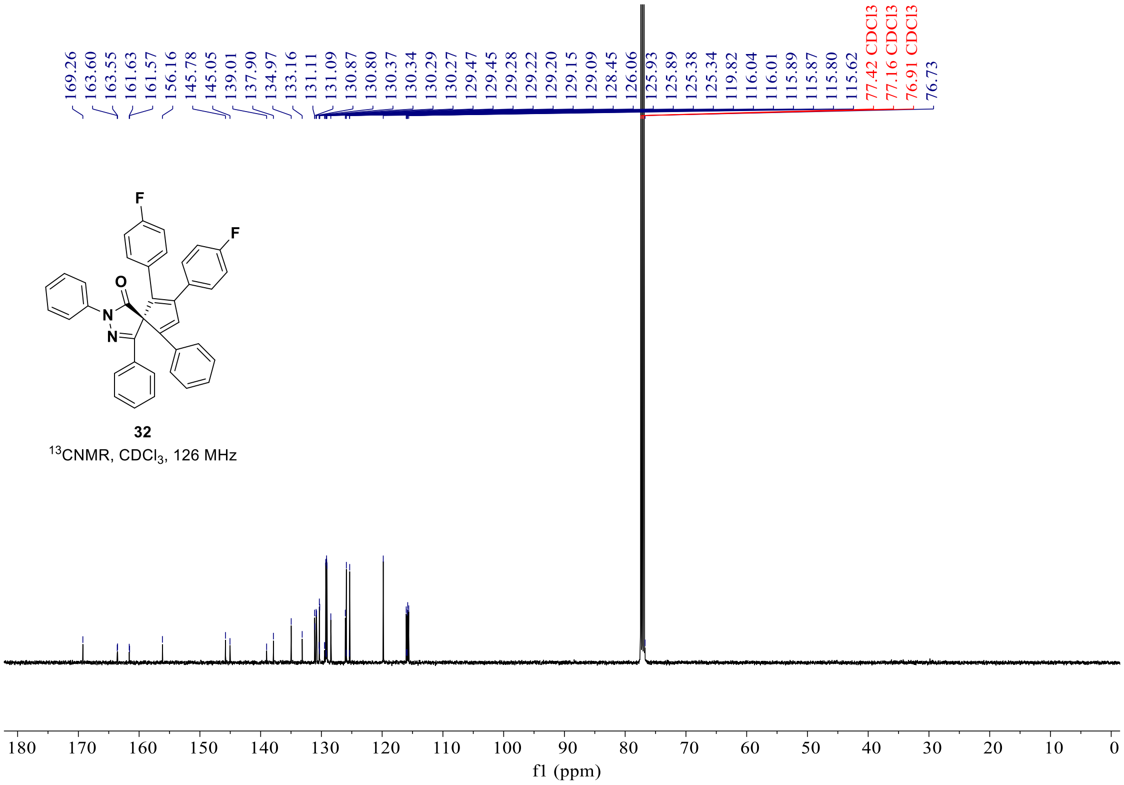

32

^{13}CNMR, $CDCl_3$, 126 MHz

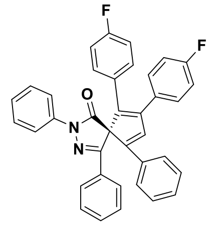

32

^{19}FNMR, $CDCl_3$, 471 MHz

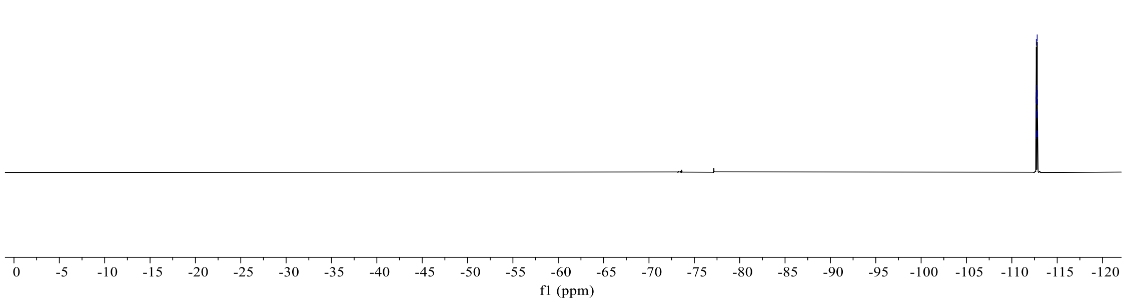

(*R*)-6,7-bis(3-fluorophenyl)-2,4,9-triphenyl-2,3-diazaspiro[4.4]nona-3,6,8-trien-1-one (33)

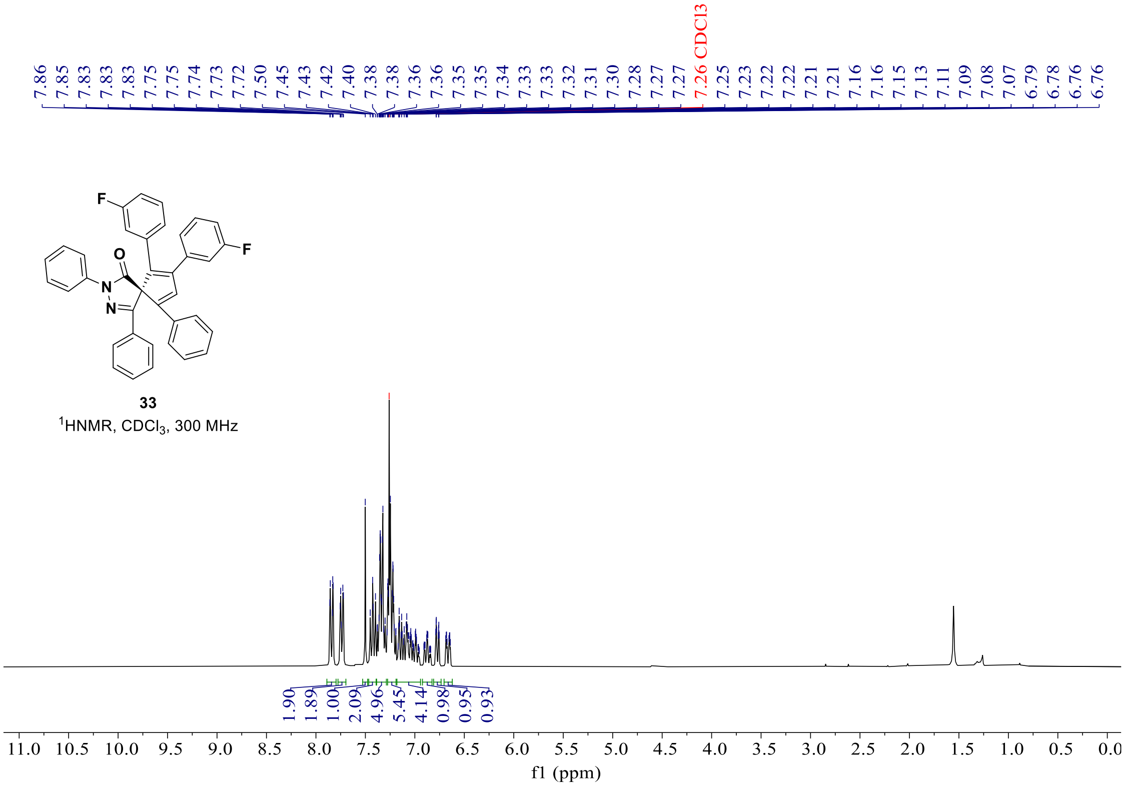

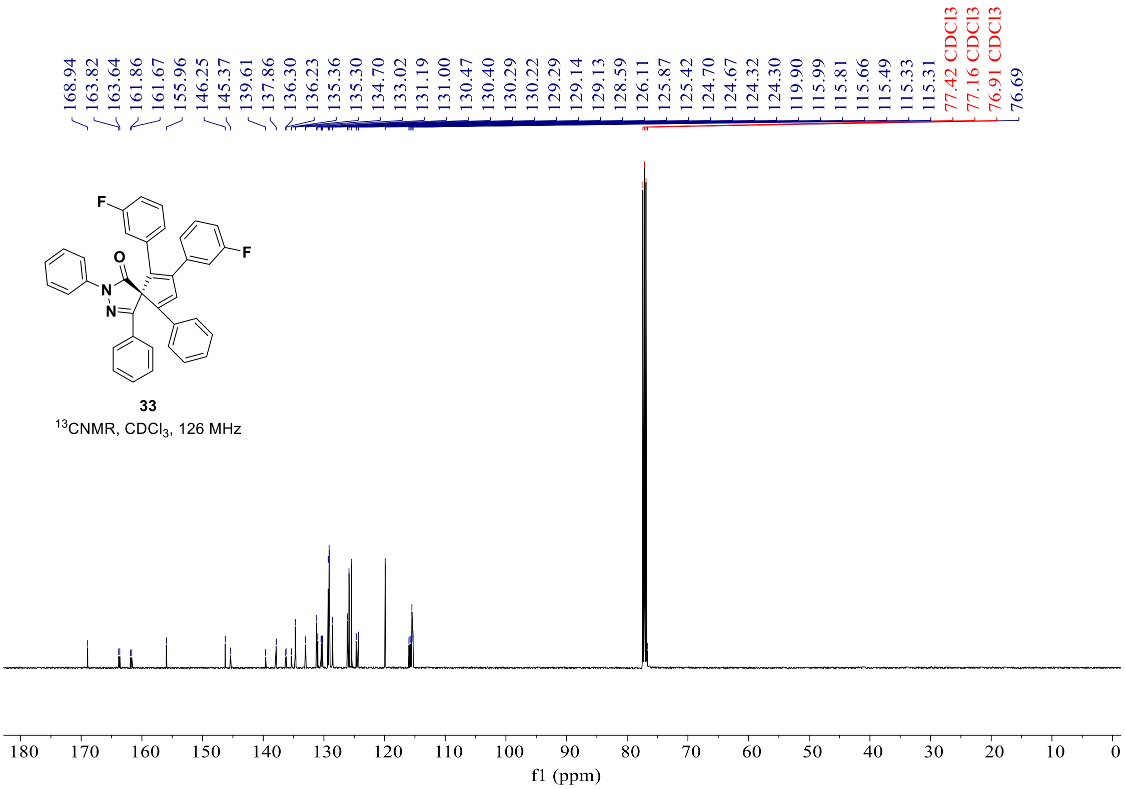

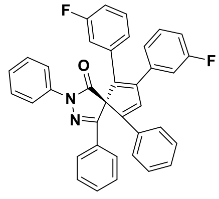

33

^{19}FNMR, $CDCl_3$, 471 MHz

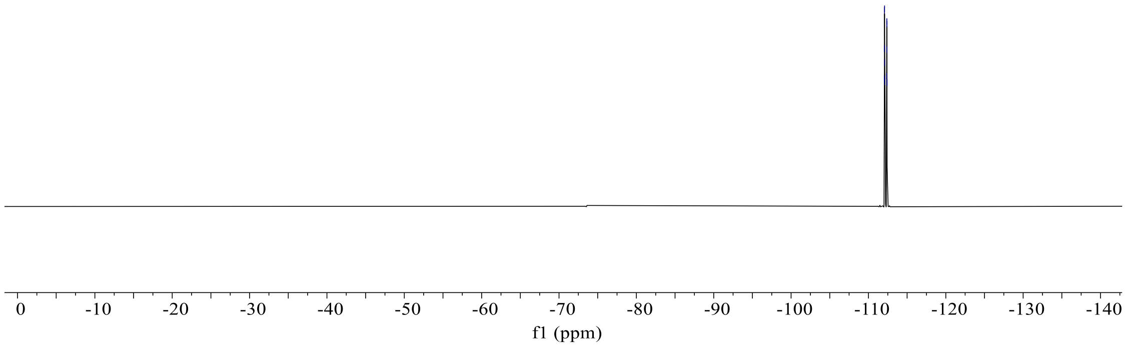

(*R*)-6,7-bis(3-chlorophenyl)-2,4,9-triphenyl-2,3-diazaspiro[4.4]nona-3,6,8-trien-1-one (34)

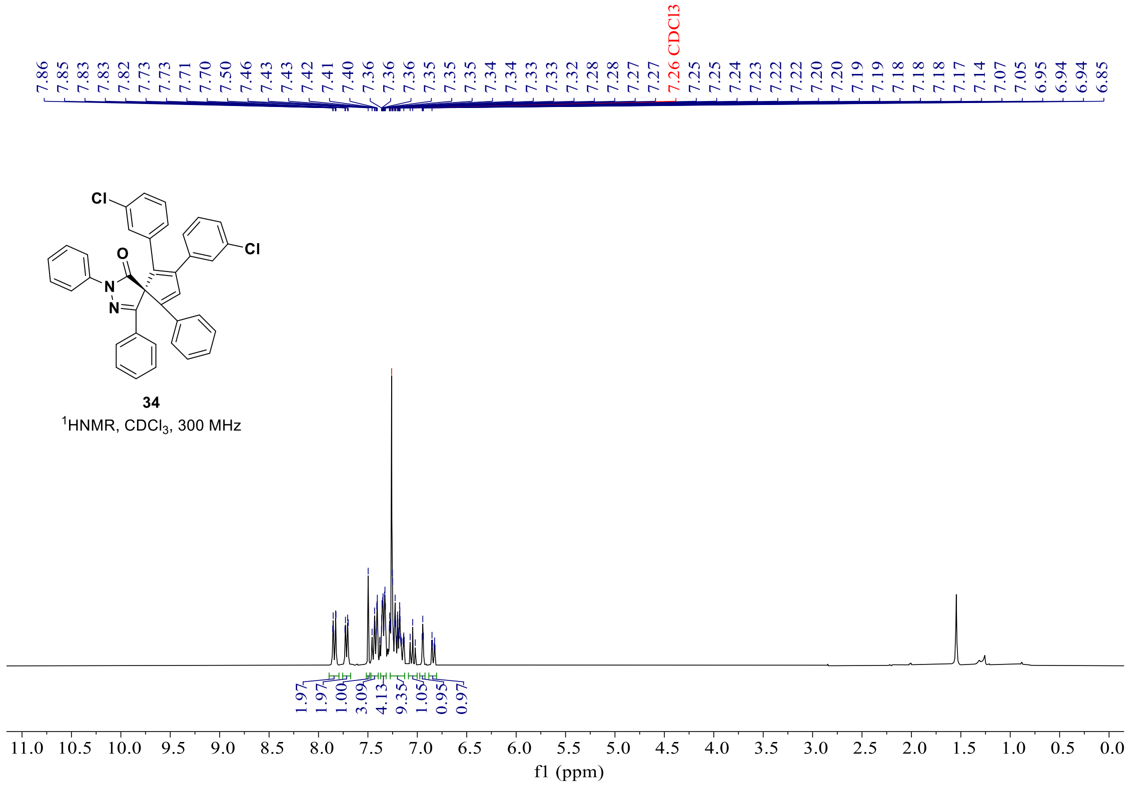

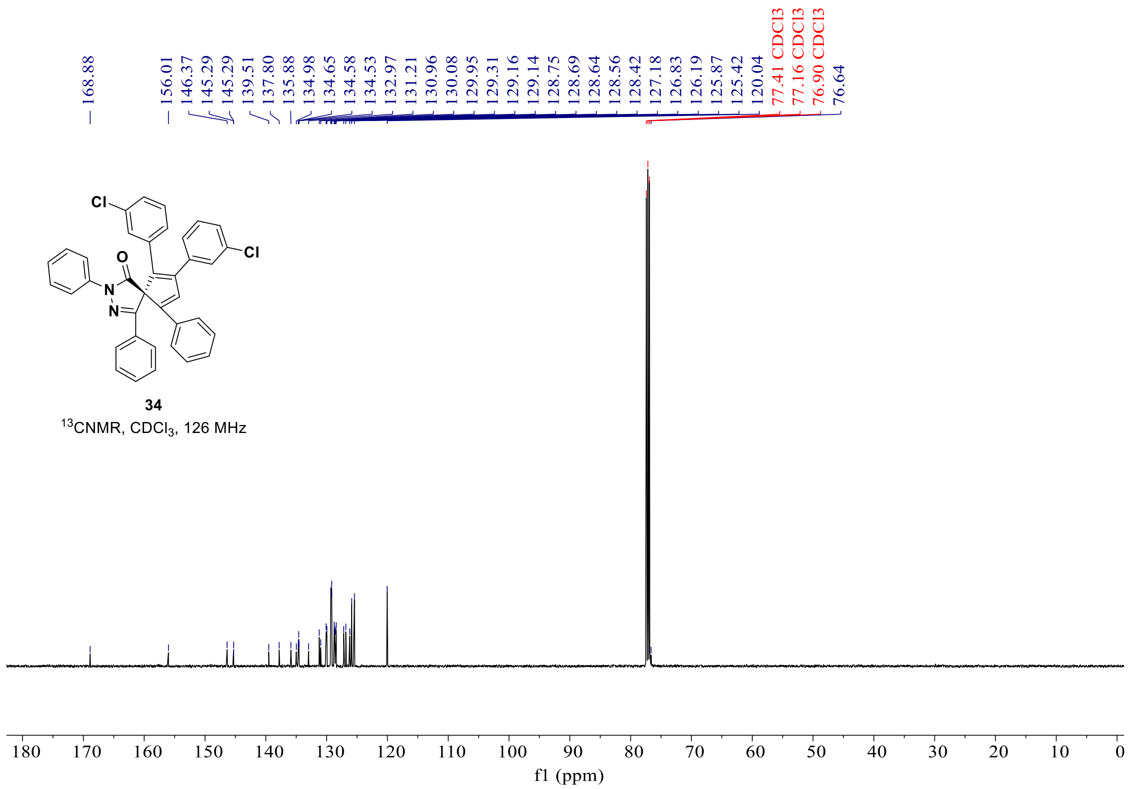

(*R*)-2,4,9-triphenyl-6,7-bis(3-(trifluoromethyl)phenyl)-2,3-diazaspiro[4.4]nona-3,6,8-trien-1-one (35)

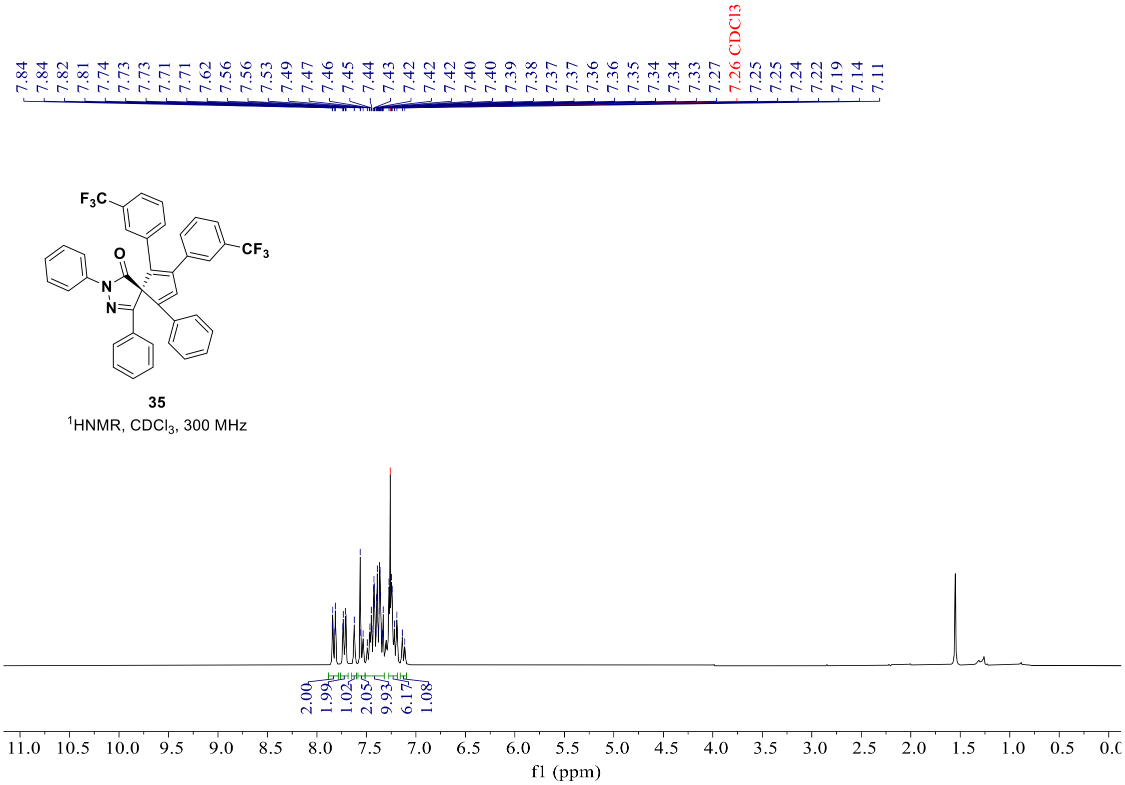

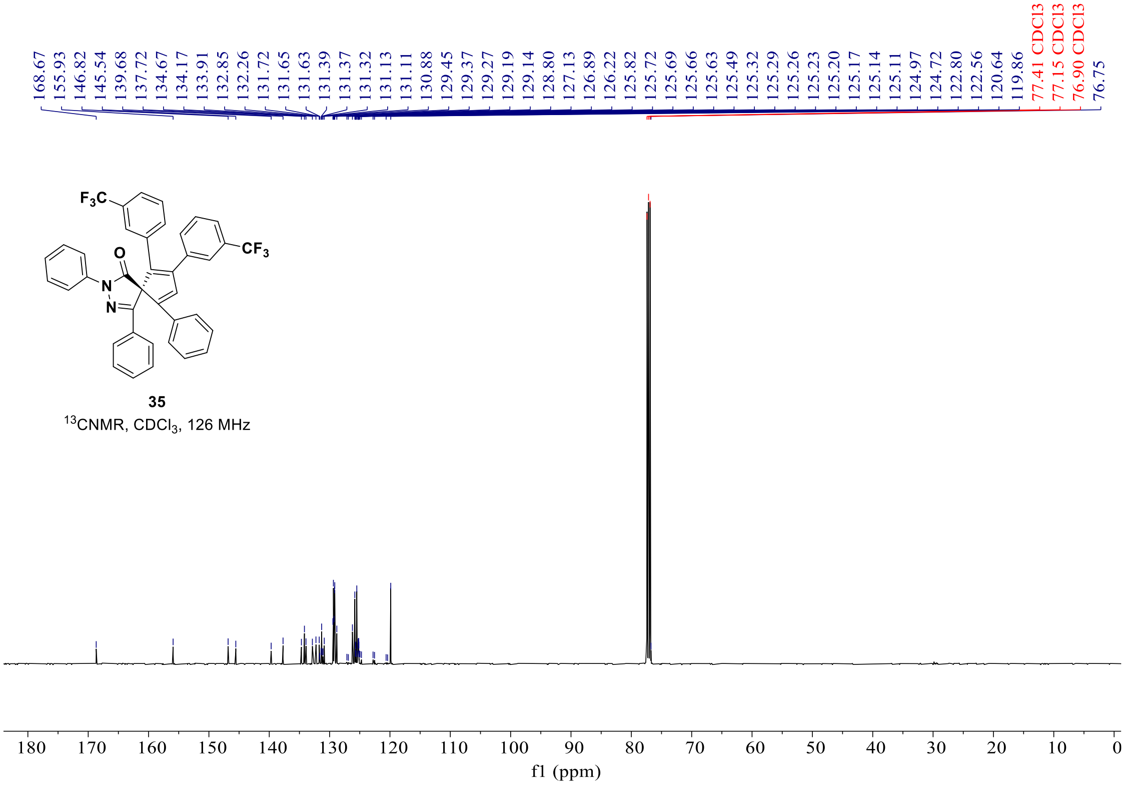

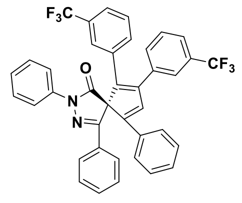

35

^{19}FNMR, $CDCl_3$, 471 MHz

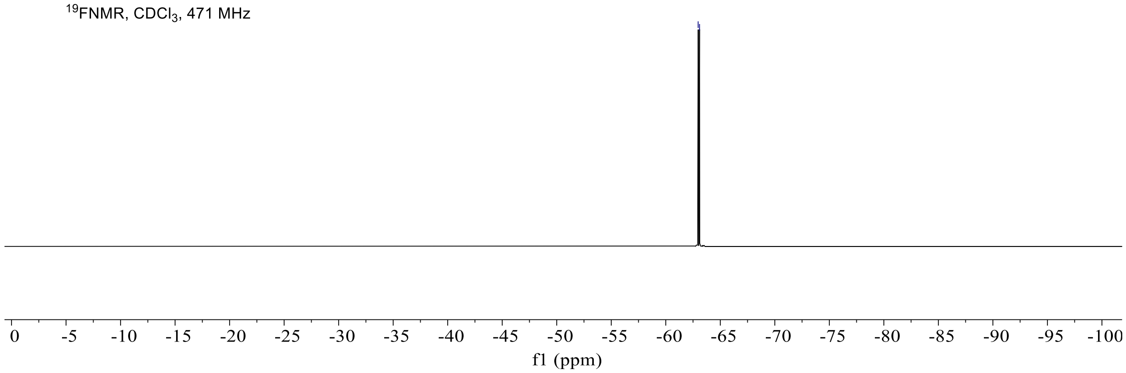

(*R*)-1,1'-((4-oxo-1,3,9-triphenyl-2,3-diazaspiro[4.4]nona-1,6,8-triene-6,7-diyl)bis(3,1-phenylene))bis(ethan-1-one) (36)

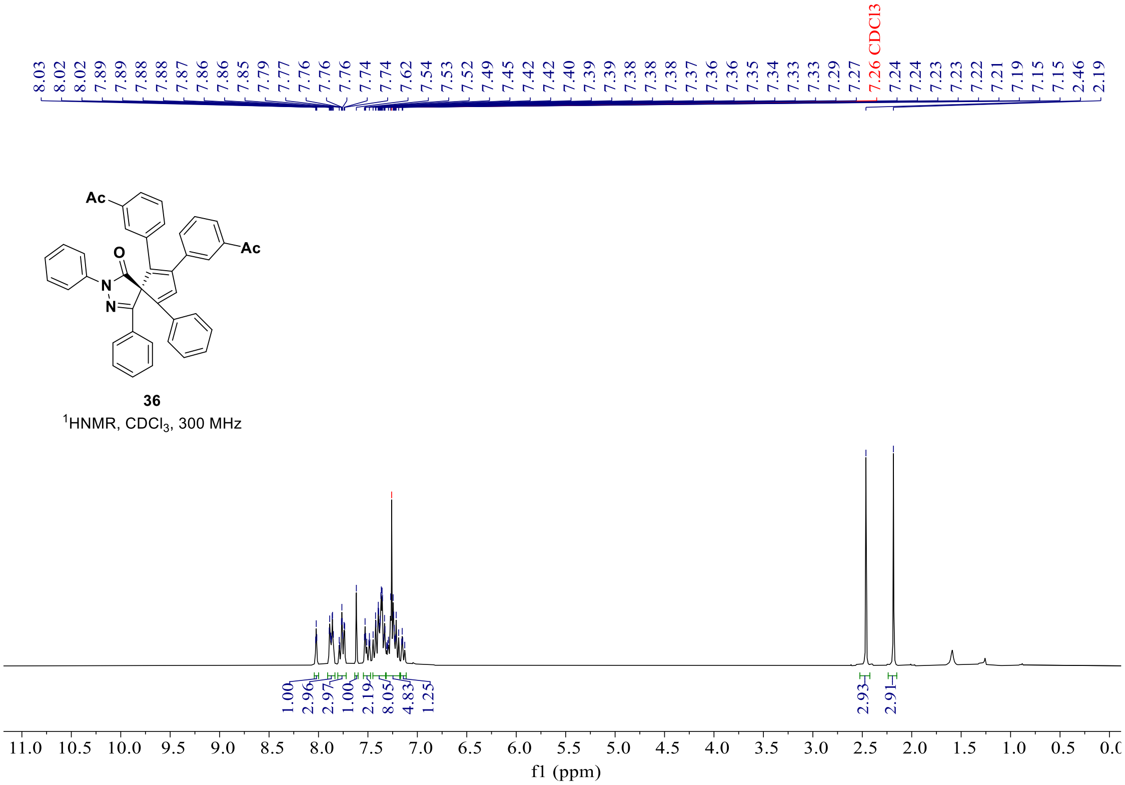

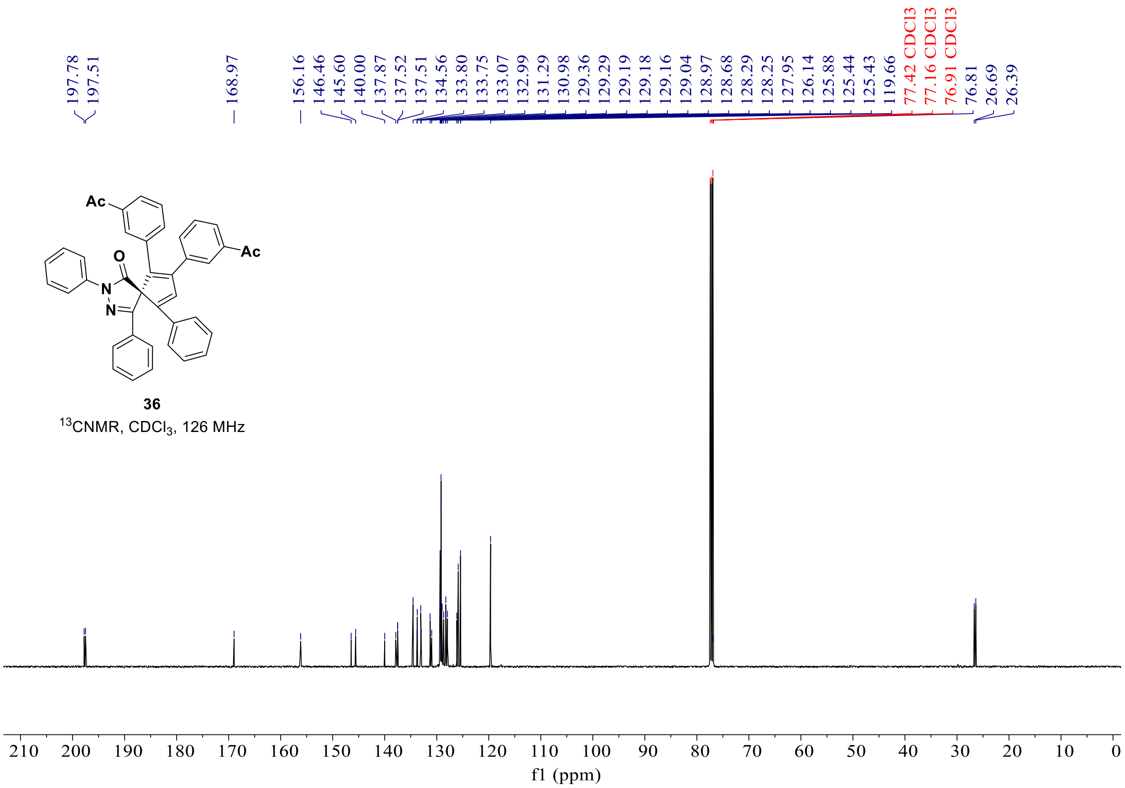

(*R*)-4-(4-fluorophenyl)-2,6,7,9-tetraphenyl-2,3-diazaspiro[4.4]nona-3,6,8-trien-1-one (37)

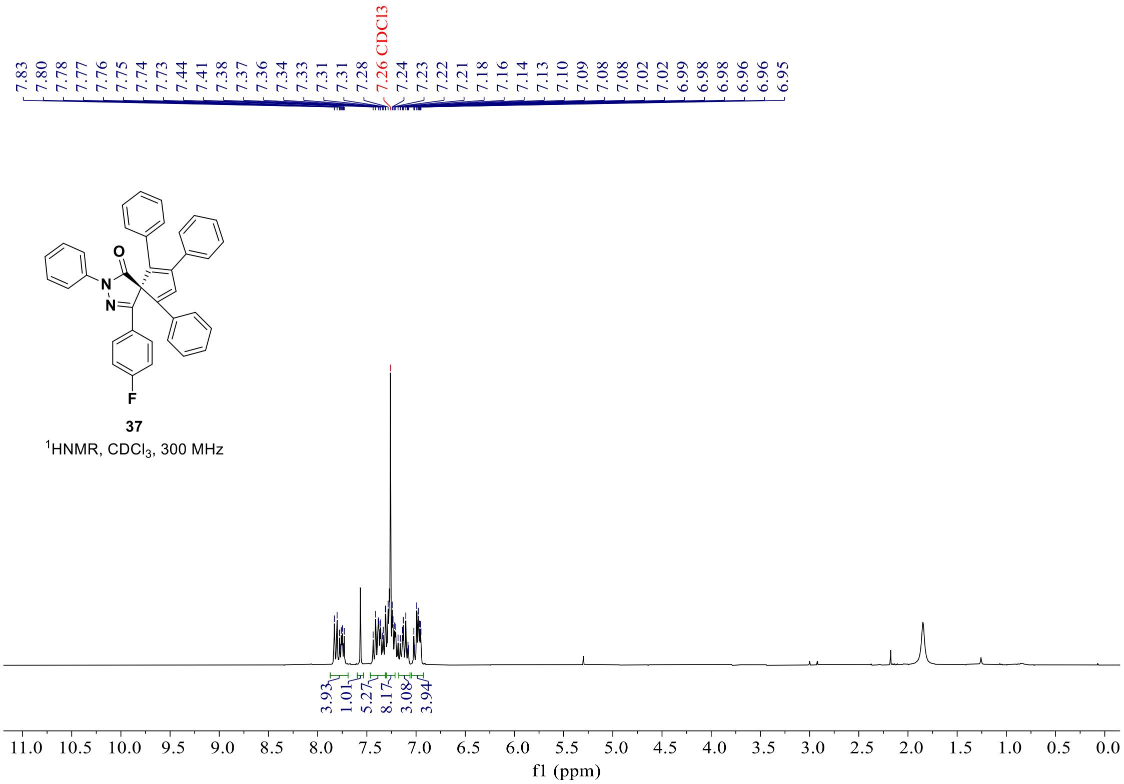

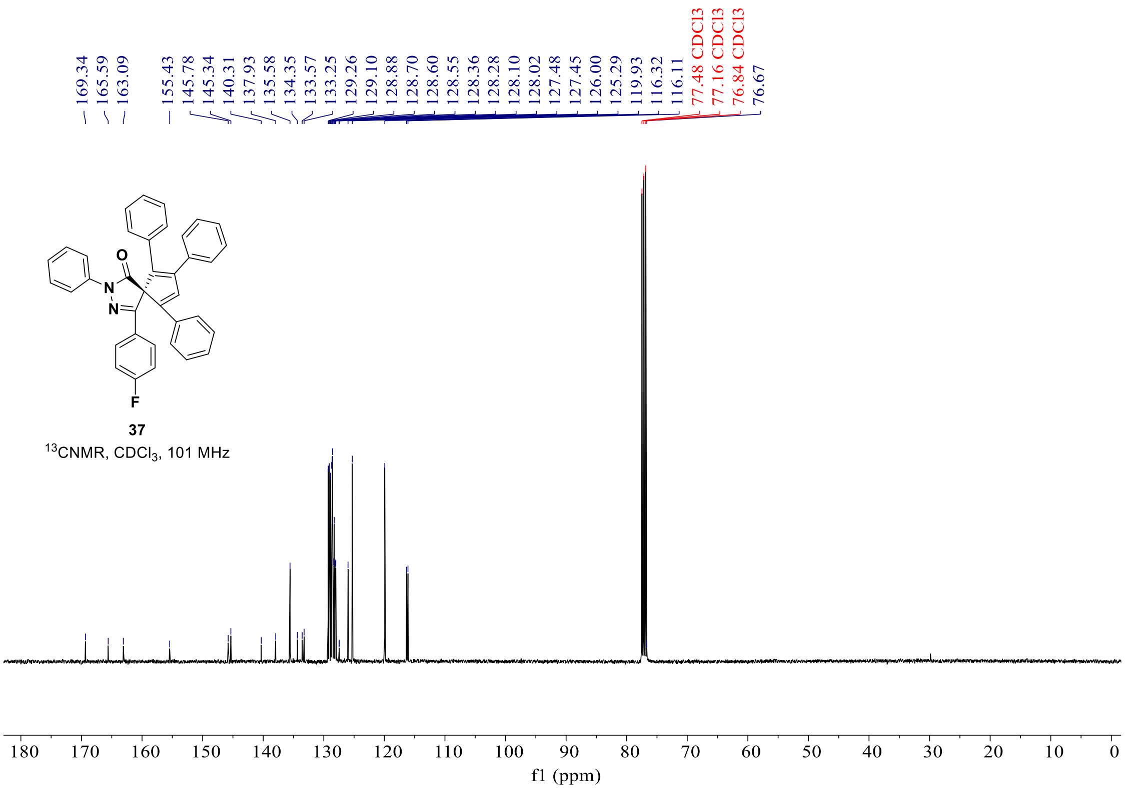

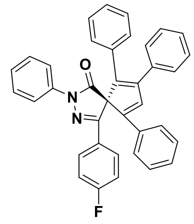

37

^{19}FNMR, $CDCl_3$, 377 MHz

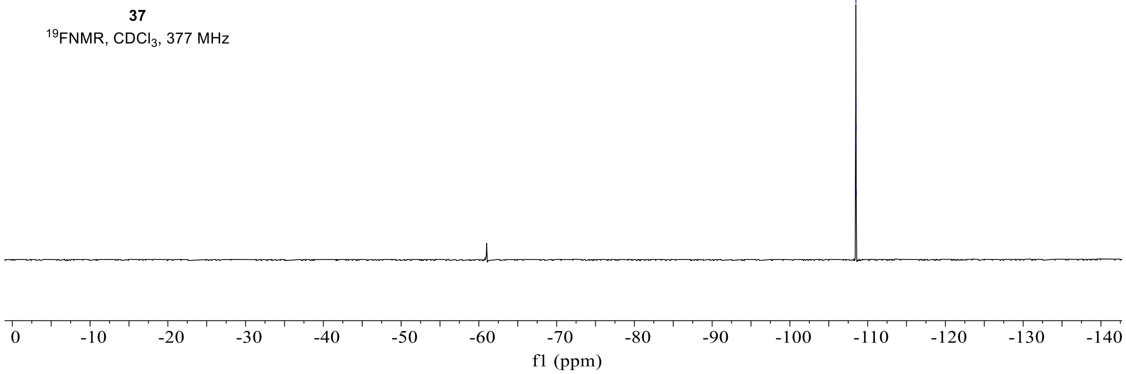

(*R*)-2,6,7,9-tetraphenyl-4-(4-(trifluoromethyl)phenyl)-2,3-diazaspiro[4.4]nona-3,6,8-trien-1-one(38)

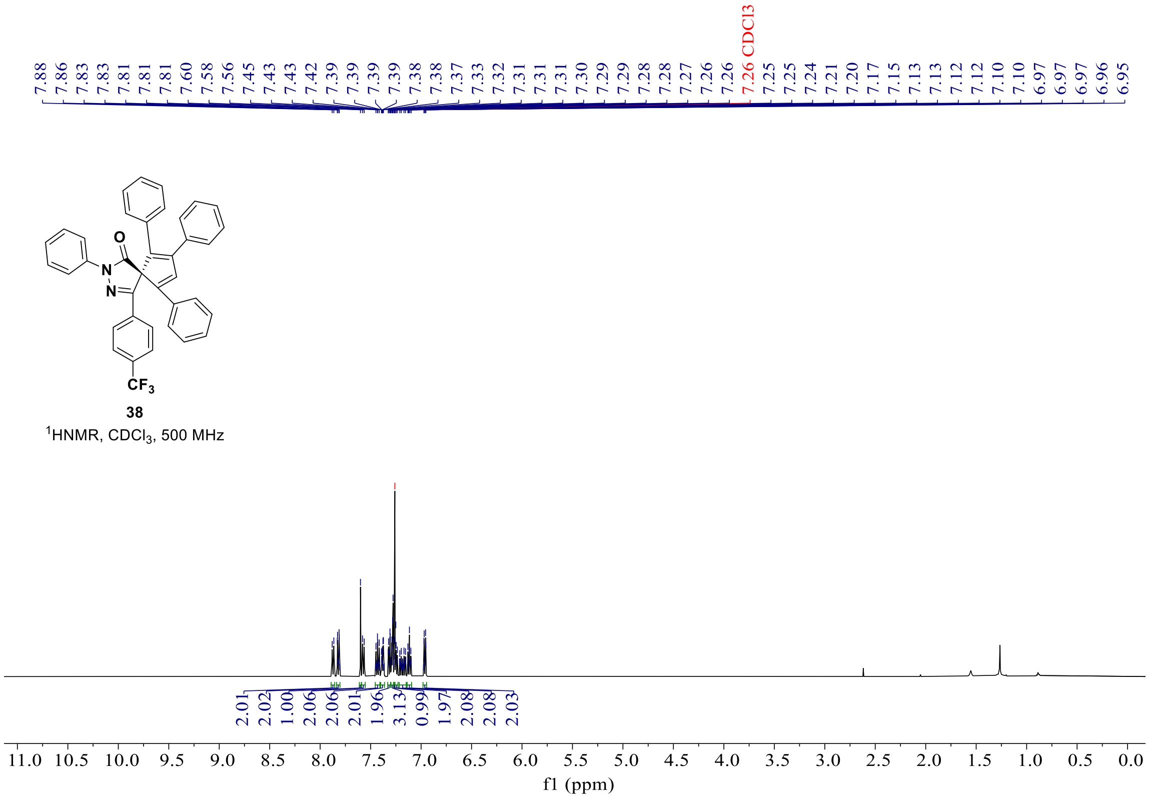

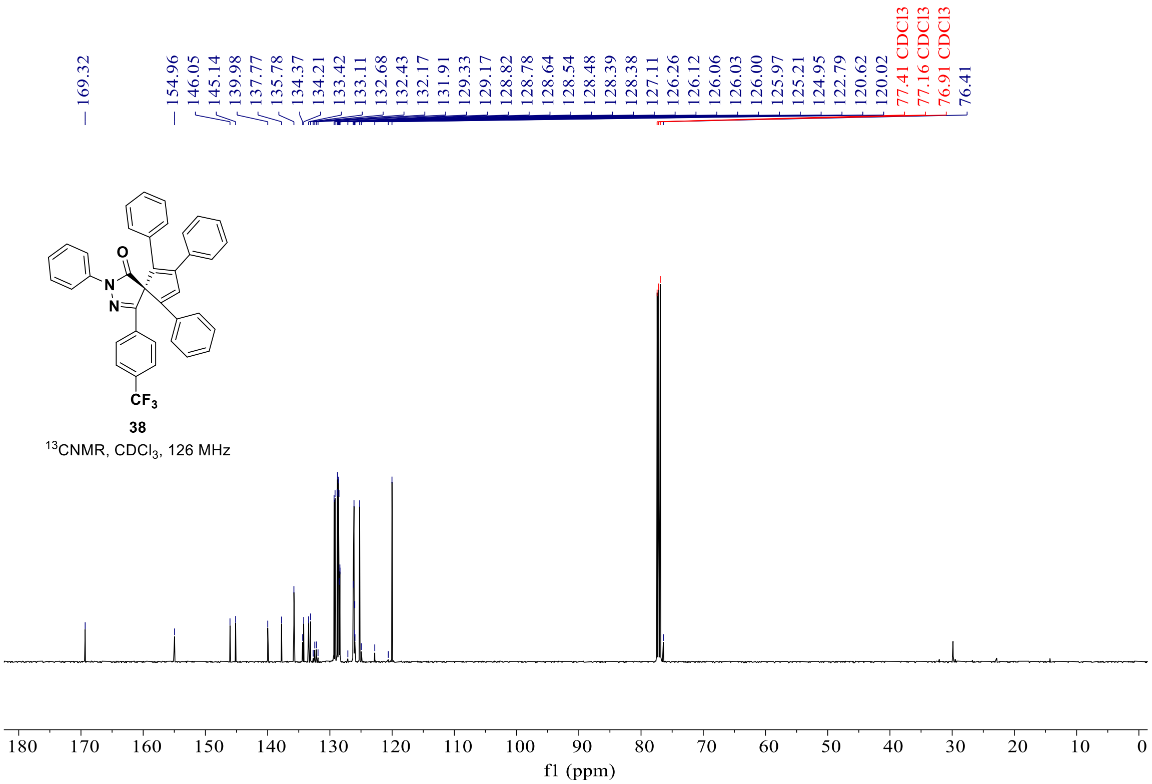

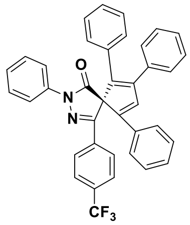

38

^{19}FNMR, $CDCl_3$, 471 MHz

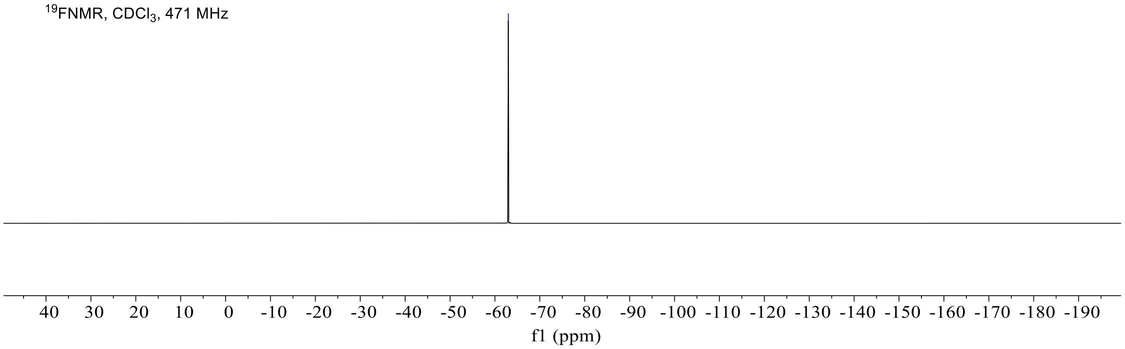

(*R*)-4-(4-methoxyphenyl)-2,6,7,9-tetraphenyl-2,3-diazaspiro[4.4]nona-3,6,8-trien-1-one (39)

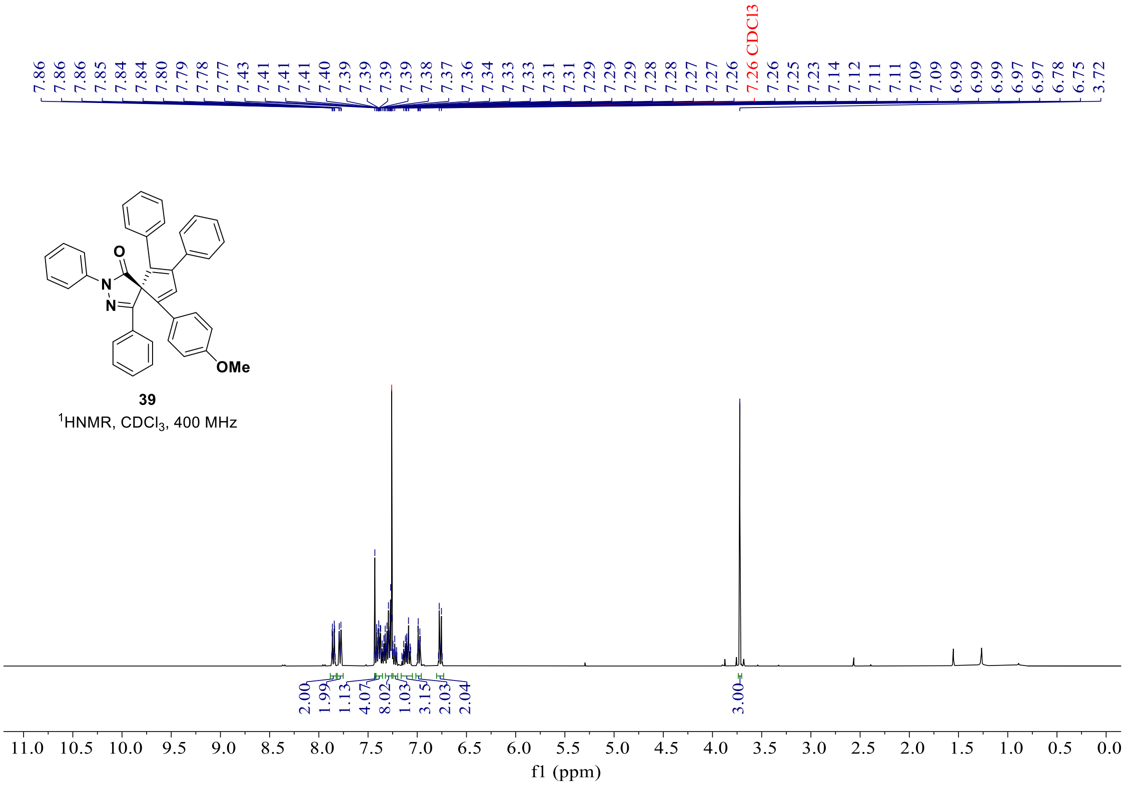

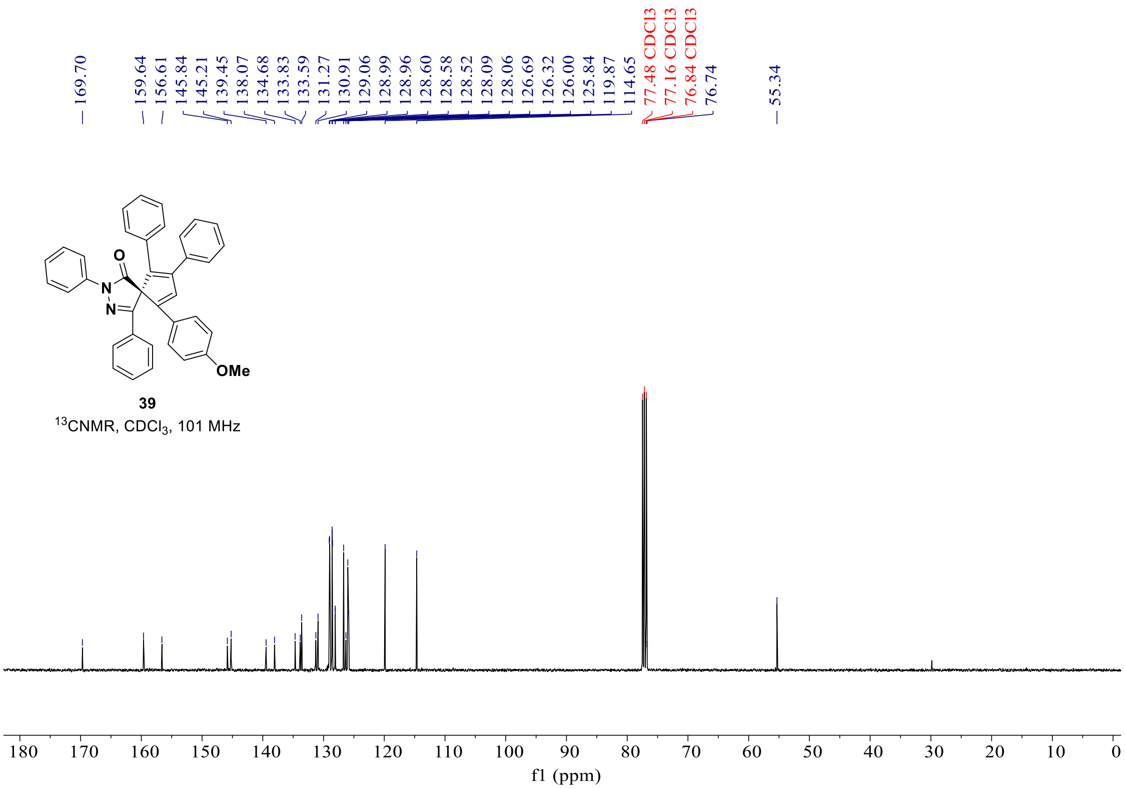

(*R*)-9-(benzo[d][1,3]dioxol-5-yl)-2,4,6,7-tetraphenyl-2,3-diazaspiro[4.4]nona-3,6,8-trien-1-one (40)

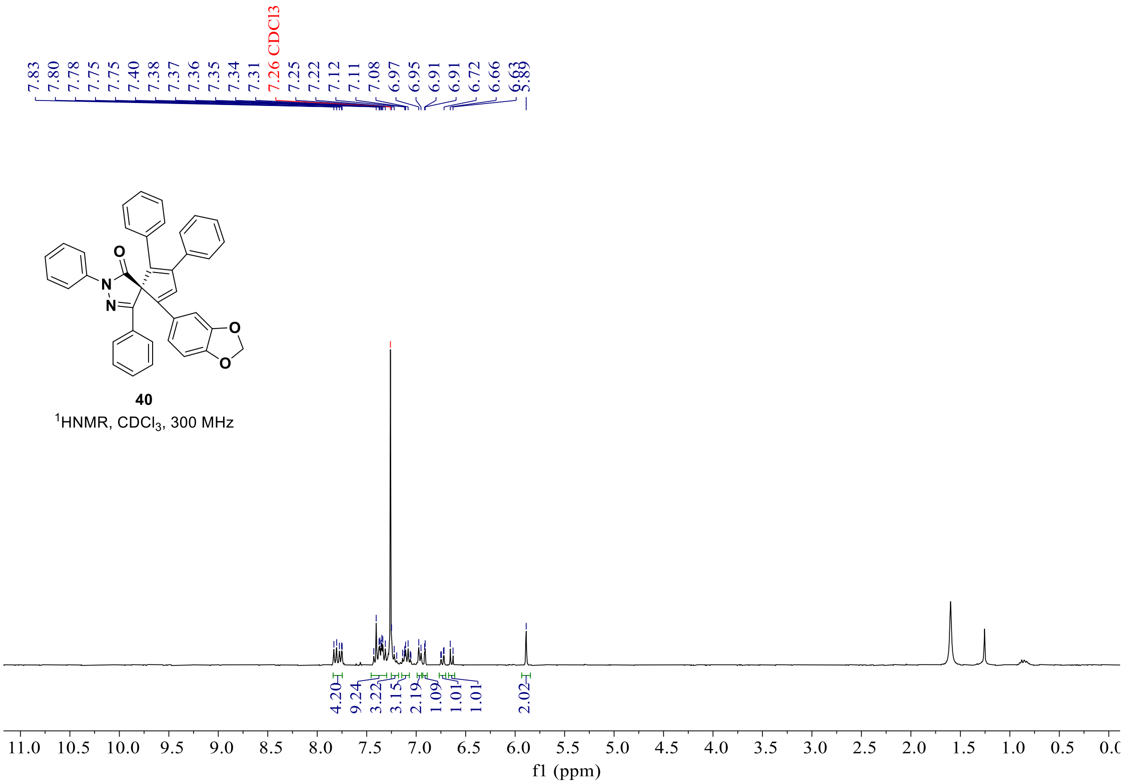

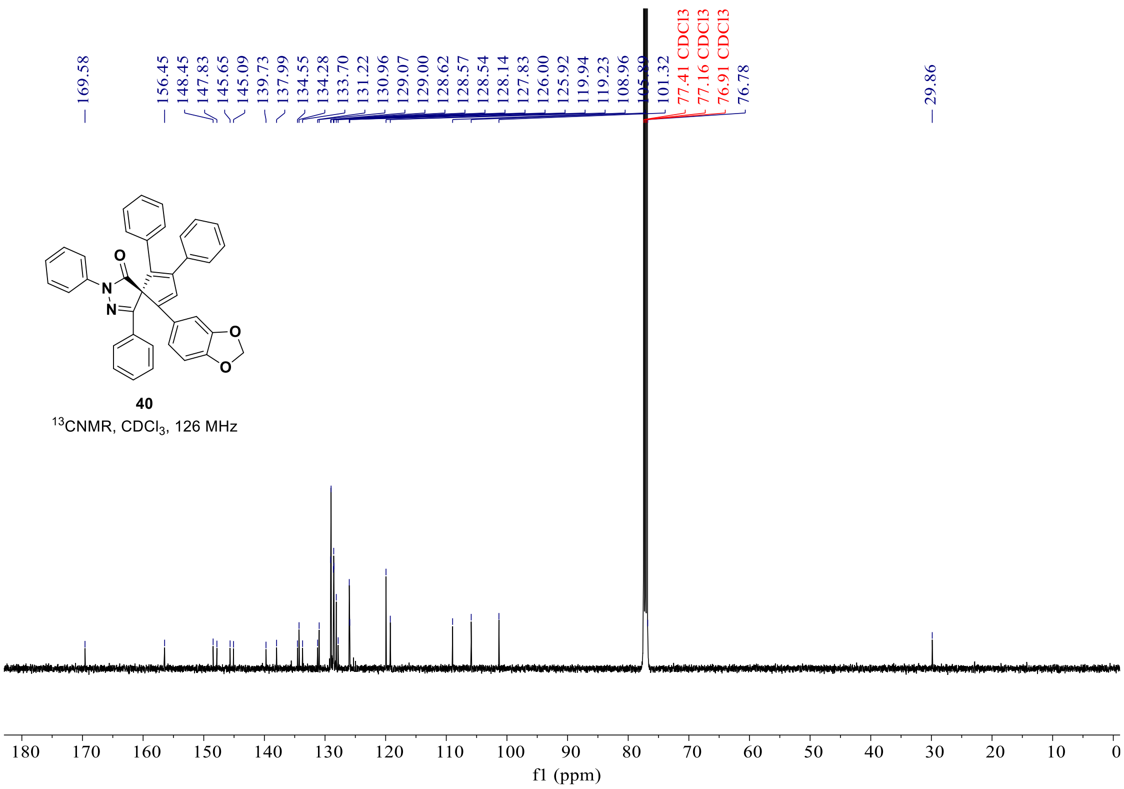

(*R*)-4-methyl-2,6,7,9-tetraphenyl-2,3-diazaspiro[4.4]nona-3,6,8-trien-1-one (41)

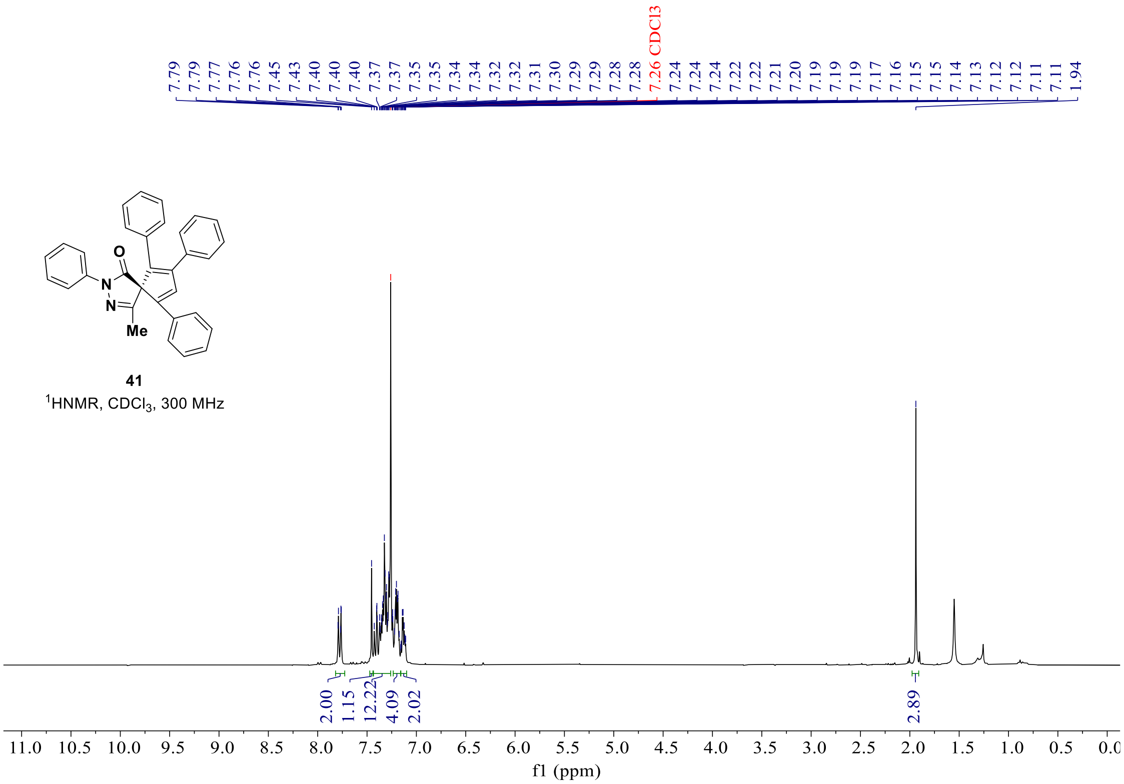

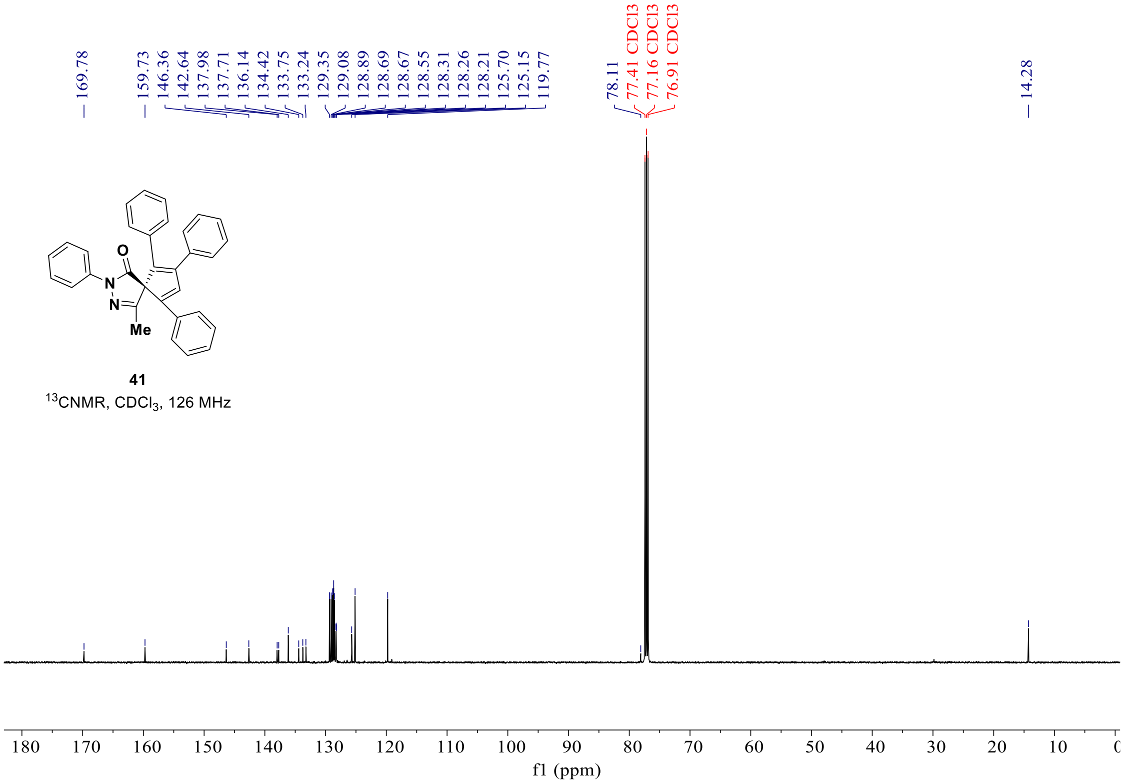

(*S*)-2,4,9-triphenyl-6,7-dipropyl-2,3-diazaspiro[4.4]nona-3,6,8-trien-1-one (42)

42

^{1}HNMR, $CDCl_3$, 500 MHz

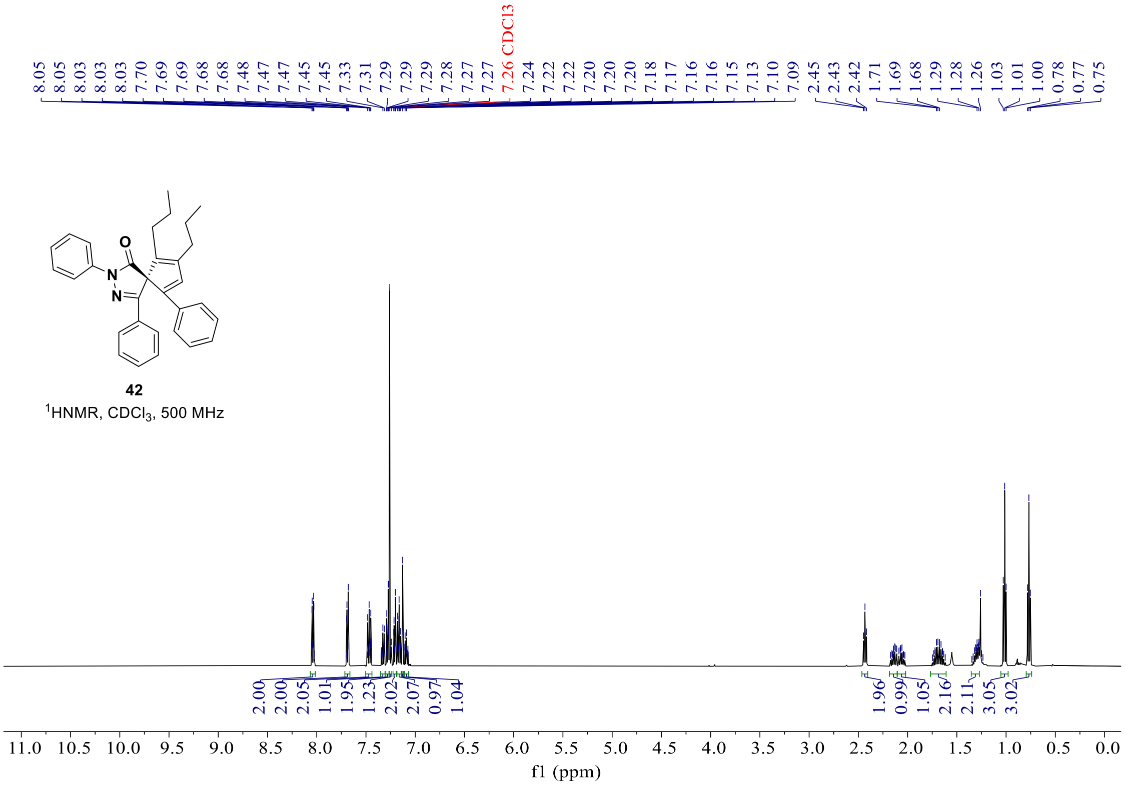

42

^{13}CNMR, $CDCl_3$, 126 MHz

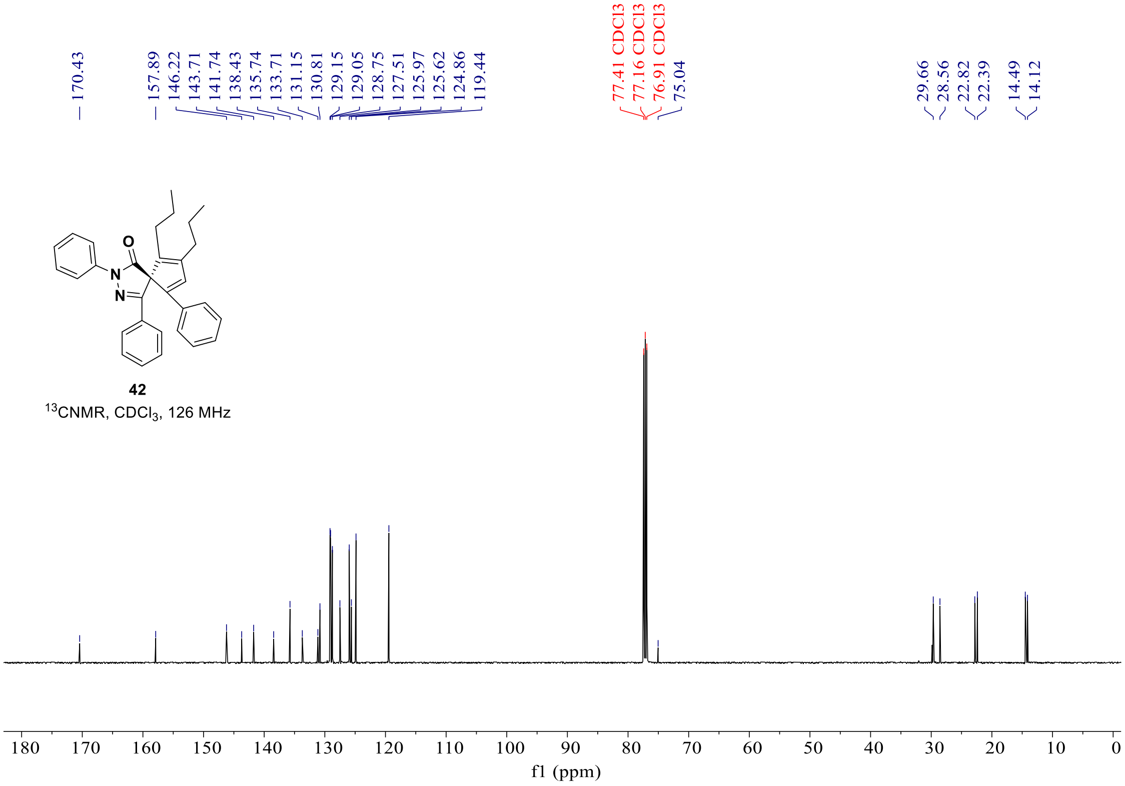

(*R*)-6-(*tert*-butyl)-7-butyl-2,4,9-triphenyl-2,3-diazaspiro[4.4]nona-3,6,8-trien-1-one (43)

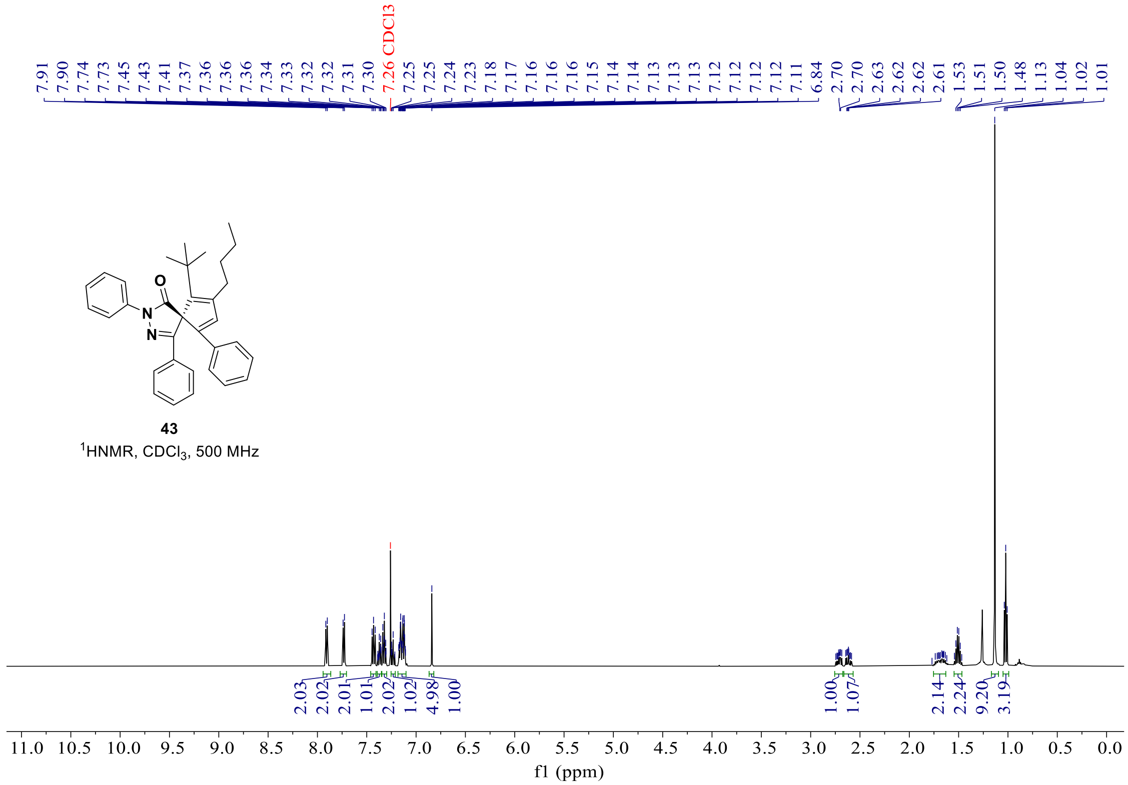

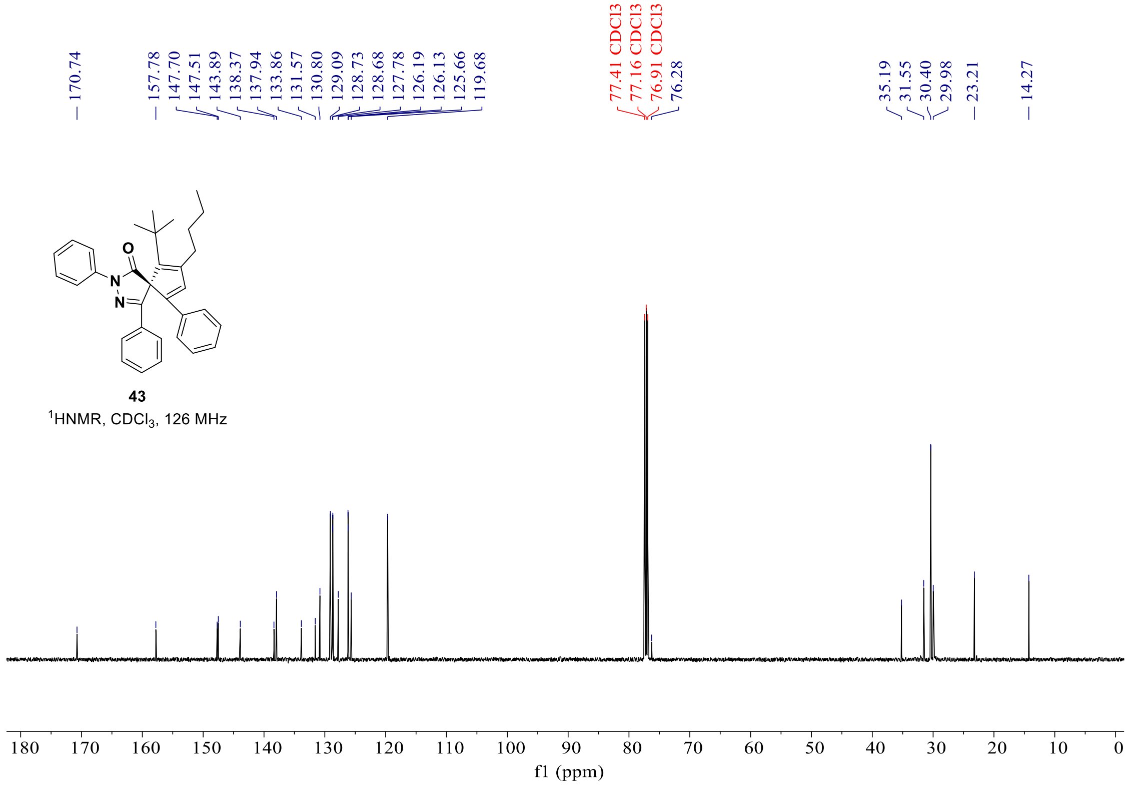

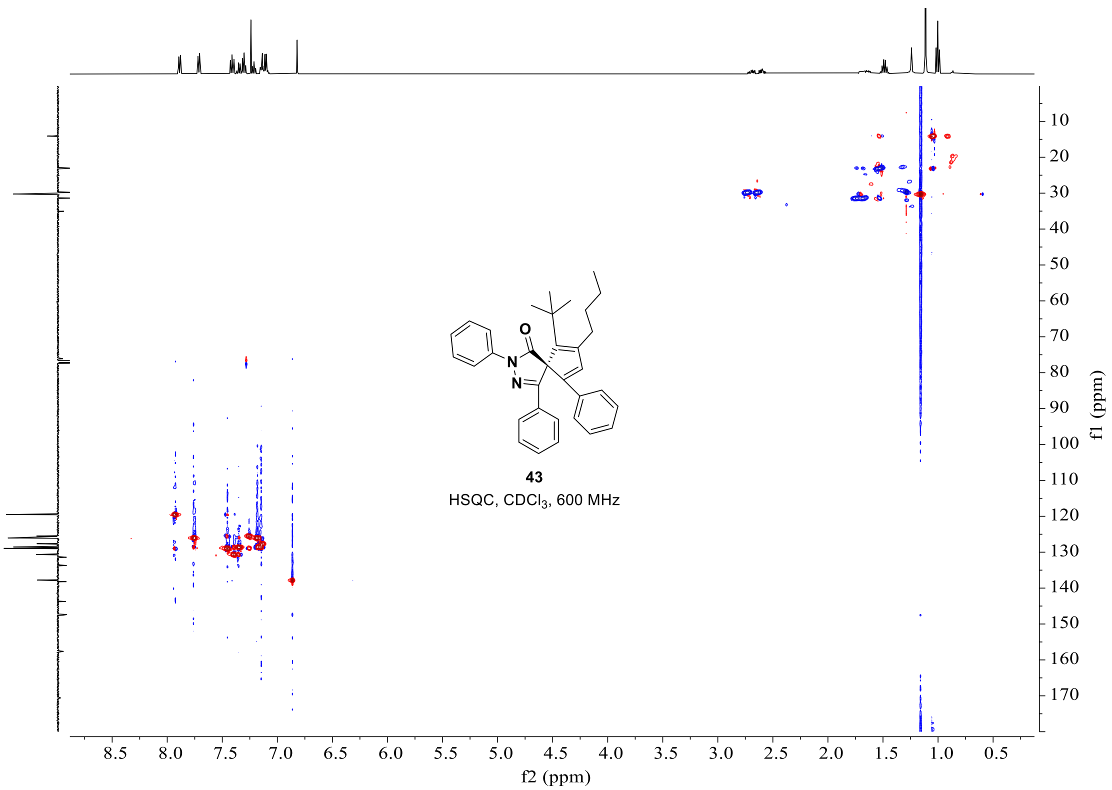

O
N
N
43
HSQC, $CDCl_3$, 600 MHz
f1 (ppm)
f2 (ppm)

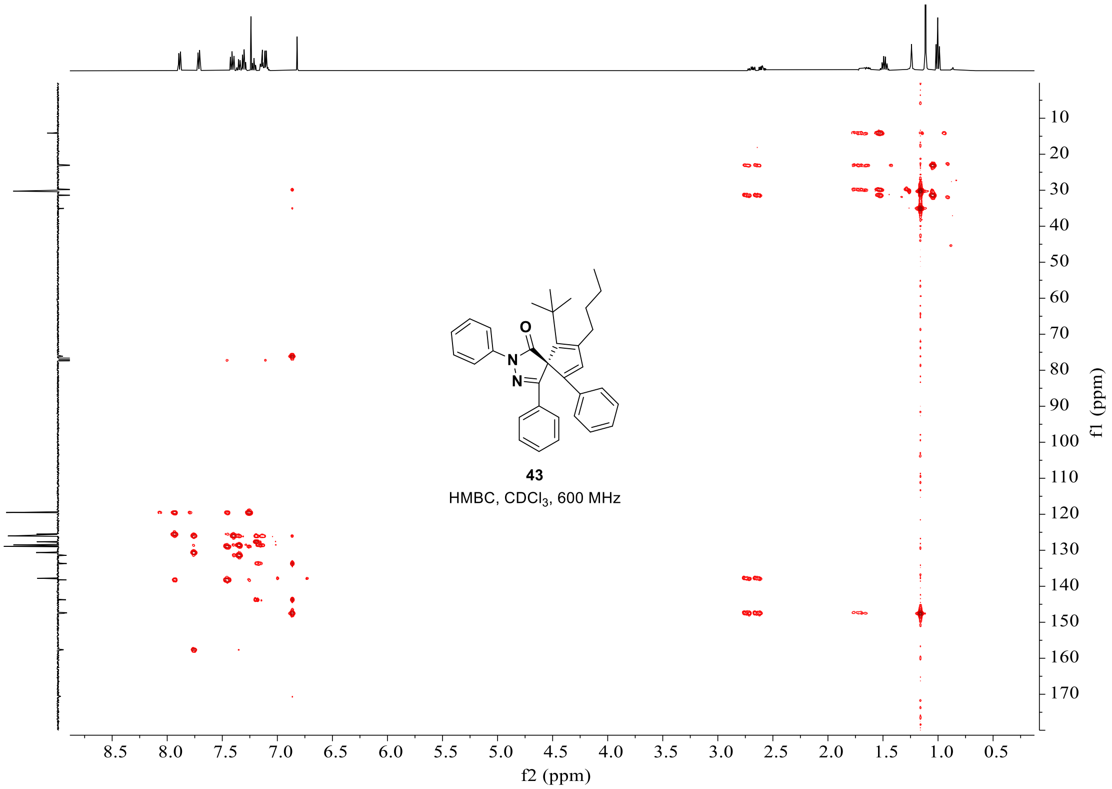

O
N
N
43
HMBC, $CDCl_3$, 600 MHz
f1 (ppm)
f2 (ppm)
